# Exploring transcription factors reveals crucial members and regulatory networks involved in different abiotic stresses in *Brassica napus L.*

**DOI:** 10.1186/s12870-018-1417-z

**Published:** 2018-09-19

**Authors:** Pei Wang, Cuiling Yang, Hao Chen, Longhai Luo, Qiuli Leng, Shicong Li, Zujing Han, Xinchun Li, Chunpeng Song, Xiao Zhang, Daojie Wang

**Affiliations:** 1 Key Laboratory of Plant Stress Biology; School of Mathematics and Statistics; State Key Laboratory of Cotton Biology; College of Life Sciences; Institute of Applied Mathematics; Laboratory of Data Analysis Technology; Henan University, Kaifeng, Henan, 475004, China, Jinming avenue, Kaifeng, 475004 China; 2 Beijing igeneCode Biotech Co.,Ltd, Changping District Xisanqi Center for the Olympic Century, Beijing, 100096 China

**Keywords:** Transcription factor, Abiotic stress, RNA-Seq, Gene regulatory network, *Brassica napus*

## Abstract

**Background:**

*Brassica napus (B. napus)* encompasses diverse transcription factors (TFs), but thorough identification and characterization of TF families, as well as their transcriptional responsiveness to multifarious stresses are still not clear.

**Results:**

Totally 2167 TFs belonging to five families were genome-widely identified in *B. napus*, including 518 BnAP2/EREBPs, 252 BnbZIPs, 721 BnMYBs, 398 BnNACs and 278 BnWRKYs, which contained some novel members in comparison with existing results. Sub-genome distributions of BnAP2/EREBPs and BnMYBs indicated that the two families might have suffered from duplication and divergence during evolution. Synteny analysis revealed strong co-linearity between *B. napus* and its two ancestors, although chromosomal rearrangements have occurred and 85 TFs were lost. About 7.6% and 9.4% TFs of the five families in *B. napus* were novel genes and conserved genes, which both showed preference on the C sub-genome. RNA-Seq revealed that more than 80% TFs were abiotic stress inducible and 315 crucial differentially expressed genes (DEGs) were screened out. Network analysis revealed that the 315 DEGs are highly co-expressed. The homologous gene network in A. thaliana revealed that a considerable amount of TFs could trigger the differential expression of targeted genes, resulting in a complex clustered network with clusters of genes responsible for targeted stress responsiveness.

**Conclusions:**

We identified and characterized five TF families in *B. napus*. Some crucial members and regulatory networks involved in different abiotic stresses have been explored. The investigations deepen our understanding of TFs for stress tolerance in *B. napus*.

**Electronic supplementary material:**

The online version of this article (10.1186/s12870-018-1417-z) contains supplementary material, which is available to authorized users.

## Background

The *Brassica* genus includes a diverse range of vegetable and oilseed crops which are important for human nutrition, such as *Brassica rapa (B. rapa)*, *Brassica oleracea (B. oleracea)* and *B. napus*. The genomes of many species in the *Brassica* genus have been published recently [1–4]. *B. napus* is allopolyploid offspring of *B. rapa* and *B. oleracea* with genome AACC, which inherits sub-genome A from *B. rapa* and C from *B. oleracea*, and is cultivated as one of the most important economic crops [3].

AP2/EREBP, bZIP, MYB, NAC and WRKY consist of five large TF families in eukaryotes, which are ubiquitous and some of which (AP2/EREBP, NAC, WRKY) are unique in plants [5–23]. Different TF families posses featured structure domains or conserved motifs. The distinguishing characteristic of AP2/EREBPs is their AP2 DNA-binding domain [5]. The AP2/EREBPs can be divided into two subfamilies [5, 19]: AP2 and EREBP. The EREBP subfamily can be further split into three subfamilies [19]: DREB, ERF and RAV. The AP2 proteins contain two repeated AP2 domains, and the EREBP proteins contain one AP2 domain. The RAV proteins further contain a B3 domain and the DREB proteins is rich in acidic amino acids in the C-terminal (CT). The bZIP proteins have a basic region that binds DNA and a leucine zipper dimerization motif [7], which can also be classified into several subfamilies according to their amino acid rich domain [24]. The MYB proteins typically go with a conserved MYB DNA-binding domain [10, 12], which can be classified into four subfamilies depending on the number of adjacent repeats in the MYB domain [12]. MYB proteins with one, two and three repeats are denoted as 1R, R2R3 and 3R respectively [12]. Members in the smallest subfamily 4R contain four R1/R2-like repeats [10]. The plant-specific NAC family contains a highly conserved NAC domain at the N-terminal (NT) and a variable CT functioning as the activation domain [13]. The WRKY proteins are featured with their highly conserved WRKY domains and zinc finger motifs C2H2 or C2HC [17, 18], which can be split into three groups [17]. Group I goes with two WRKY domains: the NT and CT domains [17]. Both group I and II have the same zinc finger motif C2H2. Group II proteins can be further divided into IIa to IIe based on the additional amino acid motifs outside the WRKY domain and their phylogenetic distance [17]. Proteins with one WRKY domain and a C2HC zinc finger motif are categorized into group III.

Various abiotic stresses or hormone stimulus, such as low temperature, heat, drought, salinity and abscisic acid (ABA), can crucially affect the growth and production of plants [25, 26]. TFs can regulate the stimulus responsiveness of plants [27]. Increasingly works and databases have focused on the identification and characterization of TF families. The TAIR [28], BRAD [29] and PlantTFDB [27] have collected tens to hundreds of TFs for each family. Additionally, some works have reported that the AP2/EREBPs, bZIPs, MYBs, NACs and WRKYs play important roles during stimulus responsiveness [5, 7, 9, 11, 14, 24, 30, 31]. For example, Du et al. [30] identified 132 BnAP2/ERF genes by RNA-Seq of rapeseed leaves exposed to low temperature, and it was reported that the 132 BnAP2/ERFs involved several molecular pathways that may enhance cold resistance in plants. Hwang et al. identified 136 BrbZIPs [31] in *B. rapa* and 119 BobZIPs [24] in *B. oleracea*, and cold responsiveness of these TFs were investigated by using RNA-Seq and qRT-PCR. Zhao et al. [32] found that the expression of a bZIP gene, BnABF2 in *B. napus*, could be induced by drought and salt stresses. Zhou et al. [8] identified 247 BnbZIPs in *B. napus* and performed detailed structural analyses. Chen et al. [33] identified 76 R2R3 MYBs in the *B. napus* genome through excavating expressed sequence tags (ESTs), and the transcript abundance level analysis during abiotic conditions and ABA treatment identified a group of MYBs that responded to one or more treatments. Recently, Hajiebrahimi et al. [34] identified 249 R2R3 MYBs in *B. napus*, and the authors reported that these genes were highly expressed in the roots under high salinity treatment after 24 h. Three MYB genes, BnaMYB21, BnaMYB141, and BnaMYB148 have been suggested improve salt-tolerance in *B. napus*. For the NAC family, Wang et al. [35] identified 60 BnNACs in *B. napus* through excavating ESTs. Two *B. napus* genes encoding NACs, BnNAC2 and BnNAC5 were reported that they can respond to high-salinity stress [15]. A specific BnNAC gene in *B. napus*, BnNAC55, was reported to be a novel transcriptional activator modulating reactive oxygen species accumulation and cell death [16]. The WRKYs can mediate diverse stimulus responsiveness in plants [17, 18, 36–38]. For example, in *B. napus*, Yang et al. [39] identified 46 BnWRKYs by excavating the EST database, 13 BnWRKYs responding to both fungal pathogens and hormone stimuli are identified. Recently, Wu et al. [40] identified 289 BnWRKYs, while He et al. [41] identified 287 BnWRKYs in *B.napus*. Wang et al. [42] isolated a WRKY gene–BnWRKY33, and it was found to be highly responsive to *S. sclerotiorum* infection in *B. napus*.

Although the five TF families have been extensively investigated, their concrete numbers are still debated, especially for the relatively rarely investigated *B. napus*. Moreover, thoroughly understanding of the regulatory networks of these TFs responding to various stresses/stimuli are also a vacancy. Motivated by the arising questions, genome-wide identification of the five TF families in *B. napus* will be performed. Evolutionary analysis of TFs in *B. napus* with related species will be explored. Moreover, we will investigate the responsiveness of the five TF families under diverse stresses/stimuli based on RNA-Seq.

## Results

### Genome-wide identification and chromosome distributions of five TF families

We genome-widely identified AP2/EREBPs, bZIPs, MYBs, NACs and WRKYs in *A. thaliana*, *B. rapa*, *B. oleracea* and *B. napus* by following rigorous procedures (Materials and methods, Additional file 1). The identified numbers of genes and transcripts as well as databases reporting ones, are summarized in Table 1. The *B. napus* genome encompasses 518 BnAP2/EREBPs, 252 BnbZIPs, 721 BnMYBs, 398 BnNACs and 278 BnWRKYs. The *B. rapa* genome encompasses 283 BrAP2/EREBPs, 126 BrbZIPs, 348 BrMYBs, 198 BrNACs and 141 BrWRKYs. While the TF numbers of *B. oleracea* are 282, 127, 370, 210 and 142 respectively (Additional file 2). Compared with *B. rapa* and *B. oleracea*, the number of TFs is almost doubled in *B. napus*. Moreover, the identified results are more or less different from the existing databases, which include some novel members (Additional file 1).

**Table 1:** The numbers of genes and transcripts for the five TF families in *A. thaliana*, *B. rapa, B. oleracea* and *B. napus*

Species	Sources	AP2/EREBP		bZIP		MYB		NAC		WRKY	
		Genes	Transcr.	Genes	Transcri.	Genes	Transcr.	Genes	Transcr.	Genes	Transcr.
*A. thaliana*	Our	141	170	72	120	196	269	112	137	71	84
	PlantTFDB	146	176	74	127	144	168	113	138	72	90
	TAIR	149	149	73	73	131	131	96	96	72	74
*B. rapa*	Our	283	283	126	126	348	348	198	198	141	141
	PlantTFDB	323	323	200	200	293	293	256	256	180	180
	BRAD	289	289	127	127	368	368	188	188	147	147
*B. oleracea*	Our	282	282	127	127	370	370	210	210	142	142
	PlantTFDB	317	317	217	217	306	306	271	271	191	191
*B. napus*	Our	518	518	252	252	721	721	398	398	278	278
	PlantTFDB	533	533	264	264	740	740	411	411	285	285

**Fig. 1:**
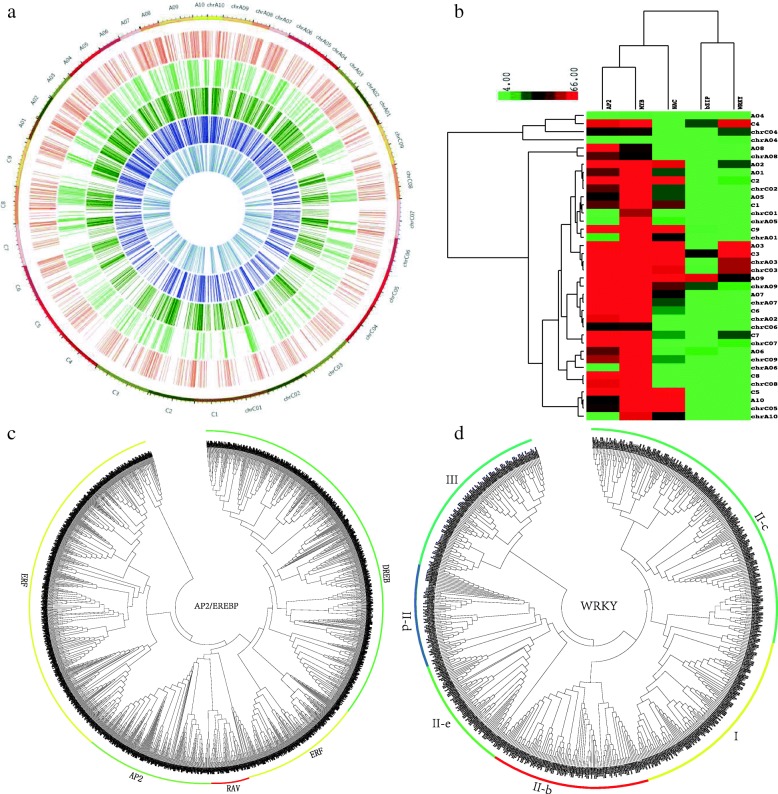
Chromosome distributions and phylogenetic trees of TF families. **a** Circos plot of the five TF families in *B. rapa* (A01-A10) and *B. oleracea* (C1-C9) and *B. napus* (ChrA01-ChrA10, ChrC01-ChrC09). From outside to inside of the circos plot, we show the three genomes, AP2/EREBPs, bZIPs, MYBs, NACs and WRKYs respectively. **b** Hierarchical clustering analysis for numbers of TFs that are anchored to chromosomes of the three species. Correlation coefficient and the average linkage method are used to evaluate the closeness among samples and clusters. **c** The unrooted phylogenetic tree for BnAP2/EREBPs in *B. napus* in comparison to those in *A. thaliana*, *B. rapa* and *B. oleracea*. **d** The unrooted phylogenetic tree for WRKYs

Most of the identified TFs can be anchored onto chromosomes (Fig. 1a), while 105 BnAP2/EREBPs, 45 BnbZIPs, 151 BnMYBs, 72 BnNACs and 46 BnWRKYs are mapped onto unanchored scaffolds according to the current databases. The distributions of bZIPs, MYBs, NACs and WRKYs on the 10 chromosomes of the A sub-genome in *B. napus* are not significantly different from those in *B. rapa* (*χ*^2^ test [43], *p*>0.05. Additional file 1). However, such distribution in *B. napus* is significantly different from *B. rapa* (*p*=0.0253<0.05) for AP2/EREBPs. For the 9 chromosomes of the C sub-genome, distributions of AP2/EREBPs (*p*=0.0140) and MYBs (*p*=0.0071) in *B. napus* are all significantly different from those in *B. oleracea*, while distributions of other three TF families in the two species don’t have statistical differences (*p*>0.05). Moreover, compared with different species and different chromosomes, the AP2/EREBPs and the MYBs have the highest similarity on chromosome distributions, while the similarity among bZIPs, WRKYs and other three families are relatively lower (Fig. 1b). Furthermore, amounts of TFs on some chromosomes in *B. napus* tend to be proportional with those on the corresponding chromosomes of ancestral genomes (Fig. 1b). For example, the four chromosomes chrA04, chrC04, A04 and C04 can be clustered as one group.

### Phylogenetic analysis and subfamily distributions

The phylogenetic trees analysis revealed that TFs are structurally clustered (Fig. 1c and d for AP2/EREBPs and WRKYs. See Additional file 1 for other families). For each family, the phylogenetic tree resolves into several clades based on their primary amino acid sequences. The AP2/EREBPs can be divided into four subfamilies: AP2s, RAVs, ERFs and DREBs. The DREBs and ERFs in *B. napus* were further distributed into six subgroups, A1 to A6, B1 to B6 respectively. More than 10% AP2/EREBPs in the three *Brassica* species belong to ERF B3, DREB A4 and ERF B1 respectively. However, more than 15% AtAP2/EREBPs in *A. thaliana* are classified into ERF B6. Specifically, for *B. napus*, we find that a total of 194 DREBs were distributed into groups A1- A6, containing 17, 30, 3, 66, 42 and 36 genes respectively. Additionally, 244 ERFs were distributed into groups B1-B6 containing 61, 18, 76, 21, 25, and 43 genes respectively, and the rest 28 ERFs can not be assigned to any specific subgroups. Finally, 32 genes were classified into the AP2 subfamily and 20 genes are assigned into the RAV subfamily. The AtbZIPs can be classified into 7 subfamilies, while the bZIPs in *B. rapa, B. oleracea* and *B. napus* can be divided into 9, 11 and 12 subfamilies. Almost 50% AtbZIPs (59) belong to subfamily I, while only 12 BnbZIPs are subfamily I. Most of the MYBs in the four species belong to the 1R and R2R3 super-families. The AtNACs can be classified into 14 subfamilies, and those in the three *Brassica* genus can be divided into 21, 16 and 17 subfamilies. More than 20% BoNACs belong to subfamilies X and IV respectively. The BnNACs is the most rich in subfamily VI members. Among the 278 BnWRKYs, 49 TFs belong to subfamily I, and 183 TFs are part of subfamily II, while 46 TFs are classified into subfamily III. More than 25% WRKYs in subfamily II belong to group IIc. Interestingly, no group IIa BnWRKYs were found.

### Cis-elements analysis

**Fig. 2:**
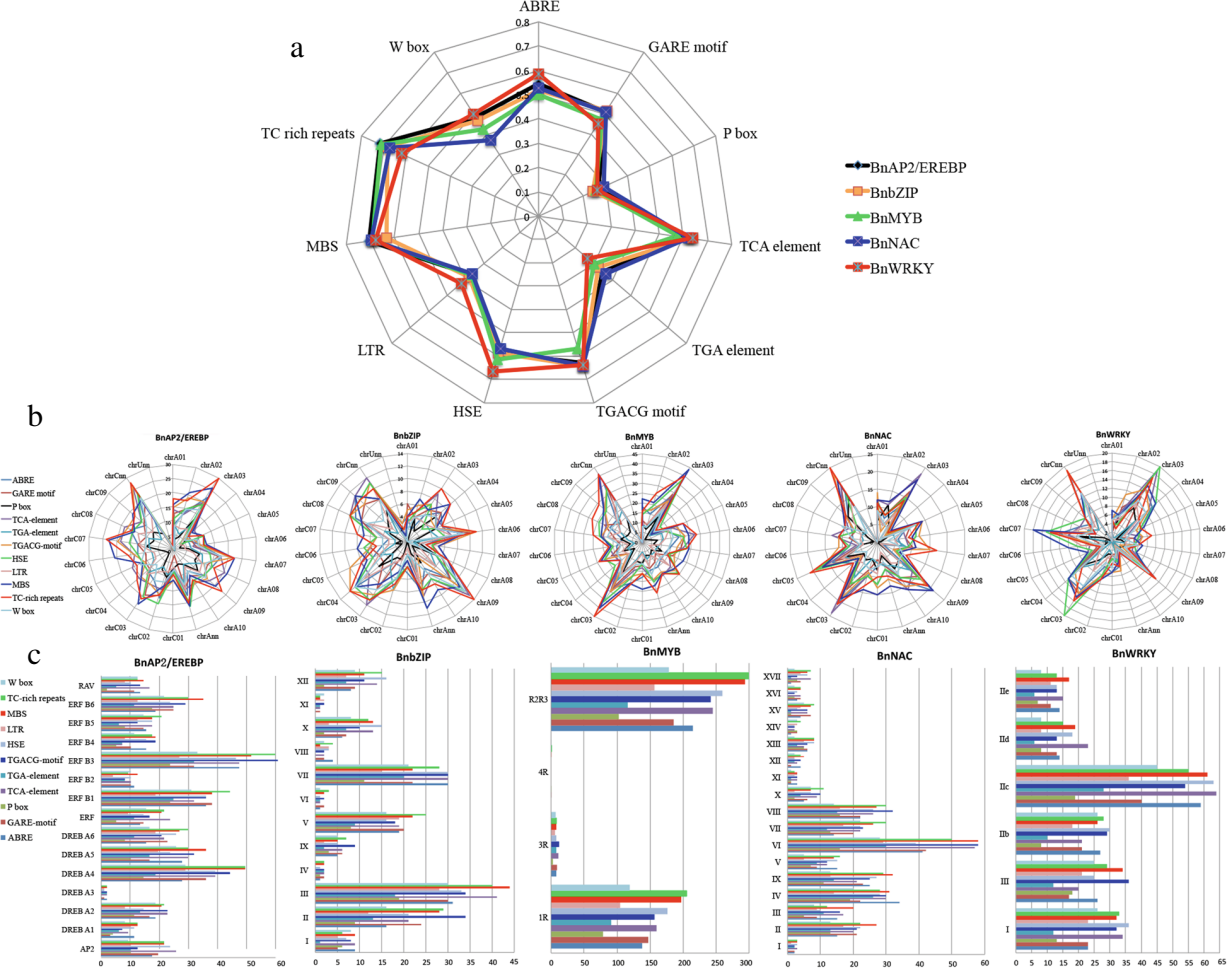
Statistical analysis for cis-elements of TFs in *B. napus*. **a** Proportions of genes with various cis-elements for each family. **b** Chromosome distributions. **c** Subfamily distributions

TFs can bind with promoters of regulated genes and control their expression under treatment [44]. Cis-elements are essential to temporal, spatial, and cell type-specific control of gene expression [25, 45]. In *B. napus*, the promoters of the five families of TFs contain various cis-elements (Fig. 2), such as HSE, MBS and ABRE (Additional file 1). For the five families, proportions of TFs with P-box, TGA-element and LTR in their promoters are all very low. Whereas, more than 60% BnNACs and BnWRKYs contain TCA-element, TGACG-element, MBS and TC-rich repeats in their promoters. Besides, compared with other families, promoters of the BnWRKYs posses the most LTR, ABRE and HSE elements. Chromosome distributions show that different cis-elements prefer certain chromosomes (Fig. 2b). For example, BnAP2/EREBP with TC-rich repeats in their promoters are enriched in chrA03 and chrC07. Many BnMYBs, BnNACs and BnWRKYs with cis-elements in their promoters are enriched in chrA03 and chrC03. Cis-elements in promoters of BnbZIPs are rich in chrA09, chrC03 and chrC04. It also shows that some chromosomes always contain rare cis-elements, such as chrC01. Subfamily distributions show that different cis-elements prefer certain subfamilies (Fig. 2c). For example, cis-elements in promoters of BnAP2/EREBPs are enriched with ERF B3, B1 and DREB A4. All subfamilies of BnWRKYs contain considerable numbers of cis-elements in their promoters, among which, group IIc are overwhelmingly higher than other subfamilies.

### Synteny analysis

**Fig. 3:**
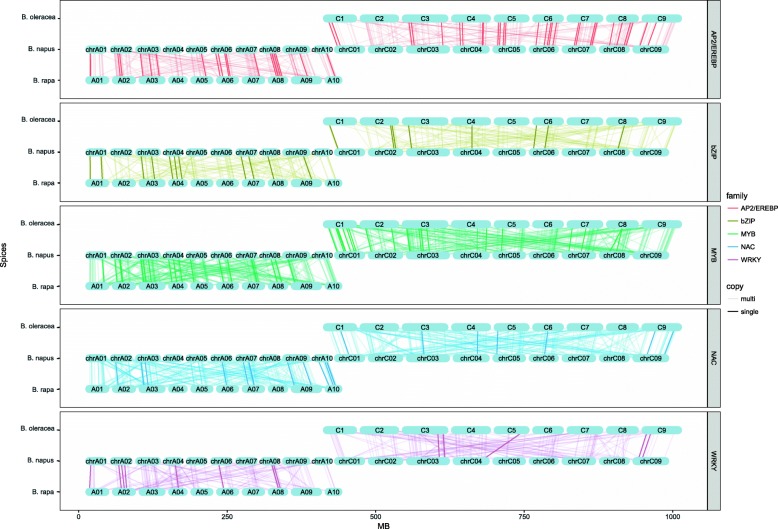
Synteny maps show orthologous relationships between TFs in *B. napus*, *B. rapa* and *B. oleracea*. Genes in the three species are plotted against their predicted linked counterparts

Synteny analysis of TFs in the three *Brassica* genus show strong co-linearity, although chromosomal rearrangements and gene duplication events have occurred between *B. napus* and ancestral species after their divergence from the common ancestor (Fig. 3, Additional files 1 and 3). Synteny analysis allows us to characterize four categories of genes: 1) Novel genes, which emerged in *B. napus* but not in ancestral species. 2) Conserved genes, which have a unique copy from *B. rapa, B. oleracea* to *B. napus*. 3) Multi-copy genes. If two or more genes from the same species were detected in a homologous gene set of the four species [14]. 4) Lost genes [14], which were found in ancestral genomes but not in *B. napus*. There are 33, 22, 65, 29, 19 novel, and 66, 26, 54, 30, 24 conserved BnAP2/EREBPs, BnbZIPs, BnMYBs, BnNACs and BnWRKYs among the identified TFs in *B. napus* respectively. While 419 BnAP2/EREBPs, 204 BnbZIPs, 602 BnMYBs, 339 BnNACs and 235 BnWRKYs are multi-copy genes. Novel genes averagely take up about 7.6%. About 9.4% TFs in the five families are conserved in *B. napus*, including 12.7% BnAP2/EREBPs, and about 7.5% BnMYBs and BnNACs. More than 80% TFs in *B. napus* are multi-copy ones. For the A sub-genome, a total of 30 TFs lost during the evolution, including 12 BrAP2/EREBPs, 7 BrbZIPs, 8 BrMYBs, 1 BrNACs and 2 BrWRKYs. For the C sub-genome, a total of 55 TFs lost, including 14 BoAP2/EREBPs, 5 BobZIPs,16 BoMYBs,16 BoNACs and 4 BoWRKYs.

Statistical analysis reveals that novel, conserved and lost genes prefer the C sub-genome, while multi-copy genes show no sub-genome preferences (Additional file 3). Specifically, 36 BnMYBs are novel genes, and 44 BnAP2/EREBPs are conserved genes, which are all positioned on the C sub-genome, and take up the most, compared with other families and the A sub-genome. For multi-copy genes, both the A and C sub-genomes contain about 40% members in each family, and show no sub-genome preferences. Statistical analysis also reveals that the four categories of genes show subfamily preferences. In fact, one third of novel genes in BnAP2/EREBPs belong to ERF B3. Subfamilies DREB A4 (16) and ERF B3 (15) contain the most conserved BnAP2/EREBPs, while ERF B1(54) and ERF B3 (50) posses the most multi-copy genes. Seven of the 12 lost BrAP2/EREBPs and 9 of the 14 lost BoAP2/EREBPs belong to the ERF subfamily. Subfamily VII of bZIPs is rich in novel genes, V and XII are rich in conserved genes, III is enriched with multi-copy genes, while the 12 lost bZIPs from *B. rapa, B. oleracea* to *B. napus* show no subfamily preferences. The R2R3 BnMYBs contains the most novel, conserved and multi-copy genes. Whereas, 17 of the 24 lost MYBs belong to the 1R, indicating that the lost MYBs especially prefer subfamily 1R. Subfamilies VI, II and IV of BnNACs all contain relative much more novel genes. IV contains 12 conserved genes, and VI contains 70 multi-copy genes. Only 1 BrNAC is lost from *B. rapa* to *B. napus*, while 16 BoNACs are lost from *B. oleracea* to *B. napus*, and 7 of the 16 BoNACs belong to the IV subfamily, 5 and 4 members are positioned on chromosomes C5 and C2 respectively. Subfamily IIc of BnWRKYs contains the most novel genes, conserved genes and multi-copy genes, and their numbers are 10, 13 and 66 respectively. Among the 6 lost WRKYs, 5 are from subfamily II, and only one is from subfamily III.

### Expression profiles of TFs under five treatments in *B. napus* by RNA-Seq

**Fig. 4:**
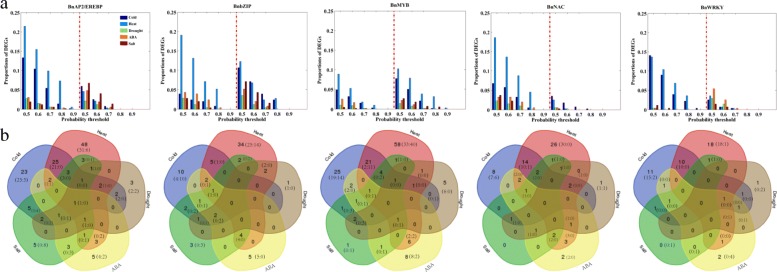
Statistical analysis of DEGs in *B. napus*. **a** Proportions of DEGs under various probability thresholds for each family under each treatment. In each panel, the left half shows the case for up-regulated DEGs, while the right half is for down-regulated ones. **b** Venn diagrams for DEGs under the five treatments with cut-off probability 0.6. Numbers in brackets show the corresponding numbers of DEGs in Venn diagrams for up/down regulated DEGs

The five TF families play important roles during stress response in plants [30, 32, 35, 40]. For *B. napus*, we consider five treatments, including cold, heat, drought, salt and ABA (**Material and methods**). The 7-days-old seedlings were treated under each condition lasting 12 hours for transcriptome sequencing. Three repeats were considered under each treatment. Totally 18 samples were sequenced using Illumina Hiseq 4000 platform, including 15 treated samples and 3 non-treated control samples. On average, we generated about 6.56Gb bases from each sample. Averagely 72.09% clean reads are mapped to reference genome. After mapping sequenced reads to reference genome and reconstruct transcripts, we finally identify 53948 genes on average for each sample, including 50815 known genes and 3133 novel genes on average (Additional file 1). RNA-Seq data reveals that 449 of the 518 BnAP2/EREBPs, 227 of the 252 BnbZIPs, 585 of the 721 BnMYBs, 332 of the 398 BnNACs, 241 of the 278 BnWRKYs can respond to the five treatments (Additional file 4). For each of the five families, bar plots for the numbers of up and down regulated DEGs [46, 47] (**Material and methods**) under the five conditions with various cut-off probabilities are shown in Fig. 4. Under cutoff probability 0.6, the numbers of unique DEGs at least under one treatment are 140, 75, 141, 68 and 50 in the five families respectively.

Statistical analysis of responsive genes reveals that the numbers of DEGs quickly decrease with the increasing of probability thresholds. Moreover, DEGs in different TF families show diverse expression patterns. For BnAP2/EREBPs, under given thresholds, the number of up-regulated heat responsive DEGs is the highest, following by cold responsive ones. Whereas, for down-regulated DEGs, it shows that the amount of salt responsive DEGs is the highest. For BnbZIPs and BnMYBs, both up and down-regulated DEGs under heat treatment are higher than those under other treatments. However, the number of down-regulated BnMYBs is relatively higher than up-regulated ones under cold, heat, drought and salt treatments. The numbers of down-regulated salt responsive BnNACs are very low. The amounts of up-regulated BnWRKYs with responsive to cold or heat are higher than those under other three conditions. While under ABA stimulus, down-regulated DEGs are more than other cases. Furthermore, drought and salt responsive down-regulated BnWRKYs are both higher than up-regulated ones.

To verify whether there are DEGs that can respond to multiple treatments for the unique 140 BnAP2/EREBPs, 75 BnbZIPs, 141 BnMYBs, 68 BnNACs and 50 BnWRKYs under threshold 0.6, Fig. 4 shows the corresponding Venn diagrams. It shows that common DEGs under the five treatments are very low, each family has no more than 1 common DEGs. Whereas, common DEGs under two treatments are considerable, especially for those responding to cold and heat stresses, which are 25, 5, 21,14 and 10 in the five families respectively. Moreover, most of the drought, ABA and salt responsive DEGs can simultaneously respond to other stresses/stimuli. For example, 3 of the 4 drought responsive BnWRKYs can also respond to cold, heat or ABA. The observed results of all DEGs are also applicable for solely up-regulated or solely down-regulated ones. Therefore, we conclude that some TFs can respond to multiple treatments, especially for those inter-correlated stresses/stimuli.

### Exploring crucial TFs in *B. napus* from RNA-Seq

**Fig. 5:**
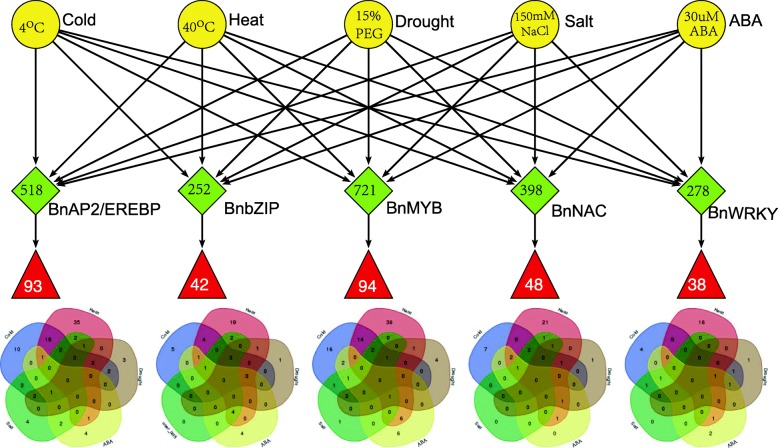
Identification of crucial DEGs and the corresponding Venn diagrams. Based on RNA-Seq data under five treatments and GO annotations, 315 crucial DEGs are screened out. The Venn diagrams show the numbers of responsive crucial DEGs under the five treatments

With cutoff probability 0.6 and |*log*2(*fold* *change*)|≥1, we further identify crucial responsive DEGs of each family. Our selection criterion is based on a prior GO biological process annotations. Genes responding to a certain kind of stimulus are usually found to be rich in certain biological processes or pathways [5, 7, 20, 48–50]. For example, the WRKYs have been reported to play significant roles in development and responding to stimuli [50]. Next, based on GO annotations, we manually filter crucial DEGs of each family (Fig. 5). The finally identified crucial DEGs include 93 BnAP2/EREBPs, 42 BnbZIPs, 94 BnMYBs, 48 BnNACs and 38 BnWRKYs (Additional file 5). The amounts of DEGs from different families that can respond to the same treatment are varied (Fig. 5). Among the identified 315 DEGs, totally 131 DEGs are cold responsive ones, including 41 BnAP2/EREBPs, 11 BnbZIPs, 39 BnMYBs, 22 BnNACs and 18 BnWRKYs. However, 62 BnAP2/EREBPs, 32 BnbZIPs, 61 BnMYBs, 40 BnNACs and 27 BnWRKYs are heat responsive ones. Only 30 DEGs are drought responsive ones, including 11 BnAP2/EREBPs, 2 BnbZIPs, 9 BnMYBs, 4 BnNACs and 4 BnWRKYs. For the salt stress, the number of crucial DEGs in the five families are 17, 7, 9, 5 and 2 respectively. Totally 46 DEGs can respond to ABA stimulus, which consist of 9 BnAP2/EREBP, 10 BnbZIPs, 16 BnMYBs, 5 BnNACs and 6 BnWRKYs. Apparently, heat responsive DEGs take up the most (> 70*%*), followed by cold responsive ones (> 41*%*). However, the numbers of drought, salt or ABA responsive crucial DEGs are very low in each family. For instance, there are only 2 drought responsive BnbZIPs, 4 drought responsive BnWRKYs and 2 salt responsive BnWRKYs.

**Fig. 6:**
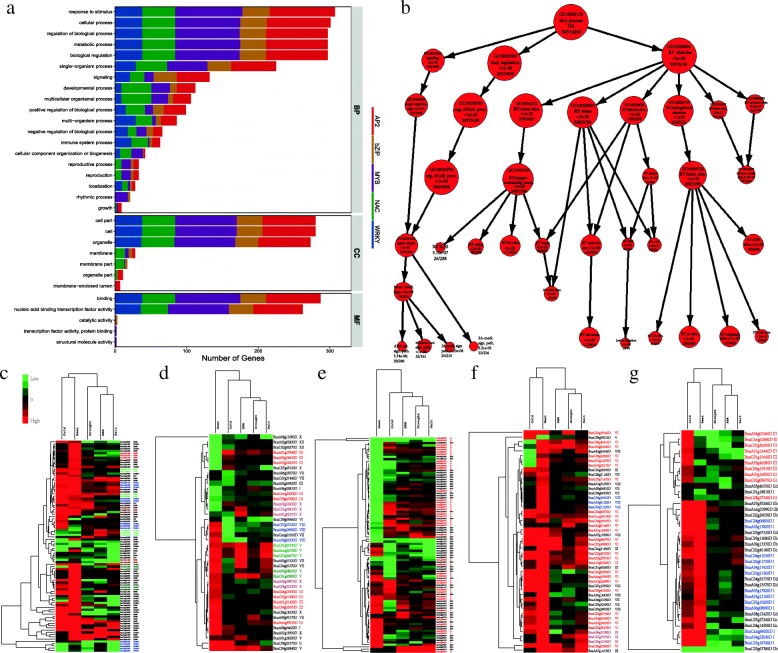
GO enrichment analysis and heat maps of relative expression profiles for the identified 315 DEGs. **a** GO involvement analysis for the identified 315 crucial TFs. BP: biological process; MF: molecular function; CC: cellular component. **b** Part of GO tree of biological processes for the 315 DEGs. Size of nodes is proportional to the numbers of genes. **c**–**g** Cluster analysis and heat maps of relative expression profiles for the identified DEGs. Kendall’s *τ* correlation coefficient is used for arrays to evaluate the similarity among different treatments, Euclidean distance is used to evaluate the closeness between genes, and the average linkage method is used in cluster analysis. DEGs from the same subfamilies are marked with the same colors in each family

GO enrichment analysis performed by WEGO [51] (Fig. 6) reveals that the 315 TFs are enriched in nucleus, cell part, intracellular and organelle, playing functional roles in TF activity, binding and nucleic acid binding TF activity, and they are totally enriched in 213 biological process terms (*P*<0.01), including various biological regulation processes, responding to various stimulus, diverse signaling pathways. Actually, out of the 315 DEGs, 306 DEGs involve the response to stimulus, which further includes many enriched child terms, such as responding to stress (232 DEGs), abiotic stimulus (199 DEGs), endogenous stimulus (229), chemical stimulus (276). Among the 315 DEGs, 53, 41, 111 and 98 DEGs are annotated with responding to cold, heat, salt stress and ABA stimulus respectively, and these terms are all enriched ones.

Except the enriched biological processes, promoter regions of the 315 DEGs are also rich in various cis-elements. About 50% of the critical BnAP2/EREBPs contain the TGA element in their promoters. Among the 42 BnbZIPs, 29 contain ABREs in their promoters and represents an enriched cis-element. Moreover, 21 of the 42 BnbZIPs posses the TCA element in their promoters. The promoters of the 94 BnMYBs are rich in HSE, P box, GARE motif and TGA element. The 48 BnNACs are rich in GARE and TGACG motifs in their promoter regions. Among the 38 BnWRKYs, 30 DEGs contain the MBS and 29 DEGs contain the TC-rich repeats, 22 DEGs contain the HSE and 15 DEGs contain the ABRE elements in their promoters.

Venn diagrams for the 315 DEGs show that although most DEGs are stress/stimulus specific ones, a few DEGs can respond to several different treatments. For each family, DEGs that are unique for heat responsive take up a large fractions. Whereas, the number of genes that are unique under drought or ABA or salt treatments are very low. For example, for the 93 BnAP2/EREBPs, BnaC07g31340D is crucial under four treatments (cold, drought, salt and ABA); while 8 genes are common under three of the treatments, and 28 genes are common ones under two conditions. However, 56 genes are crucial only under one treatment, especially, 35 DEGs are unique under heat stress.

Expression profiles for the identified DEGs are shown in Fig. 6c, where relative expression levels of genes under the five treatments have been used. The expression profiles for the identified genes under drought, salt and ABA treatments are all similar in the five families. A large amount of BnAP2/EREBPs, BnNACs and BnWRKYs are up-regulated under cold or heat treatments. Whereas, a large amount of BnWRKYs under drought, salt and ABA stimulus are down-regulated. It also shows that expressions of genes in each family are hierarchically clustered. For example, 38 BnWRKYs can be classified into three clusters according to their expression profiles. The first cluster includes one subfamily II gene BnaC03g67380D, which is lowly expressed under the five treatments. The second cluster mainly includes subfamilies I and II of BnWRKYs, which are highly expressed under cold and heat stresses, but lowly expressed under other conditions. The third cluster mainly contains subfamily III genes, most of which are up-regulated under cold but down-regulated under other four treatments. Interestingly, we also observe that TFs from the same subfamily tend to be clustered. For example, several clusters of BnAP2/EREBPs mainly consist of DREB or ERF genes, and several clusters of BnWRKYs mainly come from subfamilies I or III.

### Regulatory networks of crucial TFs responsible for stress responsiveness in *B. napus*

**Fig. 7:**
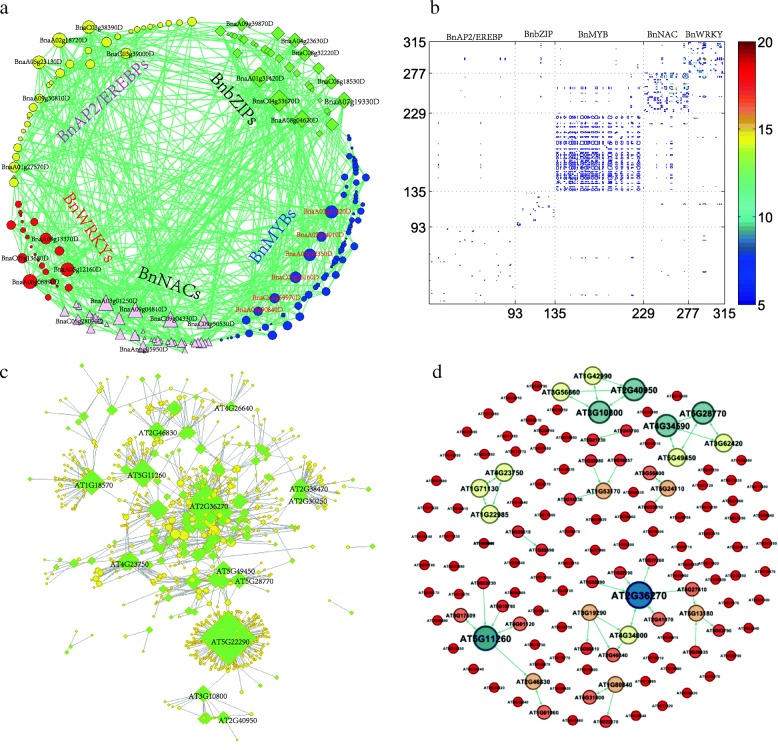
Network analysis on the 315 crucial DEGs in *B. napus*. **a** Gene co-expression network with 1003 directed edges. Two genes with gray correlation coefficient higher than 0.94 are treated with co-expressed. **b** Heat-maps for the process-gene network. Color bars represents the number of shared GO process terms between two DEGs. **c** Gene regulatory network constructed from BioGRID for homologous genes in *A. thaliana*. The network consists of 789 genes and 1253 directed edges. The 315 crucial B. napus genes have 133 homologous *A. thaliana* genes, which are shown in green diamond, while other genes are shown in yellow circles. **d** The regulation relationships among the 133 genes in *A. thaliana*, which contains 61 directed edges

TFs respond to stresses through complex regulatory networks [44, 52]. Gene co-expression network can reflect the similarity of expression dynamics among genes. Based on the RNA-Seq data and gray correlation analysis [53], we detected 542 co-expression gene pairs among the 315 DEGs (Materials and methods, Additional file 1) by taking the threshold of gray correlation coefficient as 0.94, resulting in a co-expression network with average degree 3.4413, as shown in Fig. 7a. On one hand, the network shows that the 315 DEGs have strong co-expression relationships, especially for the 166 DEGs (Including 24 BnAP2/EREBPs, 28 BnbZIPs, 58 BnMYBs, 36 BnNACs and 20 BnWRKYs) that consist of the largest connected component. Among the 315 DEGs, BnaC04g33670D and BnaA06g08890D connect with 23 and 22 other DEGs respectively, which are characterized as two of the most connected genes in the network. BnaC04g33670D belongs to subfamily III of BnbZIPs, which involves the response to ABA. BnaA06g08890D is a WRKY of subfamily I, it involves the response to ethylene, SA, JA. On the other hand, genes in the same family tend to be co-expressed. The relative density of co-expression relationships within families are 1.5 times higher than those among families (Additional file 1). Furthermore, the degrees of the 315 DEGs in the co-expression network have no apparent correlation with their expression levels (Spearman correlation coefficients are all lower than 0.17. Additional file 1). Actually, some highly connected genes have low absolute expression levels under the five treatments. For example, the most connected genes BnaC04g33670D and BnaA06g08890D have very low absolute log2 fold change values under the five conditions. The two genes are both DEGs under heat stress with relative expression level 1.8653 and 2.0570 respectively. Though these densely connected DEGs have low absolute expression levels, they generally co-expressed with many DEGs from the five families. For example, the BnbZIP gene BnaC04g33670D is co-expressed with 4 BnAP2/EREBPs, 5 BnMYBs, 7 BnNACs, 2 BnWRKYs and 5 BnbZIPs. The highly connected BnWRKY gene BnaA06g08890D is co-expressed with 4 BnAP2/EREBPs, 3 BnbZIPs, 3 BnMYBs, 8 BnNACs and 4 BnWRKYs. On the contrary, some genes with high relative expression levels are isolated nodes or loosely connected with other crucial DEGs. For example, the BnAP2/EREBP gene BnaCnng31550D is a DEG with relative expression level 4.2730, 9.3219, 3.3692 respectively under cold, heat and salt conditions. However, it has no co-expression relationships with other DEGs.

To verify whether co-expressed genes are functionally correlated, we construct a process-gene network through GO biological process annotations for the 315 DEGs (Materials and methods, Additional file 1). The process-gene network consists of 315 DEGs and 29466 undirected weighted edges among them. Heat-map for the process-gene network is shown in Fig. 7b, and subnetworks for each family can be seen in Additional file 1. The constructed networks reveal that DEGs from the same family tend to be connected with each other, while DEGs from different families are sparsely connected with each other. Especially, the 94 BnMYBs are densely connected with each others (link density=0.9. Additional file 1) through functioning in some common biological processes. In the process-gene network for the 315 DEGs, the two BnMYBs, BnaC02g16640D and BnaA07g11930D have 283 neighbors. The BnNAC gene BnaA02g05540D connects with another 57 genes, revealing that these genes involve some common popular biological processes as other DEGs.

Finally, based on BioGRID [54], we construct a large-scale gene network for *A. thaliana*, and blast the *A. thaliana* genes with *B. napus*, we find 133 genes in *A. thaliana* that are homologous with members of the 315 crucial genes in *B. napus*. In *A. thaliana*, the 133 genes are connected with other 657 genes, as shown in Fig. 7c. The regulatory network of the 133 genes in *A. thaliana* is shown in Fig. 7d. It reveals that the 133 genes can regulate a large amount of genes, while they can rarely regulate each other. Among the 133 genes, the hub node AT5G22290 can regulate 29 genes and be regulated by 209 genes. However, none of these 238 neighbors are members of the 133 genes. Gene AT5G22290 is homologous with *B. napus* gene BnaA02g05540D, which is a BnNACs and involves in plant-type hypersensitive response, negative regulation of flower development, response to endoplasmic reticulum stress and so on. In RNA-Seq data, BnaA02g05540D is up-regulated under heat stress, while it is down-regulated under cold stress. AT2g36270 is highly connected in Fig. 7c and is reported to be an ABA responsive element [54]. AT2g36270 is homologous with BnaA05g08020D, which is an up-regulated BnbZIP gene under heat stress. In *A. thaliana*, AT2g36270 can directly regulate the expression of 37 genes and be regulated by 44 genes. Whereas, in the gene network of the 133 genes as shown in Fig. 7d, AT2g36270 can regulate or be regulated by another 6 of the 315 crucial TFs, and represents one of the most connected genes in Fig. 7d. Moreover, subnetwork of the 133 genes consists of 6 triangle motifs, and genes that consist of the motif come from the same TF family. For example, the four AtbZIP genes AT5G49450, AT5G28770, AT4G34590 and AT3G62420 form two mutually regulated triangle motifs. AT3G56660, AT3G10800, AT2G40950 and AT1G42990 are also four AtbZIPs, which also consist of two mutually regulated triangle motifs.

The 133 TFs in *A. thaliana* can regulate or be regulated by 657 other genes, and 465 of them are homologous with 600 treatment responsive genes of *B. napus*. For simplicity, if one homologous gene in *B. napus* is a DEG under a treatment, then we call its counterpart in *A. thaliana* as a DEG. Statistical analysis (Additional file 1) shows that the AtAP2/EREBPs, AtbZIPs, AtMYBs, AtNACs and AtWRKYs can regulate 22, 38, 42, 24, and 11 down-stream DEGs respectively. About 45%-81% down-stream genes of the five TF families are heat inducible. About 50% down-stream genes of AtbZIPs are cold responsive. Down-stream genes of AtAP2/EREBPs are short of drought responsive ones. However, 2 of the 11 down-stream genes of AtWRKY are drought responsive ones, and 6 of the 42 down-stream genes of AtMYBs are drought responsive ones. Three down-stream genes of AtWRKY and 9 down-stream genes of AtbZIPs are salt inducible. The down-stream genes regulated by AtAP2/EREBPs have the highest fractions of ABA responsive ones (40.9%). Moreover, among the TF regulated genes, the AtAP2/EREBP regulated gene AT2G44040 has been reported to involve in response to abiotic stresses [55], and we found its homologous genes in *B. napus* are ABA, heat and salt responsiveness. It is also reported that the AtbZIP regulated gene AT3G11410 can negatively regulate ABA signalling and up-regulated by drought and ABA [28], and we found its homologous genes in *B. napus* are ABA, cold, heat and salt responsiveness. Therefore, we speculate that the identified TFs can actually trigger the differential expression of their down-stream genes.

## Discussion

### Genome-wide screening reveals novel members in the five TF families

We genome-widely identified five families of TFs for *B. napus*, *B. rapa, B. oleracea* and *A. thaliana* respectively. Compared with the existing databases or references, our results include some novel members and they are more accurate.

Compared with 87 [56], 132 [30] and 515 BnAP2/EREBPs [57] in *B. napus* that the references have reported, we identified 518 BnAP2/EREBPs, which include some novel members. Existing works also identified 136 BrbZIPs in *B. rapa* [31], 119 BobZIPs in *B. oleracea* [24] and 247 BnbZIPs in *B. napus* [8]. While the numbers of bZIPs for the three species identified by us are 126,127 and 252. Compared with the existing results, we exclude some improperly identified TFs while include some unreported ones. Hajiebrahimi et al. [34] identified 249 R2R3 BnMYBs in *B. napus*. However, we found that 424 of the identified 721 BnMYBs belong to the R2R3 subfamily and include 235 of the 249 TFs reported by Hajiebrahimi et al. as members. Whereas, the left 14 TFs (Additional file 1) are not identified as BnMYBs by us, but they are all collected as BnMYBs in PlantTFDB. Moreover, we find 50 BnMYBs, which have not been reported by existing databases and references. Compared with the identified 60 BnNACs from *B. napus* by Wang et al.[35], we identified 398 BnNACs, whose number is six times more than the existing references. WRKYs in various species have been extensively investigated [21, 27, 40, 41, 58]. Researchers have found that some WRKY amino acid sequences have been replaced by WRRY, WSKY, WKRY, WVKY, WKKY or WKNY in WRKY proteins [17]. We found that the WRKY domain of a group IIc BnWRKYs BnaCnng77260D has been replaced by WKNY, while two group IIc BnWRKYs BnaCnng28950D and BnaCnng39890D have been replaced by WKKY. The number of our initially identified WRKYs via sequence comparisons of the four species are 91, 145, 148 and 285 respectively. However, 7 transcripts (Additional file 1) in *A. thaliana* don’t have either WRKY domains or the zinc finger-like motifs with multiple sequences alignment by ClustalW. Therefore, we finally identified 84 AtWRKY transcripts in *A. thaliana*. The two genes Bra008456 and Bra036563 in *B. rapa* don’t have WRKY domain. Moreover, although the two genes Bra034482 and Bra037637 have WRKY domains, but don’t have zinc finger motifs. Thus, we finally identified 141 BrWRKYs in *B. rapa*. Similarly, six (Additional file 1) of the 148 *B. oleracea* transcripts don’t have either WRKY domains or zinc finger motifs. Thus, only 142 BoWRKYs are identified in *B. oleracea*. Further domain analysis reveals that the *B. napus* gene BnaA02g19590D has inconsistent WRKY domain; the six *B. napus* genes BnaC04g19430D, BnaC05g14560D, BnaC02g09600D, BnaC07g25890D, BnaA02g02500D, BnaA01g13720D all lack the zinc finger motifs. Therefore, we finally confirmed 278 BnWRKYs in *B. napus*. Compared with PlantTFDB [27] and the 289 BnWRKYs reported by Wu et al. [40], the finally identified BnWRKYs are relatively fewer. Compared Ref. [40] with our results, 271 genes are common (Additional file 1). But 18 genes are exclusively reported by Wu et al, which actually don’t have either WRKY domain or zinc finger motifs. For example, BnaA01g13720D lacks the zinc finger motif. Some novel members, such as BnaA06g13050D and BnaA09g21040D, are confirmed to be BnWRKYs. Our new results indicate that the reported TF families in existing databases or references may contain inaccurate information, or some special genes/transcripts do not follow the usual structures.

### Statistical analysis reveals distinctive structural and functional features of TF families

The identified TFs in *B. napus* are almost doubled as those in *B. rapa* and *B. oleracea*, it indicates that the genome of *B. napus* is far more complex than its diploid ancestors. Moreover, TFs in each family are widely distributed on the 19 chromosomes of *B. napus*. Some chromosomes are enriched with certain families of TFs, and such phenomena can be also observed in its ancestral species *B. rapa* and *B. oleracea*. Non-parameter tests show that the BnAP2/EREBPs and BnMYBs have suffered from duplication or divergence effect during evolution. Furthermore, synteny analysis shows that TFs in the three *Brassica* species have a very strong co-linearity, although chromosomal rearrangements and gene duplication events have occurred between *B. napus* and its ancestral genomes after divergence from their common ancestor. The three aspects of observations indicate the TF families in *B. napus* are mainly formed as a result of whole-genome duplication [34], but suffer from chromosomal rearrangements and with some distinctive features.

Each of the identified TF families can be divided into several subfamilies according to the constructed phylogenetic tree. It is reported that genes with similar structures in *A. thaliana* usually have similar functions [18]. Thus, we speculate that genes in the same cluster for *B. napus* may also have similar functions. Interestingly, it is reported that AtWRKYs can be divided into three subfamilies in *A. thaliana*: I, II and III. The subfamily II WRKYs can be further split into five sub-groups IIa-IIe [17]. However, the identified 278 BnWRKYs lack group IIa members. In fact, Yang et al. [39] reported two group IIa BnWRKYs: BnWRKY18 and BnWRKY40, but the two BnWRKYs are clustered into the same clade as group IIb genes in the phylogenetic tree. He et al. [41] reported 11 BnWRKYs of group IIa. However, there are no classifications of these genes in PlantTFDB, and the they are all classified into group IIb in our work. Moreover, for *A. thaliana*, Wu et al. [50] reported that there are three group IIa AtWRKYs, including AtWRKY18, AtWRKY40 and AtWRKY60. However, the three group IIa AtWRKYs have close distances with group IIb AtWRKYs. Thus, we speculate that BnWRKYs in *B. napus* have distinctive structural features in comparison with other species.

Promoters of genes in different TF families prefer different cis-elements. More than 60% BnNACs and BnWRKYs contain the TCA-element, TGACG-element, MBS and TC-rich repeats in their promoters, illustrating that these TFs probably involve the SA signaling pathway, Methyl Jasmonate (MeJA) signaling pathway, drought response, defense and stress response respectively. In addition, many BnWRKYs contain the LTR, ABRE and HSE elements in their promoters, which suggests that these TFs may involve in cold responsiveness, ABA signaling pathways and heat responsiveness respectively. The preferences of different families with varied cis-elements in their promoter regions indicate different TF families may prefer distinct functions.

Synteny analysis reveals that although more than 80% genes are multi-copied ones, each TF family in *B. napus* encompasses 7.6% novel genes and 9.4% conserved genes in compared to its diploid ancestors. The novel genes and conserved genes prefer the C sub-genome. Chromosomes chrC03, chrC04, chrA06 and chrA09 are rich in novel genes. ChrC03, chrC01, chrA03 and chrA08 are rich in conserved genes. These results indicate that the associated sub-genome and chromosomes may play much more important roles in the formation of the *B. napus* genome than the others. Novel genes, conserved genes and multi-copy genes in some TF families also show subfamilies preferences. Gene loss is also observed by synteny analysis between *B. rapa, B. oleracea* and *B. napus*. More gene loss events occurred in the C sub-genome, which further indicates that the C sub-genome may suffer more natural selection pressure. Gene loss prefers the AP2/EREBPs and MYBs, indicating that the two TF families are more vulnerable during evolution, which also illustrates that BnAP2/EREBPs and BnMYBs may suffer more selection pressure during evolution.

### Transcriptional expression profiles of TFs under treatments reveal their functional roles

RNA-Seq data reveals that more than 80% of the identified TFs in *B. napus* are responsive to stresses/stimuli, where 474 members of all the 2167 TFs are DEGs under at least one treatment, including 140 BnAP2/EREBPs, 75 BnbZIPs, 141 BnMYBs, 68 BnNACs and 50 BnWRKYs. Among the 474 DEGs, 303 genes can only differentially respond to one treatment, the left 171 genes can differentially respond to at least two treatments. This indicates that most members are stress/stimulus specific ones, while about 30%-45% genes play multiple roles.

The number of common DEGs responding to cold and heat stresses are 25, 5, 21,14 and 10 in the five families respectively. Which may because cold and heat stresses are both dominated by temperature, these common DEGs have evolved into some common functions to respond to temperature changes. Furthermore, most of the drought, salt and ABA responsive DEGs can simultaneously respond to other treatments, which may indicate drought, salt and ABA are concomitant stress/stimulus factors, which may be affected by other common factors. For instance, drought may be caused by heat and salt stresses.

Under cold or heat stresses, most DEGs in BnAP2/EREBPs, BnNACs and BnWRKYs are up-regulated, while DEGs in BnMYBs tend to be down-regulated, and most DEGs in BnbZIPs are down-regulated under cold stress but up-regulated under heat stress. DEGs are relatively fewer under drought, salt and ABA treatments. Six of the 7 drought responsive DEGs in BnNACs are up-regulated, and the 4 drought responsive DEGs in BnWRKYs are all down-regulated. Under salt stress, most of responsive DEGs in BnAP2/EREBPs, BnbZIPs and BnMYBs are down-regulated, while the 9 responsive BnNACs are all up-regulated. Under ABA stimulus, the 12 DEGs in BnNACs are all up-regulated and the 7 responsive DEGs in BnWRKYs are all down-regulated. The varied regulation profiles indicate that TFs in different families play distinct regulation roles.

### Exploring regulatory networks of crucial TFs involving different abiotic stresses

Based on GO biological process annotations for DEGs, we curated 315 crucial DEGs. Compared with the 315 DEGs with the 132 BnAP2/ERFs identified by Du et al. [30], the authors confirmed 13 cold-induced BnAP2/ERF TFs by qRT-PCR. Out of the 13 genes, 7 genes are members identified by us, including BnaA09g30360D, BnaA04g29310D, BnaA08g13860D, BnaA10g25000D, BnaC09g49920D, BnaC09g27360D and BnaA08g19490D. In fact, BnaA09g30360D is down-regulated under salt and ABA stimulus; BnaA04g2-9310D is up-regulated under cold and heat, but down-regulated under drought. The three DEGs, BnaA08g13860D, BnaA10g25000D and BnaC09g49920D, are all up-regulated under heat stress. BnaC09g27360D and BnaA08g19490D are both down-regulated under salt stress. For the three salt responsive BnMYBs reported by Hajiebrahimi et al. [34], we found that BnaMYB21(BnaA02g33410D) is a member of the 315 crucial DEGs, but it is ABA responsiveness. Compared with the 79 candidate genes with water stress tolerance in *B. napus* reported by Zhang et al. [59], BnaA05g08020D is a member of the 42 BnbZIPs identified by us. BnaA05g08020D is homologous with *A. thaliana* genes ABI5. ABI5 participates in ABA-regulated gene expression during seed development and subsequent vegetative stage by acting as the major mediator of ABA repression of growth. In RNA-Seq data, BnaA05g08020D is up-regulated under heat, which involves the response to water deprivation, salt stress, ABA, gibberellin, and chitin mediated signaling pathway. Actually, the 315 DEGs are all annotated with functional roles in stimulus responsiveness. Among the 315 DEGs, we also observe that several genes are DEGs under more than one treatments. Interestingly, expression profile of each family reveals that TFs in the same subfamily tend to be clustered, which indicates that structurally similarly TFs tend to have the similar expression patterns under stresses.

The identified 315 crucial TFs involve some novel members (Additional file 1). For example, 403 BnAP2/EREBPs have been collected by PlantTFDB but not investigated by the latest reference [30], among which, 51 TFs have been identified as crucial TFs by us. Two BnMYBs, BnaA03g42350D and BnaA08g30200D, are novel TFs identified by us, which have not been identified in previous works or databases. However, we find BnaA03g42350D is a crucial heat responsive gene, which involves ethylene-activated signaling pathway and various regulation processes. It contains ABRE, HSE, LTR, MBS and TC rich repeats, indicating that it may involve the response to ABA, heat, cold, drought, defense and stress responsive. BnaA08g30200D is a crucial cold responsive gene, which involves cold acclimation, response to auxin, response to hydrogen peroxide and so on. It contains five cis-elements in its promoters, including ABRE, GARE motif, P-box, HSE and MBS, which indicates such gene may involve the response to ABA, GA, heat and drought stresses. BnaA08g30200D is homologous with the AtMYB gene AT4G16420 in *A. thaliana*. AT4G16420 is the composition of SAGA complex and is reported to control gene expression in the response to abiotic stresses [60]. AT4G16420 can regulate five genes. The homologous genes of the regulated genes in *B. napus* can respond to heat, salt and ABA. Therefore, we declare that the identified crucial TFs includes some novel members.

Stress/stimulus responsiveness in plant relies on regulatory networks [57]. The constructed gene co-expression network reveals that the 315 DEGs are highly co-expressed, and genes in the same family tend to have similar expression dynamics. Moreover, we find that these highly expressed DEGs tend to directly regulate the target genes to perform biological functions, while lowly expressed genes tend to be co-expressed with some other TFs, and act as mediators. Furthermore, in the constructed process-gene network, we find that the DEGs also tend to be connected with each other in the same family, while DEGs from different families are sparsely connected with each other, which indicates that genes from the same TF family tend to be functionally clustered via involving similar biological processes. It also indicates that structurally similar DEGs tend to have similar expression profiles and biological functions. The homologous gene network in *A. thaliana* of the 315 DEGs reveals that the identified TFs can regulate a large amount of genes. However, TFs can rarely regulate each others. Moreover, we observe that there are some triangle motifs among the TFs in the gene network, such motifs are building blocks of biological systems [61–63]. The frequently appeared triangle motifs among homologous genes in *A. thaliana* indicate the gene network for *B. napus* may also consist of building block motifs. Further analysis reveals that structurally similar genes contribute to those motifs.

**Fig. 8:**
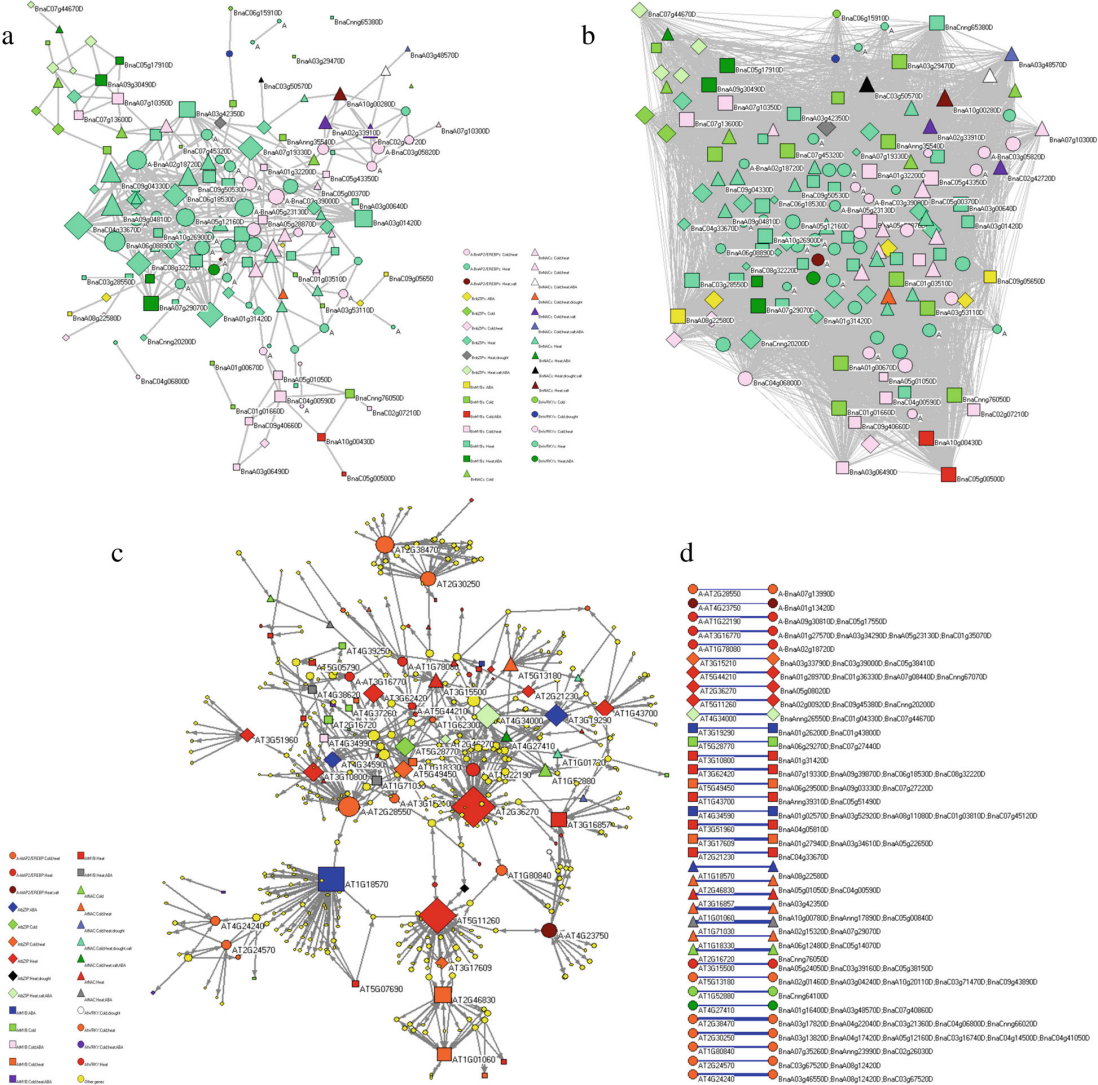
Subnetworks for common DEGs in co-expression, process-gene and homologous networks. **a** Co-expression subnetwork of the 153 DEGs that have common edges in the co-expression and process-gene networks, which has 350 undirected edges. **b** Process-gene subnetwork of the 153 DEGs with 7606 undirected edges. **c** Homologous gene network in *A. thaliana* that consists of 466 nodes and 720 directed edges. 86 of the 466 genes are homologous with the 153 common DEGs in (**a**) and (**b**). **d** Homologous relationships among the top-36 highly connected genes in (**c**) between *A. thaliana* and *B. napus*. Different shapes of nodes denote genes from different families; Different colors corresponding to different kinds of stress responsiveness. To distinguish between WRKYs and AP2/EREBPs, nodes with prefix “A” correspond to AP2/EREBPs

The three types of networks describe different aspects of relationships among the crucial TFs. The co-expression network describes the likelihood of two DEGs with similar dynamical behaviors under stresses. The process-gene network reveals the tendency of DEGs involving common biological processes. Thus, it can reflect the functional relationships among DEGs. The homologous network describes the regulatory relationships of the homologous genes in *A. thaliana*, and it may indicate similar regulation relationships of homologous genes in *B. napus*. The gene co-expression network and process-gene network commonly contain 309 undirected edges among 153 DEGs (Additional files 1 and 6). We withdraw the subnetworks for the 153 DEGs in the two networks, which are shown in Fig. 8a and b. The 153 DEGs in *B. napus* correspond to 86 homologous genes in *A. thaliana*, the regulation network of the 86 genes in *A. thaliana* is shown in Fig. 8c. This subnetwork may reflect the real-world stress responsive regulatory network in *B. napus*. A considerable DEGs in the three subnetworks are heat responsive. In fact, 127 out of the 153 DEGs in the gene co-expression and process-gene subnetworks are heat responsive, including 54 DEGs which are also critical for other stresses. For example, the BnNAC gene BnaA03g48570D is a critical DEGs under four treatments, including cold, heat, salt and ABA. Only four DEGs are responsive to drought, including BnaC03g50570D, BnaA09g00170D, BnaC02g00990D and BnaA07g16850D. However, the four drought responsive genes are also responsible for cold, heat or salt stresses. Similarly, 82 of the 86 genes in *A. thaliana* are homologous to cold or heat responsive DEGs in *B. napus*. A few homologous genes in *A. thaliana* can regulate or be regulated by a large amount of other genes. Figure 8 further reveals that stimulus responsive genes tend to exhibit similar expression dynamics under stimulus and to involve similar biological processes, regulate or be regulated by a large amount of other genes, resulting in a complex clustered regulatory network that are responsible for targeted stress responsiveness.

### qRT-PCR validations on several responsive genes

**Fig. 9:**
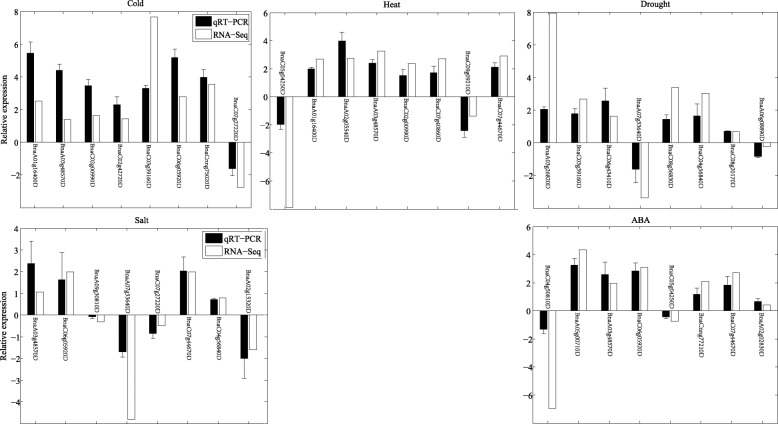
qRT-PCR validations on a few of the 315 DEGs under the five treatments. Eight DEGs under each treatment are selected for further qRT-PCR validation. RNA-Seq: the relative expression level in the RNA-Seq data; qRT-PCR: the relative expression level in the qRT-PCR experiment. If the two data have the same trend, then it indicates the reproducibility of the results

qRT-PCR validations on several members of the 315 genes are shown in Fig. 9, which confirm that the selected genes are stress inducible and some of them are DEGs under treatments. Under each condition, we consider 8 genes, the regulation patterns of these genes from the qRT-PCR experiments are all coincide with those from the RNA-Seq, though there are some differences on relative expression levels. For example, BnaA01g16400D is a member of the 315 DEGs under cold and heat stresses, which is both up-regulated under the two conditions with relative expression levels 2.5057 and 2.6966 in RNA-Seq, and with relative expression levels 5.4400 and 1.9882 in the qRT-PCR experiments. BnaA02g05540D is confirmed to be inducible by heat stress and with relative expression level 3.9726 under such stress. Moreover, we also consider several genes with relatively low expression levels (Not DEGs) under certain stresses. For example, BnaA09g30810D, BnaC07g27220D and BnaC04g56840D are all salt responsiveness in RNA-Seq, but they are not DEGs. We confirm these genes are not DEGs from the qRT-PCR data.

## Conclusions

Five families of totally 2167 TFs are genome-widely identified and characterized in *B. napus*, including 518 BnAP2/EREBPs, 252 BnbZIPs, 721 BnMYBs, 398 BnNACs and 278 BnWRKYs. Compared with the existing references and databases, the identified TFs include some novel members. Especially, we identified 50 novel BnMYBs and excluded some incorrectly reported BnWRKYs. Two novel BnMYBs, BnaA03g42350D and BnaA08g30200D, are further confirmed to be critical for stress responsiveness. We perform detailed structure analysis, classifications, phylogenetic analysis, cis-elements analysis and synteny analysis on the identified genes, and clarify their featured structures and evolutionary relationships between *B. napus* and its diploid ancestors and *A. thaliana*. Our results reveal that the TFs in *B. napus* were mainly formed by whole-genome duplication, although chromosome rearrangements had occurred and 85 TFs were lost. About 7.6% and 9.4% TFs of the five families in *B. napus* were novel genes and conserved genes, which both showed preference on the C sub-genome.

Responsiveness of the TF families under cold, heat, drought, salt and ABA is explored, 80% TFs can respond to at least one of the stimulus. On the basis of RNA-seq data under the five treatments, and further considering biological process annotations, we manually identified 315 crucial DEGs, including 93 BnAP2/EREBPs, 42 BnbZIPs, 94 BnMYBs, 48 BnNACs and 38 BnWRKYs. Promoters of the 315 crucial DEGs contain various cis-elements that play important roles in stress response, and GO enrichment analysis performed on the selected DEGs reveals that they are enriched in the response to various stimuli. We also found that the orthologous genes in *A. thaliana* for some of the crucial genes have been reported to be critical stress responsiveness, which further verified the obtained results. Expression profiles of the 315 DEGs reveals that different DEGs in each family are clustered according to their subfamilies, indicating structural similar DEGs tend to exhibit similar expression vigor. This declaration is further supported by gene co-expression network and process-gene network. Further network analysis on the 315 crucial DEGs reveals the complex regulatory networks that respond to various stimuli. Detailed literature review illustrates that some of the 315 DEGs can actually trigger the stress responsive of down-stream genes.

This investigations characterize the features and functions of TF families in *B. napus*, which have potential implications for breeding of new varieties with improved stress-tolerance [64, 65].

## Methods

### Database search, sequence retrieval and TF identification

We genome-widely identify the AP2/EREBPs, bZIPs, MYBs, NACs and WRKYs in *A. thaliana*, *B. rapa*, *B. oleracea* and *B. napus* by using the publicly available genomics and putative full-length protein sequences. We downloaded the genome sequences and protein sequences for the four species from TAIR [28], BRAD [29], ensemblgenomes [66] and Genoscope [3] respectively. For example, we downloaded 101040 genes from the associated online database GenoScope for *B. napus*. We retrieved 656177 ESTs at the RNA level from the NCBI database, and obtained the related amino acid sequence for each protein in *B. napus*. Then we blast the downloaded protein sequences with recorded sequences in Pfam [67] to verify whether they have AP2 domain (PF00847), bZIP_1_ (PF00170) and bZIP_2_ domains (PF07716), MYB domain (PF00249), NAM domain (PF02365), and WRKY domain (PF03106). Proteins with E-value < 1*e*−5 are extracted to make further domain prediction by using SMART [68]. Transcripts or genes that pass the Pfam comparison and SMART validations are deemed as putative family members. MEME [69] motif predictions are further performed for the identified sequences to verify their consistence with domain prediction results.

### Plant materials and RNA extraction

One *B. napus* variety (Zhongshuang 11) and five treatments are taken into consideration. The seedlings with 7 days on 1/2 Hoagland liquid culture were transferred to 1/2 Hoagland nutrient solution, and then seedlings were separately treated with five stress/stimulus conditions, including 15% PEG-6000, 150 mM NaCl, cold (4^∘^C), heat (40^∘^C) and ABA (30 mmol/L). The seedlings without any treatments were taken as controls. To induce responsive gene expression, seedlings were sampled at 12h after treatments for RNA extraction. Each experiment was repeated three times. Total RNA was extracted with TRIzol according to the manufacturer’s instructions (Invitrogen, USA). RNA quality was determined using a 2100 Bioanalyzer (Agilent) and quantified using the ND-2000 (NanoDrop Technologies). A high-quality RNA sample (*RIN*≥6.5,28*S*:18*S*≥1.5) was used to construct the sequencing library.

### mRNA library construction and sequencing

RNA-seq transcriptome libraries were prepared following the instructions for the TruSeq RNA Sample Preparation Kit from Illumina (San Diego, CA), using 5*mg* of total RNA. Briefly, mRNA was isolated by polyA selection using oligo (dT) beads and fragmented using fragmentation buffer. Next, cDNA synthesis, end repair, A-base addition, and ligation of the Illumina-indexed adaptors were performed according to the manufacturer protocol. After quantification by Agilent 2100 Bioanaylzer, paired-end libraries were sequenced on the Illumina HiSeq 4000 platform (2×151*bp* read length).

Our primary sequencing data has been deposited in the NCBI Sequence Read Archive (SRA, http://www.ncbi.nlm.nih.gov/Traces/sra) under accession number SRP109808. These data, denoted as raw reads, were subjected to quality control to determine whether a re-sequencing step was needed. Specifically, the raw reads were cleaned by removal of reads containing primer/adapter sequences, low-quality reads (i.e., a percentage of low quality bases greater than 50% in a read, with low quality bases defined as bases with a sequencing quality of no more than 10) and reads with more than 10% unknown bases.

### Quantifying transcript abundance and differential gene expression

After filtering, the remaining reads were called clean reads, which were aligned to the reference sequences with Bowtie2 [70] (version: v2.2.6, parameters:-q–phred33–sensitive–dpad0–gbar99999999–mp1,1–np1–score-minL, 0, -0.1-I1-X1000–no-mixed–no-discordant -p1-k 200), and then calculate gene expression level with RSEM [71] (version: v1.2.12, parameters: default). To identify the DEGs in two different samples, the expression level for each transcript was calculated using the fragments per kilobase of exon per million mapped reads (FPKM) method. NOIseq [46] was used for the differential expression analysis. DEGs of the two samples were selected using the following criteria: 1) a logarithmic fold change greater than 1, and 2) a Probability of no less than 0.6.

### Multiple sequences alignment and phylogenetic analysis

Multiple sequences alignment was performed using ClustalX [72] and DNAMAN 7.0 (http://www.lynnon.com). The parameters for alignment were chosen as follows: gap opening penalty, 10.00; gap extension penalty, 0.20; protein weight matrix, gonnet; delay divergent sequences, 30; DNA transition weight, 0.5; use negative matrix, off; DNA weight matrix, IUB. Phylogenetic trees were constructed through the NJ method using program MEGA7 [73]. The parameters of the constructed trees were: phylogeny test and options: bootstrap (1000 replicates), gaps/missing data: complete deletion, model: amino: Poisson correction, substitutions to include: all, pattern among lineages: same (homogeneous), and rates among sites: uniform rates.

### Identification of homologous genes among *B. napus, B. rapa, B. oleracea,* and *A. thaliana*

Homologous genes were predicted by means of synteny-based methods in the CoGe database [74]. Orthologous genes of *B. napus* were predicted in the target genomes of *A. thaliana, B. rapa*, and *B. oleracea* through the identification of a conserved syntenic region via SynFind with the following parameters: last comparison algorithm, minimum number of four genes, and unlimited syntenic depth. Putative paralogous genes in *B. napus* were identified via SynMap by the following parameters: last algorithm, 20 genes for the maximum distance between two genes, and five genes for the minimum number of aligned pairs. The algorithm for syntenic depth was defined as quota-align with a coverage depth of 1:1 for *B. napus* versus *B. rapa* and *B. oleracea*. The coverage depth of *B. napus* versus *A. thaliana* was determined at 6:1. Orthologous sets were visualized via McScanx (http://chibba.pgml.uga.edu/mcscan2/).

### Identification of putative cis-elements in *B. napus*

Sequences of 1500-bp upstream of the transcriptional start site of genes were submitted to the PlantCARE website (http://bioinformatics.psb.ugent.be/webtools/plantcare/html/) to identify putative cis-elements.

### Enrichment analysis

GO encompasses cellular components, molecular functions and biological processes. We mainly consider the biological process, and enrichment analysis was performed with WEGO [51]. To control false discovery rate (*FDR*), the rigorous Bonferroni correction method [75] was used. GO terms fulfilling *FDR*≤0.05 were defined as significantly enriched ones.

### Gray correlation coefficient and co-expression network construction

The traditional correlation coefficient, such as the Pearson correlation coefficient (PCC), the Spearman and the Kendall *τ* correlation coefficient all need large sample data [43]. Moreover, the PCC is sensitive to the distribution of data, which only shows good performance in linear cases. The Spearman correlation coefficient can be applied to nonlinear data.

The gray correlation coefficient [53] (GCC) is appropriate for small sample data. Assume *x*_0_=(*x*_0_(1),*x*_0_(2),…,*x*_0_(*n*))^′^ is a reference sequence, *x*_*i*_=(*x*_*i*_(1),*x*_*i*_(2),…,*x*_*i*_(*n*))^′^(*i*=1,2,…,*m*) are observations of the *i*^′^*th* gene. Define the GCC between *x*_0_ and *x*_*i*_ at point *k* as

1 $$ \xi_{i}(k)\,=\,\frac{min_{i} min_{k} |x_{0}(k)\!\,-\,x_{i}(k)|\,+\,\!\rho max_{i} max_{k} |x_{0}(k)\!\,-\,x_{i}(k)|}{|x_{0}(k)\,-\,x_{i}(k)|\,+\,\rho max_{i} max_{k} |x_{0}(k)\,-\,x_{i}(k)|}.  $$

Here, *ρ* is resolution ratio, which is usually taken as 0.5. The overall GCC between *x*_0_ and *x*_*i*_ is defined as

2 $$ r_{0i}=\frac{1}{n}\sum\limits_{k=1}^{n}\xi_{i}(k).  $$

Obviously, the GCC are always non-negative. In this paper, the expression levels of DEGs under the five treatments are orderly taken as reference sequences, and thus we obtain a gray correlation matrix that can reflect the pairwise co-expression relationships among DEGs. If *r*_*ij*_>=0.94, we set an edge with weight *w*_*ij*_=*max*{*r*_*ij*_,*r*_*ji*_} between genes *i* and *j*. The threshold value 0.94 guarantees the sparsity of the network.

### Network construction based on GO annotations

To reveal the internal relationships between the selected DEGs and their biological processes, we constructed networks and performed network analysis [52, 76]. First, we constructed a bipartite graph that mapped the DEGs to their biological processes. In detail, if a DEG involves some biological processes, then we connected the DEG with each of its biological process terms. Thus, we were able to derive a bipartite graph containing two kinds of nodes: DEGs and biological process terms. If two DEGs simultaneously involve the same biological processes, then we connected the two DEGs with an edge and thus derived a weighted process-gene network with nodes representing DEGs, where the weight of an edge denotes the number of biological processes that are common to the two DEGs.

### Expression pattern analysis

To analyze gene expression patterns, we used the Illumina RNA-seq data, containing five conditions. The FPKM log2-transformed values were used to generate heat maps by using Cluster 3.0 (http://bonsai.hgc.jp/~mdehoon/software/cluster/software.htm) and Java TreeView (http://jtreeview.sourceforge.net/).

### qRT-PCR

qRT-PCR experiment was conducted using GoTaq qPCR Master Mix (Promega Biotechnology) on an ABI 7500 FAST real-time PCR machine (Applied Biosystems, USA) with a final volume of 10 *μL* per reaction. Each reaction mixture contained 5 *μL* GoTaq Mix (GoTaq qPCR Master Mix 2X), 2.0 *μL* cDNA template, 0.5 *μL* each primer (1.0 *μM*), and 2 *μL* nuclease-free water. Each reaction was performed in triplicate. The cycling parameters were 95 ^∘^C for 5min, followed by 40 cycles at 95 ^∘^C for 15s and 60 ^∘^C for 30s. Melt-curve analyses were performed using a program of 95 ^∘^C for 15s and then a constant increase temperature from 60 ^∘^C to 95 ^∘^C. The *B. napus* Actin1 gene was used as the internal reference gene. The relative gene expression levels were determined by the 2^−*ΔCt*^ method [77].

## Additional files

Additional file 1 Supplementary materials that supporting the main text. (PDF 61,360 kb)

Additional file 2 The identified five families of TFs in the four species. (PDF 216 kb)

Additional file 3 Final statistics for synteny analsyis. (PDF 85 kb)

Additional file 4 Responsive genes of the five TF families. (PDF 531 kb)

Additional file 5 The finally identified 315 crucial DEGs. (PDF 128 kb)

Additional file 6 Detailed information for the 153 common DEGs. (PDF 61 kb)

## Abbreviations

*A. thaliana*: *Arabidopsis thaliana*
ABA: Abscisic acid
*B. napus*: *Brassica napus*
*B. oleracea*: *Brassica oleracea*
*B. rapa*: *Brassica rapa*
CT: C-terminal
DEG: Differentially expressed genes
ESTs: Expressed sequence tags
FDR: False discovery rate
FPKM: Fragments per kilobase of exon model per million mapped reads
GA: Gibberellin
GO: Gene ontology
JA: Jasmonic acid
MeJA: Methyl Jasmonate
NT: N-terminal
qRT-PCR: quantitative real time polymerase chain reaction
RNA-Seq: RNA-sequencing
SA: Salicylic acid
TF: Transcription factor
