# Supplementary material for: Exploring transcription factors reveals crucial members and regulatory networks involved in different abiotic stresses in Brassica napus L
Source: BMC Plant Biol. 2018 Sep 19;18:202. doi: 10.1186/s12870-018-1417-z (PMC6146658; doi:10.1186/s12870-018-1417-z)
Supplement: Supplementary file 1 — Supplementary materials that supporting the main text. (PDF 61,360 kb) [file 12870_2018_1417_MOESM1_ESM.pdf]

Supplementary matrials for “Exploring transcription factors reveals crucial members and regulatory networks involved in different abiotic stresses in *Brassica napus L.*”

Pei Wang[1], Cuiling Yang[1], Hao Chen[1], Longhai Luo[2], Qiuli Leng[1], Shicong Li[1], Zujing Han[2], Xinchun Li[2], Chunpeng Song[1], Xiao Zhang[1], and Daojie Wang[1*]

[1]Key Laboratory of Plant Stress Biology; School of Mathematics and Statistics; State Key Laboratory of Cotton Biology; College of Life Sciences; Institute of Applied Mathematics; Laboratory of Data Analysis Technology; Henan University, Kaifeng, Henan, 475004, China
[2]Beijing igeneCode Biotech Co.,Ltd, Beijing, 100096, China
[*]Correspondence and requests for materials should be addressed to D. Wang (wangdj@henu.edu.cn)

ABSTRACT

This file is the supplementary materials for “Exploring transcription factors reveals crucial members and regulatory networks involved in different abiotic stresses in *Brassica napus L.*”.

Genome-wide identification of five TF families

Genome-wide analysis was carried out to identify the AP2/EREBPs, bZIPs, MYBs, NACs and WRKYs in the *A. thaliana*, *B. rapa*, *B. oleracea* and *B. napus* genomes by using the publicly available genomics and putative full-length protein sequences. Procedures are as follows. Firstly, we download the genome sequences and protein sequences for the four species from TAIR[1], BRAD[2], ensemblgenomes[3] and Genoscope[4] respectively. Then we blast the downloaded protein sequences with sequences in Pfam[5] to verify whether they have AP2 domain (PF00847), $bZIP_1$ domain (PF00170) and $bZIP_2$ domain (PF07716), MYB domain (PF00249), NAM domain (PF02365), as well as WRKY domain (PF03106). Genes or proteins with E-value less than $1e-5$ are extracted to make further domain prediction by using SMART[6]. Genes or transcripts that pass the Pfam comparison and SMART validations are deemed as putative family members. MEME[7] motif predictions are further performed for the identified protein sequences to verify their consistence with domain prediction results.

The identified numbers of genes and transcripts of the five families in the four species, as well as database reporting ones, are summarized in Tab.1. The *B. napus* genome encompasses 518 BnAP2/EREBPs, 252 BnbZIPs, 721 BnMYBs, 398 BnNACs and 278 BnWRKYs. The *B. rapa* genome encompasses 283 BrAP2/EREBPs, 126 BrbZIPs, 348 BrMYBs, 198 BrNACs and 141 BrWRKYs. While the TF numbers of *B. oleracea* are 282, 127, 370, 210 and 142 respectively (**Additional file 2**). Compared with its diploid progenitors *B. rapa* and *B. oleracea*, the number of TFs in each family is almost doubled in the allopolyploids *B. napus*. Moreover, the identified TFs of each family in the four species are different from the existing databases more or less, and include some novel members or results.

Table 1. The numbers of genes and transcripts of the five TF families in *A. thaliana*, *B. rapa, B. oleracea* and *B. napus*.

Species	Sources	AP2/EREBP		bZIP		MYB		NAC		WRKY	
		Genes	Transcripts	Genes	Transcripts	Genes	Transcripts	Genes	Transcripts	Genes	Transcripts
A. thaliana	**Our**	**141**	**170**	**72**	**120**	**196**	**269**	**112**	**137**	**71**	**84**
	PlantTFDB	146	176	74	127	144	168	113	138	72	90
	TAIR	149	149	73	73	131	131	96	96	72	74
B. rapa	**Our**	**283**	**283**	**126**	**126**	**348**	**348**	**198**	**198**	**141**	**141**
	PlantTFDB	323	323	200	200	293	293	256	256	180	180
	BRAD	289	289	127	127	368	368	188	188	147	147
B. oleracea	**Our**	**282**	**282**	**127**	**127**	**370**	**370**	**210**	**210**	**142**	**142**
	PlantTFDB	317	317	217	217	306	306	271	271	191	191
B. napus	**Our**	**518**	**518**	**252**	**252**	**721**	**721**	**398**	**398**	**278**	**278**
	PlantTFDB	533	533	264	264	740	740	411	411	285	285

MEME motif prediction for the identified TF families

A motif is a sequence pattern that occurs repeatedly in a group of sequences. Motifs in the MEME[7] Suite are represented as position-dependent letter-probability matrices that describe the probability of each possible letter at each position in the pattern. Individual MEME motifs do not contain gaps. Patterns with variable-length gaps are split by MEME into two or more separate motifs.

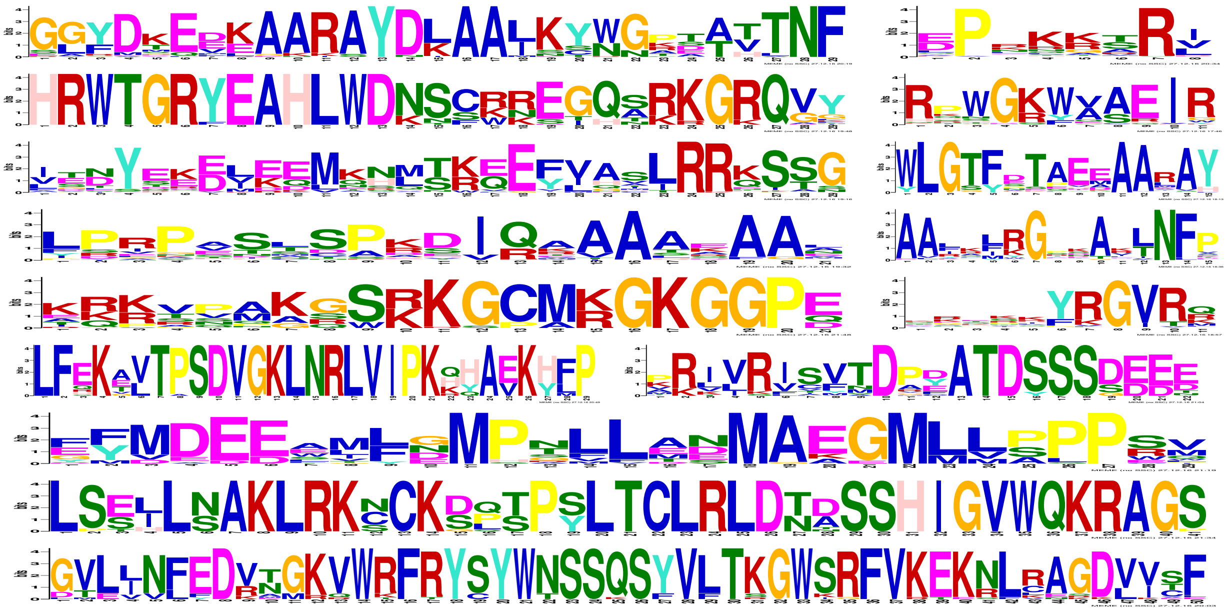

Figure 1. The identified conserved motifs in BnAP2/EREBPs by using the MEME search tool.

The identified conserved motifs for each of the five TF families by using the MEME search tool[7] are shown in Figs.1-5. Sequence logos in each figure show the conserved residues in the associated family. The $y-axis$ shows the information content[8] $IC = 4.32 - H$ (bit), here, $log_2 20 = 4.32$ denotes the maximum entropy at a certain position of multiple sequences, which corresponds to uniformly distribution of the 20 amino acids at the position. During multiple sequences alignment, one can compute the frequency distribution of the 20 amino acids at each position, and obtain the entropy[8] H for the considered position according to

$$H = -\sum_{i=1}^{20} p_i log_2 p_i. \tag{1}$$

Here, p_i denotes the frequency of the $i'th$ amino acid, and $\sum_{i=1}^{20} p_i = 1$. Each family is predicted with many conserved motifs. The bigger the amino acid letter, the higher the probability of the amino acid appeared at the associated position of protein sequences, and the corresponding amino acid is conserved.

Literature and databases comparison analysis for the identified five TF families

On the basis of genome-wide and transcriptomic data for *B. napus*, we have identified 518 BnAP2/EREBPs, 252 BnbZIPs, 721 BnMYBs, 398 BnNACs and 278 BnWRKYs. To compare our identified results with the existing results, we withdraw TFs collected by PlantTFDB and the latest references, and draw Venn diagrams, as show in Fig.6. We noted that the MYB TFs in PlantTFDB include 489 MYBs and 251 MYB-related genes, all of these TFs are considered. The latest references reported 132 BnAp2/EREBPs[9], 247 BnbZIPs[10], 249 R2R3 BnMYBs[11], 60 BnNACs[12]. Recently, Wu et al.[13] identified 289 while He et al.[14] identified 287 BnWRKYs in *B.napus*. Fig.6 shows that the identified BnAP2/EREBPs, BnbZIPs, BnNACs and BnWRKYs are all members of those in PlantTFDB. While, 50 of the identified 721 BnMYBs are not members of those as listed in the database. Moreover, for each family, we exclude several members in PlantTFDB or references in our work.

Compared with 87[15], 132[9] and 515 BnAP2/EREBPs[16] in *B. napus* that the references have reported, we identified 518 BnAP2/EREBPs, which include some novel members. For the 17 BnAP2/EREBPs that are not identified by us, we check their structures one by one. We find that 9 of the 17 genes (BnaC03g69940D, BnaC09g24990D, BnaA07g12190D,

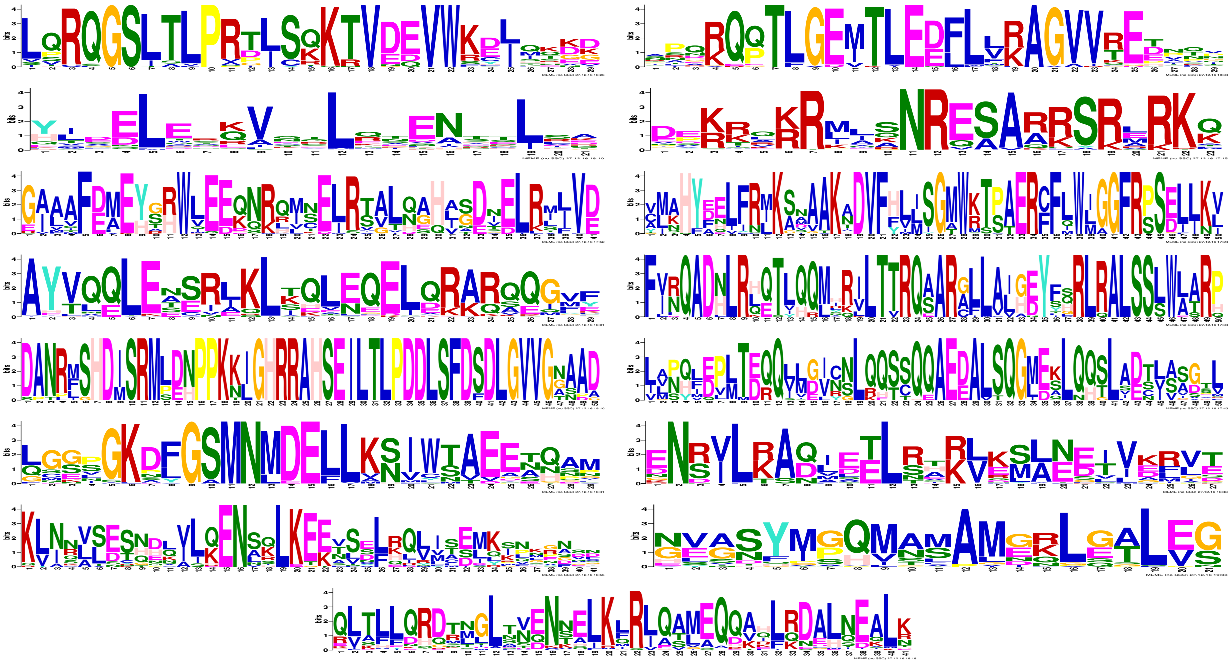

Figure 2. The identified conserved motifs in BnbZIPs by using the MEME search tool.

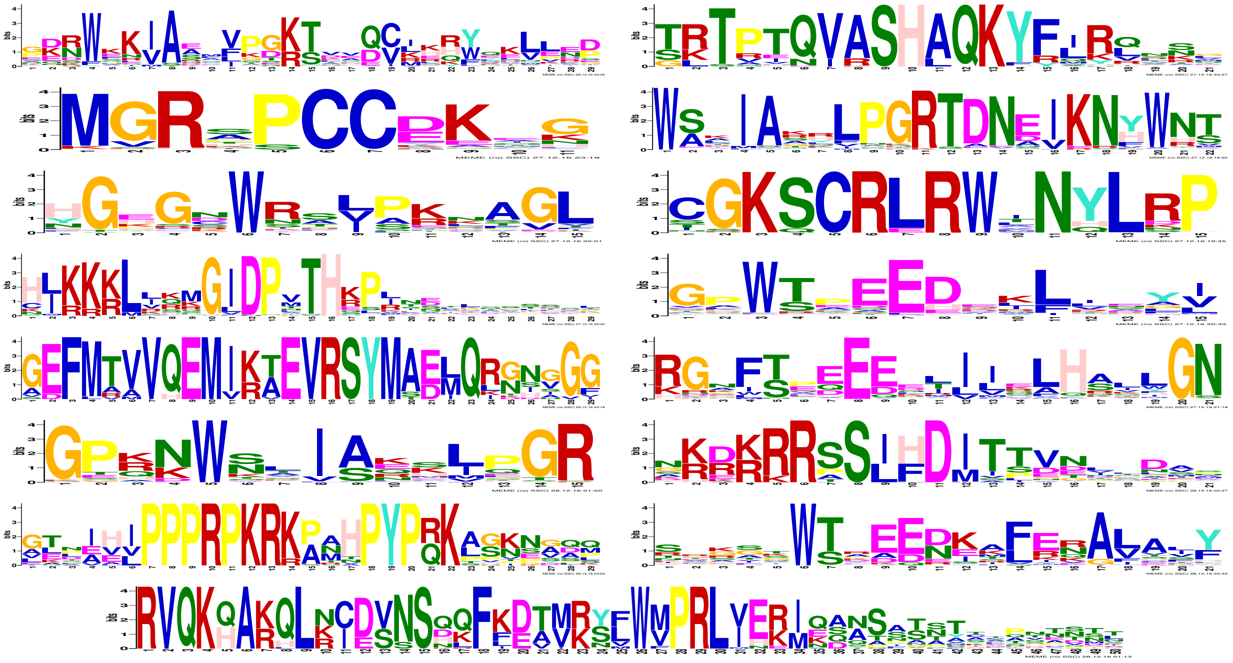

Figure 3. The identified conserved motifs in BnMYBs by using the MEME search tool.

BnaC01g34730D, BnaC01g03970D, BnaC07g50570D, BnaA08g00990D, BnaC07g48550D, BnaC02g36700D) have been not aligned to the Pfam database; Seven of the 17 genes (BnaC06g03690D, BnaC05g27060D, BnaC06g04340D, BnaA06g02760D, BnaA06g02630D, BnaC06g03900D, BnaA05g15500D) have B3 domain, but they all lack the AP2/ERF domain; Another one gene BnaA09g38470D has no structure domains as AP2/EREBPs. Existing works also identified 136 BrbZIPs[17,18] in *B. rapa*, 119 BobZIPs[19] in *B. oleracea* and 247 BnbZIPs in *B. napus*. While the numbers of bZIPs in the three species identified by our work are 126,127 and 252. Compared with the existing results, we exclude some improperly identified TFs while include other unreported ones. Hajiebrahimi et al.[11] identified 249 R2R3 BnMYBs in *B. napus*. However, we found that 424 of the

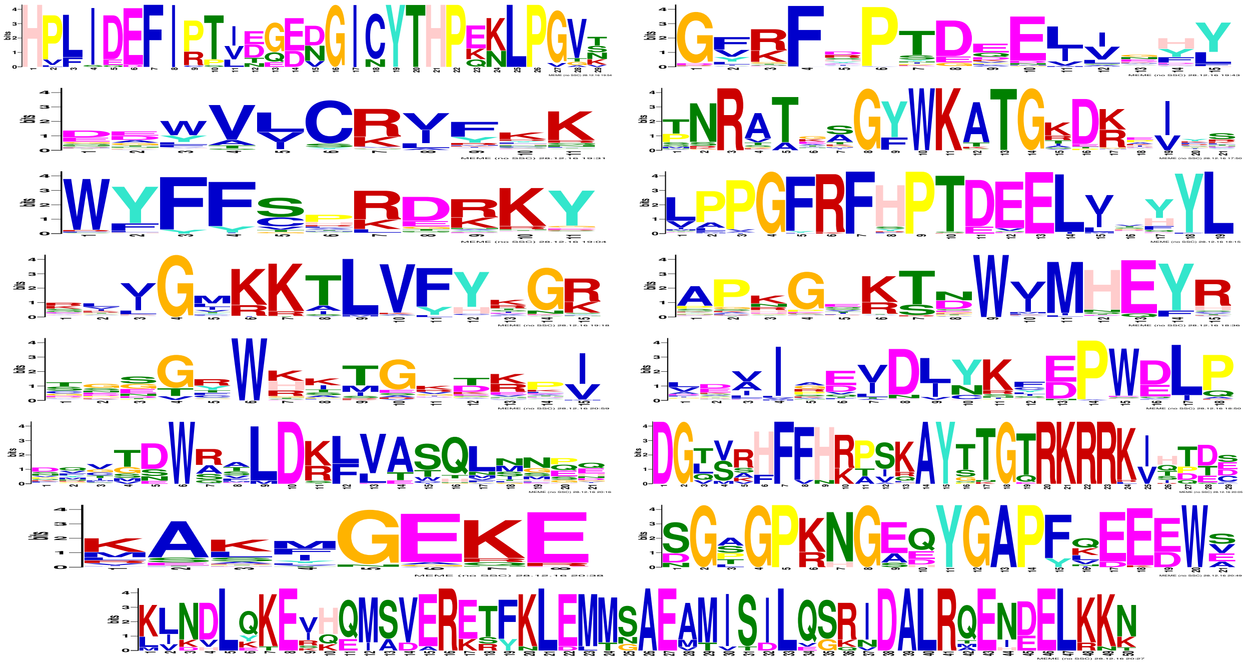

Figure 4. The identified conserved motifs in BnNACs by using the MEME search tool.

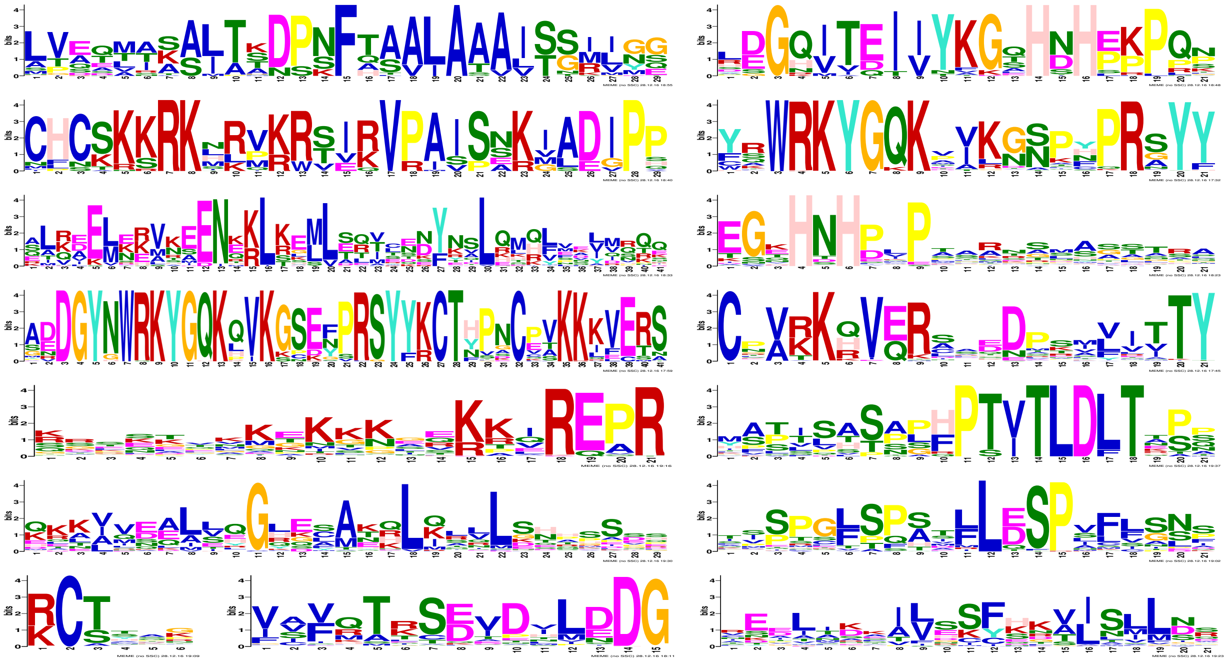

Figure 5. The identified conserved motifs in BnWRKYs by using the MEME search tool.

identified 721 BnMYBs belong to the R2R3 subfamily and include 235 of the 249 TFs reported by Hajiebrahimi et al. as members. Whereas, the left 14 of the 249 TFs (BnaA01g03490D, BnaA02g26580D, BnaA03g52540D, BnaA06g09160D, BnaAnng17310D, BnaC01g04760D, BnaC01g11200D, BnaC02g10450D, BnaC02g34810D, BnaC03g12350D, BnaC03g66570D, BnaC05g10580D, BnaC07g44270D, BnaCnng49390D) are not identified as BnMYBs by us, but they are all collected as BnMYBs in PlantTFDB. Compared with the identified 60 BnNACs from *B. napus* by Wang et al.[12], we identified 398 BnNACs, whose number is six times more than the existing references. WRKYs in various species have been extensively investigated[13,14,20–22]. Researchers have found that the WRKY amino acid sequences have been replaced by WRRY, WSKY,

WKRY, WVKY, WKKY or WKNY in some WRKY proteins[23,24]. We found that the WRKY domain of a group IIc BnWRKYs BnaCnng77260D has been replaced by WKNY, while two group IIc BnWRKYs BnaCnng28950D and BnaCnng39890D have been replaced by WKKY. The number of our initially identified WRKYs via sequence comparisons of the four species are 91, 145, 148 and 285 respectively. However, 7 transcripts in *A. thaliana*, including AT2G40740.2, AT3G32090.1, AT2G23320.2, AT3G04670.2, AT4G12020.1, AT4G12020.2, AT4G12020.3, don't have either WRKY domain or the zinc finger-like motifs with multiple sequence alignment of the WRKY domain and the zinc finger-like motifs by ClustalW. Therefore, we finally identified 84 AtWRKY transcripts in *A. thaliana*. The two genes Bra008456 and Bra036563 in *B. rapa* don't have WRKY domain. Moreover, although the two genes Bra034482 and Bra037637 have WRKY domains, but don't have zinc finger motifs. Thus, we finally identified 141 BrWRKYs in *B. rapa*. Similarly, 6 of the 148 *B. oleracea* transcripts don't have either WRKY domains or zinc finger motifs, which includes Bo2g098820.1, Bo9g064170.1, Bo7g093150.1, Bo9g064180.1, Bo6g071100.1 and Bo2g024010.1. Thus, only 142 BoWRKYs are identified in *B. oleracea*. Further domain analysis reveals that the *B. napus* gene BnaA02g19590D has inconsistent WRKY domain; the six *B. napus* genes BnaC04g19430D, BnaC05g14560D, BnaC02g09600D, BnaC07g25890D, BnaA02g02500D, BnaA01g13720D all lack the zinc finger motifs. Therefore, we finally confirmed 278 BnWRKYs in *B. napus*. Compared with PlantTFDB[20] and the 289 BnWRKYs reported by Wu et al.[13], the finally identified BnWRKYs are relatively fewer. Compared Ref.[13] with our results, 271 genes are common. And 18 genes are exclusively reported by Wu et al, which don't have either WRKY domains or zinc finger motifs. For example, BnaA01g13720D lacks the zinc finger motif, thus it is actually not a WRKY gene. Some novel members, such as BnaA06g13050D and BnaA09g21040D, are confirmed to be BnWRKYs by us, which actually have the defining features of WRKYs. Our new results indicate that the reported WRKYs in existing databases or references may contain inaccurate information, or some special genes/transcripts do not follow the usual structures.

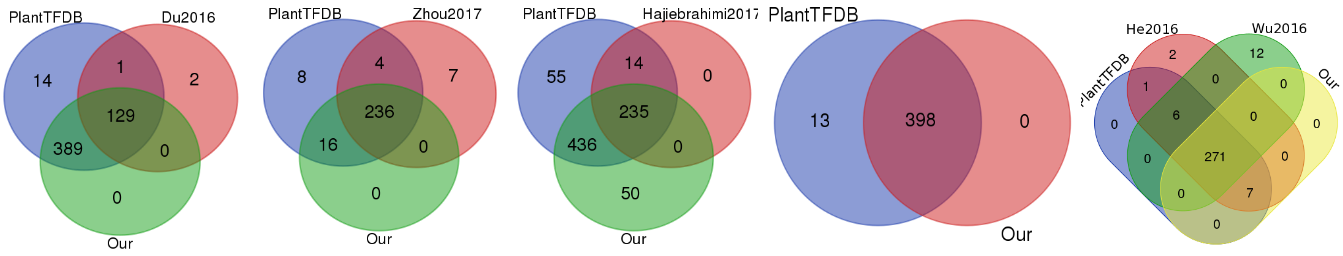

Figure 6. Comparison our identified results with literature and databases.

Non-parametric χ^2 goodness of fit test for chromosome distributions

To test whether the distributions of TFs on the 10 A sub-genomes between *B. rapa* and *B. napus*, the 9 C sub-genomes between *B. oleracea* and *B. napus* are statistically different, we use the following non-parametric χ^2 goodness of fit statistics[25]:

$$\chi^2 = \sum_{i=1}^{m} \frac{(f_i - e_i)^2}{e_i}. \tag{2}$$

Here, $m = 10$ for the A sub-genome and $m = 9$ for the C sub-genome. f_i represents the amount of TFs on the $i'th$ chromosome of *B. napus* (Observed counts). e_i denotes the number of TFs on $i'th$ chromosome of *B. rapa* or *B. oleracea* (Expected counts). The test statistic (2) has an approximate chi-square distribution with degree of freedom $m-1$.

The null hypothesis of the non-parametric χ^2 goodness of fit test is that the distributions of TFs on the 10 A sub-genomes between *B. napus* and *B. rapa* are without significant difference, and distributions of TFs on the 9 C sub-genomes between *B. napus* and *B. oleracea* are also without significant difference. The test results are summarized in Tab.2. It reveals that the distributions of BnbZIPs, BnMYBs, BnNACs and BnWRKYs on the A sub-genomes in *B. napus* are not significantly different from that in *B. rapa* ($p > 0.05$), however, for BnAP2/EREBPs, such distribution in *B. napus* is significantly different from *B. rapa* ($p = 0.0253 < 0.05$). For the C sub-genomes, distributions of BnAP2/EREBPs ($p = 0.0140 < 0.05$) and BnMYBs ($p = 0.0071 < 0.05$) in *B. napus* are all significantly different from those in *B. oleracea*, while distributions of the other three TF families in the two species have no statistical differences ($p > 0.05$). The results indicate that the BnAP2/EREBPs and BnMYBs may have suffered from duplication or divergence effect during evolution from its diploid progenitors

Multiple amino acid sequences alignment for the identified five TF families

Multiple sequences alignment analysis for the identified TFs in each family were performed by CLUSTAX[26] 2.0.11(http://www.clustal.org/clustal2/) and DNAMAN 7.0 (http://www.lynnon.com).

Table 2. Non-parametric χ^2 chromosome distribution tests between *B. napus* and *B. rapa* or *B. oleracea*.

B.rapa A chro.	*B.napus* A chro.	AP2/EREBP		bZIP		MYB		NAC		WRKY	
		B.rapa	*B.napus*	*B.rapa*	*B.napus*	*B.rapa*	*B.napus*	*B.rapa*	*B.napus*	*B.rapa*	*B.napus*
A01	chrA01	23	17	9	7	32	27	19	20	12	7
A02	chrA02	33	25	13	9	43	26	25	15	19	11
A03	chrA03	40	31	13	12	60	58	29	26	26	24
A04	chrA04	15	10	13	11	16	12	5	6	15	16
A05	chrA05	21	16	11	10	34	31	19	17	11	8
A06	chrA06	23	16	17	13	32	34	16	13	11	9
A07	chrA07	33	27	8	7	34	29	21	19	8	9
A08	chrA08	31	23	9	9	22	21	8	7	11	8
A09	chrA09	40	28	25	19	43	30	27	23	20	17
A10	chrA10	21	15	7	9	27	25	28	21	4	4
χ^2		18.9869		5.2283		13.8177		7.9941		8.1723	
DF		9		9		9		9		9	
P value		**0.0253**		0.8140		0.1290		0.5347		0.4168	
B.oleracea C chro.	*B.napus* C chro.	*B.oleracea*	*B.napus*	*B.oleracea*	*B.napus*	*B.oleracea*	*B.napus*	*B.oleracea*	*B.napus*	*B.oleracea*	*B.napus*
C01	chrC01	23	14	9	7	38	24	23	15	10	9
C02	chrC02	32	23	12	10	42	29	28	19	17	13
C03	chrC03	41	29	20	16	66	51	32	25	29	24
C04	chrC04	25	20	19	12	29	22	11	13	25	19
C05	chrC05	27	22	11	9	36	30	32	29	6	7
C06	chrC06	30	21	11	10	29	22	18	14	10	10
C07	chrC07	34	27	12	8	39	33	18	15	19	17
C08	chrC08	32	25	15	14	37	30	11	11	9	10
C09	chrC09	32	24	16	15	45	36	31	18	14	10
χ^2		19.1635		6.0738		21.0175		14.6921		4.9744	
DF		8		8		8		8		8	
P value		**0.0140**		0.6390		**0.0071**		0.0654		0.7603	

As an example, we show the highly conserved sections for the 278 WRKYs in Fig.7. Researchers have found that the WRKY amino acid sequence have been replaced by WRRY, WSKY, WKRY, WVKY, WKKY or WKNY in some WRKY proteins[23,24]. For the 278 BnWRKYs, 275 proteins are with the usual WRKY amino acid sequences. However, the WRKY amino acid sequences for three group IIc BnWRKYs have been replaced by WKKY or WKNY. Actually, the BnaCnng77260D has been replaced by WKNY, while BnaCnng28950D and BnaCnng39890D have been replaced by WKKY.

The WRKY genes can be classified into three subfamilies[27]. Subfamily I WRKY proteins have two WRKY domains, the N-terminal (NT) domain and the C-terminal (CT) domain, as shown in Fig.7. Most of WRKY genes with one WRKY domain belong to subfamily II. Both subfamily I and II WRKY proteins have the same zinc motifs. WRKY proteins with only one WRKY domain but different patterns of zinc finger motifs are categorized into subfamily III. Subfamily III WRKY domains contain a C2-HC motif (C-X7-C-X23-H-X1-C). Subfamily II proteins can be further divided into IIa to IIe based on the primary amino acid sequences. Among the identified 278 BnWRKYs in *B. napus*, detailed analysis on their amino acid sequences reveal that 49 genes belong to subfamily I, 183 genes are subfamily II, while 46 genes are classified into subfamily III. For the four species, subfamily II WRKYs take up more than one half, especially, more than 25% WRKYs belong to group IIc.

Phylogenetic trees and subfamilies distributions of the identified five TF families

Phylogenetic tree analysis was performed using MEGA by neighbor-joining method. The unrooted phylogenetic trees for the five families are shown in Fig.8. In each panel, we show the cases for *B. napus* in comparison to *A. thaliana*, *B. rapa*, *B. oleracea* and *B. napus*. Subfamilies distributions of the identified five families of TFs are shown in Fig.9.

From the constructed phylogenetic trees and subfamilies distributions, we observe that TFs in each family can be classified into several different clades. Specifically, the BnAP2/EREBPs can be divided into five clades consisting of four subfamilies: AP2, ERF, DREB and RAV. The two super-families DREBs and ERFs in *B. napus* were further distributed into six subgroups, A1 to A6, B1 to B6, respectively. We found that more than 10% AP2/EREBPs in the three *Brassica* species belong to ERF B3, DREB A4 and ERF B1 respectively. However, more than 15% AtAP2/EREBPs in *A. thaliana* are classified into ERF b6. Specifically, for *B. napus*, we find that a total of 194 DREBs were distributed into groups A1-A6, containing 17, 30, 3, 66, 42 and 36 genes respectively. Additionally, 244 ERFs were distributed into groups B1-B6 containing 61, 18, 76, 21, 25, and 43 genes respectively, with 28 ERFs can not be assigned to any specific subgroups. Finally, 32 genes were classified into the AP2 subfamily and 20 genes are assigned into the RAV subfamily. The AtbZIPs in *A. thaliana* can be classified into 7 subfamilies, while the bZIPs in *B. rapa, B. oleracea* and *B. napus* can be divided into 9, 11 and 12 subfamilies. Almost 50% AtbZIPs (59) in *A. thaliana* belong to subfamily I, while only 12 of the 518 BnbZIPs are subfamily I in *B. napus*. In *B. napus*, subfamily III BnbZIPs is the biggest subfamily, which takes up about one quarter (63), while members in subfamilies XI and VI are both less than 5. For the MYBs, most of TFs in the four species belong to the 1R and R2R3 super-families. For NACs, the AtNACs in *A. thaliana* can be classified into 14 subfamilies, and those in the three *Brassica* genus can be divided into 21, 16

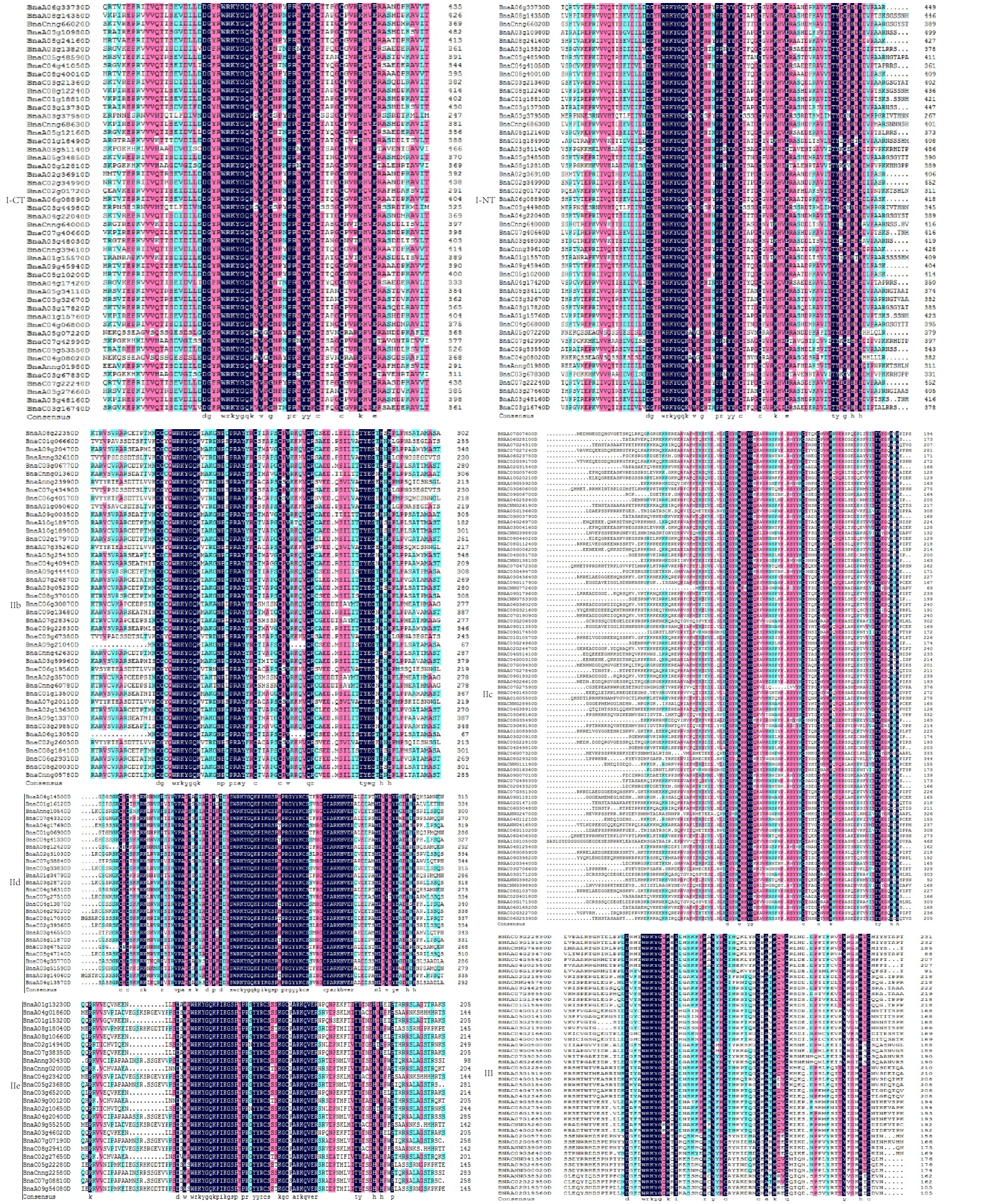

Figure 7. Amino acid sequence alignment of the BnWRKYs in B. napus. Identical amino acids are indicated by white letters on black grounds, and conserved amino acids by black on red backgrounds.

and 17 subfamilies. More than 20% BoNACs in *B. oleracea* belong to subfamilies X and IV respectively. The *B. napus* is most enriched in subfamily VI, while *B. rapa* is with more than 15% BrNACs in subfamily XIII. Among the identified 278 BnWRKYs in *B. napus*, detailed analysis on their amino acid sequences reveal that 49 genes belong to subfamily I, 183 genes are subfamily II, while 46 genes are classified into subfamily III. For the four species, subfamily II WRKYs takes up more than one half, where more than 25% WRKYs belong to group IIc. Interestingly, we have not found BnWRKY members in group IIa in *B. napus*. In fact, literature reported group IIa WRKYs are classified into group IIb in our work, and sequences of groups IIa and IIb proteins are with high similarity. The numbers of group specific WRKYs in each species can be found in Tab.3.

Table 3. The numbers of subfamily specific WRKYs in *B. rapa, B. napus, B. oleracea* and *Arabidopsis*.

Group	Types	*B. rapa*	*B. napus*	*B. oleracea*	*A. thaliana*
Group I	Gene number	24	49	25	12
	Transcript number	24	49	25	17
Group II	Gene number	93	183	96	46
	Transcript number	93	183	96	52
Group III	Gene number	24	46	21	13
	Transcript number	24	46	21	15
Total	Gene number	141	278	142	71
	Transcript number	141	278	142	84

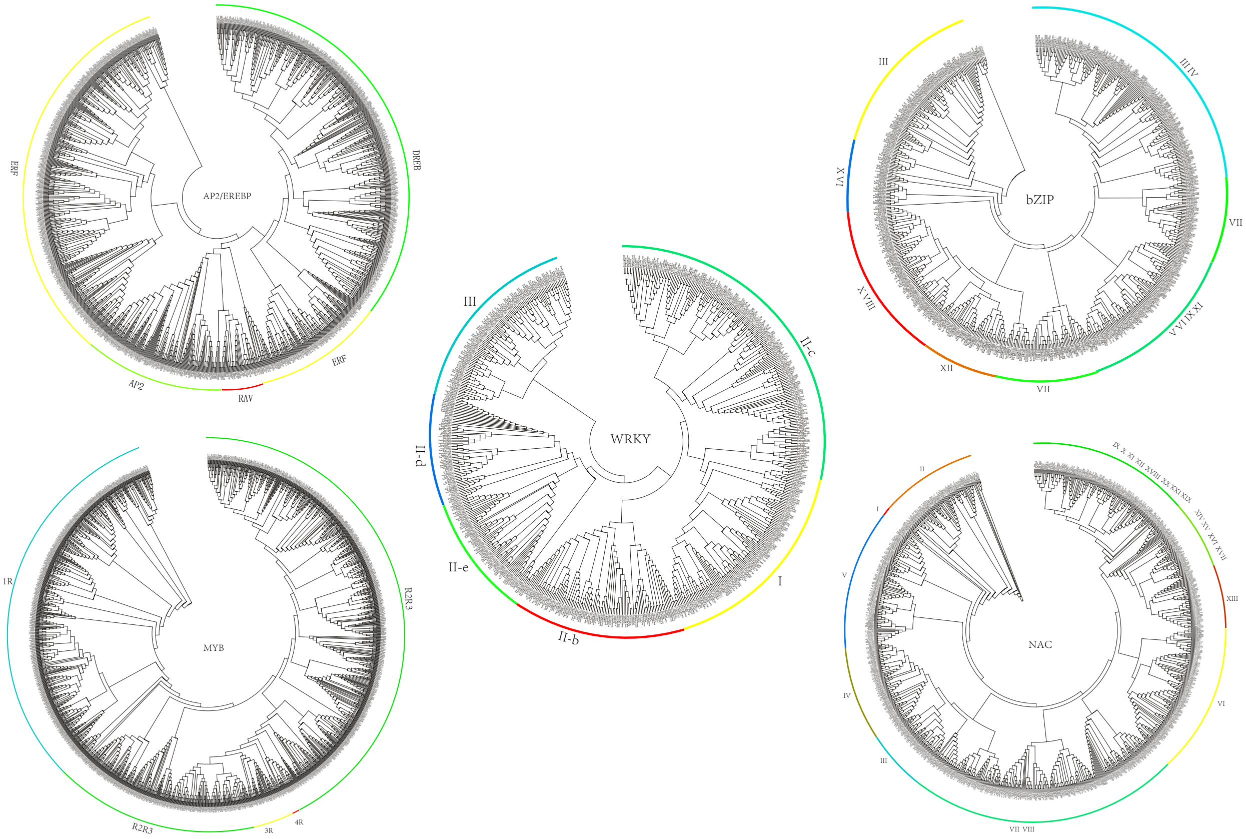

Figure 8. The unrooted phylogenetic trees constructed using MEGA by neighbor-joining method for the five TF families. The five panels correspond to the AP2/EREBPs, bZIPs, MYBs, NACs and WRKYs respectively. In each panel, we show the cases for *B. napus* in comparison to *A. thaliana*, *B. rapa*, *B. oleracea* and *B. napus*.

Heat maps for cis-acting elements of the five families

The five TF families are rich in various hormone or stress responsive cis-acting elements. In this paper, we consider 11 cis-acting elements, for details, see Tab.4. Heat maps of cis-acting elements for the five TF families can be found in Fig.10. Fig.10 reveals that the five families are all rich in MBS, TC-rich repeats, HSE and TGACG motif. Whereas, the numbers of TFs with P-box, TGA element and LTR are relatively rare.

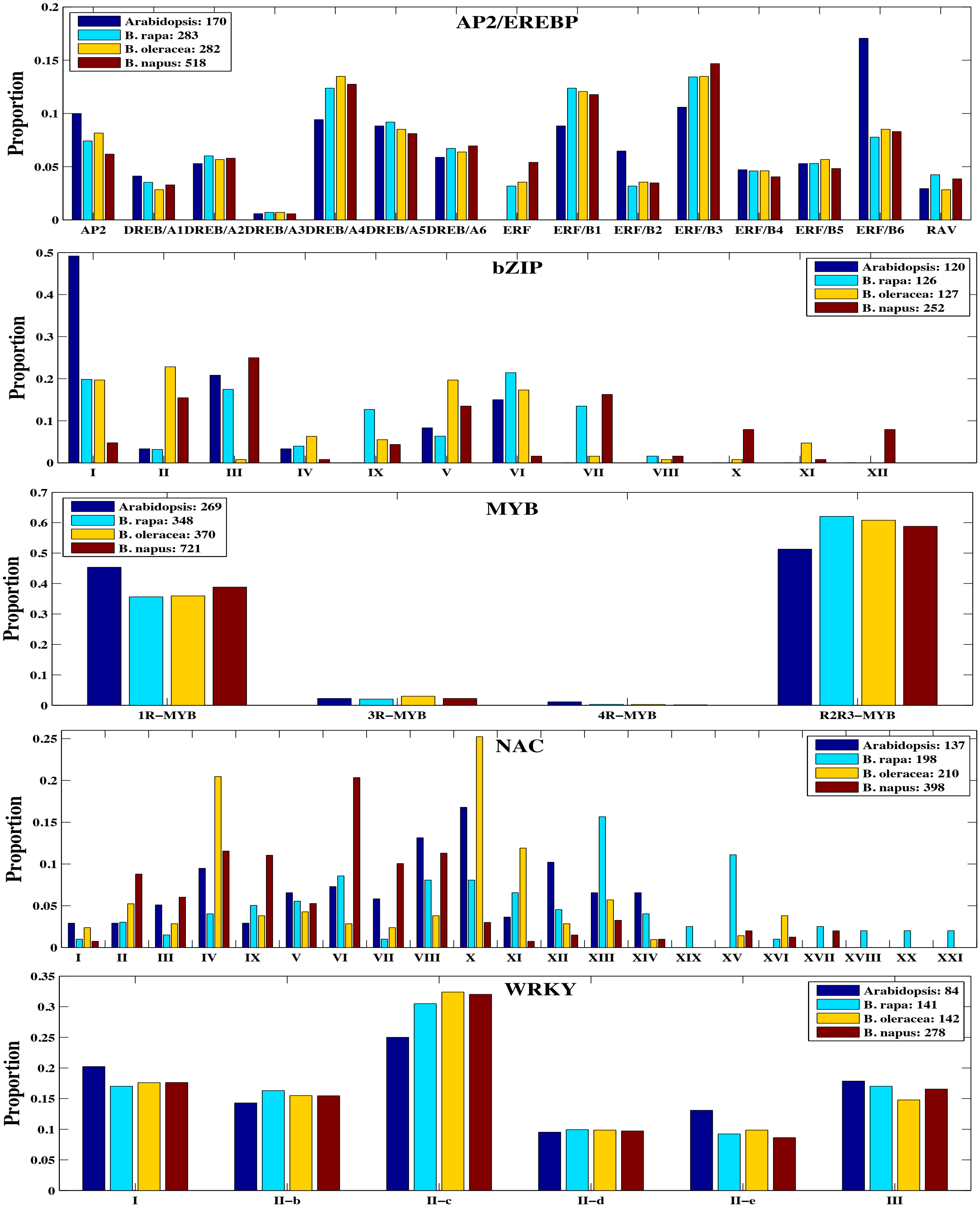

Figure 9. Proportions of group specific TFs in each family for the four species.

Table 4. Cis-acting elements that are considered in this paper.

Cis-acting elements	Functions	Cis-acting elements	Functions
ABRE	ABA-responsive	GARE-motif	Gibberellin responsive
P-box	Gibberellin responsive	MBS	Drought responsive
HSE	Heat stress responsive	W box	WRKY binding sites
TGACG-motif	MeJA-responsiveness	TGA-element	Auxin-responsive element
TCA-element	SA responsiveness	TC-rich repeats	Defense and stress responsiveness
LTR	Low-temperature responsiveness		

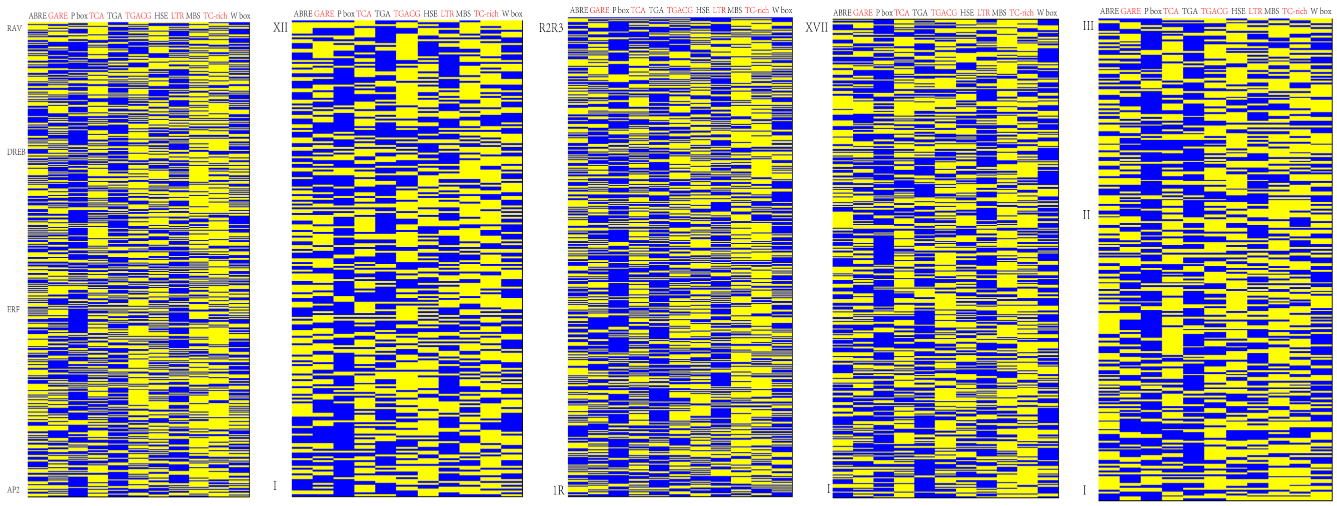

Figure 10. Heat maps of cis-acting elements for the five TF families. Yellow blocks denote that the corresponding genes have the associated cis-acting elements, while green blocks denote the corresponding genes do not have the corresponding cis-acting elements.

Synteny analysis of the five TF families in *B. napus*

Homologous genes were predicted by means of synteny-based methods in the CoGe database (https://genomevolution.org)[28]. Orthologous genes of *B. napus* were predicted in the target genomes of *A. thaliana, B. rapa*, and *B. oleracea* through the identification of a conserved syntenic region via SynFind with the following parameters: last comparison algorithm, minimum number of four genes, and unlimited syntenic depth. Putative paralogous genes in *B. napus* were identified via SynMap by the following parameters: last algorithm, 20 genes for the maximum distance between two genes, and five genes for the minimum number of aligned pairs. The algorithm for syntenic depth was defined as quota-align with a coverage depth of 1:1 for *B. napus* versus *B. rapa* and *B. oleracea*. The coverage depth of *B. napus* versus *A. thaliana* was determined at 6:1. Orthologous sets were visualized via McScanx (http://chibba.pgml.uga.edu/mcscan2/).

The identified five families of sequences in *B. napus* are evolutionary homologous with its diploid progenitors *B. rapa* and *B. oleracea*. Synteny analysis among the A sub-genomes of *B. rapa* and *B. napus*, the C sub-genomes of *B. oleracea* and *B. napus* show strong co-linearity, although chromosomal rearrangements and gene duplication events have occurred between *B. napus* and parental species after their divergence from the common ancestor (**Additional file 3**). Synteny analysis allows us to characterize four categories of genes: 1) Novel genes, which emerged during the evolution from parental species to *B. napus*. 2) Conserved genes, which have a unique copy from *B. rapa, B. oleracea* to *B. napus*. 3) Multi-copy genes. If two or more genes from the same species were detected in a homologous gene set of the four species[29]. 4) Lost genes[29], which were missing during the evolution from parental genomes to *B. napus*. There are 33, 22, 65, 29, 19 novel, and 66, 26, 54, 30, 24 conserved BnAP2/EREBPs, BnbZIPs, BnMYBs, BnNACs and BnWRKYs among the identified TFs in *B. napus* respectively. While 419 BnAP2/EREBPs, 204 BnbZIPs, 602 BnMYBs, 339 BnNACs and 235 BnWRKYs are multi-copy genes. Novel genes averagely take up about 7.6% in the five families. BnMYBs posses the most novel genes (9.0%), followed by BnbZIPs (8.7%). BnAP2/EREBPs contain the lowest novel genes (6.4%), and BnWRKYs contain about 6.8% novel ones. About 9.4% TFs are conserved in *B. napus* in the five families, including 12.7% BnAP2/EREBPs, and about 7.5% BnMYBs and BnNACs. More than 80% TFs in *B. napus* are multi-copy ones. The BnNACs and BnWRKYs contain the most multi-copy genes (about 85%), while BnAP2/EREBPs and BnbZIPs contain about 80% multi-copy ones. For the A sub-genome, a total of 30 TFs lost during the evolution from *B. rapa* to *B. napus*, including 12 BrAP2/EREBPs, 7 BrbZIPs, 8 BrMYBs, 1 BrNACs and 2 BrWRKYs. For

the C sub-genome, a total of 55 TFs lost during the evolution from *B. oleracea* to *B. napus*, including 14 BoAP2/EREBPs, 5 BobZIPs,16 BoMYBs,16 BoNACs and 4 BoWRKYs. Apparently, gene loss in the AP2/EREBPs and MYBs is considerable.

Statistical analysis reveals that novel, conserved and lost genes prefer the C sub-genome, while multi-copy genes show no sub-genome preferences (**Additional file 3**). Specifically, 36 BnMYBs are novel genes, and 44 BnAP2/EREBPs are conserved genes, which are all positioned on the C sub-genome, and take up the most, compared with other families and the A sub-genome. For multi-copy genes, both the A and C sub-genomes contain about 40% members in each family, and show no sub-genome preferences. Chromosomes chrC03, chrC04, chrA06 and chrA09 are rich in novel genes (> 10). Chromosomes chrC03, chrC01, chrA03 and chrA08 are rich in conserved genes. More than 100 multi-copy genes are anchored onto chromosomes chrA03, chrA09 and chrC03. Statistical analysis also reveals subfamily preferences for the four categories of genes. In fact, one third of novel genes in BnAP2/EREBPs are from the ERF B3 subfamily, subfamilies DREB A4 (16) and ERF B3 (15) contain the most conserved BnAP2/EREBPs, and ERF B1(54) and ERF B3 (50) have the most multi-copy genes, while 7 of the 12 lost BrAP2/EREBPs and 9 of the 14 lost BoAP2/EREBPs are from the ERF super-subfamily. For the bZIPs, subfamily VII is rich in novel genes, V and XII are rich in conserved genes, III is enriched with multi-copy genes, while the 12 lost bZIPs from *B. rapa, B. oleracea* to *B. napus* show no subfamily preferences. The R2R3 subfamily of BnMYBs contains the most novel, conserved and multi-copy genes. Whereas, 17 of the 24 lost MYBs belong to the 1R subfamily, the lost MYBs especially prefer subfamily 1R. For the BnNACs, subfamilies VI, II and IV all contain relative much more novel genes. IV contains 12 conserved genes, and VI contains 70 multi-copy genes. Only 1 BrNAC is lost from *B. rapa* to *B. napus*, while 16 BoNACs are lost from *B. oleracea* to *B. napus*, and 7 of the 16 BoNACs belong to the IV subfamily, 5 and 4 members are positioned on chromosomes C5 and C2 respectively. Group IIc of BnWRKYs contains the most novel genes, conserved genes and multi-copy genes, and their numbers are 10, 13 and 66 respectively. Subfamilies I and III both contain a considerable amount of multi-copy genes. Among the 6 lost WRKYs, 5 are from subfamily II, and only one is from subfamily III.

Synteny analysis among the A sub-genomes of *B. rapa* and *B. napus*, the C sub-genomes of *B. oleracea* and *B. napus* have strong co-linearity, although chromosomal rearrangements and gene duplication events have occurred among *B. napus*, *B. rapa* and *B. oleracea*.

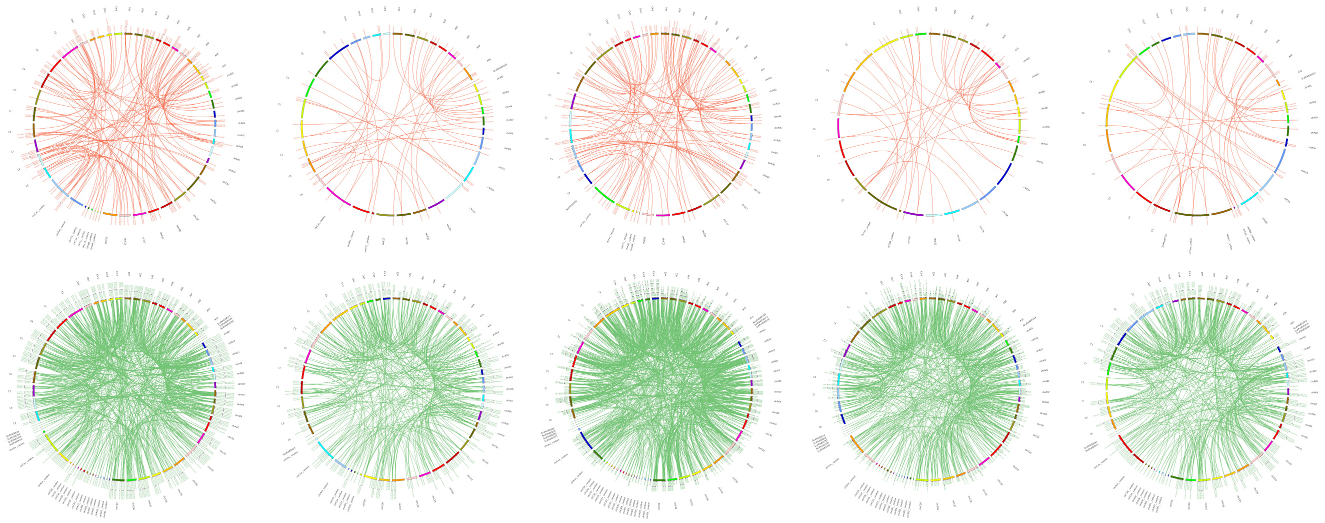

Figure 11. Synteny analysis for single-copy genes (UP) and multi-copy genes (Down) among *Arabidopsis*, *B. rapa*, *B. oleracea* and *B. napus*. The genes in the four species are plotted against their linked counterpart chromosomes. From left to right, we show the cases for AP2/EREBP, bZIP, MYB, NAC and WRKY. From outside to inside of each circos plot, the first layer shows the chromosome names, A* corresponds to *B. rapa*, chr* represnts *B. napus*; C* and S* represent *B. oleracea* and Scaffold, respectively; Chr* represents *Arabidopsis*. The second layer shows gene ID (partially); The third layer denotes length of chromosomes; The last layer links the gene IDs with synteny. Synteny block analysis reveals changes in transcription factors during the evolution from diploid to tetraploid.

Transcriptome sequencing data and results

We consider five treatments, including cold treatment at 4°C low temperature, heat treatment at 40°C high temperature, drought treatment with 15% PEG-6000, salt treatment with 150mM NaCl and ABA stimulus with 30 mmol/L ABA. The 7 days old

seedlings were treated under each condition lasting 12 hours for transcriptional sequencing. Three repeats are considered under each treatment. Totally 18 samples (5 conditions $\times$ 3 repeats) are sequenced using Illumina Hiseq platform, including 15 treated samples and 3 control samples without any treatments. On average we generated about 6.56Gb bases from each sample. Averagely 72.09% clean reads are mapped to reference genome. After mapping sequenced reads to reference genome and reconstructing transcripts, we finally identify 53948 genes on average for each sample, including 50815 known genes and 3133 novel genes on average for each sample. For details of the RNA-Seq results, see Tab. 5.

Table 5. Summary of sequencing reads after filtering, genome mapping and expressed genes. RL: Read Length(bp); TMR: Total Mapping Ratio; UMR: Uniquely Mapping Ratio; Uniquely Mapping: Reads that map to only one location of reference, called uniquely mapping. Q20: the rate of bases which quality is greater than 20.

Sample	Clean reads	Clean bases	Q20(%)	Q30(%)	GC(%)	RL(bp)	TMR	UMR	Expressed genes	Known genes	Novel genes
Cold-1	44130284	6619542600	97.73	93.65	48.08	150	73.19%	67.97%	54732	51682	3050
Cold-2	44417508	6662626200	97.78	93.97	46.43	150	72.61%	66.99%	53200	50208	2992
Cold-3	43794678	6569201700	97.50	93.10	48.07	150	69.05%	64.87%	52543	49555	2988
Heat-1	45006690	6751003500	97.72	93.60	47.37	150	72.89%	68.90%	56286	52862	3424
Heat-2	43065052	6459757800	97.61	93.47	47.29	150	73.04%	69.02%	51781	48742	3039
Heat-3	43232014	6484802100	97.44	92.56	47.22	150	72.37%	68.34%	50508	47589	2919
Droug.-1	43793428	6569014200	97.73	93.64	47.98	150	73.07%	68.26%	55697	52541	3156
Droug.-2	43315888	6497383200	97.66	93.60	47.63	150	73.57%	68.92%	54842	51646	3196
Droug.-3	44269414	6640412100	97.33	92.67	48.03	150	70.23%	65.68%	55138	51704	3434
ABA-1	44362456	6654368400	97.64	93.44	47.81	150	72.64%	67.86%	55537	52365	3172
ABA-2	43276176	6491426400	97.71	93.73	47.61	150	73.05%	68.43%	53716	50547	3169
ABA-3	43924378	6588656700	97.67	93.47	47.83	150	71.60%	67.26%	54165	50949	3216
Salt-1	43204130	6480619500	97.73	93.71	47.94	150	74.24%	69.51%	52646	49627	3019
Salt-2	43169818	6475472700	97.34	92.33	47.89	150	70.82%	66.01%	51603	48620	2983
Salt-3	43682002	6552300300	97.50	93.11	47.56	150	70.99%	66.82%	55093	51808	3285
Contr.-1	43226218	6483932700	97.68	93.63	48.00	150	74.09%	69.19%	54001	51002	2999
Contr.-2	44152200	6622830000	97.22	92.07	47.66	150	68.98%	64.92%	55535	52348	3187
Contr.-3	43156454	6473468100	97.63	93.60	47.80	150	71.26%	67.07%	54046	50882	3164

Enrichment analysis of cis-acting elements for the identified 315 crucial DEGs

To explore the enriched cis-acting elements for the selected DEGs, we use Zscore[30] to quantify the relative abundance of each cis-acting element, defined as:

$$Zscore = \frac{F_{ij} - \bar{R}_{ij}}{std(R_{ij})}. \qquad (3)$$

Here, F_{ij} denotes the actual number of curated DEGs in the $i'th$ ($i =$BnAP2/EREBP, BnbZIP, BnMYB, BnNAC, BnWRKY) family with the $j'th$ ($j =$ABRE, GARE motif, P box,TCA element,TGA element, TGACG motif, HSE, LTR, MBS, TC-rich repeats, W box) cis-acting element. R_{ij} is a vector with 5000 elements, with each element denotes the number of DEGs with the $j'th$ element, where the 5000 rounds of randomly sampled amounts of DEGs equal to the amount of curated DEGs in the $i'th$ family. $std(R_{ij})$ represents the standard deviation of R_{ij}.

$Zscore$ for each cis-acting element of each family is shown in Tab.6. Hereinafter, $Zscore > 0$ represents enriched element, while $Zscore < 0$ indicates underrepresented element. Table 6 shows that the identified 93 BnAP2/EREBPs are especially enriched with TGA element with $Zscore = 1.9025$, but are underrepresented with TCA element, TC-rich repeats and W-box. Actually, 45 of the 93 BnAP2/EREBPs are with TGA element, however, for randomly sampled 5000 sets of BnAP2/EREBPs with 93 DEGs, the average number of genes with TGA element is around 32. The 42 BnbZIPs are enriched with ABREs, but are underrepresented with TCA element. Actually, 29 of the 42 BnbZIPs are with ABREs, which is higher than the average number 21.7 for 5000 randomly sampled sets. Though 21 of the 42 BnbZIPs are with TCA element, as compared with the average number 26.5 for 5000 sets of randomly sampled genes, it is relatively lower for the 42 crucial BnbZIPs. The 94 BnMYBs are enriched with HSE, P box, GARE motif, TGA element, however, they are shorted in TC-rich repeats and W box. The 48 BnNACs are enriched with GARE motif and TGACG motif, but they are underrepresented with four cis-acting elements, including TGA element, HSE, MBS and TC-rich repeats. For the 38 BnWRKYs, results reveal that they are enriched with MBS and TC-rich repeats, but are shorted in ABRE and HSE.

Topological analysis on subnetworks of the constructed gene co-expression network

The constructed gene co-expression network consists of five families of genes, we withdraw subnetworks for each family and draw the corresponding co-expression networks, as shown in Fig.12. For the constructed co-expression networks, we perform detailed topological analysis. Table 7 shows the statistics information[31] on the constructed gene co-expression network and the

Table 6. Enrichment analysis of cis-acting elements for the identified DEGs in each family.

Cis-acting elements	BnAP2/EREBP	BnbZIP	BnMYB	BnNAC	BnWRKY
ABRE	-0.8741	**2.4274**	0.0341	-0.1116	-2.5577
GARE motif	0.6134	0.4492	**1.4008**	**1.4274**	0.3449
P box	0.4675	-0.4646	**2.2236**	-0.3771	0.0460
TCA element	-2.9553	-1.9508	-0.5340	-0.9092	0.3184
TGA element	**1.9025**	-0.1941	**1.1770**	-1.0648	-0.0665
TGACG motif	0.1088	-0.0020	-0.0870	**1.3687**	-0.4236
HSE	0.3256	-0.5685	**2.2938**	-1.2987	-1.2039
LTR	0.1636	0.0128	0.7830	-0.0987	-0.7035
MBS	0.3159	0.8192	-0.2604	-1.5111	**1.5024**
TC-rich repeats	-1.6434	0.8350	-2.6498	-1.4184	**1.9515**
W box	-1.0628	-0.5705	-1.5496	0.0838	0.7508

corresponding five subnetworks as shown in Fig.12. Specifically, average degree of undirected and unweighted network is defined as

$$< k >= \frac{2E}{N}. \tag{4}$$

E is the number of edges, and N is the number of nodes in the complex network respectively. The link density of the network is calculated as:

$$LD = \frac{2E}{N(N-1)}. \tag{5}$$

Here, $\frac{1}{2}N(N-1)$ represents all possible edges between any two of the N nodes in the complex network. Higher degree or higher link density indicates each node may have more neighbors.

If high degree nodes tend to connect with high degree ones, then the network is assortative; whereas, if high degree nodes tend to connect with low degree ones, then the network is disassortative. The degree-degree correlation coefficient DCC[32] can act as an indicator of assortativity and disassortativity, which is defined as

$$DCC = \frac{M^{-1}\sum_i j_i k_i - [M^{-1}\sum_i \frac{1}{2}(j_i+k_i)]^2}{M^{-1}\sum_i \frac{1}{2}(j_i^2+k_i^2) - [M^{-1}\sum_i \frac{1}{2}(j_i+k_i)]^2}. \tag{6}$$

Here, M is the total number of edges, k_i, j_i are the degrees of the nodes at the ends of the $i'th(i = 1,2,...M)$ edge. $DCC < 0$ indicates the disassortativity of the network, and $DCC > 0$ indicates the assortativity, while $DCC = 0$ indicates no degree correlations.

The obtained statistical results for the co-expression networks are summarized in Tab.7. The average degrees and link densities of the co-expression networks are very low. The average degrees and link densities of the BnNAC and BnMYB subnetworks are higher than the other subnetworks, which indicate genes in the two TF families may have relatively stronger co-expression relationships. The co-expression network is assortative, indicating that highly co-expressed genes tend to connect with each other in the network.

Moreover, to evaluate the relative connection density within each TF family in comparison to those among families, we define the following index, called relative density (RD):

$$RD = \frac{N_{within}/N_{maximum\ within}}{N_{among}/N_{maximum\ among}}. \tag{7}$$

Here, $N_{maximum\ within}$ represents all possible pairs of edges within each of the five TF families. In this case, $N_{within} = 30+19+67+37+18 = 171, N_{maximum\ within} = 1/2(93\times92+42\times41+94\times93+48\times47+38\times37) = 11341$. $N_{among} = 542-171 = 371, N_{maximum\ among}$ denotes all possible pairs of connections among different families, here $N_{maximum\ among} = 1/2\times315\times314-N_{maximum\ within} = 38114$. Thus,

$$RD = \frac{171/11341}{371/38114} = 1.5490. \tag{8}$$

The RD reveals that the relative connection density among families is lower than that within families, and it indicates that TFs in the same family tends to exhibit similar expression profiles under various stresses or stimuli.

Furthermore, we declare that the degrees of the 315 DEGs in the gene co-expression network have no apparent correlation with their expression levels. To verify this declaration, we compute the Spearman correlation coefficient between degree sequence and the expression levels under the five treatments. Scatter plots for each treatments can be found in Fig.13. The Spearman correlation coefficients between degree sequence and the expression levels under the five treatments are all lower than 0.17.

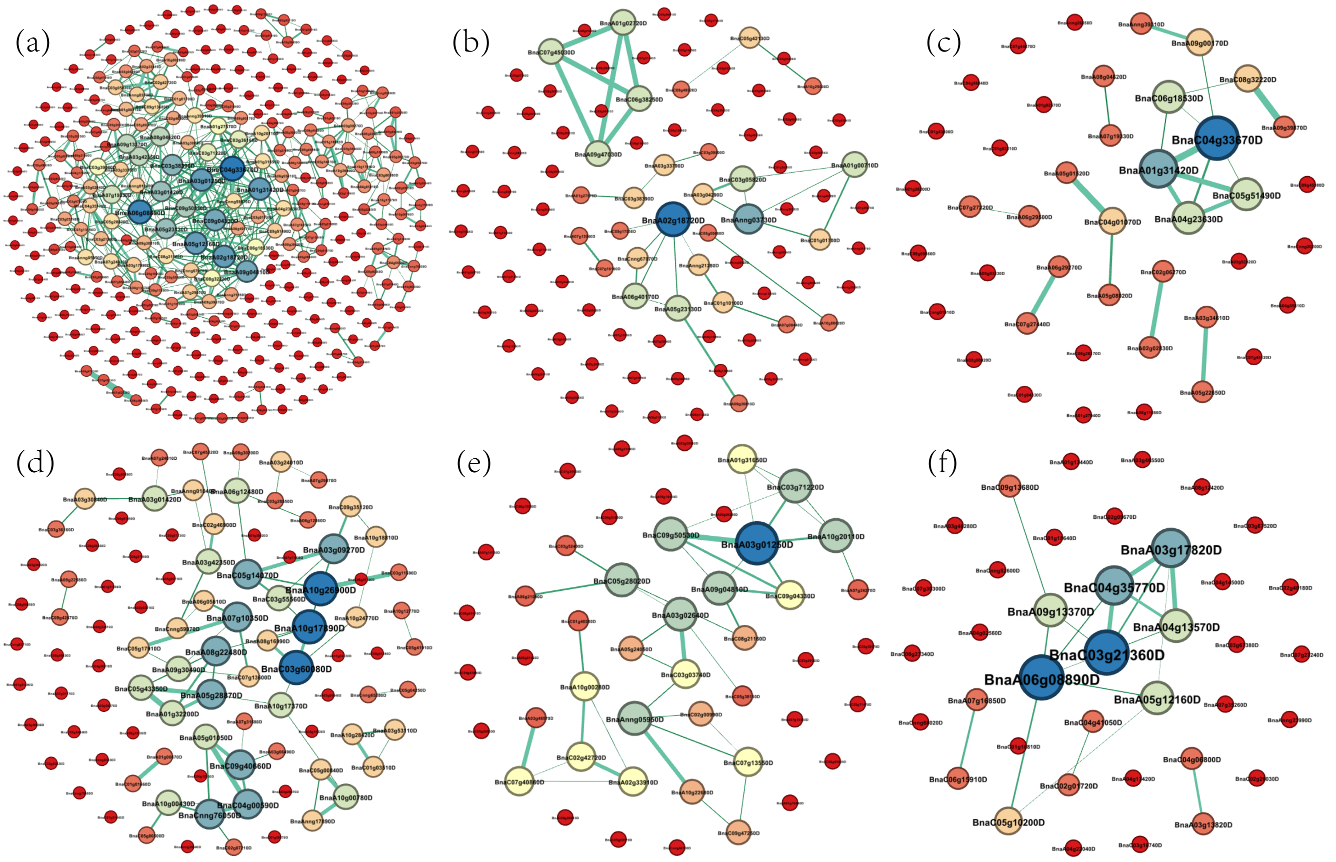

Figure 12. Gene co-expression network and its subnetworks of the five TF families. (a) The gene co-expression network for the 315 crucial DEGs; (b) Subnetwork for BnAP2/EREBPs; (c) Subnetwork for BnbZIPs; (d) Subnetwork for BnMYBs; (e) Subnetwork for BnNACs; (f) Subnetwork for BnWRKYs.

Table 7. **Statistics of the constructed gene co-expression network and its subnetworks.**

Networks	Node	Edges	Average degree	Link density	DCC
Gene co-expression network	315	542	3.4413	0.0110	0.5780
BnAP2/EREBP subnetwork	93	30	0.6452	0.0070	0.1398
BnbZIP subnetwork	42	19	0.9048	0.0221	0.6025
BnMYB subnetwork	94	67	1.4255	0.0153	0.3168
BnNAC subnetwork	48	37	1.5417	0.0328	0.0020
BnWRKY subnetwork	38	18	0.9474	0.0256	0.5135

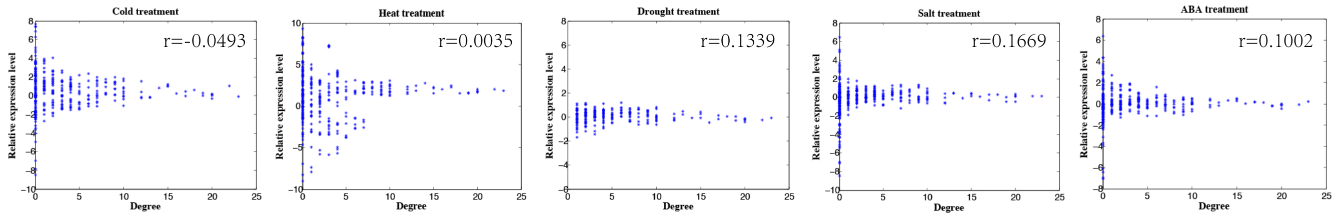

Figure 13. Scatter plots of node degree versus relative expression levels under cold, heat, drought, salt and ABA treatments for the gene co-expression network. *r* represents the corresponding Spearman correlation coefficient between degree sequence and the relative expression level.

Topological analysis on subnetworks of the constructed process-gene network

The constructed process-gene network and its subnetworks are shown in Fig.14. The obtained statistical indexes are summarized in Tab.8. From Fig.14 and Tab.8, we conclude that the process-gene network and its subnetworks are all densely connected. Especially, the link density of the BnMYB subnetwork equals 0.9, which indicates the 315 TFs involve in some closely related biological processes. The BnMYB subnetwork and the BnWRKY subnetwork are disassortative, which indicate highly connected genes in the process-gene network tend to connect with low degree ones. In the process-gene network, highly connected genes correspond to those genes who involve some popular biological processes, while low degree genes may involve some unpopular biological processes. Thus, the disassortativity of the BnMYB subnetwork and the BnWRKY subnetwork further indicate that some genes in the two families may simultaneously involve some popular and unpopular biological processes.

For the process-gene network, similar to the co-expression network, we can calculate the index *RD*. For the process-gene network, $N_{within} = 7652$, $N_{among} = 29466 - 7652 = 21814$. $N_{maximum\ within} = 11341, N_{maximum\ among} = 38114$. Therefore,

$$RD = \frac{7652/11341}{21814/38114} = 1.1789. \tag{9}$$

This also reveals that the relative connection density among families is lower than that within families, and it indicates genes in the same TF families prefer similar biological processes.

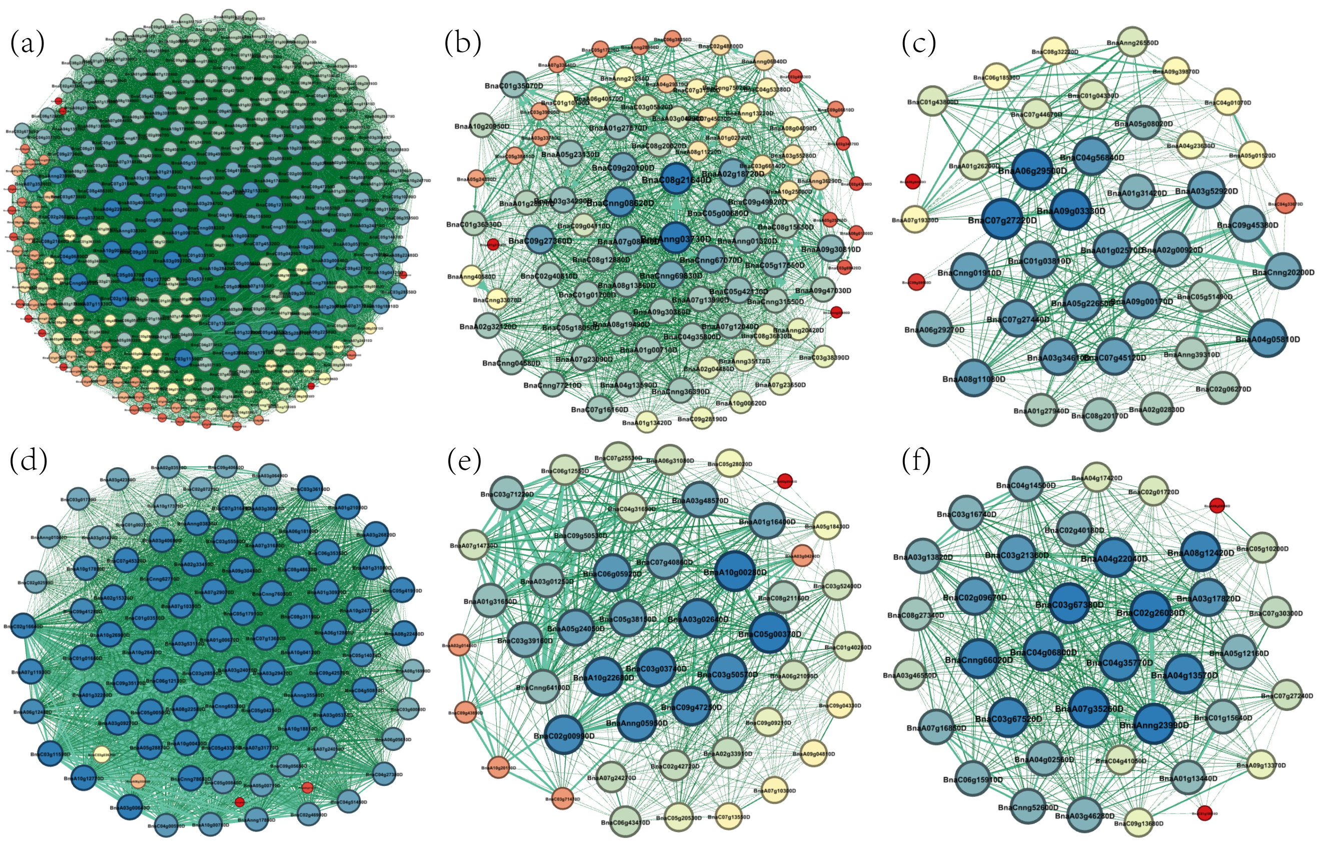

Figure 14. Process-gene network and its subnetworks of the five TF families. (a) The process-gene network for the 315 crucial DEGs; (b) Subnetwork for BnAP2/EREBPs; (c) Subnetwork for BnbZIPs; (d) Subnetwork for BnMYBs; (e) Subnetwork for BnNACs; (f) Subnetwork for BnWRKYs.

The scatter plots of unweighted node degrees versus relative expression levels under cold, heat, drought, salt and ABA treatments for the process-gene network are shown in Fig.15. Fig.15 also reveals that there are weak correlations between node degree and relative expression levels. DEGs with high relative expression levels are unnecessarily to involve many popular biological processes.

Table 8. **Statistics indexes of the constructed process-gene network and its subnetworks.**

Networks	Node	Undirected edges	Unweighted average degree	Unweighted link density	Unweighted DCC
Process-gene net.	315	29466	187.0857	0.5958	0.1194
BnAP2/EREBP subnet.	93	2050	44.0860	0.4792	0.2210
BnbZIP subnet.	42	421	20.0476	0.4890	0.2007
BnMYB subnet.	94	3934	83.7021	0.9000	-0.0602
BnNAC subnet.	48	693	28.8750	0.6144	0.0263
BnWRKY subnet.	38	554	29.1579	0.7881	-0.1006

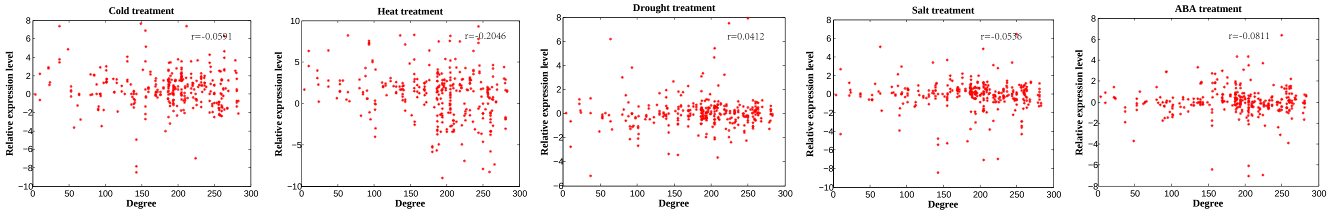

Figure 15. Scatter plots of unweighted node degrees versus relative expression levels under cold, heat, drought, salt and ABA treatments for the process-gene network. *r* represents the corresponding Spearman correlation coefficient between degree sequence and the relative expression level.

Regulatory network analysis on homologous genes in *A. thaliana* for the 315 DEGs

The identified 315 DEGs in *B. napus* have 181 *A. thaliana* homologous genes. Based on BioGrid (July 5, 2017), we constructed a gene network for *A. thaliana* with 9442 genes and 37741 directed edges. Among the 181 genes of *A. thaliana*, 133 members belong to the 9442 genes. The 133 genes can regulate or be regulated by another 657 genes (Not TFs). The constructed gene regulatory network can be found in Fig.7 of the main text. Among the 657 *A. thaliana* genes, 465 genes are homologous with 600 *B. napus* genes, and the 600 *B. napus* genes are expressed under the five treatments.

Hereinafter, we analyze the TF regulated genes or genes regulated by TFs. For simplicity, if one homologous gene in *B. napus* is differentially expressed under a treatment (log2 fold change is bigger than 1, and probability is no less than 0.6), then we call its counterpart gene in *A. thaliana* as a DEG. Statistical analysis shows that 128 of the 465 TF-related genes in *A. thaliana* (homologous with 219 of the 600 *B. napus* genes) are cold inducible, 208 genes (homologous with 373 *B. napus* genes) are heat responsive, 52 genes (homologous with 55 *B. napus* genes) are drought responsive, 51 genes (homologous with 111 *B. napus* genes) are salt responsive and 65 genes (homologous with 102 *B. napus* genes) are ABA inducible. The Venn diagrams for the DEGs in *B. napus* and *A. thaliana* are shown in Fig.16. The result reveals that up-stream or down-stream heat and cold responsive DEGs for the TFs take up the most. Moreover, three *B. napus* genes can differentially respond to five treatments, including BnaA04g19410D (AT2G33380), BnaC04g02990D (AT2G43510) and BnaA03g34110D (AT3G16240). Gene AT2G33380 has 6 homologous genes in *B. napus*, including BnaA04g19410D, BnaC04g43780D, BnaC04g11110D, BnaC03g18600D, BnaA05g10200D and BnaA03g15390D. Gene AT2G33380 is reported to be salt and drought responsiveness[33]. AT3G16240 has 7 homologous genes in *B. napus*, including BnaA03g34110D, BnaA05g23460D, BnaA01g28120D, BnaC06g05270D, BnaC01g44580D, BnaC03g39560D and BnaC05g37160D. AT2G33380 can regulate an AtbZIP gene AT2G36270 and an AtMYB gene AT5G37260. AT2G43510 and AT3G16240 both can regulate the AtNAC gene AT5G22290.

To thoroughly explore the regulation patterns of the identified TFs, we summarized the numbers and proportions of unique genes (Their homologous genes can respond to the five treatments and are DEGs in *B. napus*) that regulate or be regulated by the 133 TFs in *A. thaliana*, as shown in Tab.9. Statistical results reveal that the AtAP2/EREBPs, AtbZIPs, AtMYBs, AtNACs and AtWRKYs can regulate 22, 38, 42, 24, and 11 down-stream genes (Their homologous genes in *B. napus* are DEGs) respectively. For the five TF family, about 45%-81% down-stream genes are heat inducible. About 50% down-stream genes of AtbZIPs are cold responsive. The down-stream genes of AtAP2/EREBPs are short of drought responsive ones, however, 2 of the 11 down-stream genes of AtWRKY are drought responsive ones, and 6 of the 42 down-stream genes of AtMYBs are drought responsive ones. 3 of the 11 down-stream genes of AtWRKY and 9 of the 38 down-stream genes of AtbZIPs are salt inducible. The down-stream genes regulated by AtAP2/EREBPs have the highest fractions of ABA responsive ones (40.91%). Moreover, among the TF regulated genes, the AtAP2/EREBP regulated gene AT2G44040 has been reported to involve the response to abiotic stresses[34], and we found its homologous genes in *B. napus* are ABA, heat and salt responsiveness. It is also reported that the AtbZIP regulated gene AT3G11410 can negatively regulate ABA signaling and up-regulated by drought

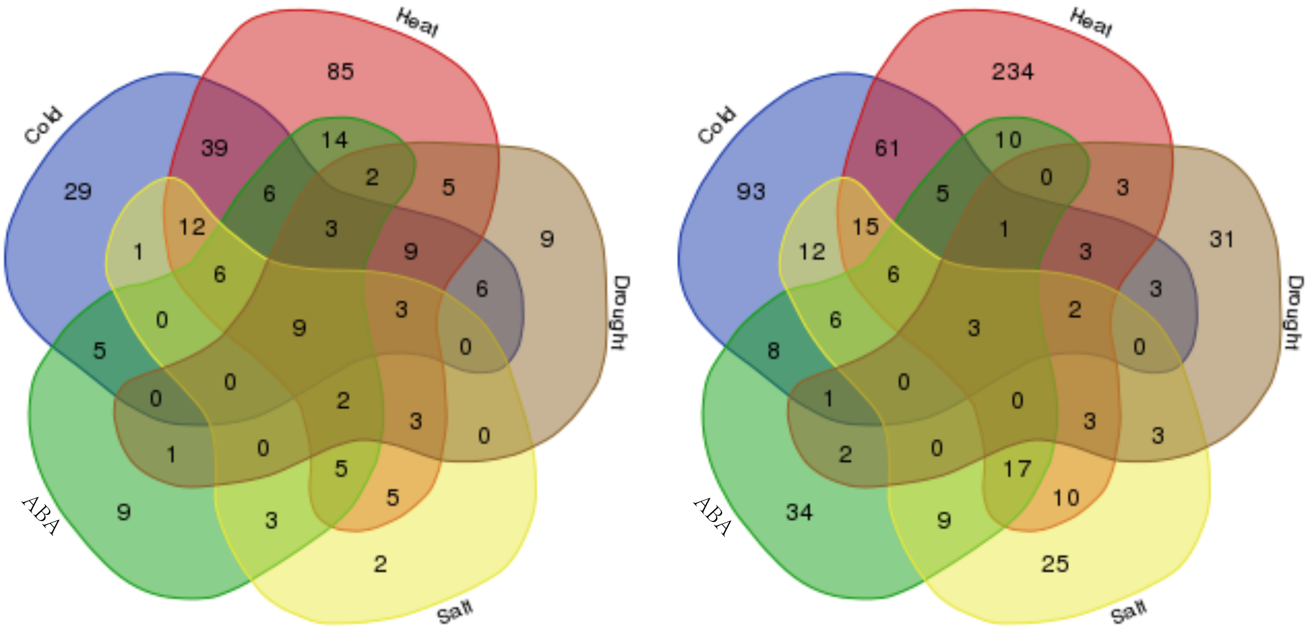

Figure 16. Venn diagrams for DEGs that regulate or be regulated by the 315 crucial TFs. (a) The 465 homologous genes in *A. thaliana*. (b) The 600 stresses/stimuli inducible genes in *B. napus*.

and ABA[1,35,36], and we found its homologous genes in *B. napus* are ABA, cold, heat and salt responsiveness. Therefore, we speculate that the identified TFs can actually trigger the differentially expression of their down-stream genes.

On the other hand, we find 25, 36, 33, 121 and 13 up-stream genes that can regulate TFs of the five families respectively, and their homologous genes in *B. napus* are differentially expressed under at least one of the five treatments. There are hundreds of up-stream genes for AtNACs. Most of the up-stream genes are heat responsiveness. Except AtWRKYs, about 50% up-stream genes for each TF families are cold responsiveness. About 25% up-stream genes of AtAP2/EREBPs and AtbZIPs are salt responsiveness, and more than 30% up-stream AtAP2/EREBPs, AtMYBs and AtWRKYs are ABA responsiveness. However, only one up-stream gene for AtWRKYs is cold responsiveness.

Table 9. The numbers and proportions of unique down-stream and up-stream genes (Their homologous genes can differentially respond to the five treatments in *B. napus*) of the 133 TFs in *A. thaliana*. The total number of up-stream and down-stream DEGs is 465, which are not members of the five TF families.

TF families	The number of unique down-stream DEGs	Cold		Heat		Drought		Salt		ABA	
		Num.	Prop.	Num.	Prop.	Num.	Prop.	Num.	Prop.	Num.	Prop.
AtAP2/EREBPs	22	10	0.4545	16	**0.7273**	1	0.0455	3	0.1364	9	**0.4091**
AtbZIPs	38	19	**0.5000**	29	0.7632	5	0.1316	9	0.2368	9	0.2368
AtMYBs	42	19	0.4524	34	**0.8095**	6	0.1429	7	0.1667	13	0.3095
AtNACs	24	11	0.4583	18	**0.7500**	3	0.1250	2	0.0833	6	0.2500
AtWRKYs	11	2	0.1818	5	**0.4546**	2	**0.1818**	3	**0.2727**	4	0.3636
TF families	The number of unique up-stream DEGs	Cold		Heat		Drought		Salt		ABA	
		Num.	Prop.	Num.	Prop.	Num.	Prop.	Num.	Prop.	Num.	Prop.
AtAP2/EREBPs	25	13	0.5200	18	**0.7200**	6	**0.2400**	6	**0.2400**	9	**0.3600**
AtbZIPs	36	18	0.5000	31	**0.8611**	5	0.1389	9	**0.2500**	9	0.2500
AtMYBs	33	18	**0.5455**	27	**0.8182**	7	**0.2121**	6	0.1818	12	**0.3636**
AtNACs	121	60	0.4959	94	**0.7769**	26	**0.2149**	22	0.1818	25	0.2066
AtWRKYs	13	1	0.0769	13	**1.0000**	2	0.1539	3	0.2308	4	**0.3077**

The above results indicate the identified TFs can trigger the differentially expression of tens of down-stream genes, and they can also be regulated by tens to hundreds of up-stream genes. To see whether the response of a gene could possibly be triggered by up-stream genes and whether the gene can trigger differentially expression of down-stream genes, we take two subnetworks for AtWRKYs as concrete examples, which are shown in Fig.17 (a). The first subnetwork consists of three AtWRKY genes

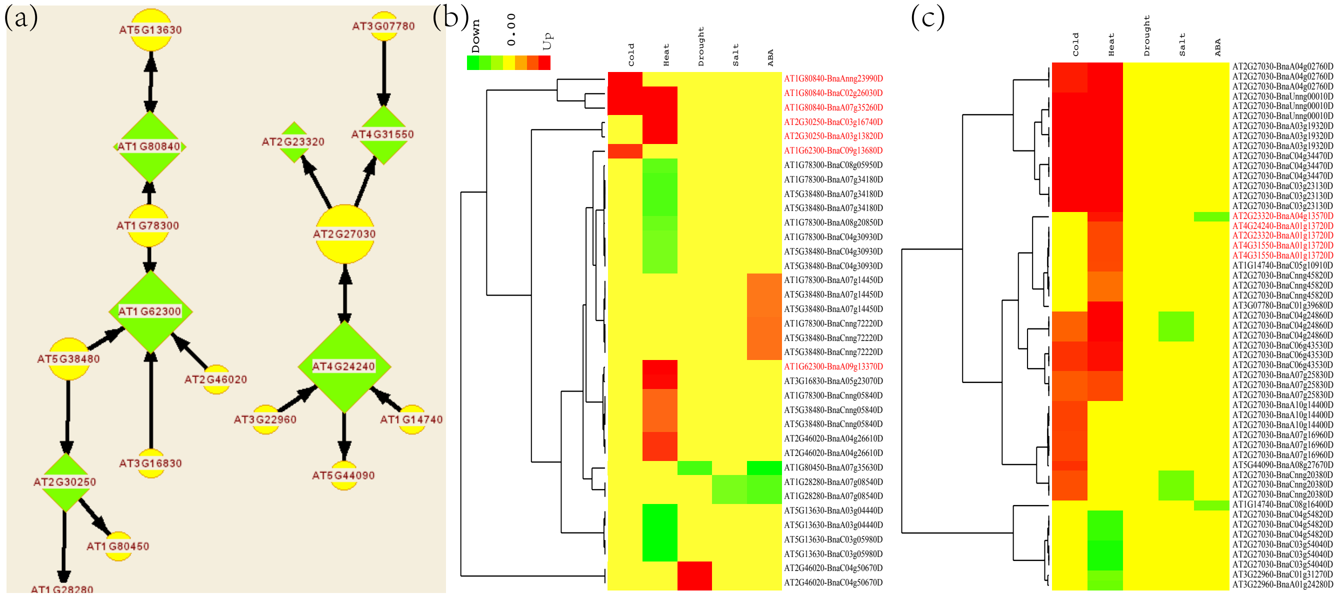

Figure 17. Concrete examples for two AtWRKY subnetworks. (a) The two AtWRKY subnetworks. Diamond nodes represent AtWRKYs, and nodes shown in circle represent up-stream or down-stream genes of AtWRKYs. (b) Expression profiles of homologous genes in *B. napus* for the first subnetwork. (c) Expression profiles of homologous genes in *B. napus* for the second subnetwork. Yellow blocks denote the corresponding gene is not a DEG under the related treatments.

AT1G80840, AT2G30250 and AT1G62300, and the second subnetwork consists of another three AtWRKY genes, including AT4G24240, AT2G23320 and AT4G31550.

In the first subnetwork, the homologous genes of the AtWRKY gene AT1G80840 are cold and heat responsive in *B. napus*, and these homologous genes are up-regulated under the two stresses. The homologous genes of AT2G30250 is heat inducible, and the homologous genes of AT1G62300 are cold and heat responsive. AT1G80840 can mutually regulate gene AT5G13630 and regulated by gene AT1G78300. The homologous genes for AT5G13630 all can respond to heat stress and down-regulated. Five of the seven homologous genes for the up-stream gene AT1G78300 are heat responsive, and two of the seven homologous genes are ABA inducible. Therefore, we speculate that the stress responsiveness of AT1G80840 is closely related to its up-stream and down-stream genes. AT1G78300 can regulate another AtWRKY gene AT1G62300, while one homologous gene of AT1G62300 can respond to cold and one can respond to heat stress. Homologous genes of another three up-stream genes for AT1G62300 are all heat responsiveness, and homologous gene of AT2G46020 in *B. napus* can further respond to drought stress, and AT5G38480 can further respond to ABA stimulus. Thus, we speculate that the cold and heat responsiveness of AT1G62300 is possible regulated by its up-stream genes. The third AtWRKY gene AT2G30250 can regulate two genes, and homologous genes for one of the two genes can respond to salt, ABA, and another one can respond to ABA and drought. Since drought and salt stresses can be induced by heat stress, thus, we speculate that the heat responsive AtWRKY AT2G30250 is responsible for the stress responsiveness of its two down-stream genes.

Similarly, in the second subnetwork, homologous genes of the three AtWRKY genes AT4G24240, AT2G23320 and AT4G31550 can respond to heat stress, and homologous genes of AT2G23320 can also respond to ABA stimulus. By analyzing the expression profiles of up-stream or down stream genes for the three AtWRKYs, we can also declare that the stress responsiveness of the three AtWRKYs correlate with their up-stream genes, and the three AtWRKYs are responsible for the stress responsiveness of their down-stream genes.

Common component analysis of the constructed networks

Between the constructed gene co-expression network and the process-gene network, we find 182 of the 315 DEGs have common connections in the two networks. The 315 DEGs in *B. napus* are homologous with 181 *A. thaliana* genes, and based on BioGrid (July 5, 2017), we constructed a gene network for *A. thaliana* with 9442 genes and 37741 directed edges. Among the 181 genes of *A. thaliana*, 133 members belong to the 9442 genes. The 133 *A. thaliana* genes have 254 homologous of the 315 crucial DEGs. Between the 182 and the 254 *B. napus* genes, 153 genes of *B. napus* are common ones. The 153 common genes are further analyzed in great detail. The 153 common genes have 86 homologous in *A. thaliana*, which are shown in Tab.9. Each gene of *A. thaliana* has one to six homologous in *B. napus*. The 86 genes of *A. thaliana* include 16 AtAP2/EREBPs, 16

AtbZIPs, 27 AtMYBs, 14 AtNACs and 13 AtWRKYs. Among the 153 genes of *B. napus*, 61 genes are cold responsive, 127 genes are heat responsive, 4 genes are drought responsive, 10 genes are salt responsive and 19 genes are ABA responsive. The heat responsive and cold responsive DEGs take up the most in comparison to other three treatments (Fig.18 and Tab.10).

Table 10. Detailed information of the 86 genes in *A. thaliana* that are homologous with the 153 DEGs in *B. napus*.

Gene	Family	Group	Response	Node degree in Fig. 9 (c)	Homologous genes in *B. napus*
AT3G19290	AtbZIP	V	ABA	21	BnaA01g26200D;BnaC01g43800D
AT4G34590	AtbZIP	X	ABA	11	BnaA01g02570D;BnaA03g52920D;BnaA08g11080D;BnaC01g03810D;BnaC07g45120D
AT1G18570	AtMYB	R2R3	ABA	50	BnaA08g22580D
AT5G62470	AtMYB	R2R3	ABA	2	BnaA02g33410D;BnaC09g05650D;BnaCnng62710D
AT5G28770	AtbZIP	VII	Cold	17	BnaA06g29270D;BnaC07g27440D
AT2G16720	AtMYB	R2R3	Cold	6	BnaCnng76050D
AT3G06490	AtMYB	1R	Cold	1	BnaA03g29470D
AT3G16350	AtMYB	1R	Cold	1	BnaAnng35540D
AT4G37260	AtMYB	R2R3	Cold	4	BnaA01g00670D;BnaC01g01660D
AT4G39250	AtMYB	1R	Cold	4	BnaC01g00270D
AT5G08510	AtMYB	R2R3	Cold	1	BnaC03g03620D
AT1G52880	AtNAC	VI	Cold	12	BnaCnng64100D
AT1G69490	AtNAC	VI	Cold	3	BnaA07g24270D;BnaC06g43410D
AT4G34990	AtMYB	R2R3	Cold;ABA	6	BnaA03g53110D;BnaC01g03510D;BnaC07g45320D
AT3G56400	AtWRKY	III	Cold;drought	2	BnaA04g02560D;BnaA07g16850D;BnaC06g15910D;BnaC08g27340D;BnaCnng52600D
AT2G28550	AtAP2/EREBP	AP2	Cold;heat	34	BnaA07g13990D
AT3G11020	AtAP2/EREBP	DREBA2	Cold;heat	2	BnaA01g31290D;BnaAnng23490D;BnaC05g42130D
AT3G15210	AtAP2/EREBP	ERFB1	Cold;heat	7	BnaA03g33790D;BnaC03g39000D;BnaC05g38410D
AT5G13330	AtAP2/EREBP	ERFB4	Cold;heat	2	BnaA03g04290D;BnaC03g05820D
AT3G17609	AtbZIP	III	Cold;heat	7	BnaA01g27940D;BnaA03g34610D;BnaA05g22650D
AT5G49450	AtbZIP	VIII	Cold;heat	14	BnaA06g29500D;BnaA09g03330D;BnaC07g27220D
AT1G01060	AtMYB	1R	Cold;heat	17	BnaA10g00780D;BnaAnng17890D;BnaC05g00840D
AT1G18330	AtMYB	1R	Cold;heat	6	BnaA06g12480D;BnaC05g14070D
AT2G46830	AtMYB	1R	Cold;heat	24	BnaA05g01050D;BnaC04g00590D
AT3G09600	AtMYB	1R	Cold;heat	2	BnaA01g32200D;BnaA03g00640D;BnaA05g28870D;BnaC05g43350D
AT5G17300	AtMYB	1R	Cold;heat	2	BnaA02g03510D;BnaA03g06490D;BnaA10g17370D;BnaC02g07210D;BnaC09g40660D
AT1G32870	AtNAC	VIII	Cold;heat	1	BnaA07g10300D;BnaC07g13550D
AT5G13180	AtNAC	VI	Cold;heat	14	BnaA02g01460D;BnaA03g04240D;BnaA10g20110D;BnaC03g71470D;BnaC09g43890D
AT1G62300	AtWRKY	IIb	Cold;heat	4	BnaA09g13370D;BnaC09g13680D
AT1G80840	AtWRKY	IIb	Cold;heat	11	BnaA07g35260D;BnaAnng23990D;BnaC02g26030D
AT2G24570	AtWRKY	IId	Cold;heat	8	BnaC03g67520D;BnaA08g12420D
AT2G30250	AtWRKY	I	Cold;heat	19	BnaA03g13820D;BnaA04g17420D;BnaA05g12160D;BnaC03g16740D;BnaC04g14500D;BnaC04g41050D
AT2G38470	AtWRKY	I	Cold;heat	26	BnaA03g17820D;BnaA04g22040D;BnaC03g21360D;BnaC04g06800D;BnaCnng66020D
AT4G24240	AtWRKY	IId	Cold;heat	7	BnaA03g46550D;BnaA08g12420D;BnaC03g67520D
AT4G31550	AtWRKY	IId	Cold;heat	3	BnaA08g12420D;BnaC03g67520D
AT1G01520	AtMYB	1R	Cold;heat;ABA	1	BnaA10g00430D;BnaC05g00500D;BnaA03g00640D
AT1G22640	AtMYB	R2R3	Cold;heat;ABA	3	BnaA07g10350D;BnaA09g30490D;BnaC05g17910D;BnaC07g13600D
AT2G23320	AtWRKY	IId	Cold;heat;ABA	1	BnaA04g13570D;BnaC04g35770D;BnaC03g67520D
AT5G08790	AtNAC	VI	Cold;heat;drought	3	BnaA03g02640D;BnaA10g22680D;BnaC09g47250D;BnaAnng05950D;BnaC02g00990D;BnaC03g03740D
AT1G01720	AtNAC	VI	Cold;Heat;drought;salt	6	BnaA10g00280D;BnaC05g00370D;BnaAnng05950D;BnaC03g50570D
AT5G63790	AtNAC	VI	Cold;heat;drought;salt	3	BnaA02g33910D;BnaAnng05950D;BnaC02g00990D;BnaC02g42720D;BnaC03g03740D;BnaC03g50570D
AT4G27410	AtNAC	VI	Cold;heat;salt;ABA	8	BnaA01g16400D;BnaA03g48570D;BnaC07g40860D
AT1G03800	AtAP2/EREBP	ERFB1	Heat	3	BnaA07g08440D;BnaCnng67070D;BnaA01g28970D
AT1G22190	AtAP2/EREBP	DREBA6	Heat	17	BnaA09g30810D;BnaC05g17550D
AT1G78080	AtAP2/EREBP	DREBA6	Heat	7	BnaA02g18720D
AT1G80580	AtAP2/EREBP	ERFB1	Heat	1	BnaA07g08440D;BnaCnng67070D
AT2G40350	AtAP2/EREBP	DREBA2	Heat	1	BnaA10g25000D;BnaC09g49920D
AT3G16770	AtAP2/EREBP	ERFB2	Heat	8	BnaA01g27570D;BnaA03g34290D;BnaA05g23130D;BnaC01g35070D
AT3G23230	AtAP2/EREBP	ERFB3	Heat	3	BnaAnng06940D;BnaC01g10100D;BnaAnng21280D
AT4G17500	AtAP2/EREBP	ERFB3	Heat	1	BnaC01g10100D
AT4G34410	AtAP2/EREBP	ERFB3	Heat	1	BnaA01g02720D;BnaA08g11220D;BnaC03g66140D;BnaC07g45030D
AT5G44210	AtAP2/EREBP	ERFB1	Heat	5	BnaA01g28970D;BnaC01g36330D;BnaA07g08440D;BnaCnng67070D
AT5G47230	AtAP2/EREBP	ERFB3	Heat	2	BnaA06g40170D;BnaC09g20100D
AT1G43700	AtbZIP	III	Heat	13	BnaAnng39310D;BnaC05g51490D
AT2G21230	AtbZIP	III	Heat	6	BnaC04g33670D
AT2G36270	AtbZIP	V	Heat	73	BnaA05g08020D
AT3G10800	AtbZIP	III	Heat	15	BnaA01g31420D
AT3G51960	AtbZIP	I	Heat	8	BnaA04g05810D
AT3G62420	AtbZIP	X	Heat	15	BnaA07g19330D;BnaA09g39870D;BnaC06g18530D;BnaC08g32220D
AT5G11260	AtbZIP	III	Heat	56	BnaA02g00920D;BnaC09g45380D;BnaCnng20200D
AT5G15830	AtbZIP	XII	Heat	2	BnaA02g02830D;BnaC02g06270D
AT1G09710	AtMYB	1R	Heat	2	BnaA06g05610D
AT1G70000	AtMYB	1R	Heat	1	BnaA07g24010D
AT3G16857	AtMYB	1R	Heat	21	BnaA03g42350D
AT4G09460	AtMYB	R2R3	Heat	3	BnaA03g24010D;BnaC03g28550D
AT5G02840	AtMYB	1R	Heat	2	BnaA10g26900D;BnaA03g00640D
AT5G04760	AtMYB	R2R3	Heat	3	BnaA03g01420D;BnaAnng01040D;BnaC03g01730D;BnaC02g02590D
AT5G05790	AtMYB	R2R3	Heat	4	BnaA10g24770D
AT5G07690	AtMYB	1R	Heat	4	BnaCnng65380D
AT5G16600	AtMYB	R2R3	Heat	1	BnaA10g17890D;BnaC09g41280D
AT3G04060	AtNAC	IX	Heat	3	BnaC01g40260D
AT3G15500	AtNAC	VI	Heat	14	BnaA05g24050D;BnaC03g39160D;BnaC05g38150D
AT3G49530	AtNAC	VII	Heat	1	BnaA06g21090D;BnaA09g04810D;BnaC03g52400D;BnaC08g21160D;BnaC09g04330D
AT5G04410	AtNAC	VIII	Heat	1	BnaC03g71220D;BnaC09g50530D
AT5G24590	AtNAC	VII	Heat	1	BnaA06g21090D;BnaA09g04810D;BnaC03g52400D;BnaC08g21160D;BnaC09g04330D
AT1G13960	AtWRKY	I	Heat	1	BnaA06g08890D;BnaC05g10200D
AT2G03340	AtWRKY	I	Heat	1	BnaC05g10200D
AT5G07100	AtWRKY	I	Heat	1	BnaC02g01720D
AT5G26170	AtWRKY	IIc	Heat	1	BnaC02g40180D
AT1G71030	AtMYB	1R	Heat;ABA	8	BnaA02g15320D;BnaA07g29070D
AT4G38620	AtMYB	R2R3	Heat;ABA	6	BnaA08g16990D;BnaC03g60080D
AT5G39610	AtNAC	IX	Heat;ABA	3	BnaA07g14730D;BnaC04g31690D;BnaC06g12550D
AT4G01120	AtbZIP	VII	Heat;drought	3	BnaA09g00170D;BnaCnng01910D
AT4G23750	AtAP2/EREBP	ERFB5	Heat;salt	20	BnaA01g13420D
AT2G46270	AtbZIP	VII	Heat;salt;ABA	4	BnaA05g01520D;BnaC04g01070D
AT4G34000	AtbZIP	V	Heat;salt;ABA	25	BnaAnng26550D;BnaC01g04330D;BnaC07g44670D

Crucial novel members and their responsiveness to the five treatments

Among the identified 315 crucial TFs, two BnMYB genes BnaA03g42350D and BnaA08g30200D are novel ones, which have not been identified in previous works. However, we find BnaA03g42350D is a crucial heat responsive gene, with relative expression level 1.5567. This gene involves ethylene-activated signaling pathway, regulation of root meristem growth, regulation

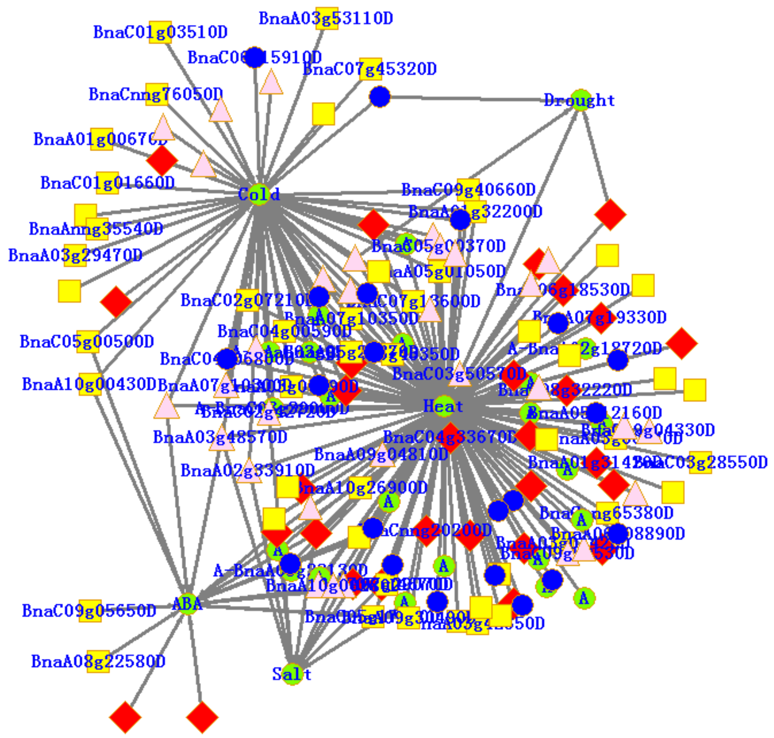

Figure 18. Stresses/stimuli responsiveness of the 153 common DEGs.

of stomatal movement, leaf senescence, regulation of seed growth and so on. Gene BnaA03g42350D contains ABRE, HSE, LTR, MBS and TC rich repeats, indicating that it may involve the response to ABA, heat, cold, drought, defense and stress responsive. BnaA08g30200D is a crucial cold responsive gene, with relative expression level -1.2474. This gene involves positive regulation of cell proliferation, cold acclimation, response to auxin, response to hydrogen peroxide and so on. Gene BnaA08g30200D contains five cis-acting elements, including ABRE, GARE motif, P-box, HSE and MBS, which further indicates that such gene may involve the response to ABA, GA, heat and drought stresses.

Gene BnaA08g30200D is homologous with the AtMYB gene AT4G16420 in *A. thaliana*. AT4G16420 is the component of SAGA complex and is reported to control gene expression in response to abiotic stresses[37]. Gene AT4G16420 can regulate the expression of five other genes (AT3G54610, AT4G25490, AT4G26840, AT5G25810, AT5G55170) in *A. thaliana* and be regulated by another three genes (AT4G25490, AT1G68185, AT3G54610). The regulated gene AT4G26840 is homologous with 11 genes in *B. napus* (BnaA01g15940D, BnaA02g09210D, BnaA03g48300D, BnaA08g14280D, BnaA10g09410D, BnaC01g19050D, BnaC02g13260D, BnaC06g21370D, BnaC07g40510D, BnaC08g12330D, BnaC09g31750D). Gene BnaA08g14280D can respond to heat and ABA. BnaC02g13260D can respond to heat. BnaC07g40510D can respond to salt and ABA.

Moreover, among the identified 315 TFs, 51 BnAP2/EREBPs are collected by PlantTFDB but not investigated in the recent work by Du et al.[9]. Further analysis also reveal the responsiveness of the 51 BnAP2/EREBPs to the five treatments. Therefore, we declare that our work has identified some novel members for the TF families and these novel members may actually play roles in stress responsiveness.

Additional Files

Additional file 1 —Supplementary materials that supporting the main text. (PDF, 62.8M)

Additional file 2 —The identified five families of TFs in the four species. (XLSX, 143KB)

Additional file 3 —Final statistics for synteny analsyis. (XLSX, 141KB)

Additional file 4 —Responsive genes of the five TF families. (XLSX, 517KB)

Additional file 5 —The finally identified 315 crucial DEGs. (XLSX, 190KB)

Additional file 6 —Detailed information for the 153 common DEGs. (XLSX,133KB)

Abbreviations

TF: transcription factor; *B. napus: Brassica napus;* B. rapa: Brassica rapa, *B. oleracea: Brassica oleracea*; *A. thaliana: Arabidopsis thaliana*; CT: C-terminal; NT: N-terminal; ABA: abscisic acid; GA: Gibberellin; JA: jasmonic acid; SA: salicylic acid; MeJA: Methyl Jasmonate; ESTs: expressed sequence tags; DEG: Differentially expressed genes; FPKM: Fragments per kilobase of exon model per million mapped reads; GO: Gene Ontology; RNA-seq: RNA-sequencing; qRT-PCR: quantitative real time polymerase chain reaction; FDR: FDR: False discovery rate.

Ethics approval and consent to participate

Not applicable.

Consent to publish

Not applicable.

Availability of data and materials

The Sequence data are deposited at the NCBI under the accessions SRP109808. All data analysed during this study are included in this published article and its supplementary information files.

Competing interests

The authors declare that they have no competing interests.

Funding

This work was supported by the National Key Research and Development Program of China (2016YFD0101900) and the National Natural Science Foundation of China (61773153 and 31671728). The Key Scientific Research Projects in Colleges and Universities of Henan under Grants 17A120002. Major science and technology special projects of Henan Province (151100111200) and Henan science and technology research project (172102110005). The Basal Research Fund of Henan University (yqpy20140049).

Author's contributions

DW, CS and XZ conceived the experiment. All authors conducted the experiments. PW, ZH and XL performed bioinformatics analysis and analyzed the results. PW, DW and ZH wrote the manuscript. All authors reviewed the manuscript.

Acknowledgements

Not applicable.

References

1. Huala E, Dickerman AW, Garcia-Hernandez M, et al. The Arabidopsis Information Resource (TAIR): a comprehensive database and web-based information retrieval, analysis, and visualization system for a model plant. Nucleic Acids Res. 2001; 29: 102-5.
2. Cheng F, Liu S, Wu J, et al. BRAD, the genetics and genomics database for *Brassica* plants. BMC Plant Biol. 2011; 11: 136.
3. Kersey PJ, Allen JE, Armean I, et al. Ensembl Genomes 2016: more genomes, more complexity.Nucleic Acids Res. 2016; 44: D574-80.
4. Chalhoub B, Denoeud F, Liu S, et al. Early allopolyploid evolution in the post-Neolithic *Brassica napus* oilseed genome. Science. 2014; 345: 950-3.
5. Finn RD, Coggill P, Eberhardt RY, et al. The Pfam protein families database: towards a more sustainable future. Nucleic Acids Res. 2016; 44: D279-85.
6. Schultz J, Milpetz F, Bork P, et al. SMART, a simple modular architecture research tool: Identification of signaling domains. Proc Natl Acad Sci USA. 1998; 95: 5857-64.

7. Bailey TL, Elkan C. Fitting a mixture model by expectation maximization to discover motifs in biopolymers. Proc Int Conf Intell Syst Mol Biol. 1994; 2: 28-36.
8. Mount WD. Bioinformatics: sequence and genome analysis. New York: Cold Spring Harbor Laboratory Press; 2004.
9. Du C, Hu K, Xian S, et al. Dynamic transcriptome analysis reveals AP2/ERF transcription factors responsible for cold stress in rapeseed (*Brassica napus* L.). Mol Genet Genomics. 2016; 291: 1053-67.
10. Zhou Y, Xu D, Jia L, et al. Genome-wide identification and structural analysis of bZIP transcription factor genes in *Brassica napus*. Genes. 2017; 8: 288.
11. Hajiebrahimi A, Owji H, Hemmati S. Genome-wide identification, functional prediction and evolutionary analysis of the R2R3-MYB superfamily in *Brassica napus*. Genome. 2017; 60: 797-814.
12. Wang B, Guo X, Wang C, et al. Identification and characterization of plant-specific NAC gene family in canola (*Brassica napus* L.) reveal novel members involved in cell death. Plant Mol Biol. 2015; 87: 395-411.
13. Wu J, Zhao Q, Yang Q, et al. Comparative transcriptomic analysis uncovers the complex genetic network for resistance to Sclerotinia sclerotiorum in *Brassica napus*. Sci Rep. 2016; 6: 19007.
14. He Y, Mao S, Gao Y, et al. Genome-wide identification and expression analysis of WRKY transcription factors under multiple stresses in *Brassica napus*. PLoS One. 2016; 11: e0157558.
15. Zhuang J, Zhu B. Analysis of *Brassica napus*, ESTs: gene discovery and expression patterns of AP2/ERF-family transcription factors. Mol Biol Rep. 2014; 41: 45–56.
16. Song X, Wang J, Ma X, et al. Origination, expansion, evolutionary trajectory, and expression bias of AP2/ERF superfamily in *Brassica napus*. Front Plant Sci. 2016; 7: 1186.
17. Hwang I, Jung HJ, Park JI, et al. Transcriptome analysis of newly classified bZIP transcription factors of *Brassica rapa*, in cold stress response. Genomics. 2014; 104: 194-202.
18. Bai Y, Zhu W, Hu X, et al. Genome-wide analysis of the bZIP gene family identifies two ABI5-like bZIP transcription factors, BrABI5a and BrABI5b, as positive modulators of ABA signalling in *Chinese Cabbage*. PLos One. 2016; 11: e0158966.
19. Hwang I, Manoharan RK, Kang JG, et al. Genome-wide identification and characterization of bZIP transcription factors in *Brassica oleracea* under cold stress. Biomed Res Int. 2016; 2016: 1-18.
20. Jin JP, Tian F, Yang DC, et al. PlantTFDB 4.0: toward a central hub for transcription factors and regulatory interactions in plants. Nucleic Acids Res. 2017; 45: D1040-45.
21. Rinerson CI, Rabara RC, Tripathi P, et al. The evolution of WRKY transcription factors. BMC Plant Biol. 2015; 15: 66.
22. Liu C, Zhang X, Zhang K, et al. Comparative analysis of the *Brassica napus* root and leaf transcript profiling in response to drought stress. Int J Mol Sci. 2015; 16: 18752-77.
23. Rushton PJ, Somssich IE, Ringler P, et al. WRKY transcription factors. Trends in Plant Sci. 2010; 15: 247-58.
24. Xie Z, Zhang ZL, Zou X, et al. Annotations and functional analyses of the rice WRKY gene superfamily reveal positive and negative regulators of abscisic acid signaling in aleurone cells. Plant Physiol. 2005; 137: 176-89.
25. Snedecor GW, Cochran WG. Statistical Methods. Iowa State University Press;1989.
26. Larkin MA, Blackshields G, Brown NP, et al. Clustal W and Clustal X version 2.0. Bioinformatics. 2007; 23: 2947-8.
27. Bakshi M, Oelm´uller R. WRKY transcription factors: Jack of many trades in plants. Plant Signal Behav. 2014; 9: e27700.
28. Lyons E, Freeling M. How to usefully compare homologous plant genes and chromosomes as DNA sequences. Plant J. 2008; 53: 661–73.
29. Jin X, Ren J, Nevo E, et al. Divergent evolutionary patterns of NAC transcription factors are associated with diversification and gene duplications in angiosperm. Front Plant Sci. 2017; 8: 1156.
30. Vinayagam A, Gibson TE, Lee HJ, et al. Controllability analysis of the directed human protein interaction network identifies disease genes and drug targets. Proc Natl Acad Sci USA. 2016; 113: 4976-81.
31. Wang P, Yu X, Lü, J. Identification and evolution of structurally dominant nodes in protein-protein interaction networks. IEEE Trans Biomed Circuits Syst. 2014; 8: 87-97.
32. Newman MEJ. The structure and function of complex networks. *SIAM Rev,* 2003; 45(2): 167-256.

33. Weston DJ, Gunter LE, Rogers A, et al. Connecting genes, coexpression modules, and molecular signatures to environmental stress phenotypes in plants. BMC Syst Biol. 2008; 2:16.

34. Less H, Galili G. Principal transcriptional programs regulating plant amino acid metabolism in response to abiotic stresses. Plant Physiol. 2008; 147: 316-30.

35. Zhang Y, Li Q, Jiang L,et al. Suppressing type 2C protein phosphatases alters fruit ripening and the stress response in *Tomato. Plant Cell Physiol. 2018; 59: 142-54.*

36. *Nee G, Kramer K, Nakabayashi K, et al. Delay of GERMINATION1 requires PP2C phosphatases of the ABA signalling pathway to control seed dormancy. Nat Commun. 2017; 8: 72.*

37. *Moraga F, Aquea F. Composition of the SAGA complex in plants and its role in controlling gene expression in response to abiotic stresses. Front Plant Sci. 2015; 6: 865.*
