# Supplementary material for: Exploring transcription factors reveals crucial members and regulatory networks involved in different abiotic stresses in Brassica napus L
Source: BMC Plant Biol. 2018 Sep 19;18:202. doi: 10.1186/s12870-018-1417-z (PMC6146658; doi:10.1186/s12870-018-1417-z)
Supplement: Supplementary file 2 — The identified five families of TFs in the four species. (PDF 216 kb) [file 12870_2018_1417_MOESM2_ESM.pdf]

Additional file 2: The identified TFs in B.napus,B. rapa, B.olercea and A. thaliana

The identified five families of TFs in B. napus

Subfamily	BnAP2/EREBP	Subfami	BnbZIP	Subfamily	BnMYB	Subfamily	BnNAC	Subfamily	BnWRKY
DREB_A-5	BnaA07g21980D	XII	BnaA03g05670D	1R-MYB	BnaC08g48430D	XVII	BnaA09g47260D	GroupII-c	BnaA06g23750D
DREB_A-6	BnaA08g13860D	XII	BnaC03g07230D	R2R3-MYB	BnaA03g06730D	XVII	BnaC08g41360D	GroupII-c	BnaC08g11240D
DREB_A-4	BnaA05g04450D	XII	BnaC03g07400D	1R-MYB	BnaA05g12950D	XVII	BnaC08g15440D	GroupII-c	BnaC08g10370D
ERF_B-1	BnaA08g19170D	XII	BnaA02g02830D	R2R3-MYB	BnaA04g10290D	XVII	BnaA08g24840D	GroupIII	BnaA02g05730D
ERF_B-6	BnaCnng57070D	XII	BnaC02g06270D	R2R3-MYB	BnaA03g17580D	XVII	BnaC05g09630D	GroupII-c	BnaA04g26970D
ERF_B-3	BnaC05g04210D	XII	BnaC09g42010D	R2R3-MYB	BnaC06g43380D	XVII	BnaA06g08290D	GroupI	BnaCnng68630D
AP2	BnaA02g03590D	XII	BnaA10g18440D	R2R3-MYB	BnaC02g00420D	XVII	BnaC09g13140D	GroupI	BnaA04g22040D
ERF_B-5	BnaAnng35170D	XII	BnaC02g38190D	1R-MYB	BnaA03g13950D	XVII	BnaA09g12800D	GroupII-c	BnaA09g07010D
DREB_A-2	BnaCnng75020D	XII	BnaA06g39730D	1R-MYB	BnaA06g11800D	XVI	BnaA09g06060D	GroupII-e	BnaCnng22580D
DREB_A-4	BnaA04g25970D	XII	BnaC07g26080D	R2R3-MYB	BnaC09g01940D	XVI	BnaC09g05610D	GroupII-c	BnaC07g49530D
ERF_B-1	BnaC02g05060D	XII	BnaA02g20110D	R2R3-MYB	BnaA08g08440D	XVI	BnaA02g33380D	GroupII-c	BnaA06g40630D
DREB_A-5	BnaA08g22160D	XII	BnaA06g08590D	1R-MYB	BnaA09g44370D	XVI	BnaC03g74980D	GroupII-b	BnaC09g13680D
ERF_B-4	BnaCnng24520D	XII	BnaC01g30400D	1R-MYB	BnaC03g70060D	XVI	BnaA06g21810D	GroupIII	BnaA09g05000D
DREB_A-5	BnaA07g12040D	XII	BnaC08g40240D	1R-MYB	BnaA04g24320D	XV	BnaC07g16780D	GroupI	BnaA02g36910D
DREB_A-6	BnaA04g12700D	XII	BnaC09g22200D	1R-MYB	BnaAnng36570D	XV	BnaA07g12690D	GroupII-d	BnaC04g36310D
DREB_A-4	BnaC04g44720D	XII	BnaA09g19470D	1R-MYB	BnaC02g46900D	XV	BnaA07g02060D	GroupII-c	BnaCnng26190D
ERF	BnaC08g08940D	XII	BnaA02g27460D	1R-MYB	BnaA08g00770D	XV	BnaAnng08430D	GroupII-c	BnaC06g07320D
ERF_B-1	BnaA05g24390D	XII	BnaC02g35400D	1R-MYB	BnaC02g07210D	XV	BnaC07g46020D	GroupII-b	BnaC03g29850D
DREB_A-4	BnaA08g12010D	XII	BnaCnng73390D	1R-MYB	BnaC05g30570D	XV	BnaC01g02440D	GroupII-b	BnaA09g44440D
ERF_B-6	BnaA10g21280D	XII	BnaA06g33560D	R2R3-MYB	BnaA10g16980D	XV	BnaA01g01440D	GroupII-c	BnaCnng28950D
DREB_A-4	BnaA05g23420D	XI	BnaA02g14100D	R2R3-MYB	BnaC08g24310D	XV	BnaC01g02450D	GroupII-b	BnaA02g35700D
ERF_B-3	BnaA06g35500D	XI	BnaC02g18730D	1R-MYB	BnaC03g65090D	XIV	BnaA02g15660D	GroupIII	BnaAnng38320D
DREB_A-4	BnaC04g50030D	X	BnaA09g39870D	R2R3-MYB	BnaAnng06630D	XIV	BnaC02g20880D	GroupII-b	BnaA09g00350D
DREB_A-5	BnaA07g33640D	X	BnaC08g32220D	1R-MYB	BnaA03g21820D	XIV	BnaA07g29730D	GroupII-b	BnaC02g26030D
ERF_B-3	BnaC02g01530D	X	BnaC06g18530D	1R-MYB	BnaA02g15320D	XIV	BnaC06g32940D	GroupIII	BnaC03g22830D
RAV	BnaC06g03910D	X	BnaA07g19330D	R2R3-MYB	BnaC03g51750D	XIII	BnaA05g17950D	GroupIII	BnaC03g22840D
ERF_B-1	BnaC07g10480D	X	BnaA07g21710D	1R-MYB	BnaC03g01040D	XIII	BnaC05g28390D	GroupIII	BnaCnng74680D
ERF_B-4	BnaAnng13660D	X	BnaC06g22430D	R2R3-MYB	BnaAnng06640D	XIII	BnaC05g24890D	GroupIII	BnaAnng39080D
ERF_B-4	BnaA02g01570D	X	BnaCnng21800D	R2R3-MYB	BnaAnng23930D	XIII	BnaA09g24190D	GroupI	BnaA08g12810D
RAV	BnaC02g18650D	X	BnaA02g17180D	1R-MYB	BnaA09g55480D	XIII	BnaC04g00680D	GroupII-d	BnaA08g12420D
AP2	BnaA01g00290D	X	BnaC06g01290D	R2R3-MYB	BnaA09g02730D	XIII	BnaA05g01110D	GroupII-c	BnaC04g49810D
ERF_B-3	BnaC08g43940D	X	BnaA06g25370D	R2R3-MYB	BnaA08g29930D	XIII	BnaC04g51650D	GroupII-c	BnaCnng75330D
DREB_A-6	BnaC08g08930D	X	BnaA07g01890D	R2R3-MYB	BnaCnng61660D	XIII	BnaAnng41940D	GroupII-c	BnaAnng39820D
ERF_B-4	BnaC09g43740D	X	BnaC09g09660D	R2R3-MYB	BnaCnng19930D	XIII	BnaA09g39480D	GroupIII	BnaC04g01540D
ERF_B-6	BnaA03g48270D	X	BnaA09g09500D	1R-MYB	BnaC07g48440D	XIII	BnaC08g31830D	GroupII-d	BnaC03g17030D
ERF_B-3	BnaCnng09130D	X	BnaA10g13430D	R2R3-MYB	BnaC06g30790D	XIII	BnaC06g18290D	GroupI	BnaC03g16740D
ERF_B-1	BnaC08g40860D	X	BnaC03g65930D	1R-MYB	BnaAnng03960D	XIII	BnaC06g13570D	GroupII-c	BnaA08g29320D
DREB_A-4	BnaC03g15720D	X	BnaA08g11080D	R2R3-MYB	BnaA06g15640D	XIII	BnaA07g19100D	GroupII-e	BnaC01g15320D
DREB_A-4	BnaC09g45000D	X	BnaA03g52920D	R2R3-MYB	BnaA02g03170D	XII	BnaA07g35010D	GroupIII	BnaC04g55610D
ERF	BnaA09g54740D	X	BnaC07g45120D	R2R3-MYB	BnaCnng21270D	XII	BnaC06g39940D	GroupII-c	BnaCnng77260D
ERF_B-5	BnaA03g12320D	X	BnaA01g02570D	1R-MYB	BnaA08g29620D	XII	BnaC02g25890D	GroupII-b	BnaA03g59960D
AP2	BnaC03g48820D	X	BnaC01g03810D	R2R3-MYB	BnaC07g37430D	XII	BnaAnng39480D	GroupII-c	BnaC02g40180D
DREB_A-2	BnaAnng23490D	IX	BnaC09g15210D	1R-MYB	BnaC01g01430D	XII	BnaCnng69540D	GroupIII	BnaA02g21850D
ERF_B-5	BnaA01g13420D	IX	BnaAnng23880D	R2R3-MYB	BnaA08g29670D	XII	BnaAnng32450D	GroupII-d	BnaA01g34790D
ERF	BnaC06g40770D	IX	BnaCnng60880D	R2R3-MYB	BnaC06g35510D	XI	BnaCnng40940D	GroupII-e	BnaC05g23680D
ERF_B-3	BnaA08g08310D	IX	BnaC08g21370D	R2R3-MYB	BnaC02g41860D	XI	BnaC03g28970D	GroupII-c	BnaC03g70660D
DREB_A-4	BnaAnng37500D	IX	BnaCnng21630D	R2R3-MYB	BnaCnng29520D	XI	BnaA03g24360D	GroupII-c	BnaC09g11790D
ERF_B-3	BnaCnng05080D	IX	BnaAnng05020D	R2R3-MYB	BnaCnng31270D	X	BnaC03g73730D	GroupII-c	BnaAnng41650D
ERF_B-5	BnaA02g10340D	IX	BnaUnng03790D	R2R3-MYB	BnaA02g13730D	X	BnaA03g32550D	GroupII-c	BnaC06g11020D
AP2	BnaA07g12050D	IX	BnaA01g00310D	R2R3-MYB	BnaA10g24770D	X	BnaA05g26240D	GroupII-c	BnaA09g26610D
ERF_B-6	BnaC08g16630D	IX	BnaC01g01320D	1R-MYB	BnaC08g18480D	X	BnaC05g40360D	GroupII-b	BnaA06g13050D
ERF_B-3	BnaA06g40170D	IX	BnaA03g54260D	1R-MYB	BnaCnng35720D	X	BnaC03g70990D	GroupI	BnaCnng39610D
ERF_B-5	BnaA03g46240D	IX	BnaC07g46750D	1R-MYB	BnaA03g12550D	X	BnaA08g00130D	GroupII-c	BnaCnng39880D
ERF_B-6	BnaC02g35790D	VIII	BnaC07g27220D	1R-MYB	BnaA08g13950D	X	BnaC01g09890D	GroupII-c	BnaCnng39890D
DREB_A-4	BnaCnng77210D	VIII	BnaA06g29500D	1R-MYB	BnaAnng39140D	X	BnaA01g08340D	GroupIII	BnaC09g36400D
AP2	BnaC02g21480D	VIII	BnaA09g03330D	R2R3-MYB	BnaC08g38370D	X	BnaA03g58970D	GroupII-e	BnaA02g10650D
DREB_A-5	BnaAnng28960D	VIII	BnaC09g02690D	1R-MYB	BnaA02g03510D	X	BnaC07g41390D	GroupIII	BnaA09g35840D
DREB_A-4	BnaA10g20950D	VII	BnaCnng29250D	R2R3-MYB	BnaC09g05060D	X	BnaA08g13670D	GroupIII	BnaCnng41380D
AP2	BnaA02g08130D	VII	BnaC08g25370D	R2R3-MYB	BnaA07g25800D	X	BnaC08g13100D	GroupII-c	BnaC04g51410D
DREB_A-2	BnaC04g53380D	VII	BnaA09g34460D	1R-MYB	BnaA10g10220D	IX	BnaA09g41400D	GroupII-c	BnaA09g55920D
ERF_B-6	BnaA06g33270D	VII	BnaC03g21720D	R2R3-MYB	BnaC07g46430D	IX	BnaC08g34030D	GroupII-b	BnaCnng42630D
ERF_B-6	BnaA08g23880D	VII	BnaA03g18190D	R2R3-MYB	BnaA06g03350D	IX	BnaC04g36420D	GroupII-b	BnaCnng05780D
DREB_A-5	BnaA07g10800D	VII	BnaA04g27730D	R2R3-MYB	BnaA04g10300D	IX	BnaA04g14380D	GroupII-d	BnaA04g17690D
ERF_B-3	BnaC08g31500D	VII	BnaC06g15330D	R2R3-MYB	BnaA05g29580D	IX	BnaC05g38460D	GroupI	BnaA04g17420D
AP2	BnaA07g13990D	VII	BnaC09g00090D	R2R3-MYB	BnaC07g24710D	IX	BnaC04g34520D	GroupIII	BnaCnng45760D
DREB_A-2	BnaC02g08060D	VII	BnaA09g00980D	1R-MYB	BnaC06g24140D	IX	BnaA03g33770D	GroupII-c	BnaA04g25840D
DREB_A-4	BnaA03g52210D	VII	BnaA02g31000D	R2R3-MYB	BnaA05g32240D	IX	BnaC03g38960D	GroupI	BnaA05g34850D
ERF_B-5	BnaCnng73120D	VII	BnaC02g39310D	1R-MYB	BnaC02g27760D	IX	BnaC01g36350D	GroupII-c	BnaA02g34400D
RAV	BnaCnng37790D	VII	BnaC07g27440D	1R-MYB	BnaA01g37390D	IX	BnaA01g28990D	GroupI	BnaA05g12160D
DREB_A-4	BnaC03g19550D	VII	BnaA06g29270D	R2R3-MYB	BnaA04g10190D	IX	BnaA02g09970D	GroupII-d	BnaA05g11870D
ERF_B-2	BnaAnng20420D	VII	BnaC07g29750D	R2R3-MYB	BnaA04g18810D	IX	BnaC02g13890D	GroupII-e	BnaA03g46020D
ERF_B-1	BnaC03g41530D	VII	BnaA06g27230D	1R-MYB	BnaAnng30810D	IX	BnaA02g18100D	GroupIII	BnaA03g46280D
ERF_B-3	BnaA01g02720D	VII	BnaC02g41050D	R2R3-MYB	BnaA01g11280D	IX	BnaC02g24050D	GroupII-d	BnaA03g46550D
ERF_B-4	BnaA10g23760D	VII	BnaA02g32330D	1R-MYB	BnaA07g11710D	IX	BnaA07g32710D	GroupII-c	BnaA09g39820D

ERF_B-5	BnaA03g24540D	VII	BnaCnng21210D	R2R3-MYB	BnaA08g10420D	IX	BnaC06g37170D	GroupII-b	BnaC06g30870D
ERF_B-3	BnaA01g09230D	VII	BnaA03g39740D	R2R3-MYB	BnaA03g10180D	IX	BnaA01g26760D	GroupIII	BnaC06g15910D
ERF	BnaA02g06490D	VII	BnaC02g41810D	1R-MYB	BnaA04g01380D	IX	BnaCnng63190D	GroupII-c	BnaA10g10500D
ERF_B-2	BnaA05g23130D	VII	BnaAnng06600D	R2R3-MYB	BnaA02g01290D	IX	BnaCnng55590D	GroupII-c	BnaA09g16360D
DREB_A-5	BnaC01g07340D	VII	BnaA09g05450D	R2R3-MYB	BnaCnng62710D	IX	BnaCnng60050D	GroupII-c	BnaA06g36020D
ERF	BnaC03g23470D	VII	BnaC09g05030D	R2R3-MYB	BnaA09g56310D	IX	BnaCnng66480D	GroupI	BnaA09g45940D
ERF	BnaC09g35430D	VII	BnaUnng01680D	R2R3-MYB	BnaA10g22930D	IX	BnaAnng11180D	GroupII-d	BnaA02g31030D
DREB_A-1	BnaAnng34260D	VII	BnaA03g02360D	R2R3-MYB	BnaA07g30860D	IX	BnaC03g40490D	GroupII-c	BnaA09g11350D
DREB_A-6	BnaC05g17550D	VII	BnaC04g01070D	R2R3-MYB	BnaA03g32470D	IX	BnaA03g07000D	GroupIII	BnaCnng52600D
DREB_A-2	BnaC04g55440D	VII	BnaA05g01520D	R2R3-MYB	BnaC07g34790D	IX	BnaC03g08880D	GroupII-c	BnaA09g17960D
ERF_B-5	BnaC07g41100D	VII	BnaC03g25660D	R2R3-MYB	BnaA03g53110D	IX	BnaA02g04040D	GroupIII	BnaA03g19190D
ERF_B-3	BnaA01g23940D	VII	BnaA09g00170D	1R-MYB	BnaC09g07170D	IX	BnaC02g07990D	GroupIII	BnaA03g19180D
DREB_A-1	BnaAnng36290D	VII	BnaCnng01910D	R2R3-MYB	BnaA02g09340D	IX	BnaC01g40260D	GroupIII	BnaC02g09670D
ERF_B-1	BnaA09g46790D	VII	BnaA08g15400D	1R-MYB	BnaA02g20260D	IX	BnaC05g47500D	GroupII-b	BnaAnng23990D
ERF_B-1	BnaA05g18740D	VII	BnaC03g61840D	1R-MYB	BnaC03g00940D	IX	BnaA05g37400D	GroupI	BnaC03g13730D
DREB_A-5	BnaC07g14220D	VII	BnaA01g01100D	1R-MYB	BnaC03g48630D	IX	BnaAnng06380D	GroupII-c	BnaC03g60600D
DREB_A-4	BnaC06g21120D	VII	BnaC01g02130D	R2R3-MYB	BnaCnng12230D	IX	BnaC02g01470D	GroupIII	BnaA09g24840D
ERF_B-5	BnaC02g20580D	VII	BnaC04g44430D	R2R3-MYB	BnaC01g17440D	IX	BnaA10g30260D	GroupI	BnaC05g10200D
ERF_B-3	BnaC03g17400D	VII	BnaC05g24270D	R2R3-MYB	BnaCnng68780D	IX	BnaCnng66010D	GroupII-d	BnaC03g67520D
ERF_B-1	BnaC01g36330D	VII	BnaA09g24610D	3R-MYB	BnaC03g04820D	IX	BnaC07g49940D	GroupI	BnaC03g67830D
DREB_A-4	BnaA08g24660D	VII	BnaA03g56410D	R2R3-MYB	BnaC06g34360D	IX	BnaAnng07000D	GroupI	BnaA03g10980D
ERF_B-1	BnaC02g30930D	VII	BnaA04g20520D	1R-MYB	BnaC04g44010D	IX	BnaA09g02500D	GroupI	BnaC02g34990D
ERF_B-3	BnaC01g31050D	VII	BnaA05g08520D	R2R3-MYB	BnaA02g10750D	IX	BnaCnng49350D	GroupI	BnaA05g34110D
ERF	BnaC09g46030D	VII	BnaC04g09600D	1R-MYB	BnaA03g40400D	IX	BnaC07g25530D	GroupIII	BnaA02g19570D
AP2	BnaA02g16050D	VI	BnaC03g76870D	R2R3-MYB	BnaA09g44500D	IX	BnaA06g31080D	GroupIII	BnaA02g19560D
AP2	BnaA07g16350D	VI	BnaC01g02720D	1R-MYB	BnaC08g26530D	IX	BnaC04g31690D	GroupII-c	BnaC04g00310D
DREB_A-5	BnaA03g53850D	VI	BnaC09g09560D	1R-MYB	BnaA05g07470D	IX	BnaA04g09470D	GroupII-c	BnaAnng24700D
DREB_A-5	BnaC06g00880D	VI	BnaA09g09300D	R2R3-MYB	BnaC09g50990D	IX	BnaC06g12550D	GroupII-c	BnaA09g11810D
ERF_B-2	BnaA01g29490D	V	BnaC05g02200D	R2R3-MYB	BnaA09g44790D	IX	BnaA07g14730D	GroupIII	BnaC02g22950D
ERF_B-6	BnaCnng59450D	V	BnaA10g02300D	1R-MYB	BnaC02g16640D	VIII	BnaA05g18430D	GroupII-b	BnaA07g28340D
ERF_B-1	BnaC08g40750D	V	BnaCnng27030D	R2R3-MYB	BnaAnng05010D	VIII	BnaC05g28020D	GroupII-d	BnaA04g13570D
AP2	BnaC05g27100D	V	BnaA06g36330D	R2R3-MYB	BnaA03g37010D	VIII	BnaC06g08090D	GroupII-c	BnaA04g11210D
ERF_B-1	BnaCnng69830D	V	BnaA06g37000D	R2R3-MYB	BnaC03g61550D	VIII	BnaA09g23230D	GroupII-b	BnaC03g67380D
ERF_B-1	BnaA06g02670D	V	BnaC04g17630D	R2R3-MYB	BnaC06g42760D	VIII	BnaA05g18390D	GroupII-b	BnaA02g13630D
DREB_A-5	BnaC08g21640D	V	BnaC04g52290D	1R-MYB	BnaC08g10990D	VIII	BnaC05g28010D	GroupII-b	BnaA09g13370D
ERF_B-3	BnaA09g50010D	V	BnaA05g01970D	1R-MYB	BnaC07g34310D	VIII	BnaA08g06490D	GroupIII	BnaC06g13990D
DREB_A-1	BnaA08g30930D	V	BnaC04g56840D	R2R3-MYB	BnaC09g54480D	VIII	BnaC08g07040D	GroupII-d	BnaC03g33830D
DREB_A-6	BnaA09g12290D	V	BnaA04g23690D	1R-MYB	BnaC04g54130D	VIII	BnaA07g10300D	GroupII-c	BnaC06g30480D
DREB_A-5	BnaC09g07480D	V	BnaC04g24820D	R2R3-MYB	BnaC05g20740D	VIII	BnaC07g13550D	GroupII-e	BnaC03g65200D
ERF_B-6	BnaC08g12290D	V	BnaA04g02780D	R2R3-MYB	BnaA03g16870D	VIII	BnaA07g13130D	GroupII-d	BnaC05g47140D
ERF_B-6	BnaC02g09020D	V	BnaA07g16990D	1R-MYB	BnaC06g02940D	VIII	BnaC04g16940D	GroupII-c	BnaA01g05500D
DREB_A-5	BnaC05g18050D	V	BnaCnng20400D	R2R3-MYB	BnaA03g44700D	VIII	BnaC01g23890D	GroupIII	BnaC04g01210D
DREB_A-2	BnaCnng07330D	V	BnaA09g36130D	R2R3-MYB	BnaAnng17460D	VIII	BnaA01g33110D	GroupI	BnaA03g48030D
ERF_B-3	BnaCnng05070D	V	BnaC08g27660D	R2R3-MYB	BnaA09g30490D	VIII	BnaA01g33090D	GroupII-c	BnaA07g07600D
ERF_B-3	BnaA07g06760D	V	BnaAnng07530D	1R-MYB	BnaA10g10930D	VIII	BnaC03g10790D	GroupI	BnaC05g48590D
ERF	BnaCnng73930D	V	BnaA05g08020D	R2R3-MYB	BnaA01g33290D	VIII	BnaA03g08460D	GroupII-c	BnaC09g17450D
DREB_A-1	BnaA10g07630D	V	BnaC04g09030D	R2R3-MYB	BnaA01g02240D	VIII	BnaA10g14070D	GroupII-e	BnaA04g20400D
DREB_A-1	BnaC08g41070D	V	BnaC03g54810D	R2R3-MYB	BnaCnng06080D	VIII	BnaA02g05540D	GroupIII	BnaC04g21660D
DREB_A-6	BnaC02g24770D	V	BnaCnng04010D	R2R3-MYB	BnaC05g45330D	VIII	BnaC02g09420D	GroupI	BnaA03g51140D
DREB_A-6	BnaA06g24080D	V	BnaA10g28780D	R2R3-MYB	BnaA07g34700D	VIII	BnaC03g36600D	GroupII-d	BnaA03g51590D
DREB_A-2	BnaCnng69090D	V	BnaC06g00420D	R2R3-MYB	BnaA09g05480D	VIII	BnaA03g31260D	GroupI	BnaC02g01720D
ERF_B-1	BnaC06g10050D	V	BnaC07g44670D	R2R3-MYB	BnaC04g17160D	VIII	BnaAnng34430D	GroupI	BnaA08g14350D
ERF_B-3	BnaC01g03990D	V	BnaAnng26550D	R2R3-MYB	BnaA09g07190D	VIII	BnaA01g31660D	GroupI	BnaC03g21360D
ERF_B-5	BnaA03g48910D	V	BnaC01g04330D	R2R3-MYB	BnaC02g22630D	VIII	BnaA01g31670D	GroupII-c	BnaC03g49470D
ERF_B-2	BnaA03g34290D	V	BnaC06g02640D	1R-MYB	BnaA05g01050D	VIII	BnaA01g31680D	GroupII-c	BnaA02g24470D
ERF_B-3	BnaC09g27360D	V	BnaA06g03040D	R2R3-MYB	BnaA05g30870D	VIII	BnaC03g36610D	GroupII-e	BnaA01g13230D
DREB_A-1	BnaA03g13620D	V	BnaCnng41320D	1R-MYB	BnaA03g43060D	VIII	BnaA03g31270D	GroupII-c	BnaCnng11190D
DREB_A-6	BnaC09g11080D	V	BnaA03g35190D	1R-MYB	BnaC01g02200D	VIII	BnaA05g28370D	GroupII-b	BnaCnng60780D
AP2	BnaC05g31430D	V	BnaA01g26200D	R2R3-MYB	BnaA02g36420D	VIII	BnaC05g42540D	GroupI	BnaA06g33730D
ERF_B-4	BnaC02g43290D	V	BnaC01g43800D	R2R3-MYB	BnaC08g20580D	VIII	BnaA03g02680D	GroupII-d	BnaA04g14500D
ERF_B-1	BnaA02g35090D	V	BnaC05g33570D	R2R3-MYB	BnaC03g32460D	VIII	BnaA10g22600D	GroupII-c	BnaCnng13810D
AP2	BnaA06g10760D	V	BnaA05g20870D	R2R3-MYB	BnaA03g49030D	VIII	BnaC03g03780D	GroupIII	BnaC04g47350D
AP2	BnaC02g44180D	IV	BnaA06g13700D	1R-MYB	BnaA01g10210D	VIII	BnaC09g47170D	GroupI	BnaA03g48160D
DREB_A-5	BnaC01g01700D	IV	BnaC05g15020D	1R-MYB	BnaC06g33880D	VIII	BnaC02g00940D	GroupII-c	BnaA03g17120D
ERF	BnaCnng71740D	III	BnaA01g27940D	3R-MYB	BnaC03g35980D	VIII	BnaA06g23210D	GroupII-c	BnaA03g17130D
DREB_A-2	BnaA07g21680D	III	BnaA03g34610D	R2R3-MYB	BnaC07g36700D	VIII	BnaC03g50150D	GroupII-c	BnaC02g43320D
DREB_A-4	BnaA07g33380D	III	BnaA05g22650D	1R-MYB	BnaCnng65380D	VIII	BnaC03g71220D	GroupII-d	BnaC04g13870D
ERF_B-1	BnaAnng26790D	III	BnaA09g36010D	1R-MYB	BnaA05g23050D	VIII	BnaAnng17650D	GroupII-e	BnaAnng30430D
AP2	BnaCnng39690D	III	BnaC05g35930D	R2R3-MYB	BnaAnng41910D	VIII	BnaC02g02740D	GroupII-c	BnaC03g69190D
ERF_B-3	BnaA03g40380D	III	BnaCnng20200D	1R-MYB	BnaA03g30700D	VIII	BnaAnng34420D	GroupII-c	BnaC03g69160D
RAV	BnaA09g28670D	III	BnaA02g00920D	R2R3-MYB	BnaA02g06100D	VIII	BnaA01g31650D	GroupIII	BnaA01g13440D
DREB_A-2	BnaAnng01320D	III	BnaC09g45380D	1R-MYB	BnaC02g38100D	VIII	BnaA03g01250D	GroupI	BnaCnng64000D
ERF_B-6	BnaC09g37870D	III	BnaA10g21200D	1R-MYB	BnaC03g25810D	VIII	BnaC09g50530D	GroupII-c	BnaA06g16820D
DREB_A-4	BnaC04g38070D	III	BnaA01g31420D	R2R3-MYB	BnaCnng43220D	VIII	BnaA10g25760D	GroupII-c	BnaC04g05170D
ERF_B-3	BnaA09g16520D	III	BnaA09g24670D	1R-MYB	BnaC03g13270D	VII	BnaA01g34470D	GroupII-c	BnaA04g12540D
DREB_A-5	BnaA09g07540D	III	BnaCnng38290D	1R-MYB	BnaC03g26090D	VII	BnaCnng18420D	GroupI	BnaC08g40010D
DREB_A-6	BnaC06g20590D	III	BnaA04g02670D	R2R3-MYB	BnaC06g14460D	VII	BnaA05g32430D	GroupII-e	BnaA09g00120D

ERF_B-3	BnaC03g66140D	III	BnaC04g52230D	R2R3-MYB	BnaA08g22480D	VII	BnaCnng06310D	GroupII-c	BnaC07g27590D
ERF_B-2	BnaCnng27470D	III	BnaC04g56770D	1R-MYB	BnaC02g10950D	VII	BnaC09g40700D	GroupII-d	BnaC07g27510D
RAV	BnaA08g19490D	III	BnaA04g23630D	1R-MYB	BnaC08g37720D	VII	BnaA03g06460D	GroupII-e	BnaA09g55250D
ERF_B-1	BnaC05g21820D	III	BnaC03g23710D	R2R3-MYB	BnaC03g37840D	VII	BnaC03g08290D	GroupI	BnaC09g53550D
ERF_B-1	BnaA03g33790D	III	BnaA03g19810D	R2R3-MYB	BnaC08g06060D	VII	BnaC05g35830D	GroupI	BnaCnng66020D
ERF	BnaA10g21750D	III	BnaC04g52770D	3R-MYB	BnaA04g09530D	VII	BnaA05g22540D	GroupII-b	BnaA09g20470D
DREB_A-2	BnaAnng13220D	III	BnaA05g02840D	R2R3-MYB	BnaCnng43450D	VII	BnaCnng36980D	GroupI	BnaC03g44980D
DREB_A-4	BnaA02g11090D	III	BnaC03g10840D	R2R3-MYB	BnaA06g21900D	VII	BnaA02g12310D	GroupII-b	BnaAnng32610D
ERF_B-1	BnaC05g09160D	III	BnaC06g16270D	R2R3-MYB	BnaA06g12870D	VII	BnaCnng72050D	GroupII-c	BnaA02g14710D
ERF_B-6	BnaA07g24590D	III	BnaA09g37270D	R2R3-MYB	BnaC02g05990D	VII	BnaA07g17790D	GroupII-d	BnaAnng10540D
ERF_B-1	BnaC03g06100D	III	BnaC08g29000D	3R-MYB	BnaC09g45060D	VII	BnaC03g71120D	GroupII-e	BnaA09g54080D
ERF_B-1	BnaA07g36710D	III	BnaC09g48330D	R2R3-MYB	BnaC06g39570D	VII	BnaA08g00010D	GroupIII	BnaA07g16850D
ERF_B-2	BnaC06g33570D	III	BnaA10g23610D	R2R3-MYB	BnaCnng24780D	VII	BnaA06g21090D	GroupII-b	BnaA07g20110D
ERF_B-3	BnaC07g31340D	III	BnaAnng36910D	3R-MYB	BnaA10g27190D	VII	BnaC03g52400D	GroupI	BnaC07g42990D
DREB_A-4	BnaA02g15420D	III	BnaC04g33650D	1R-MYB	BnaC02g12480D	VII	BnaC08g21160D	GroupII-d	BnaC07g43320D
DREB_A-4	BnaA09g23540D	III	BnaA04g12040D	R2R3-MYB	BnaA02g35490D	VII	BnaA06g15870D	GroupII-b	BnaC07g43490D
RAV	BnaA06g07470D	III	BnaA04g12030D	1R-MYB	BnaC01g33300D	VII	BnaA09g04810D	GroupII-c	BnaC07g47230D
DREB_A-5	BnaC07g16160D	III	BnaC04g33660D	R2R3-MYB	BnaC04g32490D	VII	BnaC09g04330D	GroupII-c	BnaC03g24960D
DREB_A-1	BnaC09g28190D	III	BnaAnng33910D	R2R3-MYB	BnaC09g05300D	VII	BnaC07g29890D	GroupIII	BnaC03g25800D
DREB_A-4	BnaA02g01080D	III	BnaC08g35920D	R2R3-MYB	BnaA03g05370D	VII	BnaA06g27060D	GroupII-c	BnaA07g27840D
ERF_B-1	BnaA09g46720D	III	BnaAnng39310D	1R-MYB	BnaA06g15430D	VII	BnaA05g17900D	GroupII-b	BnaA07g26870D
ERF_B-6	BnaC06g30140D	III	BnaC05g51490D	R2R3-MYB	BnaC08g18090D	VII	BnaCnng49700D	GroupII-e	BnaC02g14940D
DREB_A-4	BnaC03g66900D	III	BnaA08g04710D	R2R3-MYB	BnaA02g16430D	VII	BnaCnng38230D	GroupII-b	BnaA08g22350D
ERF_B-6	BnaA03g07750D	III	BnaC08g05560D	1R-MYB	BnaC09g10430D	VII	BnaA10g21230D	GroupI	BnaA08g24160D
ERF_B-6	BnaA06g27670D	III	BnaA04g12020D	1R-MYB	BnaC07g18400D	VII	BnaCnng20180D	GroupII-c	BnaC09g44020D
DREB_A-6	BnaA02g34570D	III	BnaC04g33670D	R2R3-MYB	BnaAnng03830D	VII	BnaA02g00900D	GroupI	BnaC08g12240D
ERF_B-3	BnaA01g23970D	III	BnaA09g43320D	1R-MYB	BnaA07g36950D	VII	BnaA02g36760D	GroupII-d	BnaC04g41330D
ERF_B-3	BnaC04g01080D	III	BnaC08g35930D	1R-MYB	BnaC06g02930D	VII	BnaC02g32460D	GroupI	BnaC04g41050D
DREB_A-4	BnaC06g17770D	III	BnaA06g37290D	1R-MYB	BnaC08g18470D	VII	BnaA06g40380D	GroupII-b	BnaC04g40940D
ERF_B-6	BnaA06g27540D	III	BnaC07g47540D	1R-MYB	BnaA02g32790D	VII	BnaC07g19280D	GroupI	BnaA06g08890D
ERF_B-6	BnaA08g14320D	III	BnaC03g60240D	R2R3-MYB	BnaA10g13590D	VII	BnaC07g34980D	GroupII-c	BnaC07g09430D
ERF	BnaA05g20050D	III	BnaA08g16860D	3R-MYB	BnaA10g20990D	VII	BnaA03g43500D	GroupII-e	BnaC07g08810D
ERF_B-3	BnaC07g08350D	III	BnaA04g23410D	R2R3-MYB	BnaC01g01250D	VII	BnaC01g10510D	GroupII-c	BnaC01g10570D
RAV	BnaA05g15710D	III	BnaC04g47290D	R2R3-MYB	BnaC01g20650D	VII	BnaA01g08830D	GroupII-e	BnaC05g22280D
DREB_A-4	BnaC05g37110D	III	BnaC04g01480D	1R-MYB	BnaC02g10960D	VII	BnaC08g07470D	GroupII-c	BnaC05g22810D
ERF_B-1	BnaA08g01300D	III	BnaA05g01800D	1R-MYB	BnaC05g50690D	VII	BnaA08g30060D	GroupII-e	BnaC04g23420D
ERF	BnaAnng40110D	III	BnaC03g22760D	3R-MYB	BnaA08g12050D	VII	BnaA08g29850D	GroupII-d	BnaA03g28720D
ERF_B-3	BnaC09g20100D	III	BnaA03g19140D	R2R3-MYB	BnaC02g46060D	VI	BnaC04g52880D	GroupI	BnaA03g27660D
ERF_B-6	BnaC09g04040D	III	BnaC08g44990D	R2R3-MYB	BnaC06g34910D	VI	BnaA05g34370D	GroupII-c	BnaC07g35130D
DREB_A-4	BnaC04g03870D	III	BnaA09g49720D	R2R3-MYB	BnaA04g00800D	VI	BnaC03g23960D	GroupII-b	BnaC06g29310D
DREB_A-4	BnaC08g07150D	III	BnaA08g28740D	R2R3-MYB	BnaC01g41180D	VI	BnaA04g24740D	GroupII-e	BnaC02g27650D
ERF_B-6	BnaAnng20260D	III	BnaC08g01910D	R2R3-MYB	BnaA03g22590D	VI	BnaC04g48620D	GroupII-c	BnaC04g14500D
ERF_B-4	BnaA03g04290D	III	BnaA08g28440D	R2R3-MYB	BnaA06g30710D	VI	BnaA03g19960D	GroupI	BnaC07g22240D
ERF_B-6	BnaC07g40560D	III	BnaC08g01660D	1R-MYB	BnaA10g25530D	VI	BnaC01g38180D	GroupII-c	BnaC09g19320D
DREB_A-6	BnaA01g05760D	III	BnaC05g04130D	R2R3-MYB	BnaC02g37590D	VI	BnaA01g30220D	GroupII-d	BnaC01g16120D
AP2	BnaA05g15470D	III	BnaA10g04020D	1R-MYB	BnaA05g03970D	VI	BnaA03g32520D	GroupIII	BnaC01g15640D
ERF	BnaA06g24400D	III	BnaA04g18340D	R2R3-MYB	BnaC01g37570D	VI	BnaCnng44060D	GroupI	BnaA05g07220D
ERF_B-3	BnaC01g10080D	III	BnaC04g42420D	1R-MYB	BnaC02g37370D	VI	BnaC05g40430D	GroupII-c	BnaC06g25390D
ERF_B-3	BnaC04g42060D	III	BnaCnng30720D	1R-MYB	BnaC05g47520D	VI	BnaA05g26290D	GroupIII	BnaAnng00020D
DREB_A-2	BnaC08g28620D	III	BnaA05g11370D	R2R3-MYB	BnaC08g17650D	VI	BnaA07g14780D	GroupI	BnaAnng01980D
ERF_B-6	BnaA04g14830D	II	BnaC09g48720D	R2R3-MYB	BnaA08g15610D	VI	BnaC06g12700D	GroupII-c	BnaA02g31540D
DREB_A-4	BnaC09g12830D	II	BnaA10g24100D	R2R3-MYB	BnaC09g03530D	VI	BnaCnng46350D	GroupII-d	BnaC02g39360D
ERF_B-1	BnaC02g22960D	II	BnaCnng67350D	R2R3-MYB	BnaA06g21780D	VI	BnaA08g19970D	GroupII-c	BnaC02g39170D
AP2	BnaC02g00280D	II	BnaAnng36310D	1R-MYB	BnaCnng67570D	VI	BnaA07g28210D	GroupII-b	BnaA01g05060D
DREB_A-4	BnaA09g04470D	II	BnaUnng03970D	R2R3-MYB	BnaCnng63050D	VI	BnaC06g27950D	GroupIII	BnaA04g23480D
ERF_B-3	BnaA09g39350D	II	BnaAnng33510D	1R-MYB	BnaA05g28230D	VI	BnaC04g43920D	GroupIII	BnaA04g23470D
DREB_A-4	BnaAnng34830D	II	BnaC08g43230D	R2R3-MYB	BnaC03g71860D	VI	BnaA04g19530D	GroupII-b	BnaCnng01360D
DREB_A-5	BnaA08g12710D	II	BnaA09g48940D	R2R3-MYB	BnaCnng29120D	VI	BnaA05g10110D	GroupII-e	BnaCnng02000D
ERF_B-1	BnaC03g09100D	II	BnaC05g06030D	1R-MYB	BnaC02g02590D	VI	BnaC04g11040D	GroupII-c	BnaA07g24310D
RAV	BnaCnng04580D	II	BnaA06g04770D	R2R3-MYB	BnaC03g26620D	VI	BnaC04g11030D	GroupI	BnaA03g17820D
ERF	BnaA08g04940D	II	BnaA05g26790D	1R-MYB	BnaCnng78680D	VI	BnaA10g05960D	GroupII-c	BnaA05g13660D
ERF	BnaA03g22100D	II	BnaCnng22950D	R2R3-MYB	BnaA06g11110D	VI	BnaC09g26970D	GroupIII	BnaA04g02550D
DREB_A-6	BnaA09g30810D	II	BnaA01g30760D	R2R3-MYB	BnaA02g29490D	VI	BnaC03g71470D	GroupIII	BnaA04g02560D
DREB_A-4	BnaA07g18690D	II	BnaC03g37460D	R2R3-MYB	BnaC06g29460D	VI	BnaA03g04240D	GroupII-e	BnaA08g10660D
ERF	BnaC08g25840D	II	BnaA03g32170D	1R-MYB	BnaA06g05130D	VI	BnaC09g43890D	GroupII-c	BnaA08g08590D
ERF_B-1	BnaA01g28970D	II	BnaC05g40810D	R2R3-MYB	BnaA05g00510D	VI	BnaA02g01460D	GroupII-c	BnaC08g32160D
ERF_B-3	BnaA07g06750D	II	BnaC09g48640D	1R-MYB	BnaC07g35020D	VI	BnaA10g20110D	GroupII-c	BnaC08g35490D
DREB_A-6	BnaC08g36140D	II	BnaA10g24020D	1R-MYB	BnaA05g19710D	VI	BnaC02g04540D	GroupII-c	BnaC04g38910D
DREB_A-2	BnaA10g16490D	II	BnaC02g01780D	R2R3-MYB	BnaC09g32700D	VI	BnaC01g40250D	GroupII-e	BnaA07g07190D
ERF_B-6	BnaA06g10040D	II	BnaAnng01920D	R2R3-MYB	BnaA06g24760D	VI	BnaA05g37410D	GroupII-c	BnaC07g19090D
DREB_A-2	BnaC04g47060D	II	BnaC06g30310D	R2R3-MYB	BnaC03g74080D	VI	BnaC05g47490D	GroupII-c	BnaA03g04160D
ERF_B-1	BnaCnng04890D	II	BnaA07g27320D	R2R3-MYB	BnaC09g05650D	VI	BnaA09g06870D	GroupII-b	BnaA03g05230D
DREB_A-4	BnaC08g30700D	II	BnaA09g30910D	1R-MYB	BnaAnng28150D	VI	BnaC09g06370D	GroupII-d	BnaC01g06900D
ERF_B-3	BnaC03g25680D	II	BnaC05g17700D	R2R3-MYB	BnaC05g40480D	VI	BnaC02g43170D	GroupII-b	BnaC01g06660D
DREB_A-1	BnaCnng49280D	II	BnaC08g20170D	R2R3-MYB	BnaC02g06730D	VI	BnaA02g34250D	GroupII-c	BnaA01g08990D
DREB_A-6	BnaAnng29660D	II	BnaA08g20970D	R2R3-MYB	BnaA07g11930D	VI	BnaA06g23510D	GroupII-b	BnaC06g19560D
DREB_A-1	BnaA09g47030D	II	BnaA07g20770D	R2R3-MYB	BnaA04g13540D	VI	BnaCnng30500D	GroupII-c	BnaC03g20650D

DREB_A-4	BnaA10g00620D	II	BnaC06g20630D	1R-MYB	BnaC01g21010D	VI	BnaA06g01770D	GroupIII	BnaA05g01840D
DREB_A-6	BnaC03g49170D	II	BnaA07g33790D	R2R3-MYB	BnaA06g37380D	VI	BnaCnng64100D	GroupIII	BnaA05g01410D
DREB_A-1	BnaC03g71900D	II	BnaC06g38430D	1R-MYB	BnaA06g26310D	VI	BnaA01g28710D	GroupII-c	BnaA05g00620D
ERF	BnaC04g02040D	II	BnaC02g00560D	1R-MYB	BnaCnng23360D	VI	BnaCnng30350D	GroupIII	BnaC07g30300D
ERF_B-2	BnaC01g35070D	II	BnaA02g00310D	R2R3-MYB	BnaA09g11780D	VI	BnaC08g43050D	GroupII-c	BnaC09g06700D
AP2	BnaC01g01710D	II	BnaC09g46670D	1R-MYB	BnaA02g25380D	VI	BnaA03g33910D	GroupII-b	BnaC02g17970D
ERF_B-4	BnaA03g02000D	II	BnaA10g22150D	R2R3-MYB	BnaC09g46360D	VI	BnaA05g24040D	GroupII-b	BnaA10g18970D
ERF_B-3	BnaA08g11220D	II	BnaC02g43620D	1R-MYB	BnaC06g12130D	VI	BnaC05g38130D	GroupII-b	BnaA10g18980D
ERF_B-4	BnaC04g44080D	II	BnaAnng04720D	R2R3-MYB	BnaCnng13680D	VI	BnaCnng10130D	GroupII-c	BnaA10g20210D
ERF	BnaA02g00560D	II	BnaC09g06840D	R2R3-MYB	BnaC09g13310D	VI	BnaA09g13880D	GroupII-b	BnaC08g37010D
ERF_B-5	BnaC02g14430D	II	BnaA06g24140D	1R-MYB	BnaA02g28360D	VI	BnaC06g30680D	GroupII-b	BnaC01g13500D
ERF_B-6	BnaCnng47540D	II	BnaC03g49070D	1R-MYB	BnaCnng23500D	VI	BnaA07g28000D	GroupIII	BnaA03g21390D
DREB_A-4	BnaC09g28200D	I	BnaA08g04620D	R2R3-MYB	BnaA09g03290D	VI	BnaC06g43410D	GroupII-c	BnaA03g20910D
ERF_B-1	BnaA09g27300D	I	BnaC03g59330D	R2R3-MYB	BnaC07g10840D	VI	BnaA07g24270D	GroupII-b	BnaC08g18410D
ERF_B-4	BnaCnng05370D	I	BnaA04g05810D	R2R3-MYB	BnaA03g02170D	VI	BnaA05g24050D	GroupII-c	BnaC03g05760D
ERF_B-4	BnaC03g49530D	I	BnaCnng36700D	R2R3-MYB	BnaC09g47470D	VI	BnaC05g38150D	GroupII-b	BnaC03g06770D
ERF_B-6	BnaA07g27210D	I	BnaC09g09130D	1R-MYB	BnaA06g11740D	VI	BnaA01g28930D	GroupII-d	BnaA03g14060D
ERF_B-1	BnaA05g14650D	I	BnaA09g08870D	R2R3-MYB	BnaCnng32030D	VI	BnaC03g39160D	GroupI	BnaA03g13820D
ERF_B-6	BnaC05g20650D	I	BnaA03g39300D	R2R3-MYB	BnaC03g26060D	VI	BnaA03g33890D	GroupII-b	BnaC05g20030D
DREB_A-4	BnaA04g15130D	I	BnaC03g46620D	1R-MYB	BnaCnng22510D	VI	BnaC01g36040D	GroupII-c	BnaC07g27240D
ERF_B-2	BnaA07g30130D	I	BnaAnng11690D	R2R3-MYB	BnaC02g22160D	VI	BnaC06g05930D	GroupII-d	BnaA06g29220D
ERF_B-1	BnaA02g22560D	I	BnaC01g03460D	R2R3-MYB	BnaC03g17390D	VI	BnaC06g05920D	GroupII-b	BnaA09g21040D
ERF_B-3	BnaCnng60520D	I	BnaC03g65600D	R2R3-MYB	BnaA01g00670D	VI	BnaA03g48570D	GroupII-c	BnaC09g03790D
DREB_A-4	BnaC06g37920D	I	BnaA08g10920D	R2R3-MYB	BnaA02g02300D	VI	BnaC07g40860D	GroupIII	BnaC09g04560D
ERF	BnaA01g34730D			1R-MYB	BnaC07g31380D	VI	BnaA01g16400D	GroupII-b	BnaA07g35260D
ERF_B-6	BnaC09g04110D			1R-MYB	BnaA05g29060D	VI	BnaC01g19550D	GroupII-c	BnaC02g32270D
ERF_B-3	BnaC07g20090D			R2R3-MYB	BnaA06g31880D	VI	BnaA02g18390D	GroupIII	BnaA06g26680D
DREB_A-5	BnaC07g13470D			1R-MYB	BnaA09g32720D	VI	BnaCnng09850D	GroupII-c	BnaA06g28100D
ERF_B-3	BnaA09g05710D			R2R3-MYB	BnaC02g07480D	VI	BnaC08g07270D	GroupII-b	BnaC06g40170D
DREB_A-4	BnaC06g32560D			R2R3-MYB	BnaA05g06600D	VI	BnaC05g00370D	GroupII-e	BnaA04g01860D
DREB_A-6	BnaA06g24070D			1R-MYB	BnaA05g32140D	VI	BnaA10g00280D	GroupIII	BnaA04g00590D
AP2	BnaC03g31040D			R2R3-MYB	BnaC08g48600D	VI	BnaAnng05950D	GroupI	BnaA01g15760D
ERF_B-3	BnaA10g04090D			R2R3-MYB	BnaA03g40690D	VI	BnaC02g00990D	GroupI	BnaA01g15570D
ERF_B-5	BnaC08g06130D			1R-MYB	BnaA08g15280D	VI	BnaA03g02640D	GroupII-d	BnaC04g35770D
DREB_A-5	BnaC06g22710D			R2R3-MYB	BnaC01g38230D	VI	BnaC03g03740D	GroupII-e	BnaA08g18040D
RAV	BnaC05g51390D			R2R3-MYB	BnaC02g39010D	VI	BnaC09g47250D	GroupII-c	BnaA03g43640D
ERF_B-3	BnaC01g40920D			R2R3-MYB	BnaA04g21820D	VI	BnaA06g22900D	GroupII-b	BnaA03g25430D
DREB_A-5	BnaA09g44290D			R2R3-MYB	BnaC07g47650D	VI	BnaC03g50570D	GroupI	BnaC04g08020D
RAV	BnaC03g77730D			R2R3-MYB	BnaA10g18810D	VI	BnaA10g22680D	GroupI	BnaC04g06800D
DREB_A-5	BnaC08g20020D			R2R3-MYB	BnaA07g24240D	VI	BnaA02g33910D	GroupI	BnaC03g32670D
ERF_B-5	BnaA07g23650D			1R-MYB	BnaA03g43540D	VI	BnaC02g42720D	GroupII-d	BnaC07g38840D
ERF_B-1	BnaC03g58550D			R2R3-MYB	BnaA07g26980D	V	BnaA07g03200D	GroupI	BnaC07g40660D
ERF_B-3	BnaA07g38140D			R2R3-MYB	BnaA09g02470D	V	BnaC07g06080D	GroupI	BnaA03g37950D
DREB_A-4	BnaC02g03940D			R2R3-MYB	BnaA06g18160D	V	BnaC03g60970D	GroupII-e	BnaC08g29410D
ERF_B-3	BnaC07g45030D			R2R3-MYB	BnaA08g09740D	V	BnaA03g39450D	GroupIII	BnaC08g27340D
ERF_B-3	BnaC07g08360D			1R-MYB	BnaA10g16590D	V	BnaC09g09210D	GroupII-e	BnaC07g38350D
ERF_B-2	BnaC04g51210D			1R-MYB	BnaA08g30200D	V	BnaA09g08950D	GroupIII	BnaC07g38550D
ERF_B-6	BnaA10g15390D			R2R3-MYB	BnaC05g51000D	V	BnaA09g08940D	GroupI	BnaC01g18810D
ERF_B-1	BnaC05g02080D			1R-MYB	BnaC06g25000D	V	BnaA02g26420D	GroupI	BnaC01g18490D
ERF_B-4	BnaA05g09970D			1R-MYB	BnaC01g01940D	V	BnaC02g34540D	GroupII-b	BnaC09g22830D
ERF_B-2	BnaC06g24360D			R2R3-MYB	BnaC03g15450D	V	BnaC09g21000D		
DREB_A-4	BnaC01g05620D			1R-MYB	BnaCnng62330D	V	BnaA09g19070D		
DREB_A-4	BnaC06g07710D			R2R3-MYB	BnaC08g31190D	V	BnaA06g34140D		
DREB_A-4	BnaC03g25050D			R2R3-MYB	BnaCnng31260D	V	BnaC07g21770D		
DREB_A-1	BnaC08g15650D			R2R3-MYB	BnaC07g31640D	V	BnaC03g54890D		
DREB_A-5	BnaC05g17200D			1R-MYB	BnaA01g32200D	V	BnaA06g39010D		
ERF_B-1	BnaA02g19610D			R2R3-MYB	BnaC01g31020D	V	BnaC03g53950D		
ERF_B-6	BnaC04g37670D			R2R3-MYB	BnaA03g39240D	V	BnaA06g19740D		
DREB_A-2	BnaC03g09040D			R2R3-MYB	BnaC06g19980D	V	BnaA02g05600D		
DREB_A-2	BnaA04g02310D			1R-MYB	BnaA10g29180D	V	BnaA10g13930D		
ERF_B-3	BnaC09g17590D			1R-MYB	BnaC09g33360D	V	BnaCnng60710D		
DREB_A-2	BnaA05g27930D			R2R3-MYB	BnaC08g18260D	V	BnaA03g08510D		
ERF_B-1	BnaC06g41440D			1R-MYB	BnaA03g31720D	IV	BnaA02g20430D		
ERF_B-5	BnaC07g38490D			R2R3-MYB	BnaA05g34070D	IV	BnaC02g27990D		
DREB_A-3	BnaCnng27900D			R2R3-MYB	BnaA01g31080D	IV	BnaA09g51610D		
DREB_A-2	BnaAnng09170D			R2R3-MYB	BnaA05g29680D	IV	BnaCnng01500D		
DREB_A-5	BnaA09g30360D			R2R3-MYB	BnaCnng17820D	IV	BnaA09g51620D		
ERF_B-3	BnaC08g44670D			R2R3-MYB	BnaA09g02150D	IV	BnaCnng01510D		
DREB_A-1	BnaA08g30950D			R2R3-MYB	BnaA06g05440D	IV	BnaA09g51630D		
ERF_B-6	BnaA09g04650D			R2R3-MYB	BnaC02g05740D	IV	BnaCnng01530D		
ERF_B-1	BnaA07g08450D			1R-MYB	BnaC09g34140D	IV	BnaC05g00890D		
ERF_B-6	BnaA02g32120D			R2R3-MYB	BnaAnng27960D	IV	BnaC05g00930D		
DREB_A-3	BnaA03g18970D			1R-MYB	BnaC05g46800D	IV	BnaA09g19800D		
DREB_A-6	BnaA07g20720D			1R-MYB	BnaC03g15160D	IV	BnaC01g28650D		
RAV	BnaA02g14040D			1R-MYB	BnaC06g36010D	IV	BnaC05g00910D		
AP2	BnaC07g16190D			R2R3-MYB	BnaC04g32560D	IV	BnaC05g00940D		
DREB_A-1	BnaUnng01150D			1R-MYB	BnaC05g50190D	IV	BnaA10g00830D		

ERF_B-1	BnaA08g18580D			1R-MYB	BnaC05g14070D	IV	BnaC05g00960D		
DREB_A-2	BnaA10g25000D			1R-MYB	BnaA09g38510D	IV	BnaA10g00860D		
ERF_B-3	BnaA02g25110D			R2R3-MYB	BnaA01g29790D	IV	BnaC06g15990D		
DREB_A-2	BnaC05g42130D			1R-MYB	BnaC07g00540D	IV	BnaC01g24910D		
DREB_A-6	BnaAnng37390D			R2R3-MYB	BnaA10g04120D	IV	BnaA09g35990D		
ERF_B-4	BnaA04g19700D			1R-MYB	BnaC09g33810D	IV	BnaC06g13440D		
DREB_A-4	BnaA09g38530D			R2R3-MYB	BnaC03g04220D	IV	BnaA07g15270D		
ERF_B-1	BnaC06g03860D			1R-MYB	BnaA07g38930D	IV	BnaC04g33200D		
DREB_A-6	BnaC05g27390D			1R-MYB	BnaA06g11070D	IV	BnaA04g10830D		
DREB_A-2	BnaC09g49920D			1R-MYB	BnaA08g22300D	IV	BnaC08g18550D		
DREB_A-6	BnaA02g18720D			1R-MYB	BnaC07g32760D	IV	BnaA03g04640D		
ERF	BnaA10g12950D			R2R3-MYB	BnaC09g44160D	IV	BnaC03g06170D		
ERF_B-3	BnaC06g42850D			R2R3-MYB	BnaCnng01390D	IV	BnaC09g43200D		
DREB_A-4	BnaC03g20180D			R2R3-MYB	BnaA10g19100D	IV	BnaA10g19630D		
ERF_B-6	BnaC04g37680D			1R-MYB	BnaA02g31210D	IV	BnaC03g58370D		
AP2	BnaC04g15640D			R2R3-MYB	BnaA06g28210D	IV	BnaA08g18520D		
DREB_A-2	BnaA03g31510D			1R-MYB	BnaA10g17370D	IV	BnaC08g49760D		
ERF_B-3	BnaA10g05780D			1R-MYB	BnaC03g08310D	IV	BnaA09g51470D		
ERF_B-3	BnaA10g30200D			R2R3-MYB	BnaC02g01090D	IV	BnaA09g51460D		
AP2	BnaA01g36720D			1R-MYB	BnaA04g19630D	IV	BnaC05g00990D		
ERF_B-5	BnaC01g15610D			R2R3-MYB	BnaA07g31220D	IV	BnaA10g00880D		
DREB_A-6	BnaC07g46940D			1R-MYB	BnaCnng41840D	IV	BnaC08g49770D		
DREB_A-3	BnaC03g72510D			R2R3-MYB	BnaC07g45320D	IV	BnaC05g01020D		
DREB_A-2	BnaC02g24390D			R2R3-MYB	BnaC06g35340D	IV	BnaA10g00910D		
DREB_A-4	BnaA10g07620D			1R-MYB	BnaC03g76670D	IV	BnaA10g00870D		
DREB_A-6	BnaA07g33930D			R2R3-MYB	BnaA06g24160D	IV	BnaC05g00970D		
ERF_B-6	BnaA02g27680D			R2R3-MYB	BnaC07g13600D	IV	BnaC05g01030D		
ERF_B-3	BnaA05g01500D			1R-MYB	BnaC03g39750D	IV	BnaC05g01000D		
DREB_A-4	BnaA09g12950D			3R-MYB	BnaC07g43900D	IV	BnaA10g00890D		
ERF_B-3	BnaAnng06940D			R2R3-MYB	BnaC09g41280D	IV	BnaC05g01040D		
DREB_A-1	BnaA09g10730D			R2R3-MYB	BnaA04g28770D	IV	BnaA10g00920D		
ERF_B-3	BnaA01g34910D			R2R3-MYB	BnaC05g40470D	III	BnaA07g05000D		
RAV	BnaA06g02510D			1R-MYB	BnaA01g02690D	III	BnaA03g19110D		
DREB_A-2	BnaA02g17250D			R2R3-MYB	BnaA05g27740D	III	BnaA09g28720D		
DREB_A-6	BnaC08g12880D			1R-MYB	BnaA07g22980D	III	BnaC05g20530D		
DREB_A-6	BnaC04g55700D			1R-MYB	BnaA08g21210D	III	BnaC07g11950D		
ERF_B-6	BnaC07g29370D			1R-MYB	BnaA05g00340D	III	BnaA07g37110D		
DREB_A-4	BnaC05g00680D			R2R3-MYB	BnaA09g28450D	III	BnaA03g04920D		
ERF_B-1	BnaC03g69420D			1R-MYB	BnaC09g29440D	III	BnaA03g27400D		
AP2	BnaC02g11520D			R2R3-MYB	BnaC03g01730D	III	BnaC03g32420D		
DREB_A-4	BnaA03g42840D			R2R3-MYB	BnaC03g07960D	III	BnaA07g16830D		
ERF	BnaA03g02990D			1R-MYB	BnaAnng17890D	III	BnaA03g49460D		
ERF_B-2	BnaA03g33290D			R2R3-MYB	BnaA05g10900D	III	BnaC07g41850D		
RAV	BnaA06g33420D			1R-MYB	BnaC03g00040D	III	BnaC01g09270D		
ERF_B-2	BnaC03g38390D			1R-MYB	BnaA01g00920D	III	BnaA01g07600D		
ERF_B-4	BnaC02g04660D			1R-MYB	BnaCnng35740D	III	BnaAnng19940D		
DREB_A-4	BnaC07g29050D			R2R3-MYB	BnaA03g02860D	III	BnaC09g33190D		
ERF	BnaA03g19580D			R2R3-MYB	BnaCnng76050D	III	BnaAnng33660D		
DREB_A-5	BnaC04g35800D			3R-MYB	BnaC05g43530D	III	BnaC05g21870D		
ERF_B-1	BnaC05g31720D			1R-MYB	BnaC08g30340D	III	BnaA07g08370D		
DREB_A-4	BnaCnng48950D			1R-MYB	BnaA05g01400D	III	BnaC07g10350D		
DREB_A-4	BnaC09g50850D			1R-MYB	BnaC08g23560D	III	BnaA03g49310D		
ERF_B-5	BnaCnng17120D			1R-MYB	BnaC05g00840D	III	BnaC07g41360D		
ERF_B-3	BnaA04g25900D			R2R3-MYB	BnaC08g48630D	III	BnaA01g08370D		
ERF_B-2	BnaC05g36570D			R2R3-MYB	BnaA02g29620D	III	BnaC01g09920D		
ERF_B-6	BnaA02g04480D			R2R3-MYB	BnaC04g27380D	II	BnaA09g00360D		
DREB_A-6	BnaC03g60650D			1R-MYB	BnaC02g27500D	II	BnaCnng01350D		
ERF_B-1	BnaA10g02160D			1R-MYB	BnaAnng39410D	II	BnaC03g31420D		
DREB_A-4	BnaC09g51170D			R2R3-MYB	BnaC03g55560D	II	BnaA09g00430D		
ERF_B-2	BnaA07g23090D			1R-MYB	BnaA03g21400D	II	BnaA09g00440D		
ERF_B-1	BnaC02g08170D			R2R3-MYB	BnaA09g39030D	II	BnaCnng01250D		
ERF_B-1	BnaC03g39000D			R2R3-MYB	BnaA09g06090D	II	BnaCnng35260D		
ERF_B-3	BnaAnng12120D			R2R3-MYB	BnaC04g08340D	II	BnaA05g13300D		
ERF_B-4	BnaC03g05820D			3R-MYB	BnaC03g66940D	II	BnaCnng55820D		
ERF_B-4	BnaA02g34370D			1R-MYB	BnaC01g22100D	II	BnaA05g13270D		
ERF_B-1	BnaCnng67070D			R2R3-MYB	BnaA05g27620D	II	BnaCnng43170D		
DREB_A-5	BnaC08g04820D			3R-MYB	BnaA03g03390D	II	BnaC04g01390D		
ERF_B-3	BnaC04g49870D			R2R3-MYB	BnaA08g22580D	II	BnaC04g01450D		
ERF_B-3	BnaA03g52830D			R2R3-MYB	BnaC03g55550D	II	BnaA05g01770D		
DREB_A-2	BnaA03g55280D			1R-MYB	BnaC09g01550D	II	BnaA05g01730D		
ERF_B-2	BnaC05g39400D			1R-MYB	BnaA05g28870D	II	BnaA10g11790D		
ERF_B-3	BnaA01g23960D			1R-MYB	BnaA01g21650D	II	BnaC09g34230D		
ERF	BnaCnng60480D			R2R3-MYB	BnaAnng02150D	II	BnaC09g34220D		
DREB_A-4	BnaC04g08850D			R2R3-MYB	BnaA01g21090D	II	BnaC02g10100D		
ERF	BnaC03g26480D			1R-MYB	BnaA06g37640D	II	BnaC02g10340D		
RAV	BnaAnng40580D			1R-MYB	BnaA03g34320D	II	BnaC02g10310D		
ERF_B-3	BnaC07g31350D			1R-MYB	BnaA09g07290D	II	BnaC03g51020D		

ERF_B-6	BnaC05g11660D			1R-MYB	BnaC08g36920D	II	BnaC09g18980D		
DREB_A-4	BnaA01g18170D			R2R3-MYB	BnaA03g04050D	II	BnaA01g22100D		
AP2	BnaA08g15790D			1R-MYB	BnaAnng38130D	II	BnaA01g22280D		
ERF_B-5	BnaCnng38100D			1R-MYB	BnaC08g28910D	II	BnaC01g43190D		
DREB_A-5	BnaA07g29400D			R2R3-MYB	BnaAnng07760D	II	BnaC01g43150D		
ERF_B-1	BnaCnng63180D			R2R3-MYB	BnaA05g27610D	II	BnaCnng48900D		
DREB_A-4	BnaA03g20950D			1R-MYB	BnaA03g12400D	II	BnaA09g19790D		
DREB_A-4	BnaC09g28540D			R2R3-MYB	BnaA06g29540D	II	BnaA10g10080D		
ERF_B-3	BnaC07g20040D			1R-MYB	BnaC05g36490D	II	BnaCnng32040D		
ERF_B-5	BnaC08g12710D			1R-MYB	BnaA08g22290D	II	BnaC09g32530D		
DREB_A-4	BnaA07g23500D			R2R3-MYB	BnaC03g08600D	II	BnaA10g10100D		
ERF_B-1	BnaC09g39250D			R2R3-MYB	BnaA03g39790D	II	BnaA06g25650D		
DREB_A-5	BnaA04g13590D			1R-MYB	BnaA09g44970D	II	BnaA10g10240D		
DREB_A-5	BnaA01g06090D			R2R3-MYB	BnaC08g26660D	I	BnaAnng36380D		
ERF_B-1	BnaC07g11010D			R2R3-MYB	BnaC05g14250D	I	BnaC05g07680D		
AP2	BnaA09g34250D			R2R3-MYB	BnaCnng46710D	I	BnaA04g02600D		
ERF_B-3	BnaA08g08300D			R2R3-MYB	BnaCnng44540D				
ERF_B-1	BnaA03g07180D			R2R3-MYB	BnaA03g30840D				
DREB_A-6	BnaC01g20660D			R2R3-MYB	BnaCnng13370D				
DREB_A-2	BnaA09g36920D			R2R3-MYB	BnaC04g41990D				
DREB_A-6	BnaA03g54460D			R2R3-MYB	BnaCnng06440D				
AP2	BnaC03g61270D			R2R3-MYB	BnaC03g43590D				
DREB_A-2	BnaC06g43980D			R2R3-MYB	BnaC04g05500D				
ERF_B-3	BnaAnng07060D			1R-MYB	BnaC08g30630D				
ERF_B-1	BnaA07g08610D			1R-MYB	BnaCnng08670D				
ERF_B-3	BnaC01g10100D			1R-MYB	BnaA09g11280D				
DREB_A-6	BnaA08g16220D			1R-MYB	BnaA01g17750D				
AP2	BnaC06g40040D			1R-MYB	BnaA09g00080D				
DREB_A-6	BnaC02g43540D			1R-MYB	BnaC07g31550D				
ERF_B-1	BnaA09g27310D			R2R3-MYB	BnaC07g08510D				
ERF_B-1	BnaA08g18380D			R2R3-MYB	BnaC03g36160D				
DREB_A-1	BnaC07g39680D			R2R3-MYB	BnaC03g28550D				
ERF_B-3	BnaA09g18210D			1R-MYB	BnaC01g00270D				
ERF_B-6	BnaA09g28560D			R2R3-MYB	BnaA03g58600D				
ERF_B-1	BnaA02g01920D			R2R3-MYB	BnaA06g31220D				
ERF_B-3	BnaAnng40920D			R2R3-MYB	BnaC09g02180D				
DREB_A-2	BnaA02g35180D			R2R3-MYB	BnaA02g35530D				
DREB_A-2	BnaA01g31290D			3R-MYB	BnaC01g05660D				
RAV	BnaC05g20560D			1R-MYB	BnaA07g31770D				
ERF_B-3	BnaC09g05370D			1R-MYB	BnaC07g33430D				
ERF_B-1	BnaA08g18390D			R2R3-MYB	BnaAnng32120D				
ERF_B-1	BnaC03g58300D			R2R3-MYB	BnaCnng28030D				
ERF_B-3	BnaA10g27750D			1R-MYB	BnaC03g16910D				
DREB_A-5	BnaC08g18660D			R2R3-MYB	BnaA07g38030D				
ERF_B-3	BnaC02g48800D			R2R3-MYB	BnaC04g32550D				
DREB_A-6	BnaC07g50590D			R2R3-MYB	BnaC02g03720D				
ERF_B-3	BnaC04g25670D			R2R3-MYB	BnaA07g25720D				
AP2	BnaC05g12270D			R2R3-MYB	BnaA09g48440D				
DREB_A-1	BnaA08g30910D			R2R3-MYB	BnaC05g48910D				
ERF_B-3	BnaC05g02480D			R2R3-MYB	BnaA03g24010D				
DREB_A-6	BnaA03g49050D			R2R3-MYB	BnaC05g06410D				
DREB_A-5	BnaC06g38250D			R2R3-MYB	BnaC08g37160D				
ERF_B-3	BnaAnng21280D			R2R3-MYB	BnaA02g30960D				
DREB_A-6	BnaC01g00660D			1R-MYB	BnaA08g11190D				
DREB_A-6	BnaA01g16910D			1R-MYB	BnaA02g17000D				
DREB_A-5	BnaA08g04090D			1R-MYB	BnaA07g00280D				
DREB_A-5	BnaC08g36830D			R2R3-MYB	BnaC04g32680D				
AP2	BnaC07g46770D			1R-MYB	BnaA04g29720D				
DREB_A-5	BnaC06g32600D			R2R3-MYB	BnaA01g30300D				
DREB_A-5	BnaAnng03730D			R2R3-MYB	BnaC03g06900D				
DREB_A-6	BnaC06g38580D			R2R3-MYB	BnaA02g00440D				
ERF_B-1	BnaA03g04570D			1R-MYB	BnaC09g02330D				
ERF_B-1	BnaA03g35680D			R2R3-MYB	BnaCnng63810D				
DREB_A-5	BnaCnng39660D			R2R3-MYB	BnaC03g71630D				
ERF_B-1	BnaA06g01090D			R2R3-MYB	BnaA01g23930D				
ERF_B-5	BnaA08g20700D			R2R3-MYB	BnaA01g30350D				
ERF_B-3	BnaA05g11530D			R2R3-MYB	BnaA07g31680D				
DREB_A-4	BnaAnng41450D			R2R3-MYB	BnaA07g31550D				
ERF_B-4	BnaA10g19960D			1R-MYB	BnaA03g06490D				
ERF_B-1	BnaC07g10670D			R2R3-MYB	BnaAnng35730D				
RAV	BnaA02g27630D			1R-MYB	BnaA10g28420D				
ERF_B-1	BnaA09g27330D			R2R3-MYB	BnaC04g45680D				
DREB_A-4	BnaA06g27900D			R2R3-MYB	BnaC08g18080D				
ERF_B-2	BnaA04g27050D			R2R3-MYB	BnaCnng28250D				
ERF_B-3	BnaCnng36390D			1R-MYB	BnaA09g18280D				
ERF_B-3	BnaA03g40370D			1R-MYB	BnaA03g00640D				
ERF_B-4	BnaC02g01680D			R2R3-MYB	BnaA03g27440D				

DREB_A-4	BnaA03g16280D			R2R3-MYB	BnaA08g19860D				
ERF_B-2	BnaA05g25200D			1R-MYB	BnaA01g08890D				
ERF_B-5	BnaAnng36370D			R2R3-MYB	BnaC04g43020D				
ERF	BnaC09g40510D			R2R3-MYB	BnaC07g39760D				
ERF_B-6	BnaC03g09750D			1R-MYB	BnaCnng47140D				
ERF_B-5	BnaA08g13940D			3R-MYB	BnaA01g04220D				
DREB_A-5	BnaA10g27820D			1R-MYB	BnaAnng06930D				
AP2	BnaC01g01290D			R2R3-MYB	BnaA03g37280D				
RAV	BnaA08g02030D			R2R3-MYB	BnaCnng69610D				
DREB_A-2	BnaC09g39350D			1R-MYB	BnaC01g21680D				
ERF_B-6	BnaA02g13950D			1R-MYB	BnaC04g03690D				
ERF_B-6	BnaC07g23080D			1R-MYB	BnaC04g01230D				
DREB_A-4	BnaC07g43950D			R2R3-MYB	BnaA03g04840D				
ERF_B-5	BnaC03g14960D			R2R3-MYB	BnaA05g00710D				
ERF_B-3	BnaC04g13510D			R2R3-MYB	BnaC02g22880D				
DREB_A-4	BnaCnng08620D			R2R3-MYB	BnaA06g38670D				
DREB_A-5	BnaA02g15410D			R2R3-MYB	BnaA05g05710D				
DREB_A-4	BnaC04g09380D			R2R3-MYB	BnaA02g33410D				
ERF_B-3	BnaC04g53270D			1R-MYB	BnaA10g11980D				
RAV	BnaC02g35650D			R2R3-MYB	BnaA05g26450D				
DREB_A-5	BnaA01g00710D			1R-MYB	BnaAnng34960D				
AP2	BnaA03g53830D			1R-MYB	BnaC09g40660D				
DREB_A-5	BnaAnng29870D			1R-MYB	BnaA05g37380D				
AP2	BnaC08g25150D			R2R3-MYB	BnaC09g42790D				
ERF_B-1	BnaA07g08440D			R2R3-MYB	BnaC03g20340D				
DREB_A-6	BnaAnng09570D			3R-MYB	BnaC01g12000D				
DREB_A-4	BnaA03g56380D			R2R3-MYB	BnaC03g21050D				
ERF_B-6	BnaC02g40810D			R2R3-MYB	BnaA03g30340D				
DREB_A-6	BnaC04g34610D			1R-MYB	BnaA10g00430D				
ERF_B-6	BnaA04g14840D			R2R3-MYB	BnaC08g07960D				
DREB_A-4	BnaCnng07950D			R2R3-MYB	BnaA10g10250D				
DREB_A-2	BnaA04g29310D			R2R3-MYB	BnaA03g12690D				
AP2	BnaA07g35130D			R2R3-MYB	BnaA09g44780D				
ERF	BnaA05g02350D			1R-MYB	BnaC07g03440D				
ERF_B-5	BnaC03g29310D			R2R3-MYB	BnaC07g25120D				
ERF_B-6	BnaA09g04580D			1R-MYB	BnaC08g10700D				
ERF_B-1	BnaCnng78540D			R2R3-MYB	BnaA10g19370D				
RAV	BnaCnng33070D			R2R3-MYB	BnaC07g25960D				
ERF	BnaC06g15540D			1R-MYB	BnaC06g32180D				
ERF_B-3	BnaAnng06420D			1R-MYB	BnaC05g44230D				
ERF_B-1	BnaC05g38410D			R2R3-MYB	BnaA06g12690D				
ERF_B-4	BnaA06g23710D			R2R3-MYB	BnaC06g23040D				
ERF_B-6	BnaC06g25810D			R2R3-MYB	BnaC05g22860D				
DREB_A-4	BnaC07g33940D			R2R3-MYB	BnaC07g28680D				
DREB_A-5	BnaA07g31860D			R2R3-MYB	BnaC05g04250D				
ERF_B-5	BnaA02g35550D			1R-MYB	BnaA07g36940D				
ERF_B-3	BnaC09g20090D			R2R3-MYB	BnaC01g26440D				
ERF_B-2	BnaA01g27570D			1R-MYB	BnaA06g12480D				
DREB_A-1	BnaCnng31550D			1R-MYB	BnaCnng59200D				
ERF	BnaA10g29740D			R2R3-MYB	BnaC03g64160D				
ERF_B-4	BnaC09g48530D			R2R3-MYB	BnaC07g27140D				
ERF_B-5	BnaC07g13400D			1R-MYB	BnaA06g05610D				
RAV	BnaC07g22870D			1R-MYB	BnaC08g32380D				
DREB_A-4	BnaA09g10750D			R2R3-MYB	BnaA08g26320D				
ERF_B-4	BnaC09g06610D			1R-MYB	BnaA01g34510D				
DREB_A-5	BnaC06g35730D			1R-MYB	BnaC08g17410D				
DREB_A-5	BnaA07g10270D			1R-MYB	BnaC07g33650D				
DREB_A-4	BnaA01g04150D			1R-MYB	BnaC09g15550D				
ERF_B-3	BnaA05g04380D			1R-MYB	BnaCnng62030D				
				R2R3-MYB	BnaC03g71080D				
				R2R3-MYB	BnaCnng06970D				
				1R-MYB	BnaA03g52170D				
				R2R3-MYB	BnaC05g41910D				
				R2R3-MYB	BnaC09g35120D				
				3R-MYB	BnaAnng13080D				
				1R-MYB	BnaCnng72020D				
				R2R3-MYB	BnaA08g00060D				
				R2R3-MYB	BnaA03g11930D				
				R2R3-MYB	BnaAnng19460D				
				R2R3-MYB	BnaA02g03700D				
				1R-MYB	BnaA05g29910D				
				1R-MYB	BnaC03g15430D				
				R2R3-MYB	BnaA07g06740D				
				1R-MYB	BnaAnng35540D				
				1R-MYB	BnaA09g10270D				
				R2R3-MYB	BnaA03g21790D				
				R2R3-MYB	BnaC03g11590D				

				1R-MYB	BnaA02g07870D				
				1R-MYB	BnaA07g32000D				
				R2R3-MYB	BnaCnng16520D				
				1R-MYB	BnaA06g28660D				
				R2R3-MYB	BnaC09g49710D				
				1R-MYB	BnaC05g36990D				
				R2R3-MYB	BnaA08g22850D				
				R2R3-MYB	BnaA03g09270D				
				1R-MYB	BnaA03g26820D				
				R2R3-MYB	BnaA07g12970D				
				1R-MYB	BnaA07g29070D				
				R2R3-MYB	BnaC02g04390D				
				1R-MYB	BnaCnng59970D				
				R2R3-MYB	BnaA08g26750D				
				1R-MYB	BnaC05g35410D				
				1R-MYB	BnaA10g23980D				
				R2R3-MYB	BnaAnng06040D				
				R2R3-MYB	BnaA03g55780D				
				R2R3-MYB	BnaA03g11630D				
				R2R3-MYB	BnaA07g05740D				
				R2R3-MYB	BnaA05g07510D				
				R2R3-MYB	BnaC09g42570D				
				1R-MYB	BnaC09g29430D				
				1R-MYB	BnaC03g12320D				
				1R-MYB	BnaC05g17030D				
				R2R3-MYB	BnaA01g12370D				
				1R-MYB	BnaA06g25300D				
				1R-MYB	BnaC01g03950D				
				R2R3-MYB	BnaA01g32800D				
				R2R3-MYB	BnaA10g17890D				
				R2R3-MYB	BnaA05g33770D				
				R2R3-MYB	BnaA02g02580D				
				R2R3-MYB	BnaA06g29370D				
				1R-MYB	BnaA03g34140D				
				1R-MYB	BnaA01g16760D				
				1R-MYB	BnaA03g29470D				
				1R-MYB	BnaC01g27820D				
				R2R3-MYB	BnaA06g12860D				
				R2R3-MYB	BnaCnng58140D				
				R2R3-MYB	BnaAnng15260D				
				1R-MYB	BnaC09g50770D				
				1R-MYB	BnaC03g48330D				
				R2R3-MYB	BnaC04g35730D				
				R2R3-MYB	BnaA03g01420D				
				1R-MYB	BnaCnng01850D				
				1R-MYB	BnaAnng21440D				
				1R-MYB	BnaC08g03980D				
				R2R3-MYB	BnaC08g42690D				
				R2R3-MYB	BnaC01g01660D				
				1R-MYB	BnaA10g00780D				
				R2R3-MYB	BnaCnng52970D				
				R2R3-MYB	BnaA03g06190D				
				R2R3-MYB	BnaCnng01000D				
				R2R3-MYB	BnaA08g16990D				
				1R-MYB	BnaC07g30660D				
				R2R3-MYB	BnaC09g41070D				
				1R-MYB	BnaA02g36430D				
				R2R3-MYB	BnaCnng49790D				
				R2R3-MYB	BnaA03g47510D				
				R2R3-MYB	BnaC03g12860D				
				R2R3-MYB	BnaA03g53870D				
				1R-MYB	BnaA09g40030D				
				R2R3-MYB	BnaA10g12770D				
				R2R3-MYB	BnaC07g27330D				
				R2R3-MYB	BnaA07g20440D				
				1R-MYB	BnaUnng04070D				
				R2R3-MYB	BnaC09g11720D				
				R2R3-MYB	BnaA07g31540D				
				R2R3-MYB	BnaC05g17910D				
				1R-MYB	BnaA03g41680D				
				R2R3-MYB	BnaC02g05270D				
				R2R3-MYB	BnaA09g33480D				
				R2R3-MYB	BnaC03g06410D				
				R2R3-MYB	BnaC01g37360D				
				R2R3-MYB	BnaC09g12530D				
				1R-MYB	BnaCnng52990D				
				R2R3-MYB	BnaCnng51960D				

				1R-MYB	BnaC05g42410D				
				R2R3-MYB	BnaC05g44010D				
				R2R3-MYB	BnaA02g16690D				
				R2R3-MYB	BnaA01g16900D				
				1R-MYB	BnaC09g54190D				
				1R-MYB	BnaC04g22430D				
				R2R3-MYB	BnaC04g31750D				
				1R-MYB	BnaA10g26900D				
				R2R3-MYB	BnaA03g14350D				
				1R-MYB	BnaC05g07150D				
				1R-MYB	BnaC01g26310D				
				1R-MYB	BnaA08g23070D				
				R2R3-MYB	BnaC02g43910D				
				R2R3-MYB	BnaA07g22240D				
				1R-MYB	BnaA05g36000D				
				R2R3-MYB	BnaC02g44890D				
				1R-MYB	BnaA07g02020D				
				R2R3-MYB	BnaCnng75540D				
				1R-MYB	BnaA03g42350D				
				1R-MYB	BnaA09g14920D				
				R2R3-MYB	BnaCnng49840D				
				R2R3-MYB	BnaA07g18670D				
				1R-MYB	BnaA06g24950D				
				R2R3-MYB	BnaA06g31780D				
				R2R3-MYB	BnaC09g31560D				
				R2R3-MYB	BnaA01g30920D				
				R2R3-MYB	BnaC09g40110D				
				1R-MYB	BnaA07g24010D				
				R2R3-MYB	BnaC07g16030D				
				R2R3-MYB	BnaC06g35350D				
				1R-MYB	BnaC03g31750D				
				R2R3-MYB	BnaA10g21970D				
				R2R3-MYB	BnaAnng13560D				
				3R-MYB	BnaCnng03520D				
				R2R3-MYB	BnaAnng26800D				
				R2R3-MYB	BnaC01g03510D				
				R2R3-MYB	BnaC09g02660D				
				R2R3-MYB	BnaC06g22860D				
				1R-MYB	BnaAnng01040D				
				1R-MYB	BnaC07g28070D				
				R2R3-MYB	BnaAnng32620D				
				R2R3-MYB	BnaA01g08670D				
				R2R3-MYB	BnaC08g25380D				
				R2R3-MYB	BnaC02g40390D				
				1R-MYB	BnaC03g68390D				
				1R-MYB	BnaAnng25220D				
				R2R3-MYB	BnaA10g30020D				
				R2R3-MYB	BnaCnng17910D				
				R2R3-MYB	BnaC05g48630D				
				1R-MYB	BnaA01g05400D				
				1R-MYB	BnaC03g66110D				
				R2R3-MYB	BnaC02g39260D				
				1R-MYB	BnaC04g50810D				
				1R-MYB	BnaA03g10580D				
				R2R3-MYB	BnaA04g10420D				
				R2R3-MYB	BnaA05g37010D				
				1R-MYB	BnaC05g43350D				
				R2R3-MYB	BnaC03g03620D				
				R2R3-MYB	BnaC03g03210D				
				1R-MYB	BnaAnng15540D				
				R2R3-MYB	BnaA01g14800D				
				R2R3-MYB	BnaC06g08330D				
				R2R3-MYB	BnaA10g17700D				
				R2R3-MYB	BnaC04g52010D				
				R2R3-MYB	BnaA03g40190D				
				R2R3-MYB	BnaA03g12580D				
				R2R3-MYB	BnaC04g12330D				
				1R-MYB	BnaC04g00590D				
				R2R3-MYB	BnaCnng49340D				
				R2R3-MYB	BnaA07g28100D				
				R2R3-MYB	BnaA09g35310D				
				1R-MYB	BnaA03g42550D				
				R2R3-MYB	BnaC07g25380D				
				1R-MYB	BnaA09g00230D				
				1R-MYB	BnaC08g30650D				
				R2R3-MYB	BnaA09g52130D				
				R2R3-MYB	BnaC09g36060D				

				R2R3-MYB	BnaC01g14080D				
				1R-MYB	BnaA01g21010D				
				1R-MYB	BnaA09g37190D				
				R2R3-MYB	BnaA05g25530D				
				R2R3-MYB	BnaC07g08320D				
				1R-MYB	BnaCnng03200D				
				R2R3-MYB	BnaC03g15570D				
				R2R3-MYB	BnaA06g31470D				
				R2R3-MYB	BnaCnng28540D				
				4R-MYB	BnaA05g22120D				
				R2R3-MYB	BnaC04g51450D				
				1R-MYB	BnaC04g08290D				
				1R-MYB	BnaA03g40600D				
				R2R3-MYB	BnaAnng13960D				
				1R-MYB	BnaCnng02030D				
				R2R3-MYB	BnaC03g60080D				
				R2R3-MYB	BnaCnng69750D				
				R2R3-MYB	BnaA07g10350D				
				R2R3-MYB	BnaA07g22130D				
				1R-MYB	BnaA06g25640D				
				1R-MYB	BnaC06g23020D				
				1R-MYB	BnaCnng66770D				
				1R-MYB	BnaC05g00500D				
				R2R3-MYB	BnaA06g31230D				
				R2R3-MYB	BnaA07g16010D				
				R2R3-MYB	BnaC05g39780D				
				1R-MYB	BnaCnng61080D				
				R2R3-MYB	BnaC08g37480D				
				1R-MYB	BnaC02g38120D				
				R2R3-MYB	BnaA02g31750D				
				1R-MYB	BnaC05g13640D				
The identified five families of TFs in B. rapa									
Subfamily	BrAP2/EREBP	Subfami	BrbZIP	Subfamily	BrMYB	Subfamily	BrNAC	Subfamily	BrWRKY
AP2	Bra017852	IX	Bra037533	3R-MYB	Bra011376	XXI	Bra025374	GroupII-d	Bra011282
RAV	Bra018894	IX	Bra001742	1R-MYB	Bra004539	XXI	Bra036223	GroupII-b	Bra011299
DREB_A-5	Bra024539	IX	Bra022409	R2R3-MYB	Bra032176	XXI	Bra025658	GroupII-c	Bra011861
ERF_B-3	Bra027493	IX	Bra018800	1R-MYB	Bra016164	XXI	Bra028435	GroupII-c	Bra013289
ERF_B-3	Bra017493	IX	Bra011485	1R-MYB	Bra032635	XX	Bra028685	GroupII-b	Bra013584
ERF_B-3	Bra021712	IX	Bra040260	R2R3-MYB	Bra012910	XX	Bra009246	GroupII-e	Bra013708
DREB_A-2	Bra023674	IX	Bra016953	R2R3-MYB	Bra036317	XX	Bra029313	GroupIII	Bra013732
DREB_A-4	Bra029147	IX	Bra004597	1R-MYB	Bra007892	XX	Bra012960	GroupII-d	Bra013779
DREB_A-6	Bra010657	IX	Bra003253	1R-MYB	Bra025967	XIX	Bra040153	GroupII-e	Bra026228
ERF_B-4	Bra019622	IX	Bra014668	1R-MYB	Bra020373	XIX	Bra031950	GroupI	Bra026414
ERF_B-4	Bra005480	IX	Bra007274	R2R3-MYB	Bra039344	XIX	Bra002148	GroupI	Bra026438
ERF_B-1	Bra010901	IX	Bra007276	1R-MYB	Bra037839	XIX	Bra023669	GroupII-b	Bra040505
DREB_A-5	Bra016518	IX	Bra019436	1R-MYB	Bra019998	XIX	Bra006449	GroupII-b	Bra007884
DREB_A-5	Bra040309	IX	Bra017251	R2R3-MYB	Bra029990	XVIII	Bra021592	GroupII-b	Bra008435
ERF_B-2	Bra022176	IX	Bra005287	1R-MYB	Bra027170	XVIII	Bra001586	GroupIII	Bra008454
AP2	Bra028584	IX	Bra033649	1R-MYB	Bra034130	XVIII	Bra022685	GroupIII	Bra008459
AP2	Bra030451	VIII	Bra015281	1R-MYB	Bra002145	XVIII	Bra003023	GroupIII	Bra020196
DREB_A-4	Bra007539	VIII	Bra033719	R2R3-MYB	Bra004394	XVII	Bra008259	GroupIII	Bra020197
DREB_A-6	Bra031880	VII	Bra013005	1R-MYB	Bra003871	XVII	Bra015750	GroupII-c	Bra020546
DREB_A-4	Bra008952	VII	Bra035957	1R-MYB	Bra037045	XVII	Bra001705	GroupII-d	Bra020617
ERF_B-1	Bra036016	VII	Bra029353	R2R3-MYB	Bra036202	XVII	Bra037585	GroupII-c	Bra020628
DREB_A-4	Bra023026	VII	Bra005971	R2R3-MYB	Bra024224	XVII	Bra022319	GroupII-c	Bra022033
DREB_A-5	Bra003780	VII	Bra025743	R2R3-MYB	Bra021033	XVI	Bra007855	GroupII-e	Bra022612
ERF_B-6	Bra004318	VII	Bra038341	1R-MYB	Bra004503	XVI	Bra032095	GroupII-b	Bra023505
ERF_B-5	Bra003936	VII	Bra026895	1R-MYB	Bra029380	XV	Bra027237	GroupI	Bra026554
ERF_B-6	Bra009837	VII	Bra026896	R2R3-MYB	Bra034738	XV	Bra021114	GroupII-c	Bra028707
ERF_B-3	Bra027447	VII	Bra019715	3R-MYB	Bra008956	XV	Bra001597	GroupIII	Bra028918
DREB_A-2	Bra008200	VII	Bra026523	3R-MYB	Bra034531	XV	Bra018997	GroupII-c	Bra031900
DREB_A-4	Bra012693	VII	Bra017359	1R-MYB	Bra009245	XV	Bra027101	GroupIII	Bra033158
DREB_A-2	Bra028759	VII	Bra025144	1R-MYB	Bra025973	XV	Bra003998	GroupII-b	Bra033956
DREB_A-2	Bra015840	VII	Bra025418	R2R3-MYB	Bra023742	XV	Bra004385	GroupII-c	Bra038313
DREB_A-4	Bra033274	VII	Bra020735	1R-MYB	Bra009950	XV	Bra040152	GroupI	Bra000064
DREB_A-2	Bra006460	VII	Bra008670	R2R3-MYB	Bra013000	XV	Bra031949	GroupIII	Bra000202
RAV	Bra014282	VII	Bra023540	R2R3-MYB	Bra007186	XV	Bra027238	GroupIII	Bra000203
RAV	Bra024735	VII	Bra006324	R2R3-MYB	Bra001941	XV	Bra001596	GroupII-c	Bra000362
DREB_A-6	Bra026280	VI	Bra039631	1R-MYB	Bra008169	XV	Bra021113	GroupIII	Bra000423
ERF_B-5	Bra003068	VI	Bra024478	R2R3-MYB	Bra008875	XV	Bra018998	GroupII-b	Bra000793
ERF_B-6	Bra020063	VI	Bra037235	1R-MYB	Bra004831	XV	Bra026353	GroupII-c	Bra001023
ERF	Bra007123	VI	Bra034639	R2R3-MYB	Bra003443	XV	Bra019052	GroupII-d	Bra001117
DREB_A-4	Bra017302	VI	Bra011545	1R-MYB	Bra024438	XV	Bra033239	GroupII-c	Bra006178
ERF_B-3	Bra012939	VI	Bra017664	1R-MYB	Bra011533	XV	Bra008309	GroupII-b	Bra006283
DREB_A-6	Bra024358	VI	Bra015847	R2R3-MYB	Bra006694	XV	Bra029201	GroupII-c	Bra012614
DREB_A-6	Bra030255	VI	Bra008192	1R-MYB	Bra033527	XV	Bra038663	GroupI	Bra013187
ERF_B-4	Bra023404	VI	Bra003755	1R-MYB	Bra008539	XV	Bra028645	GroupII-c	Bra019095

DREB_A-2	Bra005113	VI	Bra003500	1R-MYB	Bra014227	XV	Bra006015	GroupI	Bra019105
ERF_B-3	Bra014924	VI	Bra007679	1R-MYB	Bra039283	XV	Bra009370	GroupII-b	Bra019123
DREB_A-4	Bra037041	VI	Bra011780	R2R3-MYB	Bra021389	XIV	Bra031915	GroupII-d	Bra019239
DREB_A-5	Bra006599	VI	Bra017850	1R-MYB	Bra033257	XIV	Bra037779	GroupIII	Bra019265
DREB_A-4	Bra016136	VI	Bra036025	R2R3-MYB	Bra008096	XIV	Bra024306	GroupII-e	Bra019297
DREB_A-4	Bra017235	VI	Bra031845	R2R3-MYB	Bra000531	XIV	Bra021856	GroupI	Bra022786
ERF_B-1	Bra030957	VI	Bra024424	R2R3-MYB	Bra024526	XIV	Bra005492	GroupII-d	Bra022808
ERF_B-5	Bra007963	VI	Bra027855	R2R3-MYB	Bra030743	XIV	Bra006186	GroupI	Bra023112
DREB_A-5	Bra036061	VI	Bra010035	R2R3-MYB	Bra030812	XIV	Bra023395	GroupII-b	Bra023983
ERF_B-6	Bra019086	VI	Bra036142	R2R3-MYB	Bra028369	XIV	Bra008849	GroupII-d	Bra023998
ERF_B-5	Bra010389	VI	Bra004550	R2R3-MYB	Bra023085	XIII	Bra030820	GroupI	Bra024035
DREB_A-4	Bra027177	VI	Bra037382	R2R3-MYB	Bra029258	XIII	Bra001480	GroupI	Bra028939
DREB_A-1	Bra019777	VI	Bra011701	R2R3-MYB	Bra039108	XIII	Bra034713	GroupI	Bra028971
AP2	Bra011939	VI	Bra010572	R2R3-MYB	Bra035954	XIII	Bra010362	GroupIII	Bra014426
AP2	Bra034249	VI	Bra017316	R2R3-MYB	Bra004456	XIII	Bra011037	GroupII-e	Bra014556
ERF_B-4	Bra037794	VI	Bra023243	R2R3-MYB	Bra020837	XIII	Bra024194	GroupIII	Bra014692
DREB_A-4	Bra009862	VI	Bra023012	R2R3-MYB	Bra039036	XIII	Bra000709	GroupIII	Bra014693
DREB_A-2	Bra014617	VI	Bra005335	1R-MYB	Bra012936	XIII	Bra037914	GroupIII	Bra016974
DREB_A-5	Bra017811	V	Bra020471	R2R3-MYB	Bra023486	XIII	Bra008423	GroupIII	Bra016975
ERF_B-4	Bra029302	V	Bra009793	R2R3-MYB	Bra018027	XIII	Bra035127	GroupI	Bra017117
ERF_B-3	Bra023748	V	Bra020620	R2R3-MYB	Bra031035	XIII	Bra003602	GroupII-c	Bra021393
AP2	Bra008040	V	Bra010010	R2R3-MYB	Bra005597	XIII	Bra010077	GroupI	Bra021623
DREB_A-6	Bra003659	V	Bra036251	3R-MYB	Bra006102	XIII	Bra029264	GroupII-d	Bra021652
DREB_A-5	Bra010258	V	Bra000102	R2R3-MYB	Bra003413	XIII	Bra029266	GroupII-e	Bra021934
ERF	Bra010610	V	Bra014802	R2R3-MYB	Bra013633	XIII	Bra035896	GroupII-c	Bra025490
DREB_A-5	Bra025719	V	Bra007083	1R-MYB	Bra000456	XIII	Bra004509	GroupII-c	Bra030273
ERF_B-6	Bra036543	IV	Bra008976	1R-MYB	Bra000941	XIII	Bra040486	GroupII-d	Bra032084
DREB_A-5	Bra036022	IV	Bra023317	R2R3-MYB	Bra026048	XIII	Bra003478	GroupII-d	Bra032174
RAV	Bra026509	IV	Bra021258	R2R3-MYB	Bra039339	XIII	Bra007640	GroupII-c	Bra004466
ERF	Bra004641	IV	Bra001671	R2R3-MYB	Bra025564	XIII	Bra023288	GroupIII	Bra004540
ERF_B-4	Bra006195	IV	Bra022225	1R-MYB	Bra039503	XIII	Bra010183	GroupIII	Bra004586
ERF_B-1	Bra006325	III	Bra004689	R2R3-MYB	Bra029129	XIII	Bra016098	GroupII-c	Bra004864
ERF_B-3	Bra004873	III	Bra003320	R2R3-MYB	Bra000453	XIII	Bra007996	GroupI	Bra005104
ERF_B-1	Bra003701	III	Bra007380	R2R3-MYB	Bra028892	XIII	Bra016783	GroupI	Bra005210
DREB_A-5	Bra016133	III	Bra000256	1R-MYB	Bra025914	XIII	Bra019750	GroupII-e	Bra005388
ERF_B-1	Bra006466	III	Bra009288	1R-MYB	Bra005997	XIII	Bra026981	GroupII-d	Bra018303
RAV	Bra038346	III	Bra030310	R2R3-MYB	Bra032028	XIII	Bra027024	GroupI	Bra018325
DREB_A-1	Bra010463	III	Bra030312	R2R3-MYB	Bra027389	XIII	Bra012065	GroupII-c	Bra037106
ERF_B-3	Bra009250	III	Bra032191	R2R3-MYB	Bra034841	XIII	Bra039915	GroupI	Bra039147
DREB_A-5	Bra012298	III	Bra034916	R2R3-MYB	Bra003801	XIII	Bra011667	GroupIII	Bra009734
ERF_B-4	Bra008835	III	Bra030314	R2R3-MYB	Bra008705	XIII	Bra017750	GroupII-c	Bra009885
ERF_B-2	Bra021401	III	Bra031172	R2R3-MYB	Bra017963	XII	Bra016294	GroupII-d	Bra010005
DREB_A-6	Bra010383	III	Bra010722	1R-MYB	Bra036412	XII	Bra024677	GroupII-c	Bra010032
ERF_B-1	Bra010881	III	Bra033582	1R-MYB	Bra016530	XII	Bra004403	GroupI	Bra019697
ERF_B-5	Bra019270	III	Bra030663	R2R3-MYB	Bra022831	XII	Bra028441	GroupII-c	Bra024328
ERF_B-1	Bra010972	III	Bra031541	R2R3-MYB	Bra034853	XII	Bra034719	GroupI	Bra024780
ERF_B-1	Bra012499	III	Bra000195	1R-MYB	Bra011870	XII	Bra039353	GroupII-c	Bra025021
AP2	Bra018899	III	Bra016980	R2R3-MYB	Bra003793	XII	Bra037743	GroupII-b	Bra025693
ERF_B-1	Bra032898	III	Bra004582	1R-MYB	Bra026297	XII	Bra000282	GroupII-c	Bra033616
ERF_B-2	Bra003883	III	Bra030637	R2R3-MYB	Bra009346	XII	Bra004736	GroupII-c	Bra038006
ERF_B-3	Bra015310	III	Bra015471	1R-MYB	Bra015766	XI	Bra013272	GroupIII	Bra003239
ERF	Bra000487	III	Bra021735	R2R3-MYB	Bra002107	XI	Bra012626	GroupII-b	Bra003588
ERF_B-3	Bra021048	III	Bra018250	R2R3-MYB	Bra004297	XI	Bra022046	GroupII-c	Bra004002
ERF_B-1	Bra001588	II	Bra034147	R2R3-MYB	Bra022602	XI	Bra025012	GroupII-b	Bra004285
ERF_B-3	Bra024954	II	Bra023224	R2R3-MYB	Bra029553	XI	Bra035488	GroupII-c	Bra004370
ERF_B-6	Bra004033	II	Bra016959	1R-MYB	Bra027882	XI	Bra030807	GroupII-b	Bra016263
ERF_B-1	Bra038261	II	Bra014680	R2R3-MYB	Bra001284	XI	Bra003335	GroupII-c	Bra030178
ERF_B-1	Bra006222	I	Bra034925	R2R3-MYB	Bra033078	XI	Bra039745	GroupII-b	Bra035148
AP2	Bra006065	I	Bra024310	R2R3-MYB	Bra040274	XI	Bra022236	GroupII-b	Bra010220
DREB_A-4	Bra016761	I	Bra013048	1R-MYB	Bra012815	XI	Bra021366	GroupII-d	Bra010231
DREB_A-4	Bra034535	I	Bra037290	R2R3-MYB	Bra025311	XI	Bra032009	GroupI	Bra010266
ERF_B-5	Bra022643	I	Bra011580	1R-MYB	Bra017943	XI	Bra008567	GroupI	Bra010431
AP2	Bra031234	I	Bra034668	R2R3-MYB	Bra025574	XI	Bra006392	GroupII-c	Bra010683
ERF_B-3	Bra032433	I	Bra006886	1R-MYB	Bra014512	X	Bra034118	GroupII-e	Bra010839
DREB_A-6	Bra040381	I	Bra033464	1R-MYB	Bra037837	X	Bra034119	GroupII-b	Bra016535
DREB_A-5	Bra014056	I	Bra024366	R2R3-MYB	Bra012641	X	Bra029844	GroupI	Bra016716
ERF	Bra020262	I	Bra031871	R2R3-MYB	Bra039040	X	Bra001365	GroupII-e	Bra020814
DREB_A-4	Bra015686	I	Bra037809	R2R3-MYB	Bra016285	X	Bra001366	GroupII-c	Bra030847
DREB_A-4	Bra003415	I	Bra028604	R2R3-MYB	Bra008740	X	Bra023314	GroupII-c	Bra030848
ERF_B-6	Bra009824	I	Bra009063	R2R3-MYB	Bra020228	X	Bra008980	GroupIII	Bra007243
ERF_B-1	Bra032900	I	Bra037374	R2R3-MYB	Bra029033	X	Bra035712	GroupII-e	Bra007413
DREB_A-5	Bra037124	I	Bra015646	R2R3-MYB	Bra004155	X	Bra009456	GroupII-c	Bra007674
ERF_B-3	Bra014925	I	Bra016389	R2R3-MYB	Bra013900	X	Bra028790	GroupII-c	Bra017561
ERF_B-3	Bra021047	I	Bra031364	R2R3-MYB	Bra027668	X	Bra005804	GroupIII	Bra023211
DREB_A-6	Bra024357	I	Bra018634	R2R3-MYB	Bra031847	X	Bra034120	GroupIII	Bra026467
ERF_B-1	Bra001784	I	Bra031622	R2R3-MYB	Bra012038	X	Bra024263	GroupI	Bra026869
DREB_A-2	Bra017011	I	Bra004329	R2R3-MYB	Bra006977	X	Bra009379	GroupII-b	Bra027057

ERF_B-3	Bra018271	I	Bra028713	1R-MYB	Bra010560	X	Bra028640	GroupII-c	Bra027480
ERF_B-6	Bra033923	I	Bra009241	1R-MYB	Bra016409	X	Bra006019	GroupII-c	Bra027675
ERF_B-1	Bra008460	I	Bra038705	R2R3-MYB	Bra034842	IX	Bra017980	GroupII-c	Bra027768
ERF_B-3	Bra017495	I	Bra001443	1R-MYB	Bra030496	IX	Bra019599	GroupII-b	Bra029491
ERF_B-1	Bra027270	I	Bra034767	R2R3-MYB	Bra029582	IX	Bra009774	GroupII-b	Bra031053
ERF_B-3	Bra023744			R2R3-MYB	Bra006166	IX	Bra036520	GroupII-c	Bra031221
DREB_A-1	Bra019162			R2R3-MYB	Bra012149	IX	Bra010193	GroupII-e	Bra032300
ERF_B-6	Bra016663			1R-MYB	Bra016598	IX	Bra012024	GroupII-c	Bra032340
DREB_A-4	Bra034273			R2R3-MYB	Bra020744	IX	Bra021471	GroupII-c	Bra036138
ERF_B-3	Bra032512			R2R3-MYB	Bra028063	IX	Bra006619	GroupII-b	Bra036315
DREB_A-2	Bra034159			1R-MYB	Bra012183	IX	Bra020182	GroupII-e	Bra037368
ERF_B-3	Bra028690			R2R3-MYB	Bra023637	IX	Bra002403	GroupII-c	Bra037796
ERF_B-3	Bra017612			R2R3-MYB	Bra035532	VIII	Bra034439	GroupIII	Bra002421
DREB_A-2	Bra007341			R2R3-MYB	Bra025361	VIII	Bra034436	GroupI	Bra002828
ERF	Bra023617			R2R3-MYB	Bra011576	VIII	Bra036744	GroupII-b	Bra008719
ERF_B-6	Bra034250			R2R3-MYB	Bra000038	VIII	Bra028039	GroupII-c	Bra008858
DREB_A-4	Bra010210			R2R3-MYB	Bra008131	VIII	Bra012342	GroupII-d	Bra040396
ERF_B-4	Bra031903			R2R3-MYB	Bra038774	VIII	Bra013034	GroupII-c	Bra040557
DREB_A-5	Bra011744			1R-MYB	Bra007371	VIII	Bra037283	GroupII-c	Bra040926
DREB_A-1	Bra022770			R2R3-MYB	Bra004162	VIII	Bra002018	GroupII-e	Bra041029
ERF_B-1	Bra023440			R2R3-MYB	Bra033572	VIII	Bra024818		
ERF_B-3	Bra040159			R2R3-MYB	Bra001907	VIII	Bra026595		
ERF_B-1	Bra018864			R2R3-MYB	Bra010022	VIII	Bra017416		
DREB_A-5	Bra011244			R2R3-MYB	Bra039395	VIII	Bra019442		
DREB_A-6	Bra008343			R2R3-MYB	Bra022888	VIII	Bra019459		
RAV	Bra025159			1R-MYB	Bra014169	VIII	Bra006624		
AP2	Bra024394			1R-MYB	Bra034132	VIII	Bra002408		
RAV	Bra030458			R2R3-MYB	Bra020289	VIII	Bra020188		
DREB_A-4	Bra040333			R2R3-MYB	Bra006811	VII	Bra006229		
DREB_A-2	Bra032665			R2R3-MYB	Bra016575	VII	Bra008788		
DREB_A-4	Bra023070			1R-MYB	Bra040578	VI	Bra036326		
DREB_A-6	Bra017879			R2R3-MYB	Bra021483	VI	Bra036327		
ERF_B-3	Bra024953			1R-MYB	Bra008523	VI	Bra008553		
ERF_B-3	Bra029319			R2R3-MYB	Bra029113	VI	Bra036328		
ERF_B-6	Bra008985			1R-MYB	Bra012743	VI	Bra027596		
AP2	Bra011782			1R-MYB	Bra031041	VI	Bra033297		
DREB_A-6	Bra031146			1R-MYB	Bra015856	VI	Bra033296		
ERF_B-1	Bra023684			R2R3-MYB	Bra005818	VI	Bra010895		
ERF_B-1	Bra019801			R2R3-MYB	Bra008596	VI	Bra033302		
DREB_A-6	Bra027697			1R-MYB	Bra026253	VI	Bra033305		
ERF_B-6	Bra026140			R2R3-MYB	Bra013055	VI	Bra033306		
DREB_A-6	Bra015634			1R-MYB	Bra039470	VI	Bra033298		
DREB_A-6	Bra024222			R2R3-MYB	Bra024419	VI	Bra032615		
ERF_B-1	Bra030095			1R-MYB	Bra012919	VI	Bra033299		
ERF_B-1	Bra030076			R2R3-MYB	Bra037419	VI	Bra033303		
ERF_B-6	Bra036536			R2R3-MYB	Bra017813	VI	Bra033308		
ERF_B-6	Bra036360			R2R3-MYB	Bra020526	VI	Bra033300		
ERF_B-3	Bra023746			R2R3-MYB	Bra011785	V	Bra001000		
ERF_B-1	Bra030094			R2R3-MYB	Bra024368	V	Bra006257		
ERF_B-6	Bra002262			R2R3-MYB	Bra011749	V	Bra012470		
ERF_B-1	Bra032901			R2R3-MYB	Bra012337	V	Bra024733		
ERF_B-1	Bra015266			1R-MYB	Bra031060	V	Bra011090		
ERF_B-2	Bra021543			R2R3-MYB	Bra027771	V	Bra024152		
AP2	Bra003178			1R-MYB	Bra012665	V	Bra002802		
ERF	Bra002510			R2R3-MYB	Bra018772	V	Bra032910		
DREB_A-4	Bra032630			R2R3-MYB	Bra021708	V	Bra030099		
DREB_A-6	Bra027736			R2R3-MYB	Bra018223	V	Bra024197		
DREB_A-4	Bra022573			R2R3-MYB	Bra021382	V	Bra011034		
DREB_A-5	Bra020162			R2R3-MYB	Bra017685	IV	Bra033405		
ERF_B-6	Bra020490			1R-MYB	Bra029673	IV	Bra004575		
DREB_A-1	Bra010460			R2R3-MYB	Bra019368	IV	Bra004579		
ERF_B-6	Bra024743			1R-MYB	Bra018818	IV	Bra020326		
ERF_B-2	Bra001640			R2R3-MYB	Bra001472	IV	Bra020327		
ERF_B-6	Bra025170			R2R3-MYB	Bra028997	IV	Bra036314		
DREB_A-4	Bra028291			R2R3-MYB	Bra026281	IV	Bra036304		
AP2	Bra011741			R2R3-MYB	Bra016546	IV	Bra036305		
DREB_A-6	Bra011833			R2R3-MYB	Bra015029	III	Bra002605		
ERF_B-6	Bra010429			R2R3-MYB	Bra001917	III	Bra007254		
ERF_B-2	Bra001541			1R-MYB	Bra015890	III	Bra028486		
DREB_A-4	Bra040092			R2R3-MYB	Bra001404	II	Bra003237		
DREB_A-6	Bra032724			R2R3-MYB	Bra035893	II	Bra025701		
ERF_B-4	Bra028703			1R-MYB	Bra002790	II	Bra029571		
ERF_B-1	Bra026942			1R-MYB	Bra029111	II	Bra002857		
ERF_B-3	Bra013310			R2R3-MYB	Bra034057	II	Bra031518		
ERF_B-5	Bra012352			1R-MYB	Bra021864	II	Bra035412		
DREB_A-5	Bra015660			R2R3-MYB	Bra015912	I	Bra000192		
DREB_A-5	Bra007974			R2R3-MYB	Bra005139	I	Bra015108		

ERF_B-5	Bra040839			1R-MYB	Bra039650						
DREB_A-1	Bra026963			R2R3-MYB	Bra025575						
DREB_A-5	Bra015882			R2R3-MYB	Bra036145						
ERF_B-1	Bra035792			R2R3-MYB	Bra025589						
ERF_B-3	Bra015478			1R-MYB	Bra016037						
ERF_B-3	Bra040158			1R-MYB	Bra029978						
ERF_B-5	Bra000736			1R-MYB	Bra001311						
RAV	Bra018868			R2R3-MYB	Bra036048						
AP2	Bra012139			1R-MYB	Bra017487						
AP2	Bra007066			1R-MYB	Bra001643						
ERF_B-1	Bra008793			1R-MYB	Bra020390						
ERF_B-6	Bra026770			R2R3-MYB	Bra025678						
ERF_B-4	Bra009272			1R-MYB	Bra009699						
RAV	Bra026917			R2R3-MYB	Bra001005						
ERF_B-3	Bra035919			R2R3-MYB	Bra012482						
ERF_B-3	Bra011529			3R-MYB	Bra009597						
ERF_B-3	Bra037630			R2R3-MYB	Bra001323						
ERF	Bra034895			1R-MYB	Bra006915						
ERF_B-4	Bra024325			R2R3-MYB	Bra025410						
AP2	Bra013978			R2R3-MYB	Bra010085						
DREB_A-4	Bra028009			R2R3-MYB	Bra006370						
ERF_B-1	Bra039658			1R-MYB	Bra033291						
DREB_A-1	Bra016763			R2R3-MYB	Bra010593						
DREB_A-2	Bra029889			R2R3-MYB	Bra010021						
AP2	Bra026082			R2R3-MYB	Bra031008						
DREB_A-2	Bra009112			1R-MYB	Bra036035						
RAV	Bra011002			R2R3-MYB	Bra017218						
AP2	Bra020444			R2R3-MYB	Bra036588						
AP2	Bra035137			1R-MYB	Bra036679						
AP2	Bra009026			R2R3-MYB	Bra019154						
ERF_B-6	Bra006518			1R-MYB	Bra007957						
DREB_A-5	Bra016400			R2R3-MYB	Bra003995						
DREB_A-1	Bra028290			R2R3-MYB	Bra015992						
ERF_B-1	Bra010880			R2R3-MYB	Bra026720						
ERF	Bra023921			R2R3-MYB	Bra002938						
ERF_B-3	Bra017656			1R-MYB	Bra028780						
DREB_A-4	Bra005314			1R-MYB	Bra029856						
AP2	Bra017809			1R-MYB	Bra004441						
RAV	Bra030476			R2R3-MYB	Bra028550						
ERF_B-2	Bra021200			R2R3-MYB	Bra006055						
DREB_A-1	Bra027612			1R-MYB	Bra002610						
DREB_A-4	Bra003924			R2R3-MYB	Bra029696						
DREB_A-5	Bra032173			1R-MYB	Bra005751						
ERF_B-5	Bra016367			R2R3-MYB	Bra020892						
ERF_B-1	Bra021594			R2R3-MYB	Bra028593						
DREB_A-4	Bra004878			1R-MYB	Bra022127						
ERF_B-4	Bra021871			R2R3-MYB	Bra015899						
ERF_B-3	Bra022115			R2R3-MYB	Bra033948						
ERF_B-1	Bra002168			R2R3-MYB	Bra003619						
ERF_B-3	Bra035732			R2R3-MYB	Bra012961						
ERF_B-2	Bra027355			1R-MYB	Bra009591						
ERF_B-5	Bra026295			R2R3-MYB	Bra008522						
ERF_B-1	Bra038107			R2R3-MYB	Bra006249						
ERF_B-5	Bra003462			1R-MYB	Bra009432						
DREB_A-2	Bra005852			1R-MYB	Bra029778						
DREB_A-3	Bra004978			1R-MYB	Bra031952						
DREB_A-4	Bra027616			R2R3-MYB	Bra035097						
RAV	Bra019821			1R-MYB	Bra037388						
ERF_B-3	Bra022832			R2R3-MYB	Bra017138						
DREB_A-3	Bra000178			1R-MYB	Bra041027						
DREB_A-4	Bra027614			1R-MYB	Bra003969						
DREB_A-4	Bra036552			R2R3-MYB	Bra015911						
ERF_B-2	Bra016063			1R-MYB	Bra011691						
DREB_A-5	Bra012345			R2R3-MYB	Bra035547						
DREB_A-6	Bra031371			R2R3-MYB	Bra034173						
DREB_A-1	Bra010461			R2R3-MYB	Bra025300						
DREB_A-2	Bra002159			R2R3-MYB	Bra006296						
ERF_B-4	Bra005932			R2R3-MYB	Bra021791						
ERF_B-1	Bra026949			R2R3-MYB	Bra025666						
DREB_A-2	Bra003752			1R-MYB	Bra023610						
DREB_A-4	Bra007975			1R-MYB	Bra016893						
ERF_B-3	Bra012938			R2R3-MYB	Bra015939						
ERF_B-3	Bra034624			R2R3-MYB	Bra010736						
ERF_B-3	Bra021049			R2R3-MYB	Bra018267						
ERF_B-5	Bra029079			R2R3-MYB	Bra039067						
DREB_A-5	Bra012140			R2R3-MYB	Bra029349						
DREB_A-4	Bra011383			R2R3-MYB	Bra013526						

DREB_A-5	Bra002377			1R-MYB	Bra003870				
ERF_B-6	Bra019087			1R-MYB	Bra030992				
DREB_A-5	Bra031069			R2R3-MYB	Bra002448				
DREB_A-2	Bra001386			1R-MYB	Bra039617				
ERF_B-5	Bra004548			R2R3-MYB	Bra000680				
DREB_A-4	Bra000366			R2R3-MYB	Bra025652				
ERF_B-5	Bra013730			1R-MYB	Bra005230				
DREB_A-4	Bra023332			1R-MYB	Bra024760				
				R2R3-MYB	Bra030783				
				R2R3-MYB	Bra005234				
				R2R3-MYB	Bra039142				
				R2R3-MYB	Bra016553				
				1R-MYB	Bra022637				
				R2R3-MYB	Bra023515				
				R2R3-MYB	Bra027423				
				1R-MYB	Bra037365				
				R2R3-MYB	Bra004473				
				R2R3-MYB	Bra002533				
				1R-MYB	Bra009562				
				R2R3-MYB	Bra020624				
				1R-MYB	Bra025681				
				R2R3-MYB	Bra008149				
				R2R3-MYB	Bra014929				
				4R-MYB	Bra022280				
				1R-MYB	Bra029762				
				3R-MYB	Bra034067				
				R2R3-MYB	Bra037828				
				3R-MYB	Bra013415				
				R2R3-MYB	Bra014446				
				1R-MYB	Bra029320				
				R2R3-MYB	Bra031669				
				1R-MYB	Bra020584				
				R2R3-MYB	Bra021515				
				1R-MYB	Bra034074				
				1R-MYB	Bra012623				
				R2R3-MYB	Bra005006				
				R2R3-MYB	Bra039763				
				R2R3-MYB	Bra025337				
				1R-MYB	Bra006741				
				R2R3-MYB	Bra001202				
				R2R3-MYB	Bra039065				
				1R-MYB	Bra007536				
				R2R3-MYB	Bra028654				
				1R-MYB	Bra009910				
				R2R3-MYB	Bra006422				
				R2R3-MYB	Bra035929				
				1R-MYB	Bra007506				
				R2R3-MYB	Bra005949				
				R2R3-MYB	Bra040181				
				1R-MYB	Bra008563				
				R2R3-MYB	Bra033067				
				1R-MYB	Bra003117				
				1R-MYB	Bra009044				
				1R-MYB	Bra035457				
				1R-MYB	Bra006394				
				R2R3-MYB	Bra020428				
				1R-MYB	Bra022183				
				R2R3-MYB	Bra002847				
				R2R3-MYB	Bra029311				
				1R-MYB	Bra031282				
				R2R3-MYB	Bra009134				
				R2R3-MYB	Bra029350				
				R2R3-MYB	Bra018598				
				R2R3-MYB	Bra008160				
				R2R3-MYB	Bra015481				
				R2R3-MYB	Bra007588				
				R2R3-MYB	Bra035604				
				1R-MYB	Bra030894				
				1R-MYB	Bra012721				
				1R-MYB	Bra006853				
				R2R3-MYB	Bra020016				
				R2R3-MYB	Bra004041				
				R2R3-MYB	Bra008764				
The identified five families of TFs in B. olercea									
Subfamily	BoAP2/EREBP	Subfami	BobZIP	Subfamily	BoMYB	Subfamily	BoNAC	Subfamily	BoWRKY
DREB_A-5	Bo1g004940.1	XI	Bo3g073080.1	R2R3-MYB	Bo8g049720.1	XVI	Bo1g005810.1	GroupII-d	Bo00834s070.1
ERF_B-3	Bo8g114710.1	XI	Bo5g115300.1	1R-MYB	Bo8g117860.1	XVI	Bo7g117820.1	GroupIII	Bo00913s010.1

ERF_B-1	Bo5g055860.1	XI	Bo1g114840.1	R2R3-MYB	Bo2g030240.1	XVI	Bo7g011640.1	GroupIII	Bo01002s040.1
DREB_A-4	Bo5g086680.1	XI	Bo6g018480.1	R2R3-MYB	Bo3g041750.1	XVI	Bo3g093950.1	GroupII-e	Bo9g003610.1
DREB_A-4	Bo9g028820.1	XI	Bo7g116570.1	1R-MYB	Bo3g068550.1	XVI	Bo7g065010.1	GroupII-b	Bo9g004230.1
DREB_A-4	Bo6g121150.1	XI	Bo1g007700.1	R2R3-MYB	Bo3g023270.1	XVI	Bo5g017120.1	GroupII-c	Bo9g009900.1
DREB_A-6	Bo1g002840.1	X	Bo6g006970.1	3R-MYB	Bo3g007070.1	XVI	Bo8g108300.1	GroupII-c	Bo9g011080.1
DREB_A-6	Bo3g100470.1	IX	Bo4g034870.1	1R-MYB	Bo8g095200.1	XVI	Bo8g062650.1	GroupIII	Bo9g013780.1
ERF_B-6	Bo7g111070.1	IX	Bo4g186090.1	R2R3-MYB	Bo9g008750.1	XV	Bo3g107700.1	GroupII-c	Bo9g018440.1
ERF_B-1	Bo7g048190.1	IX	Bo3g122120.1	R2R3-MYB	Bo1g144680.1	XV	Bo2g164090.1	GroupII-c	Bo9g030630.1
ERF_B-1	Bo6g029320.1	IX	Bo4g012940.1	R2R3-MYB	Bo9g140750.1	XV	Bo9g014940.1	GroupII-c	Bo9g033920.1
ERF_B-3	Bo8g118250.1	IX	Bo4g112690.1	R2R3-MYB	Bo1g138250.1	XIV	Bo2g075130.1	GroupII-b	Bo9g038600.1
DREB_A-4	Bo1g053270.1	IX	Bo8g091160.1	1R-MYB	Bo1g005280.1	XIV	Bo6g114440.1	GroupII-c	Bo9g059010.1
ERF_B-3	Bo7g098850.1	IX	Bo6g072380.1	1R-MYB	Bo7g104390.1	XIII	Bo2g110370.1	GroupII-b	Bo9g078130.1
DREB_A-5	Bo5g034880.1	VIII	Bo4g078610.1	R2R3-MYB	Bo9g014610.1	XIII	Bo9g088300.1	GroupI	Bo9g123740.1
ERF_B-3	Bo9g001020.1	VII	Bo5g004230.1	R2R3-MYB	Bo5g021910.1	XIII	Bo6g123980.1	GroupII-b	Bo9g165380.1
ERF_B-3	Bo1g102210.1	VII	Bo7g067030.1	R2R3-MYB	Bo8g076540.1	XIII	Bo2g095440.1	GroupII-c	Bo9g169080.1
ERF_B-6	Bo2g060040.1	VI	Bo7g118720.1	1R-MYB	Bo8g098010.1	XIII	Bo6g080930.1	GroupII-c	Bo6g034690.1
ERF_B-2	Bo4g198690.1	VI	Bo1g003470.1	1R-MYB	Bo5g002760.1	XIII	Bo5g049360.1	GroupII-c	Bo6g051390.1
ERF_B-2	Bo5g123710.1	VI	Bo3g097630.1	1R-MYB	Bo2g006670.1	XIII	Bo5g086900.1	GroupIII	Bo6g066790.1
DREB_A-5	Bo5g038890.1	VI	Bo2g168170.1	1R-MYB	Bo7g104190.1	XIII	Bo4g006990.1	GroupIII	Bo6g071110.1
AP2	Bo2g013700.1	VI	Bo1g080160.1	R2R3-MYB	Bo8g111090.1	XIII	Bo4g197330.1	GroupII-b	Bo6g080740.1
ERF_B-6	Bo2g158960.1	VI	Bo8g079020.1	R2R3-MYB	Bo3g019230.1	XIII	Bo8g097430.1	GroupII-c	Bo6g095590.1
ERF_B-3	Bo1g019590.1	VI	Bo9g048720.1	R2R3-MYB	Bo5g025730.1	XIII	Bo6g066300.1	GroupII-b	Bo6g106590.1
ERF_B-2	Bo1g134510.1	VI	Bo9g009810.1	R2R3-MYB	Bo8g105490.1	XIII	Bo6g079180.1	GroupII-c	Bo6g108750.1
ERF_B-1	Bo7g048170.1	VI	Bo7g092760.1	R2R3-MYB	Bo3g162580.1	XII	Bo3g185760.1	GroupII-b	Bo6g110430.1
ERF_B-5	Bo9g111050.1	VI	Bo9g024710.1	R2R3-MYB	Bo9g172290.1	XII	Bo5g132990.1	GroupII-b	Bo6g124190.1
DREB_A-5	Bo3g015600.1	VI	Bo3g156810.1	3R-MYB	Bo3g171130.1	XII	Bo3g065240.1	GroupII-e	Bo5g058590.1
DREB_A-1	Bo8g050800.1	VI	Bo8g097830.1	R2R3-MYB	Bo4g046000.1	XII	Bo1g017380.1	GroupII-c	Bo5g061050.1
DREB_A-4	Bo4g024220.1	VI	Bo6g079450.1	1R-MYB	Bo8g104830.1	XII	Bo7g113180.1	GroupII-e	Bo5g063280.1
DREB_A-4	Bo5g002600.1	VI	Bo6g086380.1	R2R3-MYB	Bo9g164230.1	XII	Bo8g054390.1	GroupIII	Bo5g068250.1
ERF_B-5	Bo3g045150.1	VI	Bo2g083760.1	R2R3-MYB	Bo9g174380.1	XI	Bo01320s020.1	GroupII-c	Bo5g153870.1
ERF	Bo11579s010.1	VI	Bo6g119550.1	R2R3-MYB	Bo3g039690.1	XI	Bo3g071500.1	GroupI	Bo5g156460.1
DREB_A-5	Bo6g004620.1	VI	Bo7g011520.1	3R-MYB	Bo9g184660.1	XI	Bo1g117610.1	GroupII-e	Bo7g043420.1
DREB_A-2	Bo9g177130.1	VI	Bo3g093980.1	R2R3-MYB	Bo2g009800.1	XI	Bo4g154590.1	GroupII-c	Bo7g044930.1
ERF_B-3	Bo3g168910.1	VI	Bo9g026190.1	R2R3-MYB	Bo3g153550.1	XI	Bo8g099810.1	GroupII-c	Bo7g075300.1
ERF_B-6	Bo7g111100.1	VI	Bo3g168760.1	R2R3-MYB	Bo6g018000.1	XI	Bo6g120460.1	GroupI	Bo7g081130.1
DREB_A-5	Bo2g072950.1	VI	Bo7g116940.1	1R-MYB	Bo6g018740.1	XI	Bo2g089890.1	GroupII-e	Bo7g088570.1
ERF_B-2	Bo5g130490.1	VI	Bo1g007140.1	1R-MYB	Bo6g057640.1	XI	Bo5g127410.1	GroupII-c	Bo7g092790.1
ERF_B-1	Bo5g109800.1	V	Bo4g038560.1	R2R3-MYB	Bo1g024170.1	XI	Bo1g129730.1	GroupII-d	Bo7g093090.1
DREB_A-4	Bo6g113020.1	V	Bo3g028300.1	1R-MYB	Bo6g119390.1	XI	Bo3g066690.1	GroupII-c	Bo7g095430.1
DREB_A-5	Bo1g011650.1	V	Bo5g048660.1	3R-MYB	Bo7g115810.1	XI	Bo2g043280.1	GroupIII	Bo7g097170.1
ERF_B-5	Bo6g077880.1	V	Bo4g183320.1	R2R3-MYB	Bo2g047590.1	XI	Bo9g113820.1	GroupII-c	Bo7g105800.1
DREB_A-6	Bo5g037400.1	V	Bo1g005460.1	3R-MYB	Bo9g171130.1	XI	Bo9g008770.1	GroupII-e	Bo7g108870.1
AP2	Bo5g021520.1	V	Bo9g003700.1	R2R3-MYB	Bo9g163020.1	XI	Bo7g087220.1	GroupIII	Bo7g109140.1
DREB_A-1	Bo8g108050.1	V	Bo3g039150.1	R2R3-MYB	Bo8g090160.1	XI	Bo00578s090.1	GroupII-d	Bo7g109430.1
ERF_B-3	Bo2g132270.1	V	Bo01108s040.1	R2R3-MYB	Bo3g165540.1	XI	Bo6g062160.1	GroupI	Bo7g110920.1
DREB_A-2	Bo6g119660.1	V	Bo4g010410.1	1R-MYB	Bo3g184590.1	XI	Bo2g008760.1	GroupI	Bo7g111000.1
DREB_A-6	Bo00790s010.1	V	Bo9g014350.1	1R-MYB	Bo3g002070.1	XI	Bo9g175670.1	GroupI	Bo7g114840.1
DREB_A-5	Bo9g020380.1	V	Bo7g098020.1	1R-MYB	Bo7g011290.1	XI	Bo2g161570.1	GroupII-d	Bo7g115250.1
ERF_B-6	Bo6g096060.1	V	Bo2g161130.1	R2R3-MYB	Bo1g134790.1	XI	Bo7g098620.1	GroupII-b	Bo7g115410.1
ERF_B-6	Bo6g106990.1	V	Bo3g004720.1	R2R3-MYB	Bo1g090540.1	XI	Bo9g157320.1	GroupII-c	Bo7g119200.1
ERF_B-3	Bo7g116860.1	V	Bo7g081750.1	1R-MYB	Bo9g069510.1	XI	Bo3g013050.1	GroupII-c	Bo4g004680.1
ERF_B-1	Bo5g127380.1	V	Bo9g075700.1	R2R3-MYB	Bo6g118710.1	XI	Bo2g015440.1	GroupIII	Bo4g009270.1
DREB_A-5	Bo8g068370.1	V	Bo2g138370.1	R2R3-MYB	Bo7g050560.1	XI	Bo5g148430.1	GroupIII	Bo4g012750.1
DREB_A-4	Bo8g095240.1	V	Bo8g107310.1	R2R3-MYB	Bo7g118450.1	XI	Bo1g152230.1	GroupII-c	Bo4g024050.1
DREB_A-4	Bo3g028500.1	V	Bo5g017580.1	1R-MYB	Bo5g101040.1	X	Bo3g009040.1	GroupI	Bo4g027280.1
ERF_B-5	Bo7g109100.1	V	Bo2g061410.1	1R-MYB	Bo5g138190.1	X	Bo2g011120.1	GroupI	Bo4g030720.1
DREB_A-2	Bo3g063910.1	V	Bo7g088760.1	1R-MYB	Bo4g182070.1	X	Bo9g168950.1	GroupII-e	Bo4g039260.1
ERF_B-5	Bo2g070850.1	V	Bo2g149490.1	1R-MYB	Bo1g113580.1	X	Bo4g043670.1	GroupII-d	Bo4g056260.1
AP2	Bo4g070350.1	V	Bo9g163740.1	1R-MYB	Bo1g054810.1	X	Bo4g181990.1	GroupI	Bo4g059620.1
ERF_B-3	Bo3g025110.1	V	Bo2g012800.1	1R-MYB	Bo3g012470.1	X	Bo02109s010.1	GroupII-b	Bo4g092630.1
DREB_A-6	Bo6g121730.1	V	Bo3g010620.1	1R-MYB	Bo3g178250.1	X	Bo2g009250.1	GroupIII	Bo4g101290.1
ERF_B-3	Bo1g102220.1	V	Bo3g010750.1	R2R3-MYB	Bo4g151410.1	X	Bo2g165880.1	GroupII-e	Bo4g108250.1
ERF	Bo4g013420.1	IV	Bo2g159200.1	R2R3-MYB	Bo7g085930.1	X	Bo9g174170.1	GroupIII	Bo4g114050.1
DREB_A-5	Bo2g023630.1	IV	Bo7g096540.1	1R-MYB	Bo8g095170.1	X	Bo3g005100.1	GroupII-c	Bo4g142200.1
DREB_A-2	Bo8g092010.1	IV	Bo7g093030.1	R2R3-MYB	Bo3g081700.1	X	Bo5g001100.1	GroupII-c	Bo4g148210.1
AP2	Bo7g064000.1	IV	Bo2g151850.1	R2R3-MYB	Bo2g057760.1	X	Bo2g092550.1	GroupII-d	Bo4g151440.1
DREB_A-6	Bo00615s260.1	IV	Bo9g005970.1	1R-MYB	Bo6g112670.1	X	Bo7g111390.1	GroupII-d	Bo4g154730.1
ERF_B-3	Bo2g008700.1	IV	Bo3g032910.1	R2R3-MYB	Bo3g005530.1	X	Bo1g048300.1	GroupI	Bo4g171890.1
ERF_B-6	Bo2g018360.1	IV	Bo4g119810.1	R2R3-MYB	Bo2g036530.1	X	Bo6g030940.1	GroupII-d	Bo4g173290.1
ERF_B-4	Bo9g018330.1	IV	Bo8g084770.1	R2R3-MYB	Bo6g099800.1	X	Bo1g128450.1	GroupII-e	Bo4g182940.1
DREB_A-5	Bo7g063980.1	III	Bo5g027510.1	R2R3-MYB	Bo6g099880.1	X	Bo5g127040.1	GroupI	Bo4g187630.1
AP2	Bo8g083590.1	II	Bo3g069000.1	1R-MYB	Bo5g123620.1	X	Bo3g066850.1	GroupIII	Bo4g190380.1
ERF_B-1	Bo1g129710.1	II	Bo5g122830.1	3R-MYB	Bo1g144510.1	X	Bo5g148420.1	GroupIII	Bo4g190390.1
AP2	Bo1g108580.1	II	Bo1g119430.1	R2R3-MYB	Bo3g023140.1	X	Bo1g152220.1	GroupII-c	Bo4g195160.1
RAV	Bo2g061320.1	II	Bo9g171430.1	R2R3-MYB	Bo3g025100.1	X	Bo6g095510.1	GroupII-c	Bo4g198560.1
DREB_A-4	Bo2g048150.1	II	Bo2g010330.1	R2R3-MYB	Bo5g009920.1	X	Bo6g108930.1	GroupII-c	Bo1g002540.1
AP2	Bo5g081310.1	II	Bo8g024280.1	R2R3-MYB	Bo1g155040.1	X	Bo01282s010.1	GroupII-b	Bo1g010960.1

ERF_B-3	Bo1g007330.1	II	Bo4g112860.1	R2R3-MYB	Bo5g038720.1	X	Bo6g030920.1	GroupII-d	Bo1g011200.1
DREB_A-4	Bo9g036590.1	II	Bo4g190610.1	R2R3-MYB	Bo5g150350.1	X	Bo3g066880.1	GroupII-c	Bo1g019260.1
DREB_A-5	Bo6g113060.1	II	Bo4g012880.1	1R-MYB	Bo7g072540.1	X	Bo5g127030.1	GroupII-b	Bo1g025900.1
DREB_A-5	Bo8g079660.1	II	Bo4g015960.1	R2R3-MYB	Bo2g012520.1	X	Bo1g128420.1	GroupII-e	Bo1g035810.1
ERF_B-3	Bo7g040810.1	II	Bo6g074510.1	R2R3-MYB	Bo3g022210.1	X	Bo9g018140.1	GroupII-d	Bo1g037740.1
ERF_B-1	Bo3g143340.1	II	Bo8g092430.1	R2R3-MYB	Bo3g008870.1	X	Bo2g166280.1	GroupI	Bo1g045230.1
RAV	Bo3g179890.1	II	Bo9g175190.1	R2R3-MYB	Bo6g118730.1	X	Bo3g101220.1	GroupI	Bo1g046510.1
ERF_B-4	Bo2g008570.1	II	Bo5g070650.1	R2R3-MYB	Bo2g082270.1	X	Bo3g009490.1	GroupII-b	Bo1g158770.1
ERF_B-3	Bo8g049450.1	II	Bo8g034370.1	1R-MYB	Bo5g010120.1	X	Bo9g166250.1	GroupII-c	Bo3g008970.1
DREB_A-4	Bo4g184540.1	II	Bo4g052150.1	R2R3-MYB	Bo6g100940.1	X	Bo1g019130.1	GroupII-b	Bo3g010100.1
ERF_B-6	Bo5g020790.1	II	Bo4g176430.1	R2R3-MYB	Bo2g168190.1	X	Bo7g105630.1	GroupI	Bo3g021100.1
AP2	Bo7g118400.1	II	Bo5g007170.1	1R-MYB	Bo9g157350.1	X	Bo7g075540.1	GroupI	Bo3g021460.1
DREB_A-6	Bo8g102130.1	II	Bo8g005940.1	R2R3-MYB	Bo5g143460.1	X	Bo2g130240.1	GroupI	Bo3g024450.1
ERF_B-4	Bo4g182140.1	II	Bo4g012700.1	R2R3-MYB	Bo8g091100.1	X	Bo8g028730.1	GroupII-d	Bo3g024750.1
ERF_B-5	Bo3g022760.1	II	Bo4g190320.1	1R-MYB	Bo2g066810.1	X	Bo9g071690.1	GroupI	Bo3g031650.1
ERF_B-3	Bo1g019600.1	II	Bo3g035070.1	R2R3-MYB	Bo1g051220.1	X	Bo7g080470.1	GroupI	Bo3g032500.1
ERF_B-6	Bo9g011440.1	II	Bo8g007220.1	R2R3-MYB	Bo3g009730.1	X	Bo2g136280.1	GroupIII	Bo3g035130.1
ERF_B-1	Bo2g011600.1	II	Bo8g114410.1	R2R3-MYB	Bo9g175680.1	X	Bo9g024360.1	GroupIII	Bo3g035140.1
DREB_A-5	Bo7g057680.1	II	Bo6g066380.1	R2R3-MYB	Bo6g116800.1	X	Bo3g092020.1	GroupII-c	Bo3g037400.1
ERF_B-5	Bo6g094430.1	II	Bo4g145800.1	1R-MYB	Bo01274s020.1	X	Bo7g015050.1	GroupIII	Bo3g039240.1
DREB_A-6	Bo4g149390.1	II	Bo8g101920.1	1R-MYB	Bo2g028820.1	X	Bo8g076410.1	GroupII-b	Bo3g048600.1
ERF_B-6	Bo9g152440.1	II	Bo7g119550.1	R2R3-MYB	Bo6g081140.1	X	Bo5g045190.1	GroupI	Bo3g055330.1
ERF_B-3	Bo9g059180.1	II	Bo3g149620.1	R2R3-MYB	Bo8g095790.1	X	Bo6g109250.1	GroupII-d	Bo3g058510.1
DREB_A-4	Bo3g030140.1	I	Bo9g024230.1	R2R3-MYB	Bo5g043390.1	X	Bo6g064260.1	GroupI	Bo3g085560.1
ERF_B-4	Bo3g004290.1	I	Bo3g091670.1	R2R3-MYB	Bo8g104300.1	X	Bo5g133050.1	GroupII-c	Bo3g100860.1
ERF_B-6	Bo8g106100.1	I	Bo1g006780.1	R2R3-MYB	Bo7g117880.1	X	Bo3g065200.1	GroupII-e	Bo3g143900.1
DREB_A-2	Bo4g190040.1	I	Bo3g167300.1	R2R3-MYB	Bo4g025810.1	X	Bo1g138800.1	GroupII-c	Bo3g150120.1
ERF_B-2	Bo3g184310.1	I	Bo8g081100.1	1R-MYB	Bo2g160810.1	X	Bo3g036290.1	GroupII-e	Bo3g165850.1
ERF_B-3	Bo9g069410.1	I	Bo4g125710.1	1R-MYB	Bo3g024610.1	X	Bo4g192870.1	GroupII-b	Bo3g173640.1
DREB_A-2	Bo5g136930.1	I	Bo3g099410.1	1R-MYB	Bo00877s020.1	X	Bo4g018490.1	GroupII-d	Bo3g173880.1
DREB_A-4	Bo1g009990.1	I	Bo00615s190.1	R2R3-MYB	Bo3g044210.1	IX	Bo1g143830.1	GroupI	Bo3g175320.1
AP2	Bo6g024850.1	I	Bo9g018570.1	R2R3-MYB	Bo2g025410.1	IX	Bo3g002800.1	GroupII-c	Bo3g180930.1
DREB_A-4	Bo6g046850.1	I	Bo9g173550.1	R2R3-MYB	Bo1g006820.1	IX	Bo9g180050.1	GroupII-c	Bo3g180960.1
AP2	Bo5g105180.1	I	Bo2g009670.1	1R-MYB	Bo8g094870.1	IX	Bo1g143840.1	GroupII-c	Bo3g184540.1
ERF_B-3	Bo4g024140.1	I	Bo5g035170.1	R2R3-MYB	Bo2g161170.1	IX	Bo1g143850.1	GroupII-c	Bo3g185390.1
DREB_A-2	Bo2g083920.1	I	Bo8g071300.1	R2R3-MYB	Bo1g017860.1	IX	Bo5g137360.1	GroupII-c	Bo3g185400.1
ERF_B-1	Bo3g066710.1	I	Bo6g081910.1	R2R3-MYB	Bo3g099390.1	IX	Bo3g062700.1	GroupII-c	Bo3g185420.1
DREB_A-6	Bo4g130860.1	I	Bo6g121590.1	R2R3-MYB	Bo7g092890.1	IX	Bo3g062710.1	GroupII-c	Bo8g049940.1
ERF_B-5	Bo8g096140.1	I	Bo9g175780.1	R2R3-MYB	Bo3g065150.1	VIII	Bo3g185870.1	GroupI	Bo8g051360.1
DREB_A-6	Bo5g069450.1	I	Bo3g004060.1	R2R3-MYB	Bo1g138990.1	VIII	Bo6g036040.1	GroupI	Bo8g065800.1
ERF_B-3	Bo5g004520.1	I	Bo5g008940.1	R2R3-MYB	Bo7g098590.1	VIII	Bo2g052630.1	GroupII-b	Bo8g068160.1
ERF	Bo9g141100.1	I	Bo8g112540.1	R2R3-MYB	Bo9g033970.1	VIII	Bo5g122710.1	GroupIII	Bo8g090820.1
RAV	Bo5g015210.1	I	Bo6g107140.1	1R-MYB	Bo8g092340.1	VIII	Bo9g161440.1	GroupII-e	Bo8g092840.1
AP2	Bo7g118740.1	I	Bo2g007460.1	R2R3-MYB	Bo8g082570.1	VIII	Bo5g150170.1	GroupII-c	Bo8g097780.1
AP2	Bo1g003450.1	I	Bo9g175710.1	R2R3-MYB	Bo7g090950.1	VIII	Bo1g154830.1	GroupII-c	Bo8g101460.1
DREB_A-6	Bo7g119000.1	I	Bo1g139470.1	1R-MYB	Bo8g104190.1	VIII	Bo3g012450.1	GroupII-b	Bo8g103030.1
ERF_B-1	Bo8g107890.1	I	Bo3g064830.1	1R-MYB	Bo2g011790.1	VII	Bo5g086780.1	GroupI	Bo2g007520.1
ERF_B-1	Bo6g022410.1	I	Bo5g133550.1	R2R3-MYB	Bo3g149450.1	VII	Bo3g109540.1	GroupII-b	Bo2g012390.1
DREB_A-2	Bo2g006960.1			1R-MYB	Bo4g002420.1	VII	Bo8g077840.1	GroupIII	Bo2g024040.1
AP2	Bo3g006680.1			R2R3-MYB	Bo8g071580.1	VII	Bo9g012590.1	GroupII-e	Bo2g047450.1
RAV	Bo8g107500.1			R2R3-MYB	Bo2g081100.1	VII	Bo7g096710.1	GroupII-b	Bo2g057610.1
ERF_B-6	Bo2g138790.1			R2R3-MYB	Bo4g187390.1	VI	Bo7g057730.1	GroupII-c	Bo2g062990.1
ERF_B-1	Bo3g009410.1			1R-MYB	Bo7g097510.1	VI	Bo5g085440.1	GroupII-b	Bo2g064630.1
DREB_A-2	Bo4g027470.1			1R-MYB	Bo8g068220.1	VI	Bo5g085430.1	GroupII-b	Bo2g095640.1
RAV	Bo5g043120.1			R2R3-MYB	Bo1g028520.1	VI	Bo6g044390.1	GroupIII	Bo2g095820.1
RAV	Bo6g022460.1			1R-MYB	Bo9g127350.1	VI	Bo8g027120.1	GroupIII	Bo2g109030.1
AP2	Bo6g124080.1			R2R3-MYB	Bo8g078940.1	VI	Bo2g010310.1	GroupII-c	Bo2g130010.1
DREB_A-6	Bo6g081850.1			1R-MYB	Bo7g101470.1	V	Bo1g063720.1	GroupI	Bo2g136900.1
DREB_A-2	Bo4g111040.1			R2R3-MYB	Bo3g004970.1	V	Bo3g016820.1	GroupII-c	Bo2g137260.1
ERF_B-1	Bo3g143350.1			R2R3-MYB	Bo9g008370.1	V	Bo2g023820.1	GroupII-c	Bo2g151740.1
ERF	Bo8g087280.1			R2R3-MYB	Bo3g107740.1	V	Bo4g075560.1	GroupII-d	Bo2g151880.1
ERF_B-3	Bo8g049470.1			R2R3-MYB	Bo1g082480.1	V	Bo3g064460.1	GroupII-c	Bo2g155960.1
ERF_B-1	Bo3g013220.1			R2R3-MYB	Bo6g087030.1	V	Bo3g101690.1	GroupII-c	Bo2g166420.1
DREB_A-6	Bo04101s010.1			1R-MYB	Bo2g161470.1	V	Bo2g009300.1		
ERF_B-5	Bo7g111760.1			1R-MYB	Bo2g013560.1	V	Bo9g174090.1		
DREB_A-4	Bo3g171090.1			R2R3-MYB	Bo1g040300.1	V	Bo3g005150.1		
DREB_A-6	Bo3g152610.1			1R-MYB	Bo9g003750.1	IV	Bo2g027080.1		
DREB_A-6	Bo7g111910.1			R2R3-MYB	Bo5g136560.1	IV	Bo2g027170.1		
DREB_A-4	Bo6g083410.1			1R-MYB	Bo7g098880.1	IV	Bo2g027140.1		
DREB_A-2	Bo1g141310.1			R2R3-MYB	Bo2g012170.1	IV	Bo2g027150.1		
ERF_B-4	Bo9g175390.1			1R-MYB	Bo8g102960.1	IV	Bo2g027110.1		
ERF_B-6	Bo7g096000.1			1R-MYB	Bo1g055180.1	IV	Bo2g027010.1		
DREB_A-3	Bo3g034810.1			R2R3-MYB	Bo1g147890.1	IV	Bo9g135860.1		
DREB_A-4	Bo2g010530.1			R2R3-MYB	Bo5g045110.1	IV	Bo9g135820.1		
RAV	Bo2g138650.1			1R-MYB	Bo4g197120.1	IV	Bo13819s010.1		
AP2	Bo3g153310.1			R2R3-MYB	Bo2g024590.1	IV	Bo9g135830.1		
ERF_B-6	Bo8g066410.1			R2R3-MYB	Bo2g013970.1	IV	Bo9g135840.1		

DREB_A-1	Bo3g024240.1			R2R3-MYB	Bo3g107600.1	IV	Bo5g002870.1		
ERF_B-3	Bo7g040820.1			R2R3-MYB	Bo5g133210.1	IV	Bo9g004070.1		
ERF_B-1	Bo8g107780.1			R2R3-MYB	Bo9g010860.1	IV	Bo9g061230.1		
ERF_B-1	Bo2g095830.1			1R-MYB	Bo5g034720.1	IV	Bo00853s060.1		
ERF_B-1	Bo5g016400.1			R2R3-MYB	Bo00835s060.1	IV	Bo9g004110.1		
ERF_B-5	Bo4g009400.1			1R-MYB	Bo3g054440.1	IV	Bo9g004100.1		
DREB_A-4	Bo6g094210.1			1R-MYB	Bo5g131980.1	IV	Bo9g004090.1		
DREB_A-4	Bo7g104770.1			1R-MYB	Bo3g168900.1	IV	Bo6g119080.1		
DREB_A-2	Bo9g155940.1			1R-MYB	Bo8g043610.1	IV	Bo3g122200.1		
ERF_B-3	Bo7g098860.1			R2R3-MYB	Bo3g091600.1	IV	Bo3g123480.1		
DREB_A-6	Bo9g030900.1			R2R3-MYB	Bo9g176900.1	IV	Bo9g148540.1		
ERF_B-4	Bo2g161710.1			1R-MYB	Bo9g178980.1	IV	Bo2g023870.1		
ERF_B-1	Bo3g143080.1			R2R3-MYB	Bo3g010230.1	IV	Bo3g016870.1		
DREB_A-4	Bo2g072910.1			1R-MYB	Bo4g031010.1	IV	Bo9g097190.1		
DREB_A-1	Bo01335s010.1			1R-MYB	Bo7g003580.1	IV	Bo4g112990.1		
ERF_B-3	Bo14558s010.1			1R-MYB	Bo3g094110.1	IV	Bo5g002830.1		
ERF	Bo3g035770.1			R2R3-MYB	Bo4g101470.1	IV	Bo5g002860.1		
ERF_B-2	Bo6g115850.1			R2R3-MYB	Bo9g076410.1	IV	Bo1g093090.1		
ERF_B-3	Bo9g014660.1			R2R3-MYB	Bo3g062160.1	IV	Bo3g081220.1		
ERF_B-6	Bo5g043190.1			R2R3-MYB	Bo9g147050.1	IV	Bo5g002810.1		
ERF_B-2	Bo3g068510.1			1R-MYB	Bo4g021680.1	IV	Bo5g002850.1		
ERF_B-4	Bo9g167820.1			1R-MYB	Bo8g068210.1	IV	Bo3g143160.1		
ERF_B-1	Bo3g185040.1			R2R3-MYB	Bo2g146540.1	IV	Bo5g002960.1		
DREB_A-2	Bo6g086320.1			1R-MYB	Bo3g002620.1	IV	Bo5g002890.1		
ERF_B-5	Bo7g056530.1			R2R3-MYB	Bo8g104600.1	IV	Bo2g070830.1		
DREB_A-1	Bo9g104770.1			1R-MYB	Bo9g019040.1	IV	Bo5g002900.1		
ERF_B-5	Bo2g045930.1			1R-MYB	Bo9g052030.1	IV	Bo8g117570.1		
ERF_B-1	Bo5g054550.1			R2R3-MYB	Bo2g161590.1	IV	Bo8g117560.1		
ERF_B-1	Bo5g055840.1			R2R3-MYB	Bo1g102190.1	IV	Bo5g002880.1		
ERF_B-3	Bo9g100030.1			R2R3-MYB	Bo4g176020.1	IV	Bo5g002930.1		
ERF_B-3	Bo1g017580.1			R2R3-MYB	Bo6g118860.1	IV	Bo5g002910.1		
ERF_B-1	Bo6g031240.1			R2R3-MYB	Bo7g117140.1	IV	Bo5g002950.1		
ERF_B-3	Bo2g161480.1			R2R3-MYB	Bo9g014980.1	III	Bo1g091580.1		
DREB_A-5	Bo9g149820.1			R2R3-MYB	Bo7g092680.1	III	Bo1g091800.1		
DREB_A-3	Bo4g025410.1			R2R3-MYB	Bo5g136570.1	III	Bo4g084880.1		
DREB_A-4	Bo4g158810.1			R2R3-MYB	Bo5g007320.1	III	Bo9g122180.1		
DREB_A-6	Bo1g051230.1			R2R3-MYB	Bo1g004900.1	III	Bo9g122170.1		
ERF_B-3	Bo4g052480.1			1R-MYB	Bo5g139340.1	III	Bo9g123430.1		
ERF	Bo3g040110.1			R2R3-MYB	Bo7g095130.1	II	Bo3g055070.1		
ERF_B-3	Bo7g077410.1			1R-MYB	Bo2g097680.1	II	Bo1g037810.1		
ERF	Bo9g160260.1			R2R3-MYB	Bo4g198510.1	II	Bo5g043100.1		
ERF_B-4	Bo2g166380.1			1R-MYB	Bo1g144410.1	II	Bo7g052860.1		
DREB_A-1	Bo5g016750.1			R2R3-MYB	Bo7g063830.1	II	Bo9g126190.1		
ERF	Bo6g068410.1			R2R3-MYB	Bo2g080900.1	II	Bo7g113660.1		
ERF_B-3	Bo1g017590.1			R2R3-MYB	Bo4g140330.1	II	Bo1g016630.1		
DREB_A-5	Bo7g118430.1			R2R3-MYB	Bo9g009020.1	II	Bo7g113170.1		
ERF_B-1	Bo7g048420.1			R2R3-MYB	Bo3g012180.1	II	Bo1g017410.1		
ERF	Bo2g025090.1			R2R3-MYB	Bo8g067590.1	II	Bo5g055990.1		
AP2	Bo6g067950.1			R2R3-MYB	Bo8g067910.1	II	Bo7g047040.1		
DREB_A-5	Bo6g121400.1			1R-MYB	Bo5g002430.1	I	Bo7g030700.1		
DREB_A-5	Bo6g086740.1			R2R3-MYB	Bo9g018910.1	I	Bo1g071020.1		
AP2	Bo9g160240.1			R2R3-MYB	Bo3g021790.1	I	Bo6g072200.1		
ERF_B-2	Bo6g091540.1			1R-MYB	Bo7g105690.1	I	Bo6g064960.1		
DREB_A-4	Bo4g034600.1			R2R3-MYB	Bo3g031390.1	I	Bo4g140840.1		
DREB_A-1	Bo7g110320.1			R2R3-MYB	Bo3g026690.1				
ERF_B-3	Bo4g195220.1			R2R3-MYB	Bo1g098860.1				
DREB_A-2	Bo3g013160.1			R2R3-MYB	Bo4g140210.1				
ERF_B-5	Bo1g037170.1			R2R3-MYB	Bo9g161810.1				
ERF_B-1	Bo1g108970.1			R2R3-MYB	Bo9g159830.1				
ERF_B-5	Bo8g053000.1			R2R3-MYB	Bo5g009290.1				
DREB_A-4	Bo8g027240.1			1R-MYB	Bo6g094990.1				
DREB_A-4	Bo6g077080.1			R2R3-MYB	Bo6g106730.1				
DREB_A-4	Bo3g023490.1			R2R3-MYB	Bo5g139830.1				
ERF_B-3	Bo4g176080.1			3R-MYB	Bo1g021790.1				
ERF_B-1	Bo3g182770.1			R2R3-MYB	Bo7g119670.1				
ERF_B-6	Bo7g111140.1			R2R3-MYB	Bo2g010970.1				
ERF_B-1	Bo2g122260.1			R2R3-MYB	Bo9g169270.1				
ERF_B-1	Bo7g050300.1			1R-MYB	Bo9g134990.1				
ERF_B-1	Bo3g074840.1			R2R3-MYB	Bo3g083130.1				
DREB_A-5	Bo7g059990.1			1R-MYB	Bo1g051550.1				
AP2	Bo2g032400.1			1R-MYB	Bo7g099090.1				
DREB_A-4	Bo4g036220.1			1R-MYB	Bo7g093690.1				
DREB_A-4	Bo5g126280.1			1R-MYB	Bo3g001090.1				
ERF_B-6	Bo9g171490.1			R2R3-MYB	Bo6g122640.1				
ERF_B-4	Bo3g109240.1			R2R3-MYB	Bo9g165670.1				
ERF_B-6	Bo4g157530.1			R2R3-MYB	Bo3g004500.1				
ERF_B-1	Bo5g004090.1			R2R3-MYB	Bo9g004210.1				

ERF_B-3	Bo5g007280.1			R2R3-MYB	Bo00685s060.1				
ERF_B-5	Bo8g071630.1			R2R3-MYB	Bo9g123440.1				
DREB_A-5	Bo8g071150.1			1R-MYB	Bo3g154100.1				
ERF_B-1	Bo3g140080.1			R2R3-MYB	Bo3g060620.1				
ERF_B-2	Bo3g066010.1			1R-MYB	Bo3g002160.1				
DREB_A-4	Bo8g064940.1			1R-MYB	Bo1g142610.1				
ERF_B-1	Bo9g154770.1			1R-MYB	Bo2g149310.1				
AP2	Bo1g004960.1			R2R3-MYB	Bo9g008700.1				
DREB_A-6	Bo8g054130.1			R2R3-MYB	Bo4g031070.1				
DREB_A-4	Bo3g037480.1			1R-MYB	Bo9g135730.1				
DREB_A-2	Bo2g016490.1			1R-MYB	Bo2g146330.1				
ERF_B-2	Bo1g123160.1			3R-MYB	Bo3g179620.1				
DREB_A-4	Bo7g115860.1			R2R3-MYB	Bo00954s080.1				
DREB_A-1	Bo8g050740.1			R2R3-MYB	Bo6g044070.1				
DREB_A-2	Bo6g073020.1			1R-MYB	Bo5g025310.1				
AP2	Bo2g009900.1			1R-MYB	Bo1g003590.1				
AP2	Bo2g076920.1			R2R3-MYB	Bo3g081880.1				
ERF_B-6	Bo7g096180.1			1R-MYB	Bo2g070770.1				
DREB_A-5	Bo6g119120.1			R2R3-MYB	Bo4g140220.1				
ERF_B-3	Bo7g077390.1			R2R3-MYB	Bo3g055110.1				
DREB_A-4	Bo4g195280.1			1R-MYB	Bo3g019630.1				
ERF_B-4	Bo2g011200.1			R2R3-MYB	Bo7g111870.1				
AP2	Bo9g171990.1			1R-MYB	Bo8g067280.1				
DREB_A-4	Bo8g117810.1			R2R3-MYB	Bo2g080540.1				
RAV	Bo3g138550.1			R2R3-MYB	Bo9g014380.1				
ERF_B-1	Bo9g166320.1			R2R3-MYB	Bo8g030520.1				
ERF_B-5	Bo1g051080.1			R2R3-MYB	Bo5g156410.1				
ERF_B-6	Bo8g051400.1			1R-MYB	Bo3g023120.1				
ERF_B-4	Bo3g009120.1			1R-MYB	Bo6g116150.1				
DREB_A-5	Bo4g151460.1			1R-MYB	Bo4g006930.1				
ERF_B-4	Bo3g100930.1			3R-MYB	Bo3g062020.1				
DREB_A-4	Bo9g104780.1			R2R3-MYB	Bo7g107420.1				
DREB_A-4	Bo7g095710.1			R2R3-MYB	Bo1g141120.1				
DREB_A-4	Bo4g186300.1			R2R3-MYB	Bo7g108060.1				
DREB_A-5	Bo8g102870.1			R2R3-MYB	Bo6g109070.1				
ERF_B-6	Bo9g011380.1			3R-MYB	Bo1g010040.1				
DREB_A-4	Bo9g170060.1			R2R3-MYB	Bo7g087050.1				
ERF_B-1	Bo2g016580.1			R2R3-MYB	Bo9g133340.1				
ERF_B-6	Bo7g082250.1			1R-MYB	Bo9g028250.1				
DREB_A-4	Bo9g011210.1			1R-MYB	Bo3g022870.1				
AP2	Bo3g099090.1			R2R3-MYB	Bo5g025570.1				
ERF_B-3	Bo9g175610.1			1R-MYB	Bo3g039720.1				
ERF_B-6	Bo7g111110.1			1R-MYB	Bo1g002450.1				
ERF	Bo8g033910.1			1R-MYB	Bo1g153240.1				
ERF_B-4	Bo4g043530.1			1R-MYB	Bo3g096520.1				
ERF_B-3	Bo8g115580.1			1R-MYB	Bo24301s010.1				
ERF_B-6	Bo3g013830.1			R2R3-MYB	Bo4g178040.1				
ERF_B-3	Bo9g069400.1			1R-MYB	Bo5g021890.1				
DREB_A-5	Bo8g019370.1			R2R3-MYB	Bo9g166000.1				
				R2R3-MYB	Bo7g098110.1				
				R2R3-MYB	Bo3g032160.1				
				R2R3-MYB	Bo6g118350.1				
				R2R3-MYB	Bo6g097180.1				
				R2R3-MYB	Bo7g091360.1				
				R2R3-MYB	Bo1g138940.1				
				1R-MYB	Bo01274s030.1				
				R2R3-MYB	Bo7g099180.1				
				1R-MYB	Bo9g003580.1				
				R2R3-MYB	Bo7g057770.1				
				R2R3-MYB	Bo5g155050.1				
				1R-MYB	Bo7g085920.1				
				R2R3-MYB	Bo6g077030.1				
				1R-MYB	Bo9g110930.1				
				R2R3-MYB	Bo2g009130.1				
				R2R3-MYB	Bo4g140000.1				
				1R-MYB	Bo03161s010.1				
				1R-MYB	Bo4g101460.1				
				R2R3-MYB	Bo2g151810.1				
				R2R3-MYB	Bo3g012780.1				
				R2R3-MYB	Bo6g086910.1				
				1R-MYB	Bo5g126180.1				
				R2R3-MYB	Bo2g164160.1				
				R2R3-MYB	Bo4g186460.1				
				R2R3-MYB	Bo6g095400.1				
				R2R3-MYB	Bo8g091080.1				
				R2R3-MYB	Bo2g002350.1				
				R2R3-MYB	Bo3g064180.1				

				R2R3-MYB	Bo4g140150.1				
				1R-MYB	Bo4g030640.1				
				1R-MYB	Bo8g080840.1				
				1R-MYB	Bo5g023860.1				
				1R-MYB	Bo3g039260.1				
				R2R3-MYB	Bo8g077360.1				
				R2R3-MYB	Bo4g176030.1				
				R2R3-MYB	Bo6g077600.1				
				R2R3-MYB	Bo4g029810.1				
				1R-MYB	Bo3g018460.1				
				R2R3-MYB	Bo9g029530.1				
				1R-MYB	Bo1g005570.1				
				R2R3-MYB	Bo8g058040.1				
				R2R3-MYB	Bo8g079430.1				
				1R-MYB	Bo6g091360.1				
				1R-MYB	Bo1g087910.1				
				R2R3-MYB	Bo2g156300.1				
				R2R3-MYB	Bo7g088650.1				
				1R-MYB	Bo2g098940.1				
				1R-MYB	Bo3g017760.1				
				1R-MYB	Bo7g105100.1				
				R2R3-MYB	Bo5g025790.1				
				1R-MYB	Bo5g137250.1				
				R2R3-MYB	Bo5g136680.1				
				4R-MYB	Bo00828s010.1				
				3R-MYB	Bo8g104210.1				
				1R-MYB	Bo8g084780.1				
				1R-MYB	Bo2g132340.1				
				1R-MYB	Bo1g081300.1				
				R2R3-MYB	Bo2g013190.1				
				R2R3-MYB	Bo7g040780.1				
				R2R3-MYB	Bo8g068030.1				
				1R-MYB	Bo5g077530.1				
				R2R3-MYB	Bo3g061630.1				
				R2R3-MYB	Bo7g110400.1				
				1R-MYB	Bo4g106310.1				
				R2R3-MYB	Bo7g105430.1				
				R2R3-MYB	Bo9g119060.1				
				R2R3-MYB	Bo1g157620.1				
				R2R3-MYB	Bo4g076940.1				
				R2R3-MYB	Bo3g130340.1				
				1R-MYB	Bo5g146770.1				
				1R-MYB	Bo2g046050.1				
				R2R3-MYB	Bo3g185830.1				
				R2R3-MYB	Bo3g097710.1				
				1R-MYB	Bo4g009260.1				
				1R-MYB	Bo1g007270.1				
				R2R3-MYB	Bo1g003420.1				
				1R-MYB	Bo8g071010.1				
				R2R3-MYB	Bo2g161180.1				
				1R-MYB	Bo5g141210.1				
				1R-MYB	Bo6g119010.1				
				R2R3-MYB	Bo3g002960.1				
				R2R3-MYB	Bo7g033260.1				
				1R-MYB	Bo9g161410.1				
				1R-MYB	Bo2g082370.1				
				1R-MYB	Bo9g183320.1				
				R2R3-MYB	Bo2g167960.1				
				R2R3-MYB	Bo6g067190.1				
				R2R3-MYB	Bo4g004500.1				
The identified five families of TFs in A thaliana									
Subfamily	AtAP2/EREBP	Subfami	AtbZIP	Subfamily	AtMYB	Subfamily	AtNAC	Subfamily	AtWRKY
ERF_B-4	AT2G33710.2	VI	AT3G12250.2	R2R3-MYB	AT5G56110.1	XIV	AT1G52880.1	GroupII-c	AT1G29860.1
AP2	AT5G10510.1	VI	AT3G12250.4	3R-MYB	AT4G32730.2	XIV	AT3G15510.1	GroupII-e	AT1G29280.1
ERF_B-6	AT2G41710.3	VI	AT3G12250.1	R2R3-MYB	AT3G29020.1	XIV	AT1G61110.1	GroupII-c	AT1G55600.1
RAV	AT1G13260.1	VI	AT3G12250.3	R2R3-MYB	AT3G13890.2	XIV	AT5G13180.1	GroupII-b	AT1G80840.1
ERF_B-5	AT4G27950.1	VI	AT3G12250.5	1R-MYB	AT1G18330.1	XIV	AT2G33480.1	GroupII-b	AT1G18860.1
DREB_A-6	AT5G65130.1	VI	AT5G06950.1	R2R3-MYB	AT3G11450.1	XIV	AT2G33480.2	GroupII-b	AT1G69810.1
ERF_B-1	AT3G20310.1	VI	AT5G06950.2	1R-MYB	AT1G72740.1	XIV	AT1G69490.1	GroupI	AT1G13960.1
DREB_A-4	AT2G44940.1	VI	AT5G06950.3	1R-MYB	AT4G00540.2	XIV	AT3G04070.1	GroupII-c	AT1G69310.1
DREB_A-5	AT4G36900.1	VI	AT5G06950.4	R2R3-MYB	AT4G38620.1	XIV	AT3G04070.2	GroupII-c	AT1G64000.1
ERF_B-6	AT3G25890.1	VI	AT5G06960.1	1R-MYB	AT2G36960.2	XIII	AT1G77450.1	GroupIII	AT1G80590.1
ERF_B-1	AT1G50640.1	VI	AT5G06960.2	1R-MYB	AT1G18960.1	XIII	AT1G01720.1	GroupII-e	AT1G30650.1
ERF_B-3	AT4G17500.1	VI	AT1G68640.1	1R-MYB	AT3G10590.1	XIII	AT5G63790.1	GroupII-b	AT1G62300.1
AP2	AT1G16060.1	VI	AT1G08320.1	R2R3-MYB	AT3G13540.1	XIII	AT5G08790.1	GroupII-b	AT1G68150.1
ERF_B-2	AT1G72360.1	VI	AT1G08320.3	1R-MYB	AT4G18020.2	XIII	AT1G52890.1	GroupIII	AT1G66550.1
ERF_B-6	AT1G79700.1	VI	AT1G08320.2	4R-MYB	AT3G18100.1	XIII	AT3G15500.1	GroupIII	AT1G66560.1

DREB_A-4	AT5G52020.1	VI	AT5G06839.1	R2R3-MYB	AT1G26780.1	XIII	AT4G27410.2	GroupIII	AT1G66600.1
DREB_A-6	AT1G64380.1	VI	AT5G06839.2	1R-MYB	AT5G53200.1	XIII	AT4G27410.3	GroupII-c	AT1G69310.2
DREB_A-6	AT2G20880.1	VI	AT5G06839.3	R2R3-MYB	AT1G25340.2	XIII	AT5G64530.1	GroupI	AT1G13960.2
DREB_A-6	AT2G22200.1	V	AT1G77920.1	R2R3-MYB	AT3G23250.1	XIII	AT1G76420.1	GroupIII	AT1G66550.2
ERF_B-6	AT2G41710.1	V	AT1G22070.1	R2R3-MYB	AT5G16600.1	XII	AT2G24430.1	GroupI	AT2G03340.1
ERF_B-4	AT5G50080.1	V	AT5G10030.1	R2R3-MYB	AT4G01680.2	XII	AT2G24430.2	GroupII-e	AT2G34830.1
ERF_B-6	AT1G16060.2	V	AT5G10030.2	3R-MYB	AT4G32730.1	XII	AT3G15170.1	GroupII-b	AT2G25000.1
DREB_A-5	AT1G19210.1	V	AT5G65210.6	1R-MYB	AT2G46830.1	XII	AT5G53950.1	GroupII-c	AT2G44745.1
DREB_A-5	AT1G74930.1	V	AT5G65210.2	R2R3-MYB	AT2G23290.1	XII	AT3G18400.1	GroupII-d	AT2G30590.1
DREB_A-4	AT1G33760.1	V	AT5G65210.1	R2R3-MYB	AT2G39880.1	XII	AT5G18270.1	GroupI	AT2G37260.1
DREB_A-4	AT4G32800.1	V	AT5G65210.3	R2R3-MYB	AT3G47600.1	XII	AT5G18270.2	GroupII-c	AT2G46130.1
ERF_B-3	AT5G61590.1	V	AT5G65210.4	1R-MYB	AT3G46130.3	XII	AT3G04060.1	GroupIII	AT2G40740.1
ERF_B-6	AT5G11190.1	V	AT5G65210.5	1R-MYB	AT5G02840.2	XII	AT3G29035.1	GroupIII	AT2G40750.1
ERF_B-6	AT4G13040.1	IV	AT2G16770.1	R2R3-MYB	AT1G74430.1	XII	AT5G39610.1	GroupII-c	AT2G47260.1
DREB_A-5	AT5G67190.1	IV	AT4G35040.1	1R-MYB	AT2G36960.1	XII	AT5G61430.1	GroupI	AT2G04880.1
ERF_B-5	AT1G71130.1	IV	AT3G51960.2	R2R3-MYB	AT4G21440.1	XII	AT5G07680.1	GroupIII	AT2G46400.1
DREB_A-1	AT1G63030.2	IV	AT3G51960.1	R2R3-MYB	AT1G63910.1	XII	AT5G07680.2	GroupI	AT2G38470.1
DREB_A-3	AT2G40220.1	III	AT5G49450.1	R2R3-MYB	AT5G52600.1	XI	AT4G28530.1	GroupII-c	AT2G21900.1
DREB_A-5	AT3G50260.1	III	AT5G24800.1	R2R3-MYB	AT3G46130.1	XI	AT4G28530.2	GroupII-d	AT2G24570.1
ERF_B-5	AT4G11140.1	III	AT2G42380.1	1R-MYB	AT4G39250.1	XI	AT3G12977.1	GroupII-d	AT2G23320.1
ERF_B-2	AT3G16770.1	III	AT2G42380.2	3R-MYB	AT5G11510.2	XI	AT1G56010.1	GroupI	AT2G30250.1
DREB_A-5	AT4G06746.1	III	AT3G58120.1	R2R3-MYB	AT5G40330.1	XI	AT1G56010.2	GroupII-c	AT2G46130.2
ERF_B-4	AT2G33710.1	III	AT2G13150.1	1R-MYB	AT3G04450.1	X	AT3G03200.1	GroupI	AT2G04880.2
ERF_B-6	AT1G68550.2	III	AT2G12940.1	R2R3-MYB	AT4G22680.1	X	AT5G17260.1	GroupI	AT2G37260.2
ERF_B-1	AT1G28360.1	III	AT2G12900.1	1R-MYB	AT4G18020.1	X	AT3G17730.1	GroupII-c	AT3G62340.1
DREB_A-2	AT5G05410.2	III	AT1G43700.1	R2R3-MYB	AT3G49690.1	X	AT1G65910.1	GroupIII	AT3G56400.1
ERF_B-1	AT1G24590.1	III	AT2G21230.1	1R-MYB	AT2G18328.1	X	AT1G54330.1	GroupI	AT3G01080.1
DREB_A-5	AT4G31060.1	III	AT2G21230.3	R2R3-MYB	AT3G48920.1	X	AT1G32510.1	GroupII-c	AT3G01970.1
ERF_B-4	AT5G07310.1	III	AT2G21230.2	1R-MYB	AT4G16420.1	X	AT4G17980.1	GroupII-e	AT3G58710.1
ERF_B-3	AT5G51190.1	III	AT4G38900.2	1R-MYB	AT3G53790.1	X	AT5G46590.1	GroupII-d	AT3G04670.1
ERF_B-6	AT1G49120.1	III	AT4G38900.1	R2R3-MYB	AT1G09540.1	X	AT2G18060.1	GroupII-e	AT3G58710.2
AP2	AT5G17430.1	III	AT4G38900.3	R2R3-MYB	AT4G37260.1	X	AT4G36160.1	GroupII-b	AT4G04450.1
ERF_B-1	AT1G53170.1	III	AT1G06850.1	R2R3-MYB	AT5G57620.1	X	AT5G66300.1	GroupII-e	AT4G23550.1
DREB_A-6	AT4G39780.1	III	AT1G06850.2	R2R3-MYB	AT1G69560.1	X	AT1G62700.1	GroupI	AT4G26440.1
ERF_B-4	AT5G64750.1	III	AT2G40620.1	R2R3-MYB	AT5G14750.1	X	AT1G12260.1	GroupII-c	AT4G39410.1
DREB_A-2	AT2G38340.1	III	AT1G06070.1	1R-MYB	AT5G18240.5	X	AT5G62380.1	GroupII-b	AT4G01720.1
ERF_B-2	AT1G53910.3	III	AT2G31370.5	R2R3-MYB	AT1G56160.1	X	AT1G71930.1	GroupI	AT4G26640.1
ERF_B-2	AT1G72360.2	III	AT2G31370.3	1R-MYB	AT3G09370.2	X	AT2G46770.1	GroupIII	AT4G23810.1
ERF_B-6	AT5G25190.1	III	AT2G31370.2	R2R3-MYB	AT5G60890.1	X	AT3G61910.1	GroupII-c	AT4G18170.1
ERF_B-3	AT5G47220.1	III	AT2G31370.1	1R-MYB	AT1G19000.2	X	AT1G32770.1	GroupII-d	AT4G31550.1
ERF_B-3	AT3G23220.1	III	AT2G31370.4	R2R3-MYB	AT5G17800.1	X	AT1G33280.1	GroupII-b	AT4G31800.1
ERF_B-6	AT3G54990.1	III	AT2G31370.6	R2R3-MYB	AT5G54230.1	X	AT4G10350.1	GroupI	AT4G30935.1
RAV	AT1G25560.1	II	AT1G19490.1	R2R3-MYB	AT5G23650.1	X	AT1G79580.1	GroupII-e	AT4G01250.1
AP2	AT3G54320.1	II	AT3G10800.1	1R-MYB	AT5G59780.2	X	AT1G79580.2	GroupIII	AT4G11070.1
ERF_B-6	AT5G60120.1	II	AT2G40950.1	1R-MYB	AT5G56840.1	X	AT1G79580.3	GroupII-b	AT4G22070.1
ERF_B-1	AT3G15210.1	II	AT3G56660.1	R2R3-MYB	AT1G68320.1	IX	AT1G26870.1	GroupII-d	AT4G24240.1
AP2	AT5G67180.1	I	AT1G49720.1	R2R3-MYB	AT1G16490.1	IX	AT5G39820.1	GroupII-d	AT4G31550.2
ERF_B-1	AT1G28160.1	I	AT1G49720.2	R2R3-MYB	AT3G11440.1	IX	AT2G43000.1	GroupIII	AT4G11070.2
ERF_B-5	AT1G22985.1	I	AT3G19290.1	1R-MYB	AT3G04030.2	IX	AT3G12910.1	GroupI	AT4G26640.2
ERF_B-6	AT2G39250.1	I	AT3G19290.3	1R-MYB	AT4G16420.2	VIII	AT3G10480.1	GroupII-b	AT4G31800.2
DREB_A-5	AT1G71520.1	I	AT4G34000.3	1R-MYB	AT4G34430.3	VIII	AT3G10480.3	GroupI	AT5G56270.1
ERF_B-1	AT1G12890.1	I	AT4G34000.1	1R-MYB	AT1G01060.3	VIII	AT3G10480.2	GroupIII	AT5G01900.1
DREB_A-1	AT4G25470.1	I	AT4G34000.2	R2R3-MYB	AT1G18710.1	VIII	AT3G10490.1	GroupII-c	AT5G49520.1
RAV	AT3G25730.1	I	AT1G45249.1	1R-MYB	AT5G47390.1	VIII	AT3G10490.2	GroupII-d	AT5G28650.1
RAV	AT1G68840.2	I	AT3G56850.1	R2R3-MYB	AT1G74650.1	VIII	AT5G04400.1	GroupIII	AT5G22570.1
AP2	AT3G20840.1	I	AT2G41070.1	1R-MYB	AT5G61620.1	VIII	AT1G34180.1	GroupII-e	AT5G52830.1
DREB_A-4	AT2G25820.1	I	AT2G41070.3	R2R3-MYB	AT2G32460.1	VIII	AT1G34180.2	GroupII-c	AT5G26170.1
ERF_B-6	AT2G28550.1	I	AT2G41070.2	R2R3-MYB	AT5G14340.1	VIII	AT1G34190.1	GroupII-c	AT5G46350.1
DREB_A-4	AT3G60490.1	I	AT2G36270.1	R2R3-MYB	AT5G62470.1	VIII	AT1G32870.1	GroupII-c	AT5G13080.1
ERF_B-6	AT4G13040.2	I	AT3G44460.1	R2R3-MYB	AT4G12350.1	VIII	AT1G32870.2	GroupII-c	AT5G43290.1
ERF_B-3	AT5G61600.1	I	AT5G42910.1	1R-MYB	AT5G18240.4	VIII	AT3G10500.1	GroupI	AT5G07100.1
DREB_A-2	AT2G40350.1	I	AT1G03970.1	1R-MYB	AT3G46130.4	VIII	AT5G04410.1	GroupII-c	AT5G41570.1
ERF_B-3	AT5G07580.1	I	AT5G44080.1	1R-MYB	AT5G67580.1	VIII	AT5G64060.1	GroupII-b	AT5G15130.1
ERF_B-3	AT2G44840.1	I	AT2G17770.2	1R-MYB	AT5G67580.2	VIII	AT5G09330.1	GroupIII	AT5G24110.1
ERF_B-4	AT5G13330.1	I	AT4G35900.1	R2R3-MYB	AT3G12720.1	VIII	AT5G09330.2	GroupII-e	AT5G45050.1
ERF_B-3	AT5G47230.1	I	AT5G11260.1	1R-MYB	AT3G10113.1	VIII	AT5G09330.3	GroupII-c	AT5G64810.1
DREB_A-2	AT2G40340.1	I	AT3G17609.3	1R-MYB	AT1G49950.1	VIII	AT5G09330.4	GroupII-e	AT5G45260.1
AP2	AT4G36920.1	I	AT3G17609.4	R2R3-MYB	AT5G05790.1	VII	AT5G14000.1	GroupII-e	AT5G45050.2
AP2	AT5G65510.1	I	AT3G17609.1	1R-MYB	AT1G49950.2	VII	AT5G50820.1	GroupI	AT5G07100.2
DREB_A-5	AT1G46768.1	I	AT3G17609.2	R2R3-MYB	AT5G04760.1	VII	AT2G02450.1		
ERF_B-5	AT3G61630.1	I	AT1G42990.1	R2R3-MYB	AT2G26960.1	VII	AT2G02450.2		
DREB_A-2	AT1G75490.1	I	AT5G07160.1	1R-MYB	AT1G49950.3	VII	AT2G17040.1		
ERF_B-2	AT1G72360.3	I	AT3G54620.1	1R-MYB	AT1G19000.1	VII	AT5G22380.1		
DREB_A-4	AT5G25810.1	I	AT3G54620.3	R2R3-MYB	AT3G62610.1	VII	AT3G44350.1		
DREB_A-4	AT4G16750.1	I	AT3G54620.2	R2R3-MYB	AT5G06100.2	VII	AT3G44350.2		
DREB_A-4	AT3G16280.1	I	AT4G02640.1	1R-MYB	AT5G17300.1	VI	AT3G44290.1		
ERF_B-3	AT4G34410.1	I	AT4G02640.2	R2R3-MYB	AT3G50060.1	VI	AT5G22290.1		

ERF_B-6	AT3G54320.2	I	AT5G28770.3	1R-MYB	AT4G16420.3	VI	AT2G27300.1		
DREB_A-6	AT1G22190.1	I	AT5G28770.1	1R-MYB	AT1G18330.2	VI	AT3G49530.1		
DREB_A-1	AT4G25480.1	I	AT5G28770.2	R2R3-MYB	AT2G47460.1	VI	AT5G24590.2		
ERF_B-6	AT5G25390.1	I	AT1G32150.1	1R-MYB	AT3G07740.1	VI	AT1G33060.1		
ERF_B-3	AT2G31230.1	I	AT2G35530.1	1R-MYB	AT4G18020.4	VI	AT1G33060.2		
ERF_B-6	AT1G15360.1	I	AT4G36730.1	1R-MYB	AT3G04030.1	VI	AT4G35580.1		
AP2	AT3G54320.3	I	AT4G36730.2	1R-MYB	AT5G52660.1	VI	AT4G35580.2		
DREB_A-1	AT1G12610.1	I	AT4G01120.1	1R-MYB	AT1G72650.1	VI	AT4G35580.3		
ERF_B-2	AT3G14230.1	I	AT2G46270.1	R2R3-MYB	AT3G29020.2	V	AT1G64105.1		
DREB_A-4	AT1G71450.1	I	AT2G46270.2	R2R3-MYB	AT5G61420.2	V	AT1G64100.2		
DREB_A-2	AT3G57600.1	I	AT5G08141.1	1R-MYB	AT3G10580.2	V	AT5G18300.1		
ERF_B-2	AT3G14230.2	I	AT5G60830.1	R2R3-MYB	AT1G79180.1	V	AT1G60240.1		
DREB_A-5	AT1G44830.1	I	AT2G22850.1	R2R3-MYB	AT1G66230.1	V	AT1G60280.1		
DREB_A-6	AT4G13620.1	I	AT2G22850.2	R2R3-MYB	AT2G25230.1	V	AT1G60380.1		
ERF_B-1	AT1G12980.1	I	AT4G37730.1	R2R3-MYB	AT5G15310.1	V	AT1G60350.1		
DREB_A-5	AT2G23340.1	I	AT3G49760.1	R2R3-MYB	AT2G16720.1	V	AT1G60340.1		
ERF_B-6	AT5G19790.1	I	AT1G59530.1	1R-MYB	AT3G60460.1	V	AT1G60300.1		
DREB_A-1	AT5G51990.1	I	AT1G75390.1	R2R3-MYB	AT2G31180.1	IV	AT4G01540.1		
ERF_B-4	AT1G43160.1	I	AT1G75390.2	1R-MYB	AT1G74840.2	IV	AT4G01540.2		
ERF_B-6	AT5G60120.2	I	AT4G34590.1	R2R3-MYB	AT3G28910.1	IV	AT4G01520.1		
ERF_B-1	AT1G03800.1	I	AT2G18160.1	R2R3-MYB	AT3G06490.1	IV	AT4G01550.1		
DREB_A-6	AT1G36060.1	I	AT3G62420.1	1R-MYB	AT1G21700.1	IV	AT3G04430.1		
AP2	AT1G51190.1	I	AT1G68880.1	1R-MYB	AT3G10580.1	IV	AT1G01010.1		
ERF_B-3	AT4G18450.1	I	AT1G13600.1	R2R3-MYB	AT1G66390.1	IV	AT1G02210.1		
DREB_A-6	AT4G28140.1	I	AT2G04038.1	1R-MYB	AT1G49010.1	IV	AT1G02250.1		
DREB_A-4	AT1G01250.1	I	AT5G15830.1	R2R3-MYB	AT5G62470.2	IV	AT1G02230.1		
DREB_A-6	AT1G78080.1	I	AT3G30530.1	1R-MYB	AT4G18020.5	IV	AT3G04410.1		
ERF_B-6	AT3G25890.2	I	AT5G38800.1	1R-MYB	AT4G01280.2	IV	AT1G02220.1		
ERF_B-1	AT5G18560.1			R2R3-MYB	AT5G15310.2	IV	AT3G04420.1		
ERF_B-6	AT2G41710.2			1R-MYB	AT1G17460.2	IV	AT3G04420.2		
DREB_A-2	AT5G05410.1			1R-MYB	AT1G01380.1	III	AT3G01600.1		
ERF_B-3	AT3G23240.1			1R-MYB	AT1G15720.1	III	AT5G14490.1		
AP2	AT5G57390.1			R2R3-MYB	AT5G06100.3	III	AT1G25580.1		
DREB_A-5	AT1G22810.1			R2R3-MYB	AT1G73410.1	III	AT1G28470.1		
ERF_B-3	AT5G43410.1			R2R3-MYB	AT2G26950.1	III	AT4G28500.1		
ERF_B-6	AT4G13040.3			1R-MYB	AT1G09710.2	III	AT4G29230.1		
ERF_B-1	AT5G13910.1			R2R3-MYB	AT3G61250.1	III	AT5G56620.1		
ERF_B-3	AT3G23230.1			1R-MYB	AT4G34430.1	II	AT1G03490.1		
DREB_A-4	AT2G35700.1			1R-MYB	AT4G16110.1	II	AT1G19040.1		
ERF_B-5	AT4G23750.1			1R-MYB	AT5G29000.3	II	AT3G56520.1		
DREB_A-1	AT4G25490.1			R2R3-MYB	AT4G37780.1	II	AT5G41090.1		
ERF_B-5	AT2G46310.1			R2R3-MYB	AT2G47190.1	I	AT3G56560.1		
ERF_B-6	AT1G68550.1			1R-MYB	AT1G17460.1	I	AT3G56530.1		
DREB_A-2	AT5G18450.1			R2R3-MYB	AT5G07700.1	I	AT3G55210.1		
ERF_B-3	AT4G17490.1			1R-MYB	AT2G36960.3	I	AT5G39690.1		
ERF_B-2	AT2G47520.1			3R-MYB	AT5G11510.1				
DREB_A-5	AT5G21960.1			1R-MYB	AT5G06110.1				
ERF_B-6	AT5G67010.1			R2R3-MYB	AT5G01200.1				
ERF_B-5	AT5G53290.1			3R-MYB	AT5G02320.2				
ERF_B-6	AT5G10510.3			R2R3-MYB	AT4G01680.1				
DREB_A-5	AT1G77640.1			R2R3-MYB	AT3G01140.1				
AP2	AT5G10510.2			1R-MYB	AT5G58340.2				
ERF_B-2	AT3G14230.3			R2R3-MYB	AT3G55730.1				
AP2	AT4G37750.1			1R-MYB	AT5G18240.2				
ERF_B-1	AT5G44210.1			R2R3-MYB	AT3G08500.1				
DREB_A-1	AT1G63030.1			1R-MYB	AT1G58220.1				
DREB_A-4	AT1G12630.1			R2R3-MYB	AT5G39700.1				
ERF_B-3	AT1G04370.1			R2R3-MYB	AT5G65230.1				
AP2	AT4G36920.2			1R-MYB	AT5G29000.4				
ERF_B-5	AT4G23750.2			1R-MYB	AT2G47620.1				
DREB_A-4	AT5G11590.1			1R-MYB	AT3G21430.2				
DREB_A-4	AT2G36450.1			R2R3-MYB	AT4G17785.1				
ERF_B-1	AT1G80580.1			1R-MYB	AT2G13960.1				
RAV	AT1G68840.1			R2R3-MYB	AT1G25340.1				
AP2	AT2G28550.3			R2R3-MYB	AT1G18570.1				
ERF_B-1	AT1G28370.1			R2R3-MYB	AT4G26930.1				
AP2	AT1G72570.1			R2R3-MYB	AT5G16770.2				
ERF_B-4	AT5G61890.1			1R-MYB	AT2G30420.1				
ERF_B-2	AT1G53910.2			1R-MYB	AT3G57980.1				
ERF_B-6	AT2G28550.2			R2R3-MYB	AT1G48000.1				
DREB_A-2	AT3G11020.1			1R-MYB	AT3G09600.2				
DREB_A-5	AT1G21910.1			R2R3-MYB	AT4G01680.3				
AP2	AT1G79700.2			1R-MYB	AT1G01520.1				
DREB_A-4	AT1G77200.1			R2R3-MYB	AT3G12820.1				
ERF_B-2	AT1G53910.1			1R-MYB	AT5G06110.2				
ERF_B-6	AT3G54990.2			1R-MYB	AT4G09450.1				
ERF_B-3	AT1G06160.1			R2R3-MYB	AT5G35550.1				

ERF_B-6	AT5G25390.2			R2R3-MYB	AT3G01530.1				
				1R-MYB	AT5G58900.1				
				1R-MYB	AT1G72740.2				
				R2R3-MYB	AT5G26660.1				
				R2R3-MYB	AT4G34990.1				
				R2R3-MYB	AT1G35515.1				
				1R-MYB	AT5G29000.1				
				R2R3-MYB	AT2G32460.2				
				1R-MYB	AT1G70000.1				
				1R-MYB	AT5G18240.1				
				R2R3-MYB	AT3G02940.1				
				1R-MYB	AT1G01060.4				
				1R-MYB	AT2G42150.1				
				R2R3-MYB	AT5G49620.1				
				R2R3-MYB	AT3G24310.1				
				R2R3-MYB	AT4G28110.1				
				1R-MYB	AT1G01060.2				
				R2R3-MYB	AT5G40350.1				
				R2R3-MYB	AT4G13480.1				
				1R-MYB	AT3G16857.1				
				R2R3-MYB	AT1G56650.1				
				R2R3-MYB	AT5G62320.1				
				1R-MYB	AT5G02840.3				
				1R-MYB	AT1G71030.1				
				R2R3-MYB	AT3G27920.1				
				1R-MYB	AT3G09370.1				
				R2R3-MYB	AT5G07690.1				
				R2R3-MYB	AT3G13890.1				
				1R-MYB	AT4G34430.4				
				R2R3-MYB	AT5G49620.2				
				R2R3-MYB	AT1G17950.1				
				R2R3-MYB	AT1G06180.1				
				R2R3-MYB	AT2G38090.1				
				R2R3-MYB	AT5G16770.1				
				1R-MYB	AT3G16857.2				
				R2R3-MYB	AT5G55020.1				
				1R-MYB	AT4G18020.6				
				R2R3-MYB	AT1G66370.1				
				R2R3-MYB	AT4G05100.1				
				R2R3-MYB	AT3G11280.2				
				R2R3-MYB	AT3G52250.1				
				R2R3-MYB	AT2G36890.1				
				R2R3-MYB	AT5G67300.1				
				R2R3-MYB	AT1G34670.1				
				1R-MYB	AT5G29000.2				
				R2R3-MYB	AT5G06100.1				
				1R-MYB	AT2G30432.1				
				1R-MYB	AT1G70000.2				
				1R-MYB	AT4G00540.1				
				1R-MYB	AT3G07740.4				
				R2R3-MYB	AT5G59780.3				
				4R-MYB	AT3G18100.2				
				1R-MYB	AT3G09600.1				
				1R-MYB	AT1G72650.2				
				R2R3-MYB	AT1G08810.1				
				1R-MYB	AT3G07740.3				
				1R-MYB	AT3G07740.2				
				R2R3-MYB	AT3G53200.1				
				R2R3-MYB	AT5G10280.1				
				1R-MYB	AT4G34430.2				
				1R-MYB	AT3G60110.1				
				R2R3-MYB	AT1G26780.2				
				R2R3-MYB	AT3G30210.1				
				1R-MYB	AT5G37260.1				
				R2R3-MYB	AT5G65790.1				
				1R-MYB	AT5G18240.3				
				1R-MYB	AT5G58340.1				
				1R-MYB	AT4G01280.1				
				R2R3-MYB	AT3G27810.1				
				R2R3-MYB	AT1G74080.1				
				1R-MYB	AT1G01060.1				
				1R-MYB	AT5G02840.1				
				R2R3-MYB	AT4G09460.1				
				R2R3-MYB	AT5G52260.1				
				1R-MYB	AT2G30424.1				
				R2R3-MYB	AT5G12870.1				
				R2R3-MYB	AT5G23000.1				

				1R-MYB	AT2G46410.1				
				R2R3-MYB	AT3G28470.1				
				R2R3-MYB	AT5G49330.1				
				R2R3-MYB	AT5G08520.1				
				1R-MYB	AT3G16350.1				
				1R-MYB	AT1G09710.1				
				R2R3-MYB	AT4G25560.1				
				R2R3-MYB	AT1G57560.1				
				1R-MYB	AT1G74840.1				
				R2R3-MYB	AT3G11280.1				
				1R-MYB	AT3G49850.1				
				1R-MYB	AT2G13960.2				
				1R-MYB	AT5G52660.2				
				3R-MYB	AT5G02320.1				
				1R-MYB	AT4G18020.3				
				1R-MYB	AT2G33610.1				
				R2R3-MYB	AT1G22640.1				
				4R-MYB	AT3G18100.3				
				1R-MYB	AT3G04030.3				
				1R-MYB	AT3G23250.2				
				R2R3-MYB	AT1G66380.1				
				R2R3-MYB	AT3G09230.1				
				1R-MYB	AT2G44430.1				
