# Supplementary material for: Exploring transcription factors reveals crucial members and regulatory networks involved in different abiotic stresses in Brassica napus L
Source: BMC Plant Biol. 2018 Sep 19;18:202. doi: 10.1186/s12870-018-1417-z (PMC6146658; doi:10.1186/s12870-018-1417-z)
Supplement: Supplementary file 3 — Final statistics for synteny analsyis. (PDF 85 kb) [file 12870_2018_1417_MOESM3_ESM.pdf]

Additional file 3 Statistics for synteny analysis

Subfamiliy distributions of novel, conserved and multi-copy genes in B. napus

BnAP2/EREBP

Subfamilies	Novel	Conserved	Multi-copy	Row sum
AP2	1	1	30	32
DREB_A-1	1	3	13	17
DREB_A-2	2	3	25	30
DREB_A-3	0	1	2	3
DREB_A-4	4	16	46	66
DREB_A-5	3	7	32	42
DREB_A-6	2	2	32	36
ERF	1	1	26	28
ERF_B-1	2	5	54	61
ERF_B-2	1	1	16	18
ERF_B-3	11	15	50	76
ERF_B-4	2	5	14	21
ERF_B-5	1	1	23	25
ERF_B-6	2	3	38	43
RAV	0	2	18	20
Total	33	66	419	518
Ratio	0.063706564	0.127413127	0.808880309	1

BnbZIP

Subfamilies	Novel	Conserved	Multi-copy	Row sum
I	1	1	10	12
II	2	0	37	39
III	5	4	54	63
IV	0	0	2	2
IX	1	4	6	11
V	4	5	25	34
VI	1	1	2	4
VII	6	1	34	41
VIII	0	2	2	4
X	0	3	17	20
XI	0	0	2	2
XII	2	5	13	20
Total	22	26	204	252
Ratio	0.0873016	0.1031746	0.80952381	1

BnMYB

Subfamilies	Novel	Conserved	Multi-copy	Row sum
1R-MYB	30	18	232	280
3R-MYB	1	0	15	16
4R-MYB	0	0	1	1
R2R3-MYB	34	36	354	424
Total	65	54	602	721
Ratio	0.090152566	0.074895978	0.834951456	1

BnNAC

Subfamilies	Novel	Conserved	Multi-copy	Row sum
I	0	0	3	3
II	6	4	25	35
III	0	4	20	24
IV	5	12	29	46
IX	1	0	43	44
V	2	3	16	21
VI	7	4	70	81
VII	2	2	36	40
VIII	2	0	43	45
X	0	0	12	12
XI	0	1	2	3
XII	1	0	5	6
XIII	2	0	11	13
XIV	0	0	4	4
XV	1	0	7	8
XVI	0	0	5	5
XVII	0	0	8	8
Total	29	30	339	398
Ratio	0.0728643	0.0753769	0.85175879	1

BnWRKY

Subfamilies	Novel	Conserved	Multi-copy	Row sum
GroupI	0	0	49	49
GroupII-b	2	3	38	43
GroupII-c	10	13	66	89
GroupII-d	2	2	23	27
GroupII-e	2	2	20	24
GroupIII	3	4	39	46
Total	19	24	235	278
Ratio	0.068345324	0.086330935	0.845323741	1

Chromosome distributions for novel, conserved and multi-copy genes in the three Brassica species

	Novel					
Chromosome	AP2/EREBP	bZIP	MYB	NAC	WRKY	Row sum
A01	2	0	0	0	1	3
A02	1	2	1	0	0	4
A03	2	0	1	0	1	4
A04	0	1	0	0	0	1
A05	1	1	0	0	0	2
A06	1	1	0	0	0	2
A07	1	0	1	0	0	2
A08	3	1	1	0	0	5
A09	1	1	2	0	0	4
A10	0	0	2	1	0	3
ChrA01	2	0	4	1	0	7
ChrA02	0	1	0	0	1	2
ChrA03	1	0	5	2	0	8
ChrA04	1	2	1	1	1	6
ChrA05	1	0	2	0	0	3
ChrA06	0	0	7	1	2	10
ChrA07	3	0	3	0	0	6
ChrA08	4	1	1	0	0	6
ChrA09	2	2	1	3	2	10
ChrA10	1	1	2	1	0	5
ChrAnn	2	3	3	2	2	12
C1	1	1	0	1	0	3
C2	1	0	2	4	1	8
C3	2	1	2	2	1	8
C4	2	1	5	0	0	8
C5	2	0	1	5	0	8
C6	1	1	1	1	1	5
C7	1	0	1	1	1	4
C8	0	0	1	0	0	1
C9	2	0	0	1	0	3

ChrC01	2	1	0	2	0	5
ChrC02	1	0	5	2	1	9
ChrC03	3	2	2	4	2	13
ChrC04	1	1	5	2	4	13
ChrC05	1	1	4	2	0	8
ChrC06	2	2	2	1	1	8
ChrC07	0	0	1	0	0	1
ChrC08	1	2	3	1	1	8
ChrC09	1	0	3	0	0	4
ChrCnn	4	2	11	3	1	21
ChrUnn	0	1	0	0	0	1
	Conserved					
Chromosome	AP2/EREBP	bZIP	MYB	NAC	WRKY	Row sum
A01	5	2	6	0	2	15
A02	3	2	7	1	4	17
A03	6	2	7	3	0	18
A04	5	3	7	0	2	17
A05	4	0	5	0	1	10
A06	8	1	7	3	2	21
A07	6	4	3	3	0	16
A08	8	1	2	0	4	15
A09	9	4	4	4	3	24
A10	4	0	1	7	1	13
ChrA01	3	2	1	0	1	7
ChrA02	2	0	3	1	3	9
ChrA03	3	2	3	2	0	10
ChrA04	1	3	4	0	1	9
ChrA05	1	0	2	0	0	3
ChrA06	4	0	3	1	1	9
ChrA07	1	2	2	3	0	8
ChrA08	6	1	2	0	2	11
ChrA09	1	2	1	2	2	8
ChrA10	2	0	0	5	0	7
ChrAnn	2	1	5	0	1	9
C1	6	2	9	1	2	20
C2	2	3	8	2	1	16
C3	8	4	10	3	7	32
C4	7	1	5	2	3	18
C5	7	0	6	4	1	18
C6	7	4	3	3	0	17
C7	5	2	3	0	1	11
C8	11	4	5	1	2	23
C9	5	1	3	3	2	14
ChrC01	3	1	8	1	0	13
ChrC02	3	2	2	0	0	7
ChrC03	5	3	6	1	3	18
ChrC04	4	1	2	1	1	9
ChrC05	4	0	1	4	0	9
ChrC06	5	2	0	1	0	8
ChrC07	4	0	2	0	1	7
ChrC08	4	1	2	2	0	9
ChrC09	3	1	0	2	3	9
ChrCnn	9	2	5	4	5	25
ChrUnn	0	0	0	0	0	0
	Multi-copy					
Chromosome	AP2/EREBP	bZIP	MYB	NAC	WRKY	Row sum
A01	16	7	26	19	9	77
A02	29	9	35	24	15	112
A03	32	11	52	26	25	146
A04	10	9	9	5	13	46
A05	16	10	29	19	10	84
A06	14	15	25	13	9	76
A07	26	4	30	18	8	86
A08	20	7	19	8	7	61
A09	30	20	37	23	17	127
A10	17	7	24	20	3	71
ChrA01	15	5	24	19	7	70
ChrA02	26	8	27	15	9	85
ChrA03	29	11	52	23	25	140
ChrA04	9	7	9	5	14	44
ChrA05	14	10	30	20	9	83
ChrA06	13	14	26	13	7	73
ChrA07	25	5	28	17	9	84
ChrA08	16	7	22	9	7	61
ChrA09	26	15	31	21	16	109
ChrA10	16	9	26	16	4	71

ChrAnn	25	9	27	12	8	81			
C1	16	6	29	21	8	80			
C2	29	9	32	22	15	107			
C3	31	15	54	27	21	148			
C4	17	17	19	9	22	84			
C5	18	11	29	23	5	86			
C6	22	6	25	14	9	76			
C7	28	10	35	17	17	107			
C8	21	11	31	10	7	80			
C9	25	15	42	27	12	121			
ChrC01	10	6	17	14	9	56			
ChrC02	24	8	25	17	12	86			
ChrC03	26	12	47	24	19	128			
ChrC04	18	15	17	11	15	76			
ChrC05	18	9	28	23	7	85			
ChrC06	18	6	22	13	9	68			
ChrC07	24	8	31	16	17	96			
ChrC08	20	11	28	9	8	76			
ChrC09	22	14	36	16	8	96			
ChrCnn	24	13	48	26	16	127			
ChrUnn	1	2	1	0	0	4			
Novel genes in B.napus									
BnAP2/EREBP	BnbZIP	BnMYB	BnNAC	BnWRKY					
BnaA01g00710D	BnaA02g27460D	BnaA01g16900D	BnaA01g31680D	BnaA02g19560D					
BnaA01g23970D	BnaA04g20520D	BnaA01g21010D	BnaA03g33890D	BnaA04g23470D					
BnaA03g46240D	BnaA04g27730D	BnaA01g21090D	BnaA03g33910D	BnaA06g16820D					
BnaA04g19700D	BnaA08g04710D	BnaA01g23930D	BnaA04g10830D	BnaA06g28100D					
BnaA05g11530D	BnaA09g36010D	BnaA03g01420D	BnaA06g23510D	BnaA09g11350D					
BnaA07g06760D	BnaA09g36130D	BnaA03g12400D	BnaA09g19790D	BnaA09g21040D					
BnaA07g08610D	BnaA10g28780D	BnaA03g26820D	BnaA09g19800D	BnaAnng30430D					
BnaA07g23500D	BnaAnng33510D	BnaA03g32470D	BnaA09g51470D	BnaAnng39820D					
BnaA08g08310D	BnaAnng36310D	BnaA03g34140D	BnaA10g10080D	BnaC02g32270D					
BnaA08g11220D	BnaAnng39310D	BnaA04g09530D	BnaAnng34420D	BnaC03g49470D					
BnaA08g22160D	BnaC01g02720D	BnaA05g00710D	BnaAnng41940D	BnaC03g67520D					
BnaA08g30930D	BnaC03g25660D	BnaA05g29580D	BnaC01g02440D	BnaC04g05170D					
BnaA09g28560D	BnaC03g59330D	BnaA06g03350D	BnaC01g40260D	BnaC04g14500D					
BnaA09g44290D	BnaC04g44430D	BnaA06g05130D	BnaC02g10100D	BnaC04g36310D					
BnaA10g04090D	BnaC05g35930D	BnaA06g11070D	BnaC02g10310D	BnaC04g40940D					
BnaAnng07060D	BnaC06g02640D	BnaA06g12690D	BnaC03g03740D	BnaC06g13990D					
BnaAnng21280D	BnaC06g15330D	BnaA06g21900D	BnaC03g31420D	BnaC08g29410D					
BnaC01g03990D	BnaC08g29000D	BnaA06g25300D	BnaC03g53950D	BnaC08g35490D					
BnaC01g05620D	BnaC08g40240D	BnaA06g29370D	BnaC03g54890D	BnaCnng77260D					
BnaC02g24770D	BnaCnng20400D	BnaA07g02020D	BnaC04g01450D						
BnaC03g25680D	BnaCnng21210D	BnaA07g36940D	BnaC04g11030D						
BnaC03g58550D	BnaUnng03790D	BnaA07g36950D	BnaC05g00990D						
BnaC03g66900D		BnaA08g22480D	BnaC05g01030D						
BnaC04g47060D		BnaA09g44790D	BnaC06g13570D						
BnaC05g12270D		BnaA10g25530D	BnaC08g07270D						
BnaC06g33570D		BnaA10g29180D	BnaC08g43050D						
BnaC06g37920D		BnaAnng06040D	BnaCnng38230D						
BnaC08g08930D		BnaAnng25220D	BnaCnng69540D						
BnaC09g43740D		BnaAnng41910D	BnaCnng72050D						
BnaCnng05070D		BnaC02g05740D							
BnaCnng07330D		BnaC02g16640D							
BnaCnng47540D		BnaC02g38100D							
BnaCnng73930D		BnaC02g38120D							
		BnaC02g39010D							
		BnaC03g15570D							
		BnaC03g65090D							
		BnaC04g27380D							
		BnaC04g32680D							
		BnaC04g41990D							
		BnaC04g52010D							
		BnaC04g54130D							
		BnaC05g06410D							
		BnaC05g35410D							
		BnaC05g39780D							
		BnaC05g50190D							
		BnaC06g02940D							
		BnaC06g23020D							
		BnaC07g27330D							
		BnaC08g17650D							
		BnaC08g18080D							
		BnaC08g26530D							
		BnaC09g01550D							
		BnaC09g05300D							
		BnaC09g50770D							

		BnaCnng17820D		
		BnaCnng17910D		
		BnaCnng23360D		
		BnaCnng23500D		
		BnaCnng52970D		
		BnaCnng61660D		
		BnaCnng62330D		
		BnaCnng67570D		
		BnaCnng69610D		
		BnaCnng75540D		
		BnaCnng78680D		
Conserved genes in B.napus				
BnAP2/EREBP	BnbZIP	BnMYB	BnNAC	BnWRKY
BnaA01g04150D	BnaCnng21630D	BnaCnng16520D	BnaC05g00910D	BnaAnng00020D
BnaA01g02720D	BnaCnng60880D	BnaCnng08670D	BnaA07g05000D	BnaA09g55250D
BnaA01g09230D	BnaC09g02690D	BnaCnng28540D	BnaCnng40940D	BnaCnng05780D
BnaA02g15410D	BnaC08g21370D	BnaCnng41840D	BnaC05g00930D	BnaC03g69190D
BnaA02g11090D	BnaC06g16270D	BnaCnng63050D	BnaC05g01020D	BnaA08g18040D
BnaA03g02000D	BnaC06g00420D	BnaC08g18260D	BnaC09g18980D	BnaCnng75330D
BnaA03g31510D	BnaC04g24820D	BnaC08g06060D	BnaA09g35990D	BnaC03g60600D
BnaA03g52210D	BnaC03g76870D	BnaC07g03440D	BnaC08g49770D	BnaC09g03790D
BnaA04g29310D	BnaC03g07230D	BnaC07g18400D	BnaA09g00430D	BnaC04g38910D
BnaA05g24390D	BnaC03g07400D	BnaC05g14250D	BnaC04g33200D	BnaCnng39880D
BnaA06g10760D	BnaC02g38190D	BnaC04g51450D	BnaC03g39160D	BnaCnng74680D
BnaA06g27540D	BnaC02g35400D	BnaC04g31750D	BnaA03g19110D	BnaA09g55920D
BnaA06g02510D	BnaC01g03810D	BnaC03g74080D	BnaC06g15990D	BnaC07g49530D
BnaA06g24070D	BnaAnng05020D	BnaC03g01730D	BnaA10g21230D	BnaC03g69160D
BnaA07g33380D	BnaA09g37270D	BnaC03g31750D	BnaA03g04920D	BnaC09g36400D
BnaA08g18390D	BnaA09g03330D	BnaC03g37840D	BnaCnng43170D	BnaCnng39890D
BnaA08g24660D	BnaA08g04620D	BnaC03g48330D	BnaCnng48900D	BnaC09g11790D
BnaA08g12010D	BnaA07g01890D	BnaC03g01040D	BnaA06g19740D	BnaA06g13050D
BnaA08g08300D	BnaA07g16990D	BnaC02g01090D	BnaC01g24910D	BnaA08g12420D
BnaA08g12710D	BnaA04g02780D	BnaC02g37370D	BnaA10g00910D	BnaA04g14500D
BnaA08g30910D	BnaA04g23690D	BnaC01g01250D	BnaC08g18550D	BnaA02g24470D
BnaA09g27310D	BnaA04g12040D	BnaC01g20650D	BnaA10g20110D	BnaA02g19570D
BnaA10g19960D	BnaA03g39740D	BnaC01g31020D	BnaA07g16830D	BnaA02g13630D
BnaA10g27750D	BnaA03g05670D	BnaC01g26440D	BnaA10g13930D	BnaA01g05500D
BnaAnng34830D	BnaA01g02570D	BnaC01g21680D	BnaCnng30500D	
BnaAnng28960D	BnaA01g27940D	BnaC01g33300D	BnaA07g17790D	
BnaC01g10100D		BnaC01g01430D	BnaC05g00890D	
BnaC01g01700D		BnaC01g17440D	BnaC09g43890D	
BnaC01g40920D		BnaAnng32620D	BnaA10g00880D	
BnaC03g09040D		BnaAnng35730D	BnaA02g05600D	
BnaC03g15720D		BnaAnng07760D		
BnaC03g66140D		BnaAnng39410D		
BnaC04g13510D		BnaAnng03960D		
BnaC04g42060D		BnaA09g02150D		
BnaC04g44080D		BnaA08g22850D		
BnaC04g08850D		BnaA08g10420D		
BnaC04g55700D		BnaA07g25720D		
BnaC05g09160D		BnaA07g25800D		
BnaC05g04210D		BnaA06g31780D		
BnaC05g20650D		BnaA06g12860D		
BnaC05g17200D		BnaA06g12870D		
BnaC06g24360D		BnaA05g37010D		
BnaC06g32560D		BnaA05g00510D		
BnaC06g03910D		BnaA04g28770D		
BnaC06g15540D		BnaA04g10190D		
BnaC06g21120D		BnaA04g24320D		
BnaC07g08360D		BnaA04g00800D		
BnaC07g10670D		BnaA03g04050D		
BnaC07g38490D		BnaA03g12690D		
BnaC07g43950D		BnaA03g29470D		
BnaC08g36830D		BnaA02g15320D		
BnaC08g07150D		BnaA02g17000D		
BnaC08g43940D		BnaA02g02300D		
BnaC08g18660D		BnaA01g32200D		
BnaC09g20090D				
BnaC09g06610D				
BnaC09g51170D				
BnaCnng05080D				
BnaCnng08620D				
BnaCnng07950D				
BnaCnng27900D				
BnaCnng24520D				
BnaCnng31550D				
BnaCnng09130D				

BnaCnng49280D									
BnaCnng57070D									
Lost genes from B. rapa									
BrAP2/EREBP	BrbZIP	BrMYB	BrNAC	BrWRKY					
Bra023921	Bra029353	Bra039065	Bra033306	Bra019123					
Bra011744	Bra034916	Bra037837		Bra013779					
Bra006325	Bra022225	Bra009245							
Bra034624	Bra024310	Bra015766							
Bra030458	Bra017316	Bra016546							
Bra019086	Bra026523	Bra009591							
Bra021047	Bra037374	Bra020373							
Bra025719		Bra005997							
Bra026770									
Bra020162									
Bra016518									
Bra003701									
Lost genes from B. olercea									
BoAP2/EREBP	BobZIP	BoMYB	BoNAC	BoWRKY					
Bo4g186300.1	Bo4g183320.1	Bo4g101470.1	Bo1g037810.1	Bo6g066790.1					
Bo14558s010.1	Bo1g119430.1	Bo8g104190.1	Bo5g085430.1	Bo7g088570.1					
Bo9g001020.1	Bo01108s040.1	Bo00877s020.1	Bo13819s010.1	Bo2g137260.1					
Bo5g054550.1	Bo6g066380.1	Bo2g149310.1	Bo3g064460.1	Bo3g184540.1					
Bo2g048150.1	Bo3g039150.1	Bo4g176030.1	Bo3g093950.1						
Bo5g020790.1		Bo24301s010.1	Bo5g002880.1						
Bo7g111110.1		Bo4g101460.1	Bo5g002950.1						
Bo1g007330.1		Bo5g021890.1	Bo5g002890.1						
Bo3g100470.1		Bo6g099880.1	Bo6g066300.1						
Bo6g073020.1		Bo4g140330.1	Bo7g030700.1						
Bo11579s010.1		Bo7g085920.1	Bo2g027150.1						
Bo3g185040.1		Bo2g167960.1	Bo5g086900.1						
Bo9g166320.1		Bo3g002620.1	Bo2g130240.1						
		Bo03161s010.1	Bo2g027170.1						
		Bo4g030640.1	Bo2g070830.1						
		Bo3g017760.1	Bo9g122170.1						
