# Supplementary material for: Exploring transcription factors reveals crucial members and regulatory networks involved in different abiotic stresses in Brassica napus L
Source: BMC Plant Biol. 2018 Sep 19;18:202. doi: 10.1186/s12870-018-1417-z (PMC6146658; doi:10.1186/s12870-018-1417-z)
Supplement: Supplementary file 4 — Responsive genes of the five TF families. (PDF 531 kb) [file 12870_2018_1417_MOESM4_ESM.pdf]

Additional file 4 Responsive TFs

BnAP2/EREBP										
GeneID	log2(Cold/Control)	Prob.	log2(Heat/Control)	Prob.	log2(Drought/Contro	Prob.	log2(Salt/Contro	Prob.	log2(ABA/Control)	Prob.
BnaA01g00290D	-0.132938832	0.265694899	-2.914530658	0.5804383	0.069421401	0.237816584	-1.458585215	0.5190897	-1.180120769	0.518545916
BnaA01g00710D	1.770531022	0.730952129	1.752480589	0.7003747	0.649428116	0.480742981	0.838678966	0.532627074	0.338992828	0.385926327
BnaA01g02720D	0	0.22451918	7.21916852	0.6206849	0	0.21244364	0	0.226982722	0	0.223935767
BnaA01g04150D	0.060372618	0.230703353	1.975125331	0.4506543	0.289506617	0.244749287	-1.8259706	0.328608792	1.664910657	0.459557399
BnaA01g05760D	-0.994946772	0.412404452	0.386278506	0.3115687	-0.547487795	0.337870742	-0.373458396	0.31152471	0.545621609	0.387559882
BnaA01g06090D	-1.034885518	0.35787629	-1.83720485	0.3922602	-0.760128627	0.321982106	-0.77690434	0.333430202	-1.161333853	0.389596323
BnaA01g13420D	-0.790126735	0.443818468	-2.810945206	0.6258322	-0.434693596	0.357942337	-1.879657956	0.604479288	-0.958760503	0.515082867
BnaA01g16910D	0	0.22451918	5.984893108	0.4734955	0	0.21244364	0	0.226982722	0	0.223935767
BnaA01g18170D	-2.736965594	0.4233594	-1.884522783	0.373263	-1.024247546	0.336076473	-0.658963082	0.30909199	-0.138706271	0.250004403
BnaA01g23940D	-0.061400545	0.239967505	0.043272708	0.2345847	0.329307625	0.307458435	0.198413558	0.283760876	-0.859137464	0.424570256
BnaA01g27570D	-0.162402043	0.29647927	1.658222345	0.7000907	0.257538418	0.336367079	0.397792553	0.394519004	0.4533348	0.449205678
BnaA01g28970D	2.433594297	0.736658565	1.944444852	0.6427181	0.265481726	0.288637324	1.444434886	0.588764574	2.155977884	0.738813889
BnaA01g29490D	-0.715779618	0.546538712	0.286325084	0.3645646	-0.360021028	0.411794621	-0.04741338	0.25313942	0.532041143	0.528778752
BnaA01g31290D	2.217230716	0.314604688	6.303780748	0.7869113	-2.736965594	0.243100321	2.666756592	0.356026982	0.887525271	0.252635264
BnaA01g34730D	0.462062834	0.300741484	-0.114511881	0.2424068	0.63991091	0.326567949	-0.382573104	0.281218077	1.234754922	0.477412026
BnaA01g34910D	0	0.22451918	0	0.2263112	4.984893108	0.377943482	2.321928095	0.249922946	3.415037499	0.281841118
BnaA02g00560D	-2.032421478	0.271339691	-3.906890596	0.3001427	0.03170886	0.21244364	-3.906890596	0.310144334	-1.321928095	0.264957378
BnaA02g01080D	-2.218834602	0.514420198	-2.94753258	0.5157764	-0.736965594	0.367206488	-4.321928095	0.599182342	-0.833641614	0.40593848
BnaA02g01570D	-0.983880335	0.28003364	0.265733555	0.2462309	-0.431339312	0.243657314	-4.89077093	0.384616401	-4.89077093	0.389404787
BnaA02g01920D	0	0.22451918	0	0.2263112	0	0.21244364	0	0.226982722	3.36923381	0.277510656
BnaA02g03590D	-1.874469118	0.241154144	-0.137503524	0.2263112	-1.874469118	0.229217302	-1.874469118	0.244405844	-1.874469118	0.242076597
BnaA02g04480D	7.372139541	0.664275776	5.392317423	0.4136968	0	0.21244364	0	0.226982722	0	0.223935767
BnaA02g06490D	-0.119716421	0.279518475	-1.52498684	0.6229658	0.331485848	0.371675649	0.257961745	0.342643718	0.102160107	0.281592342
BnaA02g08130D	0.341556902	0.287157878	-0.434937057	0.281064	-0.265012055	0.258319684	-0.867896464	0.341881979	-0.319459839	0.281002325
BnaA02g10340D	2.352381891	0.473671581	1.970853654	0.3987592	1.27897595	0.331679964	-1.906890596	0.298354997	-1.836501268	0.300999067
BnaA02g11090D	-1.528006616	0.443842686	-1.909313258	0.4430017	-2.234275413	0.489731938	-1.792903766	0.468332805	-2.826851098	0.544130209
BnaA02g13950D	1.006963948	0.423850347	-0.210035215	0.2579608	-0.345387068	0.272141058	-1.037719666	0.366209183	-0.548203951	0.318670964
BnaA02g14040D	1.032843257	0.36276815	-1.415037499	0.3175746	0.186700714	0.238666385	-1.756074417	0.349296823	-3.247927513	0.396731569
BnaA02g15410D	0	0.22451918	4.824428435	0.3620637	0	0.21244364	0	0.226982722	3.906890596	0.310091497
BnaA02g16050D	3.222392421	0.268539311	0	0.2263112	3.502500341	0.268900366	0	0.226982722	0	0.223935767
BnaA02g17250D	-5.345774837	0.423117229	0.167294745	0.2430321	-1.976541027	0.322362975	-5.345774837	0.428548469	0.239187664	0.252650675
BnaA02g18720D	0.006756275	0.229069798	1.737572622	0.7326936	-0.427703087	0.42764363	-0.473334535	0.437645303	-0.180883641	0.333676776
BnaA02g22560D	1.619896291	0.367842738	-1.133266531	0.2670973	1.956931278	0.404051745	-1.133266531	0.276401071	-4.502500341	0.354653211
BnaA02g25110D	-0.205951004	0.33156108	0.64040351	0.4947647	0.095413448	0.272477896	-0.276208041	0.363600338	-0.5988887	0.53781174
BnaA02g27630D	-0.637429921	0.268506288	-0.155278225	0.2384837	0.179706022	0.230890486	-1.155278225	0.295633872	-0.188445089	0.243967734
BnaA02g27680D	0.318132889	0.373690074	0.889395885	0.5555805	-1.514031815	0.683735338	0.026628823	0.23922558	-0.630288324	0.525705379
BnaA02g32120D	0.516790998	0.259339005	5.182814513	0.7984475	-0.04508789	0.217157174	-0.022367813	0.226982722	-0.977973694	0.270696837
BnaA02g34370D	7.352516415	0.917450421	6.375660079	0.8334346	1.280107919	0.265435116	0.050626073	0.226982722	-0.900464326	0.245312885
BnaA02g34570D	0	0.22451918	4.247927513	0.3211411	0	0.21244364	0	0.226982722	0	0.223935767
BnaA02g35180D	0	0.22451918	0	0.2263112	4.196397213	0.310820212	0	0.226982722	0	0.223935767
BnaA02g35550D	-0.390462245	0.418359664	-0.829961202	0.5551974	0.24748542	0.357482211	0.430141991	0.438856159	-0.134853269	0.311670013
BnaA03g02000D	0	0.22451918	0	0.2263112	0	0.21244364	0	0.226982722	0	0.223935767
BnaA03g02990D	-1.313660479	0.371178097	0.398358758	0.2838027	-1.145262947	0.355179383	-0.89862298	0.336596041	-1.145262947	0.377267604
BnaA03g04290D	2.342215494	0.74966096	3.094089163	0.7935072	0.535743873	0.385239441	1.264527106	0.582195128	0.63024338	0.442645039
BnaA03g04570D	4.273018494	0.327631301	0	0.2263112	0	0.21244364	0	0.226982722	0	0.223935767
BnaA03g07750D	0.841302254	0.251219663	3.115477217	0.3937308	-3	0.249962574	-3	0.26634656	-3	0.264244073
BnaA03g12320D	-0.981654857	0.406957801	-1.791616275	0.4616775	0.106726801	0.244302371	-3.571835067	0.573065272	-4.141200712	0.620579274
BnaA03g13620D	6.785724906	0.589973229	5.89077093	0.463179	0	0.21244364	0	0.226982722	0	0.223935767
BnaA03g16280D	-0.047828525	0.227618972	0.798366139	0.2871799	-0.665580961	0.254339269	-4.938599455	0.388896227	0.296617006	0.253397002
BnaA03g19580D	-2.151819064	0.577380323	0.559900579	0.3768141	-0.073041951	0.237816584	-1.1966733	0.491222392	-0.499742368	0.379112508
BnaA03g20950D	-5.203442128	0.761514143	-1.675063155	0.5699919	-0.266049444	0.315097837	0.159513236	0.289764521	-0.955514614	0.533067385
BnaA03g22100D	3.571247563	0.770007749	1.232258574	0.4129152	0.688273218	0.330601201	-0.339357901	0.274380042	-0.077652556	0.239947691
BnaA03g24540D	0.140660407	0.254634277	-1.606855107	0.3930396	-0.82045058	0.337745253	-2.127111918	0.448151132	-2.053111336	0.466426257
BnaA03g31510D	0.783460305	0.368545035	2.457144893	0.6408556	0.290420294	0.267594843	0.362570079	0.292490049	0.362570079	0.297472172
BnaA03g33290D	0.605859842	0.45374749	1.149815534	0.5861623	-0.423156232	0.375561397	-0.322535418	0.344530452	-0.088475188	0.268349977
BnaA03g33790D	1.346910745	0.692462309	1.364787905	0.6660811	0.043390066	0.235069041	-0.27118428	0.349935274	-0.004103425	0.225373384
BnaA03g34290D	1.439764585	0.377095882	-0.145197916	0.2384022	-1.062735755	0.274426274	0.53287399	0.275097749	2.990375581	0.629211578
BnaA03g35680D	0.560279349	0.450392317	0.272891903	0.3290403	0.219242431	0.316271267	0.226581738	0.325152348	-0.314604754	0.372719187
BnaA03g40370D	0.293557238	0.316394554	0.916662684	0.4702658	1.092024496	0.556670277	-0.0402171	0.237039434	-0.129592907	0.2722005
BnaA03g40380D	-1.689659879	0.546540914	0.935250191	0.4787242	-0.658963082	0.395619782	-1.900891855	0.568560886	-1.072314205	0.508033481
BnaA03g42840D	0	0.22451918	0	0.2263112	0	0.21244364	0	0.226982722	5.101538026	0.409011853
BnaA03g46240D	1.658076011	0.669984413	-1.740517158	0.5265134	-0.480873341	0.356410053	-2.366702321	0.607094737	-1.675945156	0.590565448
BnaA03g48270D	0	0.22451918	3.938599455	0.3001427	0	0.21244364	0	0.226982722	0	0.223935767
BnaA03g48910D	-0.59946207	0.250882824	-1.556393349	0.2647857	-4.058893689	0.30296946	-4.058893689	0.319509757	-0.029146346	0.223935767
BnaA03g49050D	0	0.22451918	0	0.2263112	0	0.21244364	0	0.226982722	0	0.223935767
BnaA03g52210D	-1.622930351	0.377932474	-2.237039197	0.3832162	0.338075517	0.268275124	-0.622930351	0.303795044	1.222392421	0.466738878
BnaA03g52830D	0	0.22451918	6.81164228	0.5696705	0	0.21244364	0	0.226982722	2.874469118	0.258500211
BnaA03g53830D	1.153453992	0.533679418	0.132215195	0.2590572	0.471964567	0.350078375	-0.049988136	0.238904153	0.674438263	0.436597362
BnaA03g53850D	1.951619538	0.652101166	2.816098555	0.7366057	0.332936419	0.295865036	0.560194596	0.358323206	0.664579669	0.406077178
BnaA03g54460D	-2.174115231	0.663027493	-2.054576533	0.6236989	-0.210447444	0.300417856	-1.323183886	0.580083835	-1.539655765	0.645199285
BnaA03g55280D	-1.725825037	0.267037849	5.163386392	0.747717	-0.381870635	0.228512804	-0.338801913	0.243564849	-0.966833136	0.256159956
BnaA04g02310D	-3.058893689	0.262619324	-3.058893689	0.2596164	-0.120294234	0.21934332	0.879705766	0.25774948	0.748461233	0.252362271
BnaA04g12700D	0.087462841	0.22952552	-0.093109404	0.2311811	1.247927513	0.294594737	1.273018494	0.310439343	-0.941106311	0.259724277
BnaA04g13590D	2.674309986	0.811828525	1.10086168	0.5730631	0.637074533	0.470725897	0.206671963	0.310611064	-0.003320358	0.224664483
BnaA04g15130D	0	0.22451918	0	0.2263112	0	0.21244364	0	0.226982722	0	0.223935767
BnaA04g19700D	0	0.22451918	3.544320516	0.2809099	0	0.21244364	0	0.226982722	0	0.223935767
BnaA04g25900D	-0.450165723	0.258559653	1.36008909	0.3518198	0.748815312	0.284707545	-1.956056652	0.317475519	1.405711707	0.392559618
BnaA04g25970D	-2.605721061	0.359490031	-5.544320516	0.4285463	-0.669851398	0.271134947	-1.669851398	0.338167952	-2.544320516	0.371715277
BnaA04g27050D	2.619896291	0.402992797	1.706953025	0.3073704	-0.180572246	0.219924531	-3.502500341	0.285733471	-3.502500341	0.284370707
BnaA04g29310D	1.669531829	0.699969619	4.061209901	0.8661167	-1.695871814	0.611385572	0.418845089	0.378075575	-1.244246681	0.586342862
BnaA05g01500D	0.992979572	0.326435855	-0.401098308	0.2514442	0.772931091	0.289652242	-0.294183104	0.252538395	-0.382719779	0.256921695
BnaA05g02350D	-0.339024089	0.303304097	0.560416202	0.351527	0.143670361	0.254537409	-0.240550392	0.28597344	0.568927702	0.394948307
BnaA05g04450D	-0.500284786	0.325218394	-3.721283972	0.5288206	0.162019803	0.255730653	0.310053995	0.301899503	0.810729649	0.448802793
BnaA05g09970D	-3.772589504	0.296939396	0.342887714	0.2419203	-3.772589504	0.285134647	-3.772589504	0.301791627	-3.772589504	0.301360122
BnaA05g11530D	1.59708606	0.379407517	1.430928872	0.3426657	0.638733757	0.265276604	0.39665897	0.258542041	-0.036994207	0.228581053
BnaA05g15470D	1.874469118	0.241154144	0	0.2263112	0	0.21244364	2.584962501	0.255299148	0	0.223935767
BnaA05g15710D	0	0.22451918	0	0.2263112	0	0.21244364	0	0.226982722	0	0.223935767
BnaA05g18740D	-0.010179324	0.229243721	-0.513993661	0.428412	0.11311218	0.275562278	0.04676338	0.250482141	-0.020137064	0.236812674
BnaA05g23130D	-0.04053816	0.231625806	2.451813191	0.6766067	-0.165389647	0.250235567	-0.333787179	0.290552679	0.158167691	0.264963983
BnaA05g23420D	-0.062735755	0.228180369	-0.062735755	0.2301309	-0.46712601	0.246746099	-1.854149134	0.314078516	-1.969626351	0.31906064
BnaA05g24390D	3.570545728	0.851476364	1.233557429	0.5779439	1.688863367	0.703214713	0.36893158	0.355483198	-0.305336846	0.342700958
BnaA05g25200D	1.248124604	0.685564831	0.207683988	0.3229024	0.087812675	0.265970094	0.177614706	0.319353447	0.297534924	0.402127585
BnaA05g27930D	2.823446199	0.697301332	5.453457718	0.8986888	0.802235951	0.355249833	0.939117758	0.389162616	-0.190942783	0.258114939
BnaA06g01090D	0.700439718	0.444705696	3.536849267	0.8307861	1.387768124	0.631633291	2.789142892	0.802434482	2.668914279	0.814067509
BnaA06g02670D	-0.986628709	0.639296911	-0.006703692	0.2323743	-0.468017365	0.471313713	0.017047958	0.237442319	-0.448579299	0.497322907
BnaA06g07470D	1.259063943	0.582666262	0.990623515	0.4871473	-0.194345367	0.275179207	-0.46987748	0.351141727	0.540674583	0.416094262
BnaA06g10760D	-0.275634443	0.252120099	-0.61667136	0.2646293	-0.597562538	0.259281764	-1.845490051	0.328670436	0.108706259	0.238954789
BnaA06g23710D	3.655917148	0.663663743	5.255873691	0.8261607	-1.639410285	0.284060287	-1.017921908	0.281693614	-1.017921908	0.283021153
BnaA06g24400D	0	0.22451918	0	0.2263112	3.273018494	0.259946634	0	0.226982722	0	0.223935767
BnaA06g27540D	-4.604862058	0.352933795	1.167727446	0.3113838	-1.060541542	0.264442214	-1.060541542	0.278996706	-4.604862058	0.361596921

BnaA06g27670D	0	0.22451918	0	0.2263112	0	0.21244364	0	0.226982722	0	0.223935767
BnaA06g27900D	1.027470375	0.603200183	-2.04631753	0.6673007	0.309684499	0.35871068	0.415998244	0.402087957	0.098180394	0.279474444
BnaA06g33270D	0.59776757	0.501072158	-0.529244948	0.4488446	-0.609555257	0.507690038	-0.700685369	0.527906936	-0.59248495	0.536065906
BnaA06g33420D	-0.026152288	0.230410546	-0.739848103	0.3658327	-0.176689972	0.265124696	-0.093685446	0.253183451	-0.70774726	0.404754042
BnaA06g35500D	-1.739716205	0.629667301	2.668897137	0.7916336	0.267835392	0.328498714	0.033410415	0.237215559	-0.637429921	0.472665469
BnaA06g40170D	0.013127434	0.226716334	2.109785303	0.661711	0.723185155	0.408945806	-0.43718554	0.321951284	-0.259346668	0.294077372
BnaA07g06750D	-4	0.311179066	2.222392421	0.3795506	-4	0.299295061	-4	0.316059918	-4	0.316335112
BnaA07g06760D	0	0.22451918	0	0.2263112	4.169925001	0.310820212	0	0.226982722	0	0.223935767
BnaA07g08440D	0.593717058	0.409309063	2.2178471	0.7191056	0.358949764	0.335970799	-0.888222768	0.443670964	-0.908441047	0.47645655
BnaA07g08450D	0.089916637	0.242316566	-0.466989579	0.2881552	-3.151487753	0.467936525	-0.06160225	0.237255187	-1.236376651	0.402805664
BnaA07g10270D	0	0.22451918	3.736965594	0.2890842	0	0.21244364	0	0.226982722	0	0.223935767
BnaA07g12040D	1.901372531	0.782561027	-0.758550319	0.5252739	0.522826754	0.481528937	0.303114394	0.377344658	0.132769229	0.309602751
BnaA07g12050D	0.069962558	0.236898535	-2.839063782	0.440558	-1.02713613	0.36228821	-0.913064363	0.353640495	-0.238159737	0.271590669
BnaA07g13990D	1.505046396	0.727183064	-2.764426956	0.7685371	0.164092297	0.303343725	0.261648471	0.353988341	-0.004035513	0.225463648
BnaA07g16350D	-2.146841388	0.288610906	3.076470826	0.5161044	0.615659298	0.253971609	-0.36923381	0.250158512	0.800691192	0.281863134
BnaA07g20720D	0.461296304	0.410194089	0.62736898	0.4501083	-0.147669239	0.282459756	0.403404139	0.388396474	0.338386122	0.390529783
BnaA07g21680D	1.184424571	0.340360703	2.788495895	0.5259321	2.012243596	0.446852214	1.821339151	0.424530628	2.237535908	0.507582162
BnaA07g21980D	-0.117356951	0.234252263	2.430452552	0.4364124	-1.04580369	0.257412642	0.882643049	0.289511342	0.475733431	0.257617387
BnaA07g23090D	1.883837721	0.760569675	0.950071419	0.5610007	-2.106768611	0.719149583	-1.278747271	0.622952552	-0.68456624	0.523825249
BnaA07g23650D	-3.187465583	0.643992832	1.993447248	0.6942874	-0.171015062	0.272794921	0.241566786	0.306864014	-0.184160438	0.29276084
BnaA07g24590D	-1.139135224	0.585466642	0.842490084	0.5234576	-0.634172046	0.466985452	-2.365524033	0.723273099	-0.673512937	0.512454208
BnaA07g27210D	-7.05166212	0.624205238	0.608928086	0.3402748	-0.252920328	0.265261193	-5.314696526	0.583260682	0.63016192	0.383007063
BnaA07g29400D	4.087462841	0.316995579	4.938599455	0.3727918	3.969626351	0.297018652	0	0.226982722	0	0.223935767
BnaA07g30130D	3.196955207	0.520481084	0.422691072	0.2446128	-1.031026896	0.246587587	1.186878135	0.301835658	0.888354644	0.276429691
BnaA07g31860D	-3.36923381	0.275018493	3.31259023	0.4472353	0.903784685	0.248875004	-3.36923381	0.279293917	0.928446739	0.263315016
BnaA07g33380D	-3.938599455	0.305333932	-3.938599455	0.3001427	-0.938599455	0.242503699	-0.938599455	0.257969636	0.952171475	0.280832805
BnaA07g33640D	-7.862120725	0.72375524	-0.854626189	0.3854244	-3.359620385	0.603876061	-4.803227036	0.653285604	-0.232764105	0.296285533
BnaA07g33930D	-0.320118505	0.394576244	-0.537748055	0.4731366	-0.379535046	0.429125277	-0.43791231	0.448703723	-0.267513843	0.400762619
BnaA07g35130D	-0.224335112	0.335385184	1.781221082	0.7387148	-0.844739063	0.575445155	-0.342082114	0.38761272	-0.158796255	0.323415319
BnaA07g38140D	1.452512205	0.476729543	2.140089772	0.5637615	1.342035668	0.459663074	1.438573014	0.477867748	1.275634443	0.47528312
BnaA08g01300D	2.917315897	0.768504086	3.068159894	0.7618356	0.79262015	0.414544366	-0.149322749	0.264779052	0.476343722	0.354193085
BnaA08g04090D	1.595490053	0.698049861	-0.092880967	0.2583967	-0.218827217	0.303235848	0.456625121	0.399212723	-0.24905238	0.33437467
BnaA08g04940D	1.099535674	0.506912889	-0.249359469	0.2769977	0.572183896	0.370224823	0.518130992	0.359969971	0.886218077	0.484972172
BnaA08g08300D	-0.041519269	0.232609902	1.110747066	0.481399	-0.02061028	0.217819842	0.415037499	0.333348744	0.180120769	0.279676988
BnaA08g08310D	-0.065095028	0.228660309	2.97593224	0.5150476	-1	0.257412642	-0.043068722	0.229441861	2.08246216	0.447145021
BnaA08g11220D	2.874469118	0.256930501	7.544320516	0.6611253	0	0.21244364	0	0.226982722	2.874469118	0.258500211
BnaA08g12010D	-2.027480736	0.32307628	-5.142957954	0.3895391	-5.142957954	0.392044454	-5.142957954	0.406962204	1.993178734	0.496598595
BnaA08g12710D	-4.029747343	0.311179066	-1.091147888	0.2577627	-0.970853654	0.245768608	-0.970853654	0.261221336	-0.091147888	0.231015974
BnaA08g13860D	3.573991383	0.458099968	7.502379789	0.9028651	3.2410081	0.414110659	3.102810806	0.407704128	0.8259706	0.252439325
BnaA08g13940D	1.896164189	0.288003276	1.736965594	0.2744087	3.115477217	0.374192029	-0.08246216	0.226982722	0	0.223935767
BnaA08g15790D	-2.098302074	0.364943288	-0.72549281	0.2844764	0.505038956	0.280465145	-0.739848103	0.297093505	-0.296241451	0.261078235
BnaA08g16220D	-1.904030941	0.593533147	-0.017921908	0.2312361	0.145116708	0.270685829	0.051240117	0.243324879	-0.629535537	0.434820705
BnaA08g18380D	1.43410805	0.625391877	3.094563002	0.7996517	0.396300937	0.34871341	-0.017165855	0.231436472	0.789067308	0.50528814
BnaA08g18390D	-0.712341807	0.347956075	-0.821820284	0.3445877	-0.768925336	0.352405421	-0.740356183	0.354593769	0.323282103	0.307374775
BnaA08g19170D	0	0.22451918	0	0.2263112	2.662965013	0.239348868	0	0.226982722	0	0.223935767
BnaA08g19490D	-0.11047352	0.275623921	0.309496275	0.3521544	-0.47102838	0.422522808	-1.757450452	0.69411788	-0.578873336	0.495931523
BnaA08g20700D	0.09231475	0.256049878	1.050582241	0.528825	-0.037340912	0.227555127	-0.105449299	0.265699303	-0.181250323	0.296254711
BnaA08g22160D	-5.183221824	0.405451936	-0.745816512	0.2701883	1.598137889	0.413113354	0.308631272	0.259931223	1.297904866	0.390584822
BnaA08g23880D	0	0.22451918	0	0.2263112	4.459431619	0.332505548	0	0.226982722	0	0.223935767
BnaA08g24660D	3.530514717	0.52572079	-3.584962501	0.2834218	1.399930607	0.289156892	0.852442812	0.267090687	0.784271309	0.26242999
BnaA08g30910D	8.217553864	0.76367387	0	0.2263112	0	0.21244364	3.222392421	0.272455881	3.169925001	0.270666015
BnaA08g30930D	5.044394119	0.393121015	0	0.2263112	0	0.21244364	0	0.226982722	0	0.223935767
BnaA08g30950D	0	0.22451918	0	0.2263112	0	0.21244364	0	0.226982722	0	0.223935767
BnaA09g04470D	-0.558490289	0.297428141	-6.339850003	0.5144973	0.830074999	0.360932051	-1.639410285	0.387799852	-6.339850003	0.556734122
BnaA09g04650D	-3.024756099	0.545647082	1.697709925	0.5915958	-2.024756099	0.504288633	-0.852367058	0.386249956	-3.002729793	0.576959826
BnaA09g05710D	0.312682298	0.3194283	0.370034005	0.3223123	0.146498312	0.263612227	0.349315195	0.33151925	0.156073756	0.282428934
BnaA09g07540D	0.873875538	0.536349907	1.862924815	0.7104512	-0.2410081	0.312083906	-0.367942382	0.35971459	-1.486406364	0.641606344
BnaA09g10730D	0	0.22451918	3.36923381	0.2716061	0	0.21244364	0	0.226982722	0	0.223935767
BnaA09g10750D	-5.491853096	0.437735567	-5.491853096	0.4231987	-1.617383978	0.320471838	-0.243925583	0.254530804	-2.491853096	0.365933989
BnaA09g12290D	-1.054447784	0.505891366	1.194552144	0.574941	-0.92665385	0.486929356	-1.079578499	0.514838494	-0.780882711	0.485016203
BnaA09g12950D	-3.906890596	0.305333932	-3.906890596	0.3001427	-3.906890596	0.293320036	-3.906890596	0.310144334	-3.906890596	0.310091497
BnaA09g18210D	2.464108106	0.447646976	0.893084796	0.2735897	-0.637429921	0.241825619	-0.559427409	0.253802089	-1.485426827	0.27716281
BnaA09g27300D	0.292026609	0.26306624	2.586503019	0.5804097	-5.700439718	0.453098031	-2	0.358871394	-0.478047297	0.277083553
BnaA09g27310D	0	0.22451918	3.736965594	0.2890842	0	0.21244364	0	0.226982722	0	0.223935767
BnaA09g27330D	5.073248982	0.396007256	0	0.2263112	4.459431619	0.332505548	3.544320516	0.28952235	5.736965594	0.479642203
BnaA09g28560D	1.94584423	0.44734096	-0.143590854	0.2381468	-0.160464672	0.227702631	1.343033728	0.369227518	-2.984893108	0.341468086
BnaA09g28670D	-0.078069263	0.267969108	0.391339685	0.4072308	-0.109266195	0.281099193	-0.544679341	0.482350118	0.241023503	0.378033745
BnaA09g30360D	-0.160040413	0.260508031	0.691534165	0.3567535	-2	0.462985223	-7.058893689	0.629555021	-7.058893689	0.650916728
BnaA09g30810D	0.207371184	0.326136444	2.819387478	0.8181206	-0.636088003	0.494198897	-0.318996663	0.369603984	0.178682425	0.329562066
BnaA09g34250D	0.253666223	0.288373137	1.268858778	0.5049799	0.148918313	0.254746557	-0.695545667	0.36279677	-0.450007909	0.332252369
BnaA09g36920D	0.534061602	0.283078393	-1.10780329	0.2930757	0.634128558	0.284698739	0.337996464	0.264948572	0.789103218	0.321510973
BnaA09g38530D	-1.042435266	0.306115485	-2.584962501	0.3420889	0.326103771	0.252227976	-2.584962501	0.361161013	-0.632791026	0.283254518
BnaA09g39350D	1.338138322	0.581968368	0.98463569	0.4692376	-0.510503509	0.344046109	-1.071000769	0.449890362	-0.016394438	0.230069305
BnaA09g44290D	-3.273018494	0.272268748	1.883485991	0.3078173	-3.273018494	0.259946634	-3.273018494	0.276222745	-3.273018494	0.274705872
BnaA09g46790D	3.415037499	0.278780954	0	0.2263112	0	0.21244364	0	0.226982722	0	0.223935767
BnaA09g47030D	0	0.22451918	7.303780748	0.6312128	0	0.21244364	0	0.226982722	0	0.223935767
BnaA09g50010D	-4.68182404	0.360670066	2.073063462	0.414245	-4.68182404	0.350397601	1.159478214	0.331922135	-4.68182404	0.370105939
BnaA09g54740D	0.162778383	0.300840554	-0.628697979	0.4468566	0.065405014	0.249863503	-0.061799112	0.254330463	0.550018227	0.499894325
BnaA10g00620D	-0.768908487	0.392442936	-1.235084835	0.4379051	0.461153783	0.350736641	0.747457968	0.433722128	1.623067106	0.676340308
BnaA10g02160D	0.176525413	0.262066734	-0.531023694	0.2947599	-0.699295176	0.319868611	-1.669851398	0.418234175	-1.640996536	0.435230195
BnaA10g04090D	0	0.22451918	0	0.2263112	0	0.21244364	0	0.226982722	0	0.223935767
BnaA10g05780D	-4.297680549	0.331045916	-1.634715536	0.2712979	2.042169454	0.398096534	-4.297680549	0.336195357	-4.297680549	0.338075487
BnaA10g07620D	0	0.22451918	8.658211483	0.7903964	3	0.249962574	3.115477217	0.269941703	0	0.223935767
BnaA10g07630D	-1.704544116	0.396865864	4.763966796	0.861478	0.184424571	0.247672954	-1.777607579	0.40383159	0.775784843	0.380871552
BnaA10g12950D	0.358219367	0.372399961	1.265313288	0.6233708	0.21398998	0.311033763	0.485612993	0.422445754	0.38341552	0.410057593
BnaA10g15390D	-0.120294234	0.230241026	4.295723025	0.5486368	0.782408565	0.238943781	-0.058893689	0.226982722	0.782408565	0.252382085
BnaA10g16490D	0	0.22451918	2.584962501	0.2496566	0	0.21244364	0	0.226982722	0	0.223935767
BnaA10g19960D	1.389706209	0.35513315	0.886043811	0.286515	0.944146765	0.292961182	-0.036994207	0.229441861	-0.036994207	0.228581053
BnaA10g20950D	-3.506677744	0.625590017	-1.901537361	0.5177468	-0.998213381	0.452428758	-1.045400066	0.459132499	-1.466600304	0.545479763
BnaA10g21750D	0.215728691	0.240000528	-0.071553261	0.2305603	-1.146841388	0.25810173	-1.253756592	0.276870002	-4.36923381	0.34393383
BnaA10g23760D	0	0.22451918	0	0.2263112	0	0.21244364	0	0.226982722	0	0.223935767
BnaA10g25000D	1.201407396	0.592877083	5.028684036	0.9056413	-0.303170531	0.315329001	0.62772482	0.433592236	0.052420917	0.246341012
BnaA10g27820D	-1.146841388	0.269891067	-4.36923381	0.3291019	-1.096215315	0.257907993	-4.36923381	0.341758692	-1.096215315	0.272823541
BnaA10g29740D	0	0.22451918	0	0.2263112	0	0.21244364	0	0.226982722	0	0.223935767
BnaA10g30200D	0.97093465	0.49711596	0.817243206	0.430411	-2.472388552	0.575475977	-0.955064769	0.433493166	-2.202030806	0.590712952
BnaAnng01320D	1.235832678	0.46539813	6.343285285	0.9421717	-0.353360603	0.270518511	1.408805546	0.501083166	0.485426827	0.325244813
BnaAnng03730D	1.483671965	0.732114551	2.246126938	0.787847	0.56435507	0.501805277	1.344014403	0.713480573	0.5997155	0.555195234
BnaAnng06420D	-2.330267396	0.459398887	-1.190864339	0.3690514	-0.438791853	0.289779932	-1.427564597	0.411019673	-2.438791853	0.489289425
BnaAnng06940D	-1.615381897	0.629689316	0.546959612	0.4200527	-1.038493518	0.552670048	-3.909042266	0.761575786	-0.403378813	0.401319613
BnaAnng07060D	-1.01713144	0.526568389	1.970506059	0.7244619	-0.345639683	0.350641974	0.1100398	0.273127356	-0.855803189	0.530156927
BnaAnng09170D	3.36923381	0.275018493	3.058893689	0.2596164	0	0.21244364	0	0.226982722	3.624490865	0.291074448
BnaAnng09570D	-4.169925001	0.322490665	0.199308808	0.2388381	0.245112498	0.228264028	-4.169925001	0.327428758	-4.169925001	0.3284789
BnaAnng13220D	0.472935883	0.273008472	3.542898441	0.67081	-0.835613182	0.272603385	-5.273018494	0.421329564	-2.273018494	0.346282891

BnaAnng13660D	0	0.22451918	0	0.2263112	2.938599455	0.248216739	4.459431619	0.348669379	3.841302254	0.303865494
BnaAnng20260D	0.22762907	0.284692134	-1.208470045	0.4130759	-0.893834443	0.392288827	-0.351743749	0.306520571	-0.664495956	0.37968051
BnaAnng20420D	-0.033166864	0.2266679	0.431339312	0.2654791	3.107890723	0.66356027	-0.044394119	0.229441861	0.723790205	0.31771769
BnaAnng21280D	0.228991532	0.295169344	2.081021009	0.6892833	-0.256751364	0.28915469	-1.166687665	0.474990313	-0.483946001	0.36640292
BnaAnng23490D	-0.648527629	0.25421378	4.527881406	0.7080735	-0.648527629	0.241852038	-4.273018494	0.332813766	-0.076621282	0.230703353
BnaAnng26790D	2.584962501	0.251618144	0	0.2263112	0	0.21244364	0	0.226982722	0	0.223935767
BnaAnng28960D	-8.509115185	0.793604037	0.432421553	0.3525749	-1.586283045	0.568230653	-5.450221496	0.740665399	-0.208381312	0.304710892
BnaAnng29660D	-0.078002512	0.22451918	1.337034987	0.261371	-0.078002512	0.21244364	-2.662965013	0.255299148	-2.662965013	0.252892846
BnaAnng29870D	-1.440572591	0.365843725	0.6387852	0.3128853	-2.10496956	0.392568424	-6.247927513	0.529472243	-0.091423028	0.242697436
BnaAnng34260D	7.375039431	0.664407869	5.653442239	0.4382155	3.169925001	0.256058685	0	0.226982722	0	0.223935767
BnaAnng35170D	3.407592687	0.811498292	1.973593269	0.6328794	1.047305715	0.490784283	1.939337218	0.664172303	1.326447112	0.586518986
BnaAnng36290D	0	0.22451918	8.215937399	0.7413875	0	0.21244364	3.415037499	0.283188471	3.321928095	0.277510656
BnaAnng36370D	0	0.22451918	3.36923381	0.2716061	0	0.21244364	0	0.226982722	0	0.223935767
BnaAnng37390D	-0.4622646	0.393149635	0.310134688	0.3386325	0.654262014	0.484410775	-0.704779986	0.464854345	-0.806293987	0.530174539
BnaAnng37500D	2.711042249	0.619159269	0.551642428	0.2821846	0.167727446	0.235956268	-0.040077439	0.229441861	-2.331843564	0.371279369
BnaAnng40110D	1	0.251897742	-2.584962501	0.2496566	-2.584962501	0.239348868	-2.584962501	0.255299148	-2.584962501	0.252892846
BnaAnng40580D	2.055560545	0.785902991	1.12659757	0.620139	-0.580168605	0.472561996	-0.046262723	0.249515657	0.483036617	0.479186481
BnaAnng40920D	-0.9274875	0.279580119	-2.014950341	0.3037796	-1.045323991	0.277490842	-3.20E-16	0.226982722	0.954676009	0.326259731
BnaAnng41450D	0.04508789	0.227618972	-4.392317423	0.3309579	-0.977279923	0.252584628	-0.977279923	0.268004333	-4.392317423	0.345725897
BnaC01g00660D	-1.131152692	0.533344781	-2.224966365	0.6190954	-0.376969458	0.35302626	-0.963811692	0.502690303	-1.57247987	0.633896492
BnaC01g01290D	0.236376651	0.267984519	-2.915111102	0.4172589	0.268488836	0.261776128	-2.113656782	0.419975607	-0.686842115	0.325365899
BnaC01g01700D	1.40330266	0.685239001	2.170528867	0.7612874	0.654298773	0.498058227	0.714173037	0.509015376	-0.008723806	0.228750572
BnaC01g01710D	0.241640306	0.274981067	-0.115477217	0.2470807	1.153851518	0.474096481	-0.667424661	0.33206964	0.210008697	0.273180193
BnaC01g03990D	3.772589504	0.296939396	6.357552005	0.5165051	0	0.21244364	0	0.226982722	0	0.223935767
BnaC01g05620D	0.210473731	0.257152859	1.422571172	0.4240287	-5.992466327	0.488369175	-0.329501315	0.268708831	1.653792353	0.525500634
BnaC01g07340D	1.742202243	0.437566047	0.644905041	0.2811014	0.898120386	0.310078287	0.447458977	0.27113935	-0.181446871	0.243782803
BnaC01g10080D	-1.271397879	0.51619025	-0.800422208	0.4095534	0.386614277	0.350994223	0.640405164	0.432740234	-0.357812631	0.350175244
BnaC01g10100D	0.17992797	0.272409648	1.951279185	0.6329454	-0.140792344	0.250301613	-0.886841043	0.392141322	-1.134768556	0.453837754
BnaC01g15610D	-0.082224131	0.253857128	1.170599316	0.575749	-0.30408113	0.325592659	0.050695844	0.244452077	-0.269523908	0.335739635
BnaC01g20660D	0	0.22451918	6.437405312	0.5262712	3.662965013	0.278871218	0	0.226982722	0	0.223935767
BnaC01g35070D	0.818027371	0.41903334	1.726731537	0.5863385	2.303818714	0.710363169	0.454241571	0.33315941	0.375509135	0.327752386
BnaC01g36330D	2.14337484	0.677749304	1.238091108	0.4863658	1.782335147	0.630409225	0.387951319	0.316975765	0.741378473	0.42358616
BnaC01g40920D	0.708660225	0.322164835	0.526068812	0.2860373	-0.631032149	0.280297827	-1.363748271	0.343257952	-1.64385619	0.367926398
BnaC02g00280D	-0.340424439	0.262064532	-2.650764559	0.3551398	-0.207157908	0.238983409	-1.165337732	0.325143542	-1.566700294	0.352440646
BnaC02g01530D	0.748815312	0.294196256	-1.599912842	0.2965805	0.342601663	0.245414157	-0.645716532	0.271768995	-1.599912842	0.314499014
BnaC02g01680D	0	0.22451918	-3	0.2596164	-3	0.249962574	-3	0.26634656	-0.061400545	0.223935767
BnaC02g03940D	-0.857980995	0.356982458	-2.470957872	0.4484197	-0.830500259	0.349171334	-0.514026594	0.31624705	0.560520359	0.360361848
BnaC02g04660D	0	0.22451918	3.841302254	0.2947973	0	0.21244364	2.938599455	0.264316725	3.841302254	0.303865494
BnaC02g09020D	4.95419631	0.384678044	3.807354922	0.2947973	0	0.21244364	0	0.226982722	0	0.223935767
BnaC02g11520D	0.575802501	0.380206682	-0.486797125	0.3220614	-0.51261786	0.337197066	-0.678071905	0.374040121	-1.332237194	0.498386259
BnaC02g14430D	-0.550103744	0.340323277	-0.139857664	0.2570318	-1.125530882	0.431141904	-2.958777337	0.552989274	-1.281975969	0.47528312
BnaC02g18650D	-2.502500341	0.250160714	-0.087462841	0.2263112	-2.502500341	0.237869421	0	0.226982722	1.436099115	0.269226197
BnaC02g20580D	-0.391640048	0.399509933	0.763815532	0.5224691	0.035396211	0.234811459	0.223037547	0.334112684	-0.112043451	0.291532372
BnaC02g21480D	0	0.22451918	2	0.2407733	3.502500341	0.268900366	2.115477217	0.245020078	0	0.223935767
BnaC02g22960D	-2.938599455	0.260714978	-2.938599455	0.257569	-2.938599455	0.248216739	-2.938599455	0.264316725	-2.938599455	0.262328719
BnaC02g24390D	0	0.22451918	3.321928095	0.2716061	0	0.21244364	0	0.226982722	0	0.223935767
BnaC02g24770D	-1.001025005	0.559635774	0.359150559	0.3664425	-0.815575429	0.523871482	-0.559319556	0.442094649	0.042902962	0.247175402
BnaC02g30930D	-2.958693026	0.429684473	-1.486624582	0.3554876	-0.693720816	0.302962855	-2.958693026	0.433968703	-2.005998741	0.419629962
BnaC02g35650D	1.71413196	0.533082796	-0.190942783	0.251977	1.038238804	0.406764064	0.200077835	0.263020008	0.21041978	0.264735021
BnaC02g35790D	3.502500341	0.281262109	3.502500341	0.2775591	0	0.21244364	0	0.226982722	0	0.223935767
BnaC02g40810D	3.544320516	0.285033376	8.156504486	0.734512	5.437405312	0.422300451	0	0.226982722	3.502500341	0.284370707
BnaC02g43290D	3.763872287	0.834491352	2.032228333	0.6404351	0.062365881	0.229362605	-0.050108848	0.237959685	-1.926578931	0.516815492
BnaC02g43540D	-2.938599455	0.260714978	-2.938599455	0.257569	-2.938599455	0.248216739	-2.938599455	0.264316725	1.38332864	0.277028515
BnaC02g44180D	0	0.22451918	0	0.2263112	0	0.21244364	0	0.226982722	0	0.223935767
BnaC02g48800D	-1.728861884	0.642715929	1.52791999	0.6659071	-2.119546207	0.683975307	-2.263608156	0.690439079	-0.354194764	0.381677322
BnaC03g05820D	1.99919917	0.75592439	2.832974286	0.8040702	0.47405928	0.409973934	1.063817022	0.594869492	0.360166255	0.391659181
BnaC03g09750D	2.59454855	0.366814611	4.039138394	0.5140041	-3.058893689	0.249962574	-3.058893689	0.26634656	-0.120294234	0.231498115
BnaC03g14960D	-1.210035215	0.322149424	-2.36923381	0.3484822	-0.210035215	0.238983409	-5.691161905	0.464838934	-1.721535554	0.35821533
BnaC03g17400D	-4.604862058	0.352933795	-0.666262603	0.2554555	-1.545968369	0.276365846	1.69891869	0.389376167	0.939458458	0.304834179
BnaC03g19550D	-0.186413124	0.231266952	-0.186413124	0.2323237	-3.459431619	0.268900366	-3.459431619	0.285733471	1.347923303	0.295682307
BnaC03g23470D	-1.26280669	0.53631248	1.255518618	0.5856119	0.374037603	0.354831537	-0.570798878	0.399298584	0.111705922	0.273475202
BnaC03g25050D	-3.836501268	0.48696238	-2.019365325	0.4032548	-0.514573173	0.293346455	-0.96203215	0.355870672	-0.658963082	0.332318416
BnaC03g25680D	3.166436015	0.51317852	1.798366139	0.3352817	-3.938599455	0.293320036	-3.938599455	0.310144334	-3.938599455	0.310091497
BnaC03g26480D	3.625226371	0.788260858	1.154722595	0.4141261	0.801900492	0.363468245	-1.60E-16	0.226982722	0.851018259	0.396515816
BnaC03g29310D	1.899835838	0.348962186	-3.772589504	0.2922413	0.909234536	0.258590475	-0.898120386	0.254513192	0.423807709	0.248181514
BnaC03g31040D	4.459431619	0.343167688	0	0.2263112	2.736965594	0.243100321	3.700439718	0.298240516	3.662965013	0.294931576
BnaC03g38390D	0.587366083	0.493375515	1.55110889	0.7069199	0.0287964	0.230252034	0.29949194	0.373463313	0.116536632	0.300620399
BnaC03g39000D	1.555678497	0.720926239	2.001398534	0.7505592	-0.065618379	0.250002202	-0.057083026	0.254193966	0.316493234	0.396355102
BnaC03g41530D	-0.268079366	0.355861866	-0.024697902	0.2357339	0.092387299	0.266003117	-0.121148241	0.289528955	-0.258651765	0.376503663
BnaC03g48820D	0.270089163	0.246536951	0.626782676	0.2625863	0.934904972	0.28515226	-0.306103128	0.246798936	-2.387023123	0.302958452
BnaC03g49530D	3.449592228	0.673823928	2.740332445	0.5482383	-5.156504486	0.395014354	-0.265733555	0.252390891	-0.493539473	0.26367387
BnaC03g58550D	-5.437405312	0.430956973	-2.068171503	0.3263126	-1.04508789	0.292327134	-5.437405312	0.436533517	-0.467778961	0.269098506
BnaC03g60650D	-0.604164755	0.429387263	0.589885053	0.4295832	0.388121866	0.378938585	0.229825293	0.320033728	0.186011986	0.316383546
BnaC03g61270D	-0.953913087	0.456241854	-0.927813369	0.4248366	-0.425651173	0.342934323	-1.072808436	0.480630702	-1.00453916	0.501946176
BnaC03g66140D	0	0.22451918	7.306821202	0.6312128	3.841302254	0.28742867	2.938599455	0.264316725	2.874469118	0.258500211
BnaC03g66900D	-4.297680549	0.331045916	-4.297680549	0.3239811	-4.297680549	0.319974286	-4.297680549	0.336195357	-1.024662054	0.268451249
BnaC03g69420D	2.787510111	0.733800944	3.984146049	0.8213899	1.177787119	0.484859893	1.227451556	0.493225809	1.416514914	0.564972348
BnaC03g71900D	4.489561285	0.621501726	-3.222392421	0.2654263	0	0.21244364	0.099535674	0.232863081	1.920565533	0.329040297
BnaC03g77730D	0.866733469	0.247939343	-2.502500341	0.2479504	-2.502500341	0.237869421	-2.502500341	0.253714027	-2.502500341	0.251349554
BnaC04g01080D	-1.768408642	0.37054625	0.56326743	0.2961468	-0.393663848	0.262418983	-0.56358885	0.292912748	-1.768408642	0.386941245
BnaC04g02040D	-0.882791238	0.464218095	-0.878426046	0.4370355	-0.119830436	0.259774913	-0.799964807	0.448732344	0.032816574	0.237371869
BnaC04g03870D	-1.350497247	0.419345961	-0.820676301	0.3411863	-0.046716499	0.223662774	-0.517607233	0.320846102	-0.141043881	0.260327504
BnaC04g09380D	6.037089319	0.499583906	3.222392421	0.2654263	3.273018494	0.259946634	4.345774837	0.338317658	5.247927513	0.425743686
BnaC04g13510D	-0.650444504	0.416723907	-1.487715004	0.5326799	-0.360448589	0.338678714	-1.329952641	0.548689633	-1.051197777	0.542390979
BnaC04g15640D	0.903303673	0.579106344	-1.154420777	0.5788532	-0.017332357	0.222806369	-0.10064952	0.274648632	-0.323261881	0.391392793
BnaC04g25670D	0	0.22451918	0	0.2263112	3.321928095	0.262696379	0	0.226982722	0	0.223935767
BnaC04g34610D	0.970853654	0.25815897	-3.058893689	0.2596164	-3.058893689	0.249962574	0.111031312	0.233114058	0.056583528	0.223935767
BnaC04g35800D	2.831356181	0.793584223	1.316568231	0.5598934	1.026504635	0.532125119	-0.629328756	0.381798408	-0.528138932	0.381120328
BnaC04g38070D	0	0.22451918	0	0.2263112	0	0.21244364	0	0.226982722	0	0.223935767
BnaC04g42060D	-1.563900885	0.283873155	-4.502500341	0.3383595	-0.156725504	0.224312234	-4.502500341	0.351808799	-1.628031223	0.289304396
BnaC04g44080D	0	0.22451918	0	0.2263112	0	0.21244364	0	0.226982722	0	0.223935767
BnaC04g44720D	-1.988318195	0.333681179	-5.357552005	0.4104076	-0.450661409	0.252005618	-0.450661409	0.266645972	-1.942514505	0.341538536
BnaC04g47060D	4.142957954	0.320454225	0	0.2263112	5.101538026	0.388011202	0	0.226982722	0	0.223935767
BnaC04g49870D	3.115477217	0.266122001	0	0.2263112	3.058893689	0.249962574	0	0.226982722	0	0.223935767
BnaC04g50030D	0	0.22451918	0	0.2263112	0	0.21244364	0	0.226982722	0	0.223935767
BnaC04g51210D	0	0.22451918	0	0.2263112	0	0.21244364	0	0.226982722	0	0.223935767
BnaC04g53380D	6.894817763	0.604060992	8.244760234	0.7446964	0	0.21244364	3.662965013	0.295451143	2.736965594	0.256833633
BnaC04g55440D	-2.676110389	0.364584434	-0.932885804	0.2896478	-0.87774425	0.286365318	-1.245476034	0.323397707	0.284143432	0.263057434
BnaC04g55700D	0.792384563	0.509684649	-4.352516415	0.7328389	0.163558783	0.284654708	0.47009235	0.404144211	0.681955057	0.517757758
BnaC05g00680D	-0.964103343	0.526914034	-0.670264491	0.43348	0.34081617	0.362972894	0.96819794	0.577327486	1.271861098	0.692803551
BnaC05g02080D	-0.702733959	0.354536528	1.40852765	0.5225902	0.114536432	0.24317077	0.21801929	0.281488869	0.027250034	0.231969249
BnaC05g02480D	0	0.22451918	0	0.2263112	5.087462841	0.388011202	0	0.226982722	4.115477217	0.322490665
BnaC05g04210D	0	0.22451918	0	0.2263112	0	0.21244364	0	0.226982722	0	0.223935767

BnaC05g09160D	-1.078002512	0.255563334	-3.662965013	0.2864842	-3.662965013	0.278871218	-3.662965013	0.295451143	-1.078002512	0.256893075
BnaC05g11660D	0	0.22451918	0	0.2263112	0	0.21244364	0	0.226982722	0	0.223935767
BnaC05g12270D	5.745954377	0.46631838	0	0.2263112	0	0.21244364	3.36923381	0.279293917	4.922832139	0.39072352
BnaC05g17200D	-4.965434498	0.711133714	0.239284835	0.2959839	-0.987460804	0.474327645	-8.424866117	0.790871905	0.450627279	0.397616594
BnaC05g17550D	0.957771765	0.547736359	1.763236016	0.6846402	-0.490986353	0.383892088	-0.804275529	0.460495262	0.193928957	0.309899961
BnaC05g18050D	2.191550386	0.590294656	3.740812317	0.7603165	-0.516575526	0.276709289	-0.477047162	0.284108722	-6.101538026	0.526172109
BnaC05g20560D	-0.073985711	0.263189528	0.503048714	0.4541834	-0.556011127	0.504447145	-0.538566242	0.486343742	0.159147988	0.331902321
BnaC05g20650D	-2.669278787	0.511465709	0.109163443	0.2494232	-0.634632644	0.337205872	0.638554478	0.375275195	-1.421351273	0.465715154
BnaC05g21820D	3.544320516	0.285033376	4.969626351	0.3742427	0	0.21244364	4.415037499	0.343660837	6.392317423	0.563484096
BnaC05g27390D	0	0.22451918	0	0.2263112	0	0.21244364	0	0.226982722	0	0.223935767
BnaC05g31720D	0.469828601	0.428513245	-0.42685809	0.3867431	0.079442351	0.255926591	0.191541003	0.315701064	0.014058041	0.230135352
BnaC05g36570D	-0.506002879	0.439976752	0.427663393	0.4035102	-0.711193211	0.516234281	-0.122988911	0.286202402	-0.027191484	0.242274737
BnaC05g37110D	1.346802764	0.434032548	0.972293071	0.3494553	-0.742202243	0.291446511	-0.742202243	0.303517648	-1.064130337	0.337901564
BnaC05g38410D	4.052341236	0.874315316	2.569933219	0.7678194	0.194026896	0.287067614	-1.062790373	0.49926468	-0.299371237	0.337674804
BnaC05g39400D	0.96411708	0.610390468	0.149301646	0.2953939	-0.268278192	0.352762073	0.163975735	0.308482159	0.422989623	0.459246979
BnaC05g42130D	1.422116129	0.602141234	4.290062625	0.8661079	0.057561958	0.232365529	0.190441181	0.285343795	-0.03166536	0.234386558
BnaC06g00880D	-5.357552005	0.4240661	-0.657112286	0.2694419	-1.161154792	0.297040667	-1.109624491	0.306181532	1.134301092	0.378711825
BnaC06g03860D	-0.049891805	0.251065554	-0.328733117	0.3809772	0.390240204	0.429156099	0.234096096	0.348975395	-0.329616377	0.427551164
BnaC06g03910D	0	0.22451918	4.273018494	0.3211411	3.415037499	0.266500669	0	0.226982722	0	0.223935767
BnaC06g10050D	0	0.22451918	0	0.2263112	0	0.21244364	0	0.226982722	0	0.223935767
BnaC06g15540D	-1.611194042	0.416994699	-3.425182057	0.4632076	0.437605256	0.302982669	-1.413594082	0.404500863	-2.824789515	0.497184209
BnaC06g17770D	-3.169925001	0.268539311	-3.169925001	0.2654263	-3.169925001	0.256058685	-3.169925001	0.272455881	-3.169925001	0.270666015
BnaC06g20590D	0.125876977	0.285489098	0.601177884	0.4564048	0.340453834	0.37668199	0.440426843	0.415376554	0.198459967	0.334273398
BnaC06g21120D	0	0.22451918	0	0.2263112	0	0.21244364	0	0.226982722	3.058893689	0.264244073
BnaC06g22710D	5.247927513	0.413307091	6.64385619	0.5500458	4.273018494	0.316359329	3.321928095	0.279293917	3.321928095	0.277510656
BnaC06g24360D	3.084316286	0.425869175	4.555725279	0.5979032	2.163975735	0.326526119	1.84502534	0.314078516	2.837569535	0.417470235
BnaC06g25810D	0.283172051	0.262821868	-0.155870911	0.2447295	0.619314006	0.298568548	-5.790076931	0.474607242	-0.352671618	0.266910159
BnaC06g30140D	-0.985644707	0.378841717	0.82984956	0.384077	0.625270489	0.358494928	-7.058893689	0.629555021	-0.567040593	0.337324756
BnaC06g32560D	1.165337732	0.321731128	3.330148602	0.5701768	1.174497731	0.313217708	-0.959358016	0.271245024	1.183599938	0.335035137
BnaC06g32600D	-4.029747343	0.311179066	1.429684275	0.3089247	-4.029747343	0.299295061	-4.029747343	0.316059918	-4.029747343	0.316335112
BnaC06g35730D	4.89077093	0.378714027	5.807354922	0.4550222	4.297680549	0.319974286	0	0.226982722	4.273018494	0.334339445
BnaC06g37920D	0	0.22451918	0	0.2263112	4.029747343	0.299295061	0	0.226982722	0	0.223935767
BnaC06g38250D	0	0.22451918	7.125843933	0.6091686	0	0.21244364	0	0.226982722	0	0.223935767
BnaC06g38580D	-0.116561505	0.290537268	-0.349780341	0.3895897	0.851524175	0.608052415	-0.230483155	0.348220261	-0.089364474	0.286856265
BnaC06g40040D	-1.073373186	0.449111011	1.949959318	0.6638245	1.161510179	0.556243175	-0.52368616	0.353779193	0.180922882	0.286528233
BnaC06g40770D	0	0.22451918	2.502500341	0.2479504	0	0.21244364	0	0.226982722	0	0.223935767
BnaC06g41440D	3.972332251	0.824663602	4.050666716	0.8172444	2.056628861	0.628009528	3.108387793	0.75568442	3.519867472	0.814325091
BnaC06g42850D	0.682947056	0.392343866	0.798156835	0.3975263	0.527076145	0.351623868	-0.056918429	0.241138733	0.868622579	0.470208531
BnaC06g43980D	0	0.22451918	3.058893689	0.2596164	0	0.21244364	0	0.226982722	2.415037499	0.247556272
BnaC07g08360D	0	0.22451918	0	0.2263112	0	0.21244364	0	0.226982722	0	0.223935767
BnaC07g10480D	-2.747648676	0.525617317	-2.827819025	0.5	-0.797230705	0.368556043	-1.182314982	0.428420779	-0.342392197	0.307788668
BnaC07g11010D	0	0.22451918	2.662965013	0.2496566	3.584962501	0.275137377	0	0.226982722	0	0.223935767
BnaC07g13400D	2.345249169	0.720120469	0.819516807	0.4063193	0.74176658	0.412512329	0.90902634	0.455319402	0.226965151	0.292080559
BnaC07g13470D	3.807354922	0.299596675	0	0.2263112	0	0.21244364	0	0.226982722	3.874469118	0.307773257
BnaC07g14220D	0	0.22451918	0	0.2263112	0	0.21244364	0	0.226982722	0	0.223935767
BnaC07g16160D	1.493030379	0.73263632	-1.116429177	0.6215193	0.556475334	0.500039628	0.078634236	0.270331378	0.053601629	0.258900895
BnaC07g16190D	0.816202824	0.441790834	-1.242473083	0.4296801	-0.215506036	0.272442671	0.548456291	0.374220649	1.032983822	0.539872398
BnaC07g20040D	-0.881109394	0.402426996	1.728052802	0.6131446	1.104825976	0.525740604	-0.232953067	0.287034591	0.408592962	0.350285322
BnaC07g20090D	1.505235308	0.440756103	2.325646383	0.5407662	1.680977636	0.472308817	1.183307213	0.393913576	0.873622714	0.351416922
BnaC07g22870D	3.982554866	0.644351686	0.230297619	0.2389834	1.861802706	0.362871623	0.60334103	0.262824069	2.093976148	0.414038008
BnaC07g23080D	0.620172122	0.509768308	-1.30991113	0.6506327	-0.335694815	0.394171158	-0.61279943	0.498148491	-0.625930144	0.553083941
BnaC07g29050D	-0.984403145	0.407968315	-2.225881407	0.4885167	-0.508611615	0.328511924	-0.198631373	0.277673571	-1.177787119	0.465006252
BnaC07g29370D	0.989028882	0.299169573	2.529253068	0.451667	1.868495217	0.388902832	0.909802191	0.292807073	1.886343218	0.415068336
BnaC07g31340D	-1.343888658	0.635574078	0.550363839	0.4416962	-1.259619741	0.634647223	-1.538704835	0.669123604	-1.515305939	0.701336785
BnaC07g31350D	-0.896943716	0.531299535	1.787643759	0.718051	-0.425506244	0.397790517	-2.292026609	0.718685054	-0.589555784	0.48625568
BnaC07g33940D	-5.129283017	0.40141428	-5.129283017	0.3895391	-1.584962501	0.300087622	-5.129283017	0.406962204	-5.129283017	0.413141974
BnaC07g38490D	0.084413205	0.248952059	-0.8259706	0.3907125	-1.056583528	0.459579415	-2.407657969	0.581539064	-0.573907312	0.388698087
BnaC07g39680D	3.053947654	0.695172426	2.271687718	0.5572603	0.936921317	0.358140477	0.988380917	0.374806263	1.73387631	0.536677939
BnaC07g41100D	0.497499659	0.247435186	1.420331799	0.287116	-3.502500341	0.268900366	0	0.226982722	-0.91753784	0.248945454
BnaC07g43950D	-2.169925001	0.340340889	-1.422691072	0.3042838	-1.304854582	0.303275476	-2.276840205	0.344453397	1.858770432	0.500206946
BnaC07g45030D	0	0.22451918	7.142957954	0.6105049	0	0.21244364	0	0.226982722	0	0.223935767
BnaC07g46770D	0.049605546	0.245176389	-1.831042147	0.6417252	-0.495555947	0.419037743	-1.956010246	0.688735073	-1.492288233	0.674411744
BnaC07g46940D	-0.798238911	0.484197224	0.52673928	0.4123824	-0.252828335	0.31945692	-0.412114104	0.376633555	-0.616768909	0.475694811
BnaC07g50590D	0	0.22451918	5.727920455	0.4468038	2.874469118	0.244608387	0	0.226982722	0	0.223935767
BnaC08g04820D	1.117569596	0.494212107	-0.49696602	0.3096666	-0.283135612	0.276500141	-0.514141164	0.32849211	-0.773724144	0.384640618
BnaC08g06130D	1.720167722	0.686568741	2.30243001	0.7345165	0.739209288	0.46713736	1.26350213	0.603603068	0.349726547	0.359690373
BnaC08g07150D	4.64385619	0.35770677	0	0.2263112	4.564784619	0.340631495	0	0.226982722	4.584962501	0.361596921
BnaC08g08940D	0.924741298	0.55110254	-1.454291247	0.5630614	0.159727287	0.284421343	-0.366695604	0.359122371	0.182412789	0.311482881
BnaC08g12710D	0.579996248	0.277699989	0.638491897	0.2753069	-0.622632919	0.253971609	-5.014950341	0.394695128	-1.014950341	0.289947251
BnaC08g12880D	1.771468442	0.486363556	3.766750444	0.7355336	0.630191755	0.29705828	-2.902303326	0.380554528	-1.4119777	0.345138082
BnaC08g15650D	3.36923381	0.275018493	7.815916936	0.6938933	0	0.21244364	0	0.226982722	0	0.223935767
BnaC08g18660D	-0.67656202	0.336869034	-1.577428828	0.4068807	-1.193724536	0.39713005	-1.1428006	0.398136162	-0.700911881	0.355042886
BnaC08g20020D	-4.018859027	0.6638861	-0.444481277	0.3325254	-1.812830052	0.567072634	-5.044394119	0.705257758	-0.31259023	0.330163091
BnaC08g21640D	0.352166672	0.396302265	1.658620437	0.7250013	1.105820619	0.669920568	1.206720983	0.683937881	0.285782278	0.398349713
BnaC08g25150D	-0.335867286	0.28853165	1.349771728	0.4905443	-0.219313028	0.259138663	-1.437405312	0.424079309	-0.541241123	0.331693173
BnaC08g25840D	0.802014897	0.559325355	-1.073048779	0.5846168	-0.729635097	0.531926979	-0.838642994	0.552198915	-0.324474278	0.4027154
BnaC08g28620D	0.012174714	0.22451918	-1.049753035	0.287279	-0.297680549	0.240048963	0.036220188	0.229441861	1.083141235	0.366708937
BnaC08g30700D	-0.788976393	0.326176072	-2.115477217	0.3974471	-0.081529885	0.228981736	-1.538168289	0.394105111	-2.048363022	0.439076315
BnaC08g31500D	1.190983944	0.622351527	0.052971315	0.2439897	-0.546466509	0.414936243	-1.172625317	0.55835667	0.032827242	0.23923879
BnaC08g36140D	0.530514717	0.247479217	2.630974898	0.3905408	0.502500341	0.235216545	0.896164189	0.267429726	-3.584962501	0.291074448
BnaC08g36830D	0	0.22451918	7.459431619	0.650417	3.36923381	0.262696379	0	0.226982722	4.345774837	0.340140547
BnaC08g40750D	2.662965013	0.251618144	0	0.2263112	0	0.21244364	0	0.226982722	0	0.223935767
BnaC08g40860D	3.459431619	0.281262109	0	0.2263112	4.297680549	0.319974286	0	0.226982722	0	0.223935767
BnaC08g41070D	0	0.22451918	6.06608919	0.483277	0	0.21244364	0	0.226982722	0	0.223935767
BnaC08g44670D	1	0.26234413	2.439111634	0.3401119	0	0.21244364	0	0.226982722	0	0.223935767
BnaC09g04040D	3.415037499	0.278780954	0	0.2263112	5.247927513	0.404161823	3.36923381	0.279293917	3.36923381	0.277510656
BnaC09g04110D	0	0.22451918	8.236811481	0.7442055	6.215937399	0.515446123	5.058893689	0.398891296	0	0.223935767
BnaC09g05370D	-0.610990053	0.42368523	-0.774814855	0.4392634	-0.810248549	0.47924152	-0.531701144	0.404003311	-0.852986469	0.519998943
BnaC09g06610D	4.88702529	0.723052943	0.621488377	0.2502268	-1.478047297	0.247023495	-0.584962501	0.247377946	-3.700439718	0.297287242
BnaC09g07480D	0.495145214	0.452781007	1.092608967	0.6180871	-0.098212045	0.26956964	-0.646069964	0.496167089	-0.869048608	0.606969249
BnaC09g11080D	0.445838199	0.387086548	0.021105633	0.2321278	0.111031312	0.260116154	0.378122179	0.364555814	-1.033802708	0.550847159
BnaC09g12830D	0	0.22451918	0	0.2263112	0	0.21244364	0	0.226982722	0	0.223935767
BnaC09g20090D	-0.513270252	0.389488446	0.542088341	0.3995055	0.033074694	0.225329353	-0.141564596	0.280982511	-0.423511006	0.388709095
BnaC09g20100D	5.12722055	0.660090616	2.935459748	0.3573501	1.807354922	0.280934077	0.925999419	0.254513192	1.827819025	0.29725642
BnaC09g27360D	0.287952038	0.332826975	-0.398046783	0.3429431	-0.254414094	0.309014935	-2.229447179	0.651029008	-0.784662336	0.493225809
BnaC09g28190D	-2.478920863	0.566568477	3.585471837	0.8248507	-0.827360614	0.406121209	-3.739685096	0.619592976	0.273861436	0.314831449
BnaC09g28200D	3.969626351	0.308750749	8.232021123	0.7436397	0	0.21244364	0	0.226982722	0	0.223935767
BnaC09g28540D	-0.857980995	0.356982458	-2.470957872	0.4484197	-0.830500259	0.349171334	-0.514026594	0.31624705	0.560520359	0.360361848
BnaC09g35430D	-0.447149684	0.391249692	1.105285905	0.5869571	0.269823571	0.334682888	-0.024708987	0.234338124	-0.030257821	0.239947691
BnaC09g37870D	-6.254241287	0.524212723	0.660642099	0.3163747	-3.315641832	0.431736324	-2.379772169	0.412455088	-1.861923865	0.40602434
BnaC09g39350D	2.502500341	0.250160714	2.502500341	0.2479504	0	0.21244364	0	0.226982722	0	0.223935767
BnaC09g40510D	0	0.22451918	0	0.2263112	0	0.21244364	5.345774837	0.428548469	4.459431619	0.351192363
BnaC09g43740D	1.539556522	0.53187194	-1.715546611	0.3935173	0.242074787	0.261677058	-6.700439718	0.58499771	-1.319617934	0.408657403

BnaC09g45000D	-2.230642844	0.54017181	-1.607575739	0.4672805	0.220146981	0.278188735	-0.597355896	0.360617229	-1.273392492	0.494253936
BnaC09g46030D	0.015767316	0.22451918	-2.807354922	0.3143119	0.192645078	0.231084223	-1.081529885	0.288397355	-2.115477217	0.3198532
BnaC09g49920D	0.940496377	0.583637148	3.909963219	0.8677833	-0.916160407	0.550930818	0.285758358	0.353627285	-0.242542354	0.348420603
BnaC09g50850D	-0.804947086	0.454929726	-2.100913387	0.5984844	-0.679572145	0.433552608	-1.695094417	0.60155562	-2.911718358	0.706860492
BnaC09g51170D	1.014355293	0.328177287	-5.058893689	0.3821991	-0.01449957	0.21244364	-5.058893689	0.398891296	-1.514573173	0.313367413
BnaCnng04580D	-0.599141436	0.473898341	-0.587048364	0.4510066	-0.735042855	0.529210257	-1.810083196	0.719829864	-0.279352252	0.379136725
BnaCnng04890D	0	0.22451918	0	0.2263112	4.169925001	0.310820212	0	0.226982722	0	0.223935767
BnaCnng05070D	0.921997488	0.311469671	0.873087888	0.2938374	-0.062060968	0.217751594	1.378511623	0.373639438	-4.984893108	0.39871297
BnaCnng05080D	-3.938599455	0.305333932	2.816288047	0.4418921	-3.938599455	0.293320036	-0.03170886	0.226982722	0.868755467	0.276231551
BnaCnng05370D	-0.601450624	0.247622319	3.271887412	0.4917177	1.529253068	0.313105428	1.2410081	0.302018387	0.862496476	0.272891789
BnaCnng07330D	0	0.22451918	0	0.2263112	0	0.21244364	0	0.226982722	0	0.223935767
BnaCnng07950D	-3.459431619	0.281262109	-0.090197809	0.2311811	0	0.21244364	-3.459431619	0.285733471	0	0.223935767
BnaCnng08620D	-1.677052693	0.573606855	-2.668924812	0.6100338	0.10445521	0.252844412	-1.46870121	0.554026207	-1.597763783	0.605097925
BnaCnng09130D	0.884522783	0.296639984	2.1740294	0.4276216	-4.700439718	0.351890257	-4.700439718	0.367996847	0.884522783	0.303136778
BnaCnng17120D	0.097202159	0.24481093	-1.665882496	0.3963859	1.651877965	0.568833879	0.803602787	0.385921924	0.96906814	0.448283226
BnaCnng24520D	-1.496425826	0.290301701	0.26607486	0.2439964	0.296132094	0.237506164	1.621031755	0.387181215	-1.60334103	0.300534538
BnaCnng27470D	0.158147834	0.247261263	-0.93128725	0.2955062	-0.898120386	0.294037743	0.286304185	0.26587983	-0.313157885	0.263731111
BnaCnng27900D	4.922832139	0.380200077	0	0.2263112	2.502500341	0.237869421	2.502500341	0.253714027	0	0.223935767
BnaCnng31550D	4.273018494	0.327631301	9.321928095	0.8533631	0	0.21244364	3.36923381	0.279293917	0	0.223935767
BnaCnng33070D	2.025776906	0.78882886	1.381788978	0.676512	1.509688307	0.732576878	-0.154693151	0.303627726	-0.263814874	0.374484836
BnaCnng36390D	0.155448124	0.296283332	-0.107372723	0.2691535	-0.873676428	0.526009194	-1.549135544	0.643829917	-2.0870169	0.728041671
BnaCnng37790D	1.3379805	0.560357885	0.234591358	0.2786885	0.177750565	0.26418243	0.16655815	0.273757001	0.012556172	0.226885854
BnaCnng38100D	0.149576356	0.241935697	-0.828728573	0.2751704	-2.894817763	0.340107524	-1.468553009	0.316980168	-0.765534746	0.2847846
BnaCnng39660D	2.337441094	0.567499736	2.282399731	0.5271034	-0.232173442	0.239285022	1.152490408	0.383863468	1.870031418	0.525874899
BnaCnng39690D	1.336224116	0.542023319	0.420266014	0.3112253	-0.191790148	0.259750696	-0.609108588	0.343301983	0.311112338	0.309930783
BnaCnng47540D	-2.040641984	0.347694089	0.906890596	0.3219733	-5.584962501	0.439688348	-5.584962501	0.453161876	-5.584962501	0.462996231
BnaCnng49280D	7.406559345	0.668694301	0	0.2263112	0	0.21244364	0	0.226982722	0	0.223935767
BnaCnng59450D	5.961931959	0.489071471	0	0.2263112	5.938599455	0.480494206	0	0.226982722	0	0.223935767
BnaCnng60520D	-0.219588498	0.334147909	0.682476996	0.5045902	0.115867552	0.282376096	-0.34260642	0.38612887	-0.403244077	0.446568213
BnaCnng63180D	1.880929518	0.697580929	2.631680353	0.7595504	0.276498375	0.306300416	-0.113683936	0.262940752	0.885419702	0.528021417
BnaCnng67070D	0.109527105	0.27193191	1.502108846	0.6643616	0.359238988	0.373104459	-0.372203661	0.367983638	-0.514192966	0.445575311
BnaCnng69090D	-1.182415946	0.363318539	1.750171133	0.517804	0.994461816	0.401497939	0.857790377	0.378150428	1.091035076	0.453756296
BnaCnng69830D	0.836501268	0.266971802	4.315041709	0.633225	0.855610091	0.25511862	-3.807354922	0.304259572	0.855610091	0.269703935
BnaCnng71740D	1.05611188	0.448041055	-0.979708596	0.3484933	0.226473598	0.262537867	-0.173505526	0.26293855	-1.763084655	0.458628342
BnaCnng73120D	0	0.22451918	3.36923381	0.2716061	0	0.21244364	0	0.226982722	0	0.223935767
BnaCnng73930D	-0.694063938	0.312583659	-0.32334877	0.2659701	0.523067967	0.305430801	-0.309204587	0.273387139	-0.174903496	0.257236518
BnaCnng75020D	3.53003629	0.777977386	5.287526641	0.892749	-3.442943496	0.451182676	-1.23349013	0.369135052	-6.442943496	0.570042534
BnaCnng77210D	-1.742503778	0.430362552	-3.398549376	0.4684825	0.502500341	0.319153105	0.54857835	0.33857524	2.096215315	0.67953917
BnaCnng78540D	-0.839535328	0.247827063	-1	0.2486835	-3.502500341	0.268900366	-3.502500341	0.285733471	-3.502500341	0.284370707
BnaUnng01150D	0	0.22451918	0	0.2263112	0	0.21244364	0	0.226982722	0	0.223935767

BnbZIP

GeneID	log2(Cold/Control)	Prob.	log2(Heat/Control)	Prob.	log2(Drought/Contro	Prob.	log2(Salt/Contro	Prob.	log2(ABA/Control)	Prob.
BnaUnng03970D	2.415037499	0.246506129	3.841302254	0.2947973	2.415037499	0.234239054	2.502500341	0.253714027	2.415037499	0.247556272
BnaUnng03790D	0	0.22451918	5.523561956	0.4262853	4.029747343	0.299295061	4.029747343	0.316059918	4.029747343	0.316335112
BnaCnng73390D	-4.754887502	0.707052027	-2.931765264	0.611456	-0.979593789	0.475468051	-1.023481433	0.480474391	-1.741081703	0.610055831
BnaCnng67350D	-2.502500341	0.250160714	1.270089163	0.2549843	-2.502500341	0.237869421	-2.502500341	0.253714027	0.770518154	0.245229226
BnaCnng41320D	-0.456525887	0.335094579	0.677807457	0.3913884	0.047851518	0.228404928	0.542021979	0.377390891	0.576476035	0.412996671
BnaCnng38290D	0.036994207	0.227088397	-1.874469118	0.2890842	-1.179323699	0.268719839	-1.266786541	0.286644915	-2.68182404	0.319210346
BnaCnng36700D	-2.101086125	0.562242418	-2.450420377	0.5501691	0.463042872	0.360597414	-0.555436548	0.367182271	-0.801041148	0.439375727
BnaCnng30720D	-0.141959993	0.291404681	-0.549463819	0.4265142	-0.167200061	0.298911991	-0.18654591	0.313567755	-0.380095521	0.416074448
BnaCnng29250D	0.327164743	0.334059847	1.459557119	0.6099986	-0.619896291	0.39834531	0.572483561	0.412331801	-0.217109921	0.306538184
BnaCnng27030D	0.673447802	0.508015869	0.824495627	0.5329177	0.389685976	0.40465277	0.493400685	0.441619113	0.380063113	0.429662457
BnaCnng22950D	-0.683972507	0.268878351	-0.155278225	0.2384837	0.689070904	0.279060552	0.73401831	0.296901969	-0.138976413	0.240057769
BnaCnng21800D	0.561213898	0.359457008	-7.351675438	0.6366661	0.132140339	0.24909516	-0.444784843	0.316698369	-1.65123572	0.495995368
BnaCnng21630D	5.594946589	0.447591937	6.528779665	0.5356036	0	0.21244364	5.564784619	0.449998239	0	0.223935767
BnaCnng21210D	0	0.22451918	3.415037499	0.2749172	3.459431619	0.268900366	0	0.226982722	0	0.223935767
BnaCnng20400D	0.359328067	0.257769294	-0.928916902	0.274314	-0.874469118	0.26718095	-2.722466024	0.333740621	-1.343954401	0.306023019
BnaCnng20200D	-0.596502634	0.500209148	-2.047363392	0.7509489	-0.426577632	0.440894801	-0.508951853	0.466637606	-0.201695659	0.352110412
BnaCnng04010D	0	0.22451918	0	0.2263112	0	0.21244364	0	0.226982722	0	0.223935767
BnaCnng01910D	-1.856189975	0.762051323	1.257114879	0.6681879	-0.367618003	0.414302195	-0.466660901	0.451253126	0.035293614	0.25214872
BnaC09g48720D	0	0.22451918	3.874469118	0.2978024	3	0.249962574	2.115477217	0.245020078	0	0.223935767
BnaC09g48640D	-0.503402871	0.370436172	1	0.5115736	0.167215714	0.276834778	0.696060645	0.452525626	0.111933279	0.270690232
BnaC09g46670D	-0.626980971	0.374489239	-1.565828334	0.4803819	-0.13308014	0.254918278	0.442699938	0.353616277	0.731948862	0.467894695
BnaC09g45380D	-0.723187847	0.515895241	-1.255865282	0.6171955	0.212681815	0.326057188	0.127346118	0.293183539	-0.416412711	0.44742682
BnaC09g42010D	-3.502500341	0.281262109	-3.502500341	0.2775591	-3.502500341	0.268900366	-3.502500341	0.285733471	-3.502500341	0.284370707
BnaC09g22200D	-0.685126663	0.290990789	-0.537373042	0.2713485	-0.671051477	0.278739124	-0.881818574	0.306155113	-0.699340522	0.298183275
BnaC09g09660D	0.94236882	0.627624256	-0.145147773	0.3037136	0.314428122	0.39318486	0.189755448	0.331635933	0.484438614	0.511036405
BnaC09g09560D	-2.781563039	0.611328331	0.259777757	0.2969988	-1.296935267	0.508114939	-1.80979672	0.559364983	0.089852755	0.259477703
BnaC09g09130D	0.162699677	0.293394889	0.573735245	0.4252417	-0.838633067	0.500614234	-1.452551446	0.608686463	-1.088395116	0.593883194
BnaC09g06840D	-0.271162287	0.275408169	-0.074853995	0.239208	-0.049468676	0.223662774	0.149975412	0.261463507	0.731280889	0.397394237
BnaC09g02690D	0.00833784	0.229827134	0.199202543	0.3233471	-0.172318385	0.313290359	-0.247935432	0.354846948	0.09047805	0.28568944
BnaC09g00090D	-0.115785025	0.279364366	-1.09986547	0.5610404	-0.082652269	0.25534538	-0.086078518	0.268462257	0.01286898	0.229849149
BnaC08g44990D	-1.407747463	0.399580383	-1.285128177	0.3714907	-1.567527907	0.409520413	-2.718087584	0.469455599	-1.076541555	0.394241608
BnaC08g43230D	-0.72935241	0.257346596	-2.276840205	0.2843575	-4.392317423	0.327201997	0.91753784	0.293080066	-0.807354922	0.262531262
BnaC08g40240D	-0.086100737	0.243520818	-7.048032696	0.5986253	0.145081932	0.248817764	-1.070752773	0.392154532	-2.423541832	0.510226232
BnaC08g35930D	2.491853096	0.31877884	4.098032083	0.4596212	0	0.21244364	0.857980995	0.247538659	-2.415037499	0.247556272
BnaC08g32220D	0.417636634	0.436725052	1.50113071	0.7102553	-0.297544329	0.384420462	-0.627488019	0.520133238	-0.300398839	0.413379742
BnaC08g29000D	-3.301364202	0.805653158	0.405673533	0.4012844	-0.992389684	0.604796312	-2.473810076	0.777325725	-1.06294167	0.659084505
BnaC08g27660D	-0.885653333	0.506109321	-0.014766711	0.2304348	-0.431784574	0.383784212	0.054066846	0.246759308	-0.269808326	0.348579115
BnaC08g25370D	0.583168018	0.491506393	0.953208062	0.5869615	-0.248949577	0.348107982	0.199148598	0.328996266	0.038588498	0.250658266
BnaC08g21370D	3.544320516	0.285033376	0	0.2263112	0	0.21244364	4.437405312	0.346839885	0	0.223935767
BnaC08g20170D	3.792362208	0.75431285	2.682809824	0.5902528	0.670935724	0.300378227	-0.872888082	0.301408556	0.283453947	0.263057434
BnaC08g05560D	0.002107663	0.22451918	0.54359718	0.357469	0.18577383	0.27104028	0.08398467	0.251276903	-0.338367731	0.323197365
BnaC08g01910D	-1.078097423	0.534491793	-0.143580921	0.2754544	-0.287325385	0.328091426	-0.898586373	0.498370848	-0.655864422	0.475767463
BnaC08g01660D	0.268105395	0.273944133	1.32395863	0.450804	0.49410907	0.306502959	0.547220407	0.326977438	-0.37496006	0.289130473
BnaC07g47540D	-0.535127262	0.440749498	-1.108951663	0.5661722	0.121704493	0.276535366	0.520337712	0.448525397	0.679118714	0.553050918
BnaC07g46750D	1.067298389	0.383176582	1.52144605	0.4317936	-0.024522319	0.214971028	-0.498453507	0.280931875	0.238512087	0.261930237
BnaC07g45120D	0.955493615	0.570148209	-2.292063454	0.657517	-0.437240671	0.387912131	-2.116675171	0.681104477	-0.10040837	0.275106555
BnaC07g44670D	0.726943675	0.486574906	2.913232122	0.8052305	0.686333036	0.482347916	1.991348812	0.753771267	2.697013469	0.831384955
BnaC07g29750D	-1.691950478	0.609373349	-1.617491486	0.5698884	0.103126114	0.257406038	-0.357847669	0.350410811	-0.357847669	0.371237539
BnaC07g27440D	-1.211092798	0.680855701	0.01078792	0.2322136	-0.297207395	0.383407746	0.051187455	0.253511483	-0.260756551	0.389728416
BnaC07g27220D	-2.73433561	0.832760929	1.473392691	0.7072436	-0.896473265	0.621642626	-0.477995856	0.463242805	-0.721666597	0.605192592
BnaC07g26080D	-5.183221824	0.405451936	-5.183221824	0.3931739	-0.638901308	0.259818944	-0.520256811	0.267757758	-5.183221824	0.41739318
BnaC06g38430D	0.678071905	0.251006112	1.115477217	0.2670973	1.975752454	0.319452517	0.378511623	0.2436375	2.242856524	0.364236588
BnaC06g30310D	0.884522783	0.244410247	0	0.2263112	2.106915204	0.270635193	-2.115477217	0.245020078	1.823122238	0.273955141
BnaC06g22430D	-0.193177733	0.281105798	-1.283306801	0.4560811	0.097297201	0.246385044	-1.15324626	0.471993994	0.162033459	0.282428934
BnaC06g20630D	0.050209291	0.250217954	-1.055354144	0.6064211	0.215448212	0.338401317	0.542269211	0.481614798	0.290910511	0.402270686
BnaC06g18530D	-0.241574934	0.349175737	2.338754102	0.7955194	-0.191641174	0.323353676	-0.589612288	0.496160485	-0.118254217	0.303744408
BnaC06g16270D	-5.474111514	0.706891314	-0.222392421	0.2830322	-0.596367264	0.380219892	-4.51063739	0.679955264	-1.284286955	0.536748388
BnaC06g15330D	-0.462034979	0.310368893	1.183730801	0.46544	0.010366716	0.213982528	-0.020959619	0.229787506	-0.347292893	0.301520836
BnaC06g02640D	4.2360525	0.871864983	2.108676622	0.6828525	-0.019024826	0.217819842	0.924561865	0.480903695	0.461530085	0.374073145
BnaC06g01290D	-1.852646424	0.594455599	-0.315528972	0.3145761	-1.040886323	0.500997305	-2.477872795	0.641564514	-1.076510232	0.540627532
BnaC06g00420D	-0.750819603	0.48348392	-1.132498506	0.5431197	-0.210844557	0.308143119	0.061948815	0.252756349	0.918459183	0.610870408

BnaC05g51490D	-0.556736078	0.483649037	1.376611148	0.6866216	-0.243936534	0.348394184	0.039404647	0.246369633	-0.257800773	0.384429268
BnaC05g40810D	0.177304532	0.257760488	-0.514573173	0.2853702	-0.587739585	0.295334461	-0.315008681	0.275520448	-0.709484858	0.32801217
BnaC05g35930D	-0.465331326	0.347407887	-2.784852924	0.5704454	0.952112671	0.510257054	-0.575399558	0.373800151	0.377034759	0.357933531
BnaC05g33570D	0.489957234	0.411112139	0.40917458	0.3662312	0.289810534	0.336307637	0.6120121	0.453754095	0.713879285	0.52839348
BnaC05g24270D	0.25352732	0.302414668	0.933647054	0.4679145	0.132325609	0.257958628	0.285948798	0.314355913	0.469090403	0.388764134
BnaC05g17700D	0.227680899	0.294526489	1.066144544	0.5018801	-1.443755008	0.507852954	-0.411333531	0.33270589	-0.764422557	0.430182025
BnaC05g15020D	3.248376741	0.834368065	-0.811629577	0.4223797	0.80996582	0.499372556	1.002436985	0.549955529	0.665978576	0.486596921
BnaC05g06030D	-0.988859442	0.296584945	-0.381870635	0.2554687	-0.022542569	0.214971028	-0.472068444	0.269349484	0.865289691	0.334762144
BnaC05g04130D	-0.171062896	0.293016221	0.645972354	0.4446991	-0.139738785	0.274446088	-0.707488393	0.455728891	-0.291438121	0.351804396
BnaC05g02200D	-0.811927652	0.451475483	3.529010394	0.8370694	0.302624465	0.328983057	-0.401405634	0.353741766	0.374819391	0.379112508
BnaC04g56840D	2.114257179	0.465994751	2.849757364	0.5405549	3.02358682	0.600245694	0.786826109	0.297643894	1.422942697	0.388528567
BnaC04g56770D	1.567040593	0.293575417	-1.432959407	0.248831	3.833827133	0.52396835	-0.584962501	0.243798214	1.05246742	0.268451249
BnaC04g52770D	-2.857042046	0.729256931	0.603626345	0.4436005	-1.071892918	0.571671686	-1.866144253	0.673674222	-0.325219237	0.378672197
BnaC04g52290D	0	0.22451918	2.807354922	0.2544626	0	0.21244364	0	0.226982722	0	0.223935767
BnaC04g52230D	0.652560579	0.451992849	0.520735367	0.3904836	-0.106014569	0.257362006	-0.241685807	0.311148244	-0.395648514	0.378020536
BnaC04g47290D	0	0.22451918	3.874469118	0.2978024	5.48112669	0.428526454	0	0.226982722	0	0.223935767
BnaC04g44430D	0.790076931	0.269430942	1.156504486	0.2888729	2.357552005	0.412232731	-0.061400545	0.23086847	1.357552005	0.322023935
BnaC04g42420D	1.035754713	0.548674222	1.070006193	0.5300072	0.442265233	0.371407059	0.204662043	0.29761087	0.156153581	0.289458505
BnaC04g33670D	-0.103674405	0.269252615	1.865346065	0.7279272	-0.064695768	0.24390609	0.129715544	0.2870478	0.21744002	0.340435556
BnaC04g24820D	-1.006679156	0.456492832	-0.623796895	0.3643026	-0.512670508	0.357691359	-0.348832279	0.324326764	-0.278442951	0.318091955
BnaC04g09600D	-0.071127846	0.246928828	0.360106881	0.3271227	-0.102492017	0.249383564	-0.343500116	0.327246028	-0.216252762	0.303654144
BnaC04g09030D	-0.430634354	0.304169309	1.506495854	0.5236601	-0.220007801	0.260545458	1.265716827	0.511776128	1.292270246	0.550869175
BnaC04g01480D	-0.33616911	0.34406152	-0.77525855	0.4387065	-0.08084967	0.248179312	-0.279585582	0.327626898	-0.268530394	0.339358995
BnaC04g01070D	-0.670064711	0.473180633	2.136223172	0.7595019	0.421929471	0.407552221	1.158817227	0.643981824	1.840853544	0.78400745
BnaC03g76870D	0	0.22451918	0	0.2263112	0	0.21244364	3.906890596	0.310144334	0	0.223935767
BnaC03g65930D	0.795322367	0.476874846	-1.140862536	0.4692751	-0.811554911	0.438981648	-1.662499101	0.565064814	-0.525397947	0.399027792
BnaC03g65600D	-1.042709082	0.504392106	0.894826831	0.4963564	-0.788593994	0.458306915	0.072597129	0.253584135	-0.401790175	0.378020536
BnaC03g61840D	0.325369403	0.380790095	1.262012098	0.6522839	-0.228243961	0.332829177	-0.165333561	0.310604459	-0.118302498	0.297135334
BnaC03g60240D	-0.276673265	0.330568178	-0.506254017	0.384088	-0.480699259	0.396414544	-0.426659419	0.379240199	-0.323309892	0.365634577
BnaC03g54810D	0	0.22451918	0	0.2263112	0	0.21244364	3.969626351	0.313622794	3.36923381	0.277510656
BnaC03g49070D	0.449606013	0.395599968	0.30194372	0.329769	-0.431814865	0.376536687	0.276261196	0.335559107	0.260062839	0.345794146
BnaC03g46620D	-0.323254101	0.350313942	-1.438869515	0.5828776	0.418181889	0.397772905	-0.353416072	0.361544084	-0.361907533	0.389136197
BnaC03g37460D	-0.541764811	0.349805382	0.917666807	0.4461763	-0.1379421	0.254537409	-0.295854583	0.30287039	-0.567527907	0.374420991
BnaC03g25660D	0	0.22451918	0	0.2263112	2.662965013	0.239348868	0	0.226982722	0	0.223935767
BnaC03g23710D	-3.796416354	0.829909912	-0.804731623	0.5237328	-1.743504823	0.730390732	-2.432004928	0.78190056	-0.885771901	0.619328789
BnaC03g22760D	2.502500341	0.250160714	2.415037499	0.244965	2.502500341	0.237869421	2.502500341	0.253714027	2.502500341	0.251349554
BnaC03g21720D	0.729845964	0.543504967	2.274075283	0.7856058	1.47175039	0.729857956	0.599519415	0.497655342	-0.136621546	0.311542323
BnaC03g10840D	-3.459431619	0.281262109	-3.459431619	0.2775591	1.613817363	0.300087622	-0.459431619	0.24372116	-0.289506617	0.23721776
BnaC03g07400D	-5.745954377	0.46631838	0.514573173	0.2809429	0.160936218	0.235650252	-1.400179541	0.336556413	0.902702799	0.360088855
BnaC03g07230D	0	0.22451918	4.321928095	0.3260616	3.415037499	0.266500669	0	0.226982722	3.36923381	0.277510656
BnaC02g43620D	-0.996276914	0.378104195	1.148644081	0.4518123	0.355094959	0.293132904	0.907635982	0.423993448	0.331588206	0.304123076
BnaC02g41050D	-0.03974174	0.243179577	0.296544344	0.3578146	-0.062517998	0.247917327	0.297191147	0.370561661	-0.131345436	0.304255169
BnaC02g39310D	-0.38358604	0.420290429	0.037993132	0.2466294	-0.287208555	0.381963525	0.011594013	0.232860879	-0.522033183	0.528210751
BnaC02g38190D	-4.736965594	0.365418824	-0.255838904	0.2417045	-1.152003093	0.272033182	-1.036525876	0.281693614	-1.112474729	0.283910582
BnaC02g35400D	-3.700439718	0.29336847	-3.700439718	0.2890842	-1.115477217	0.243250026	-3.700439718	0.298240516	-3.700439718	0.297287242
BnaC02g06270D	-0.983716587	0.517634471	-4.925017214	0.756532	0.303801477	0.341818134	-0.0256082	0.235967276	0.381280229	0.399708074
BnaC02g01780D	-1.125190262	0.464460266	0.751135367	0.4180008	-0.307943865	0.301575874	-0.699971387	0.392610254	0.162829424	0.282428934
BnaC02g00560D	-1.22571278	0.545950896	-0.792753372	0.4346248	-0.23974234	0.305527669	-0.363565385	0.349004016	0.159940913	0.297742964
BnaC01g43800D	0.239276915	0.281576931	1.406174223	0.5169058	1.545880335	0.584414298	0.577445651	0.360324421	1.817873905	0.665011096
BnaC01g30400D	-1.411598415	0.507249727	-2.591865361	0.5649481	-0.375416079	0.323186357	-0.39339382	0.332859999	-0.548415105	0.388365652
BnaC01g04330D	1.262658263	0.643173853	1.424129062	0.644224	0.277627996	0.335616348	1.525326881	0.69872794	1.692665495	0.755834126
BnaC01g03810D	-0.091660188	0.259231128	-1.486877501	0.5577821	0.064681841	0.240121614	-0.027445882	0.235967276	1.33958147	0.690712072
BnaC01g03460D	1.03072194	0.494733876	0.72882152	0.3941712	0.03539086	0.223402991	0.060803279	0.243034274	0.323837685	0.323679506
BnaC01g02720D	-0.06871275	0.228660309	0.618129365	0.2688982	-1.10433666	0.271584064	-1.033947332	0.284659111	-1.033947332	0.286499613
BnaC01g02130D	0.410441581	0.422791398	2.133232102	0.7761193	-0.410611764	0.42398244	0.002806619	0.228197982	0.200094004	0.34918014
BnaC01g01320D	0.190301137	0.275295889	2.131040863	0.6631706	-0.146363501	0.252553806	-0.41592998	0.317431488	-0.384143491	0.320577512
BnaAnng39310D	0.251731757	0.305177622	1.81400277	0.6607335	0.815831281	0.478836433	0.642246937	0.423544331	0.017584312	0.231134859
BnaAnng26550D	0.660011695	0.459984501	2.233727491	0.7456849	0.577403084	0.439560657	1.585945598	0.693943957	1.8552995	0.762218641
BnaAnng11690D	0.652945005	0.43617026	0.039048726	0.2369998	0.498238789	0.390052045	0.237328692	0.308250995	0.07836165	0.258189792
BnaAnng07530D	-0.490718699	0.317561379	1.545923144	0.541332	-0.153103967	0.250112279	0.934142918	0.442281782	0.677659176	0.403072053
BnaAnng05020D	-4.247927513	0.327631301	-4.247927513	0.3211411	-0.925999419	0.24922285	0.593374741	0.265785163	-4.247927513	0.334339445
BnaAnng04720D	-0.954833684	0.347742524	1.232552288	0.4341404	0.665913392	0.341153264	0.57985561	0.332386664	0.89151537	0.412529941
BnaAnng01920D	-0.625192346	0.358741502	-0.514573173	0.3236751	0.023846742	0.217819842	-0.845114398	0.396247226	-0.330973234	0.314252439
BnaA10g28780D	-0.100674793	0.271652313	-0.422496057	0.3876017	0.180689457	0.307150217	0.648884892	0.499815069	1.027044538	0.665816866
BnaA10g24100D	0	0.22451918	3.841302254	0.2947973	0	0.21244364	0	0.226982722	0	0.223935767
BnaA10g24020D	-0.167186919	0.280782169	0.713089618	0.4263667	-0.011741225	0.21572396	0.32105081	0.330918225	0.023199616	0.231867977
BnaA10g22150D	-0.294124984	0.295153933	-1.9878458	0.4834135	0.002439045	0.21244364	-1.002443176	0.42114023	-0.92624355	0.430466026
BnaA10g21200D	-0.360100497	0.396278048	-1.06270446	0.5971217	0.035766204	0.236200641	-0.020872278	0.236286502	0.133089004	0.307826095
BnaA10g18440D	0	0.22451918	3.459431619	0.2775591	4.415037499	0.327201997	0	0.226982722	3.502500341	0.284370707
BnaA10g13430D	0.795322367	0.476874846	-1.140862536	0.4692751	-0.811554911	0.438981648	-1.662499101	0.565064814	-0.525397947	0.399027792
BnaA10g04020D	0.344187173	0.333562295	-0.08871172	0.2518141	-0.361253752	0.324148438	-0.685156789	0.405068865	-0.39116557	0.353660309
BnaA10g02300D	-0.821413198	0.485447709	2.507541901	0.7807844	-0.931144638	0.519503593	-0.275352048	0.332591409	-0.386197816	0.392720332
BnaA09g49720D	-1.298162629	0.574174856	-1.401331382	0.5566923	-1.095645575	0.552535753	-1.320689198	0.581252862	0.079567257	0.264613935
BnaA09g48940D	-2	0.241636285	-2	0.2407733	0.874469118	0.22873296	1.459431619	0.258938321	-2	0.242580753
BnaA09g43320D	0	0.22451918	2.584962501	0.2496566	0	0.21244364	0	0.226982722	0	0.223935767
BnaA09g39870D	0.306857095	0.382987249	1.424848005	0.6932174	-0.391683288	0.421298742	-0.592491923	0.495872081	-0.739267732	0.596027511
BnaA09g37270D	-3.901748257	0.82724823	0.271325262	0.3456731	-1.358586067	0.676840061	-2.883626239	0.795977315	-1.596941921	0.738954789
BnaA09g36130D	-0.806149159	0.479384621	0.030239613	0.2360047	0.042784866	0.230945525	-0.00644781	0.229701645	-0.105060601	0.277215647
BnaA09g36010D	0	0.22451918	4.196397213	0.316126	0	0.21244364	0	0.226982722	0	0.223935767
BnaA09g34460D	0.831457288	0.580378844	0.989554285	0.6016305	-0.290435713	0.370878685	-0.303322398	0.374665363	-0.220590588	0.360309011
BnaA09g30910D	1.528928466	0.543896844	2.018430705	0.598632	0.004116108	0.21244364	-0.814586491	0.348154215	-0.063193826	0.239714326
BnaA09g24670D	-0.655450108	0.429486333	1.072353515	0.5540658	0.149248731	0.276718095	-0.07565671	0.257069199	0.044513237	0.243578058
BnaA09g24610D	0.619614689	0.408302952	0.187061454	0.276641	0.040443826	0.226168146	0.426140333	0.35383203	0.46670858	0.388090458
BnaA09g19470D	-1.270089163	0.329872486	-0.537373042	0.2713485	-0.313157885	0.24968958	-0.881818574	0.306155113	-0.699340522	0.298183275
BnaA09g09500D	0.694222238	0.547428141	-0.045907162	0.2521003	0.381530058	0.427797739	0.304126116	0.384400648	0.4346027	0.486632146
BnaA09g09300D	-3.662965013	0.290878509	-3.662965013	0.2864842	-3.662965013	0.278871218	-3.662965013	0.295451143	-3.662965013	0.294931576
BnaA09g08870D	0.520024587	0.396967135	0.243363019	0.2976725	0.326450237	0.332206136	-0.656651239	0.409588661	-0.63994836	0.433019832
BnaA09g03330D	-1.367277082	0.651222745	2.648605534	0.8070974	-0.391730126	0.397953433	-0.302092913	0.36094526	-1.777659221	0.740832717
BnaA09g00980D	-0.228361828	0.305547483	-0.510555812	0.3670414	-0.594743522	0.409093311	-0.626161618	0.416759132	-0.611194042	0.442559178
BnaA09g00170D	0.068225243	0.251563105	1.707409273	0.6861086	1.188819313	0.630664606	0.292995603	0.338997939	0.208603416	0.322673395
BnaA08g28740D	-0.454747209	0.347546585	0.554172109	0.3754954	-0.593145705	0.378108598	-1.448052557	0.522202702	-1.206701977	0.526405474
BnaA08g28440D	-2.677408749	0.503559918	1.807828636	0.5886193	0.18932472	0.259750696	-0.03355256	0.23153334	0.086877451	0.249192029
BnaA08g20970D	2.827254479	0.533014548	2.429987841	0.450575	-0.192645078	0.228083501	0.795859283	0.288276269	-0.263034406	0.244471891
BnaA08g16860D	-1.223488278	0.416554387	-0.091576602	0.2444389	-0.701261532	0.344402762	-0.250600123	0.280427718	0.277205306	0.297146342
BnaA08g15400D	0.609530926	0.478570045	1.269765775	0.6380949	-0.412041902	0.395901582	-0.155848667	0.299878474	0.062090872	0.259726479
BnaA08g11080D	-0.64992723	0.43013359	-8.997179481	0.8242035	-0.160077215	0.279474444	-3.035247522	0.698703723	-1.110466767	0.574282733
BnaA08g10920D	-0.066777599	0.25906381	0.337612657	0.3735514	0.031732503	0.229957026	-0.007906692	0.231467294	-0.401865406	0.441522245
BnaA08g04710D	1.584962501	0.480485399	1.502500341	0.4366942	0.215937399	0.245579274	0.554588852	0.306921255	0.723376529	0.343911814
BnaA08g04620D	0.970285149	0.617829529	2.251944224	0.7838291	0.248103405	0.349510374	0.460059401	0.441418771	0.39393984	0.448846824
BnaA07g33790D	-1.417611443	0.347253778	1.497499659	0.4338212	0.194572527	0.242776692	0.90843276	0.361222657	1.486528542	0.488545299
BnaA07g27320D	0	0.22451918	-5.544320516	0.4285463	-1.02075856	0.296723643	-1	0.306434711	-0.807354922	0.296697224
BnaA07g21710D	0.56711187	0.432033534	-2.590676152	0.6444728	0.177718249	0.288916922	0.115915793	0.274796136	0.764639982	0.536299271
BnaA07g20770D	-0.735311127	0.374630138	0.54722639	0.3492374	-0.829247528	0.388055233	0.096055105	0.254209377	0.545526101	0.389228663

BnaA07g19330D	0.817894875	0.533538518	2.728835549	0.8015957	-0.075345125	0.251261492	-0.103873923	0.270844341	0.293042281	0.369876977
BnaA07g16990D	-2.196397213	0.329159181	-5.196397213	0.3956638	-5.196397213	0.399041002	-0.781359714	0.280476153	-5.196397213	0.420514988
BnaA07g01890D	0.501951897	0.475032143	-1.888896949	0.7564726	0.086857405	0.271502607	-0.126279459	0.301465797	0.405624559	0.477544119
BnaA06g39730D	-0.801454321	0.303262267	-0.886343218	0.2980732	-1.386416821	0.337573532	-1.306246473	0.342687749	-5.930737338	0.50404426
BnaA06g37290D	-0.973788317	0.547163954	-1.07928891	0.5438924	-0.134475831	0.277512857	-0.066537002	0.257914597	-0.023332504	0.235425693
BnaA06g37000D	2.415037499	0.246506129	0	0.2263112	0	0.21244364	0	0.226982722	0	0.223935767
BnaA06g36330D	0.490520454	0.43887157	1.039883396	0.5922518	0.012671436	0.217797827	0.024021723	0.235410282	-0.093711679	0.277330128
BnaA06g33560D	-1.067976794	0.346130984	-6.315904307	0.5125004	0.796100718	0.353840836	-6.315904307	0.538401758	-0.751119688	0.325088503
BnaA06g29500D	-2.164245905	0.801419564	1.828795733	0.7537911	-0.530497474	0.494397038	-0.623148668	0.518876149	-0.978282539	0.67941148
BnaA06g29270D	-1.273898277	0.694553788	-0.085149669	0.2730327	-0.240238284	0.353713146	-0.326456009	0.395551534	-0.562384757	0.542005706
BnaA06g27230D	-2.001205763	0.608259361	-1.075460317	0.4746094	-0.157773076	0.273230829	-0.68993206	0.424220209	-0.75556562	0.470758921
BnaA06g25370D	-0.745564723	0.437079503	0.348340786	0.33345	-0.691697851	0.429103262	-0.676669642	0.42372706	-0.433076324	0.383376924
BnaA06g24140D	-0.233450139	0.325702737	0.566596618	0.4427617	-0.075948853	0.253901159	0.384758295	0.390769752	0.292731419	0.377923668
BnaA06g13700D	3.016633112	0.79520677	0.160822794	0.2653999	0.727406766	0.423716052	0.38928973	0.332201733	0.696945662	0.443270281
BnaA06g08590D	-0.784271309	0.244328789	-3.36923381	0.2716061	1.584962501	0.293978301	1.600392541	0.307196449	-0.047305715	0.223935767
BnaA06g04770D	-1.299560282	0.252016626	-0.607682577	0.2423496	1.044394119	0.257412642	-1.415037499	0.258911902	0	0.223935767
BnaA06g03040D	1.66279437	0.597077653	0.35968541	0.2960544	-0.530874985	0.31882067	0.698193501	0.387201029	0.20515349	0.280421114
BnaA05g26790D	-0.178748852	0.262066734	0.888264762	0.3892551	0.217356831	0.261969865	0.907151222	0.41983911	0.162520965	0.264963983
BnaA05g22650D	0.50593802	0.417965585	-3.145886435	0.6842923	-0.006702428	0.21572396	-0.103062536	0.267993325	0.38056766	0.398651326
BnaA05g20870D	0.566769384	0.451671422	-0.16189167	0.286548	0.305371029	0.353473176	0.623783632	0.470397865	0.74066171	0.555448413
BnaA05g11370D	0.457925701	0.415499841	0.546195152	0.430105	0.097135848	0.263101465	-0.16942554	0.301120152	0.063112265	0.258458382
BnaA05g08520D	-0.722852858	0.391612949	0.357834988	0.3160357	-0.326285962	0.304732907	0.040050947	0.236403184	-0.424090748	0.34810578
BnaA05g08020D	-0.698163323	0.440927824	2.168218679	0.7404959	0.267981979	0.323276621	0.971421255	0.558189352	0.687989838	0.511615414
BnaA05g02840D	-7.285402219	0.653261386	0.566346823	0.340834	-2.98772167	0.526326218	-4.410933101	0.579636919	-1.815082284	0.501926362
BnaA05g01970D	0	0.22451918	0	0.2263112	0	0.21244364	0	0.226982722	0	0.223935767
BnaA05g01800D	-0.817939673	0.419689404	0	0.2263112	-0.237943425	0.28725915	-0.881799615	0.433534996	-0.717412797	0.43051446
BnaA05g01520D	-0.913272348	0.489124309	1.626022705	0.6677256	0.458120356	0.396009458	1.158237119	0.609428388	1.899245203	0.767522192
BnaA04g27730D	-0.912537159	0.264435609	-0.093109404	0.2311811	-4.415037499	0.327201997	0.539158811	0.265380077	-1	0.272303973
BnaA04g23690D	0.328948523	0.364265208	1.062829337	0.5824659	-0.199026466	0.305739019	0.118181426	0.281286326	0.069830685	0.263548381
BnaA04g23630D	-0.111634182	0.274668446	1.357077699	0.6537964	-0.6321467	0.473502061	0.080813937	0.264312322	-0.040919773	0.247175402
BnaA04g23410D	-4.029747343	0.311179066	0.555215157	0.2523997	-0.756728849	0.242072193	-1.527247003	0.27374159	-0.756728849	0.255675614
BnaA04g20520D	-0.006869989	0.225866533	-0.360124418	0.2853174	1.129380923	0.490528902	-0.508223057	0.32142291	-0.006869989	0.225774067
BnaA04g18340D	0.643941177	0.40202191	0.99467836	0.4678727	1.051138849	0.521518018	-0.343872683	0.31281042	-0.043133109	0.238483656
BnaA04g12020D	-0.087413606	0.269430942	0.981633254	0.5893678	0.02576542	0.229481489	0.091319368	0.273281465	0.253197411	0.372380147
BnaA04g05810D	-0.449211357	0.38923967	-2.200207568	0.6519867	0.749751749	0.517467153	0.498212982	0.422549227	0.064874666	0.256767586
BnaA04g02670D	1.443606651	0.280317641	-3.058893689	0.2596164	-0.120294234	0.21934332	-0.943416472	0.247538659	-0.943416472	0.245312885
BnaA03g56410D	-2.711494907	0.434292332	-2.126532406	0.392509	-2.448460501	0.422544824	-1.36099766	0.377095882	-1.389566812	0.394342879
BnaA03g54260D	1.116723606	0.334401088	1.912283854	0.4101479	0.890588783	0.293870425	1.292450894	0.357229032	-1.016301812	0.286499613
BnaA03g52920D	0.80454272	0.539075434	-1.670126477	0.6381213	-0.398824455	0.389618338	-0.502164205	0.422890468	0.501046145	0.474248388
BnaA03g39740D	0	0.22451918	5.922832139	0.4675777	0	0.21244364	0	0.226982722	0	0.223935767
BnaA03g39300D	0.046645665	0.247672954	-1.303537235	0.618195	-0.022251124	0.223979799	-0.101903374	0.276614622	-0.0725828	0.268239899
BnaA03g35190D	-0.944555673	0.465446564	1.282085677	0.5772636	0.058032121	0.235969478	0.869075859	0.502817993	0.655009998	0.475349167
BnaA03g34610D	0.825117994	0.581517049	-3.42170123	0.8205181	0.170529272	0.312814823	-0.1714746	0.316845873	-0.095730837	0.290449206
BnaA03g32170D	0.183660027	0.275835271	0.888299734	0.4312102	0.04930764	0.227471468	0.339024089	0.316749005	0.002386593	0.223935767
BnaA03g19810D	-3.680559073	0.805846895	-0.851867689	0.519508	-1.596745327	0.695865916	-2.897430266	0.78270633	-1.25614802	0.681172725
BnaA03g19140D	0.770073906	0.302821956	1.712281331	0.4106982	0.927354698	0.315630614	0.716207034	0.3011884	-0.065887936	0.234073937
BnaA03g18190D	-0.143945202	0.301747596	2.710557853	0.8180964	-0.128379458	0.291893427	0.308511361	0.384365423	-0.365855491	0.44478275
BnaA03g05670D	-4.415037499	0.338183363	0.409390936	0.2492317	1.21916852	0.310936895	-0.093109404	0.232581281	-0.04580369	0.229285551
BnaA02g32330D	-0.536439158	0.442063828	0.333042245	0.3603222	-0.06629706	0.250002202	-0.12920256	0.288553665	-0.265439471	0.365892159
BnaA02g31000D	-0.936769142	0.621030593	0.20612775	0.3310811	-0.409693866	0.439767604	-0.241366755	0.354750079	-0.607910691	0.564298672
BnaA02g27460D	-1.160464672	0.279703406	-4.662965013	0.3491515	0.971241007	0.293938673	0.395928676	0.258542041	0.395928676	0.257069199
BnaA02g20110D	-0.784271309	0.244328789	-3.36923381	0.2716061	1.584962501	0.293978301	1.600392541	0.307196449	-0.047305715	0.223935767
BnaA02g17180D	-0.781359714	0.410134647	-0.903615464	0.4068147	0.274645696	0.302773521	-3.348400306	0.629187361	-0.101410521	0.261357832
BnaA02g02830D	-0.476206031	0.387179013	-5.663558104	0.7709016	0.360196249	0.361616735	0.155024073	0.291259379	0.423904737	0.41288219
BnaA02g00920D	-1.190554369	0.671774279	-0.938095439	0.5841303	-0.755371789	0.570669978	-0.867851361	0.592009669	-0.325939975	0.425433266
BnaA02g00310D	-0.220837583	0.318107366	0.05606737	0.2497556	0.528448976	0.447646976	0.039134136	0.24213824	-0.132495172	0.293108686
BnaA01g31420D	-0.328758069	0.375303815	1.669798899	0.7188017	-0.106420594	0.273974955	0.086183273	0.271907693	-0.022219486	0.236909542
BnaA01g30760D	-0.172734535	0.255492885	1.514011704	0.472562	0.804947086	0.360766934	0.424828002	0.298211895	0.189132305	0.261930237
BnaA01g27940D	2.250672055	0.7697964	-3.4325419	0.6689783	1.050266056	0.583874916	0.637579044	0.45077759	0.591905224	0.470281183
BnaA01g26200D	0.548298366	0.436249516	0.76231419	0.4820903	-0.107092967	0.262667759	0.247157049	0.327329688	1.23869547	0.681542587
BnaA01g02570D	-1.041542262	0.533360192	-0.639531443	0.4184257	0.0818933	0.250697894	0.671065883	0.477218289	1.746462982	0.760585086
BnaA01g01100D	0.171302276	0.312143348	1.219473668	0.647643	0.077948119	0.259598788	0.091105706	0.273323294	0.147008064	0.316632322
BnaA01g00310D	0.242869153	0.318730406	1.512015481	0.6503025	-0.051106496	0.234809257	-0.443714252	0.376686393	-0.421928175	0.395648403

BnMYB

GeneID	log2(Cold/Control)	Prob.	log2(Heat/Control)	Prob.	log2(Drought/Contro	Prob.	log2(Salt/Control	Prob.	log2(ABA/Control)	Prob.
BnaA01g00670D	-1.594191975	0.736786255	0.184572523	0.3160709	-0.371226942	0.416409085	-0.809015674	0.575942707	0.27294327	0.395615379
BnaA01g00920D	-0.477425287	0.412860175	1.466182257	0.6693812	0.191522897	0.30671651	0.60852364	0.474591831	0.663881565	0.53778312
BnaA01g02240D	0.892629902	0.475875339	-1.444237476	0.4653761	-0.358352903	0.312244619	-0.760812336	0.398768009	-0.41241203	0.344673553
BnaA01g02690D	-0.437981699	0.376943975	0.516214612	0.4005315	-0.053716548	0.23519453	0.0542877	0.245773011	-0.159803807	0.298211895
BnaA01g04220D	-0.160464672	0.230976346	-1.247927513	0.2518823	-0.247927513	0.224693103	-1.788495895	0.266661383	-0.855610091	0.252483356
BnaA01g05400D	0	0.22451918	0	0.2263112	4.807354922	0.36131292	0	0.226982722	0	0.223935767
BnaA01g08890D	-1.553064149	0.6019519	-1.954523242	0.6101945	0.016468311	0.220199725	-0.339468676	0.347612632	-0.148617052	0.293804378
BnaA01g10210D	0	0.22451918	0	0.2263112	0	0.21244364	0	0.226982722	2.874469118	0.258500211
BnaA01g11280D	0.140177658	0.253113002	1.784746114	0.5378404	-1.444784843	0.387031509	0.117740269	0.251246081	0.987679728	0.439587076
BnaA01g14800D	-3.841302254	0.299596675	-1.033947332	0.2516556	-0.06871275	0.218207316	1.516249751	0.323093892	3.04541046	0.514517066
BnaA01g17750D	-0.585378803	0.422020853	0.300964415	0.3302115	0.074624809	0.245922717	0.076994416	0.259497517	0.782040676	0.551703565
BnaA01g21010D	1.622115499	0.424768396	-0.187278568	0.2433711	-0.419225296	0.248971873	0.678071905	0.298178872	0.334419039	0.261549368
BnaA01g21090D	-0.88608668	0.433068266	1.09024912	0.5155342	-1.048209093	0.468022385	-3.449307401	0.63576121	-0.927354698	0.471023108
BnaA01g21650D	-1.294943654	0.484939149	-0.077167861	0.2466074	-0.344171169	0.309611557	-0.209923069	0.287877787	-0.181950477	0.284698739
BnaA01g23930D	-3	0.262619324	-3	0.2596164	-3	0.249962574	-3	0.26634656	-3	0.264244073
BnaA01g30920D	0.834207079	0.561533517	-1.384861618	0.6244958	1.000711739	0.62676785	0.698878137	0.521724964	-0.347243909	0.409099915
BnaA01g31080D	0.643608496	0.329165786	-3.163746427	0.4085033	2.323165929	0.633843654	1.310739538	0.457443904	-0.43792139	0.288005477
BnaA01g32200D	-1.434440031	0.66292402	-3.378786973	0.778814	0.605239164	0.500123287	0.172675612	0.310604459	0.173591319	0.324599757
BnaA01g32800D	0.202492864	0.243029871	0.704993204	0.269836	-2.102361718	0.288754007	0.167727446	0.242792103	-4.604862058	0.361596921
BnaA01g33290D	-0.86236387	0.440661436	-1.774901028	0.5337477	-0.525660422	0.371347617	-0.342496397	0.327800821	-0.280941726	0.32372794
BnaA01g34510D	0	0.22451918	0	0.2263112	0	0.21244364	0	0.226982722	0	0.223935767
BnaA01g37390D	0.553153762	0.279392987	-5.156504486	0.3919806	-0.041027268	0.216465885	-1.934112064	0.323305241	-1.013546532	0.29657834
BnaA02g00440D	2.584962501	0.251618144	0	0.2263112	2.502500341	0.237869421	0	0.226982722	3.969626351	0.31401247
BnaA02g01290D	2.874469118	0.256930501	0	0.2263112	2.736965594	0.243100321	3.736965594	0.298240516	2.807354922	0.258500211
BnaA02g02300D	-5.309855263	0.419385589	-5.309855263	0.4060684	-0.987927168	0.283457061	-5.309855263	0.42492911	-1.987927168	0.341723467
BnaA02g02580D	-1.626826019	0.550794322	-2.046421801	0.5540086	0.220918027	0.291750326	-0.715996529	0.414839374	0.365187511	0.36365978
BnaA02g03170D	1.457045026	0.331583096	-1.611434712	0.2682641	2.279336218	0.419876537	-0.026472211	0.226982722	1.876851769	0.392326253
BnaA02g03510D	0.785261151	0.418022826	-1.50622583	0.4323462	1.881554812	0.659379513	0.640615558	0.383709359	0.682947056	0.419251295
BnaA02g03700D	0	0.22451918	0	0.2263112	2.662965013	0.239348868	2.662965013	0.255299148	0	0.223935767
BnaA02g06100D	-3.584962501	0.287446282	0.353636955	0.2392762	-1.08246216	0.239685706	-3.584962501	0.291867008	-3.584962501	0.291074448
BnaA02g07870D	-1.736965594	0.237046039	1.432959407	0.248831	0	0.21244364	0	0.226982722	1.963474124	0.269741361
BnaA02g09340D	0	0.22451918	0	0.2263112	2.222392421	0.233252756	2.222392421	0.248806756	0	0.223935767
BnaA02g10750D	-0.647190801	0.332507749	0.388142272	0.2954445	-0.139789283	0.245207211	-2.090320607	0.464526313	-2.139789283	0.4902427
BnaA02g15320D	-2.329747599	0.596564691	-8.268347055	0.747389	-0.520154205	0.364192557	-1.59121471	0.544134612	-3.922572218	0.694765138
BnaA02g16430D	0	0.22451918	0	0.2263112	3.058893689	0.249962574	0	0.226982722	0	0.223935767
BnaA02g16690D	0	0.22451918	0	0.2263112	0	0.21244364	0	0.226982722	0	0.223935767
BnaA02g17000D	-3.321928095	0.275018493	-3.321928095	0.2716061	-0.152003093	0.219805647	0.765534746	0.257458875	-0.152003093	0.231936225
BnaA02g20260D	0	0.22451918	5.247927513	0.4002409	0	0.21244364	0	0.226982722	5.345774837	0.436502695

BnaA02g25380D	-0.566693493	0.425281359	-0.415758667	0.3635629	0.232553372	0.316638927	0.980035352	0.578172884	0.37111645	0.400916728
BnaA02g29490D	-1.068064275	0.507938814	0.401679116	0.3513641	0.355658952	0.351154937	-0.872807984	0.469413769	-0.234074226	0.318450808
BnaA02g30960D	-3.584962501	0.287446282	-3.584962501	0.2834218	0.473931188	0.235135088	-3.584962501	0.291867008	-3.584962501	0.291074448
BnaA02g31210D	2.189824559	0.298751277	3.851749041	0.4303119	-0.540568381	0.224873631	0.700439718	0.243811423	-2.415037499	0.247556272
BnaA02g33410D	-0.18473988	0.252351263	1.945218714	0.5206748	0.921612101	0.364234387	0.542734265	0.309591743	2.186780897	0.627756349
BnaA02g36430D	-0.862496476	0.240672003	-0.074000581	0.2263112	-2.736965594	0.243100321	-0.862496476	0.243873067	-2.736965594	0.256833633
BnaA03g00640D	-0.468679114	0.424396333	-5.812787444	0.8632701	0.298972746	0.365022544	-0.072270286	0.261065025	0.238057574	0.360196731
BnaA03g01420D	0.431735545	0.404756244	2.249370577	0.7675288	0.133579346	0.278496953	0.273076407	0.343504526	0.176317818	0.31998089
BnaA03g02170D	-0.040641984	0.22451918	-3.584962501	0.2834218	0.502500341	0.235216545	-3.584962501	0.291867008	-0.921997488	0.252635264
BnaA03g02860D	-1.46712601	0.266606344	-3.969626351	0.3032513	0.674229839	0.248102258	-1.46712601	0.269972525	0.376148486	0.248040614
BnaA03g03390D	-1.050626073	0.265774155	-0.243271151	0.2390186	-1.334419039	0.261859787	-1.273018494	0.273477403	-0.334419039	0.244663426
BnaA03g04050D	5.014950341	0.388874212	2.736965594	0.2526947	4.64385619	0.34721415	4.297680549	0.336195357	3.700439718	0.297287242
BnaA03g05370D	-2.494033535	0.624887721	-1.244913875	0.4863658	-0.536186986	0.379367889	-1.146649859	0.506864455	0.249338186	0.324430237
BnaA03g06490D	2.620661665	0.823263412	-3.188557853	0.7527519	0.420646612	0.413412766	0.767322714	0.536360914	0.831396753	0.602053172
BnaA03g06730D	0	0.22451918	0	0.2263112	0	0.21244364	0	0.226982722	0	0.223935767
BnaA03g09270D	-0.780420523	0.521764592	-1.686036653	0.6639654	0.362982419	0.39222278	-0.11950372	0.285425253	-0.099880362	0.283419634
BnaA03g10180D	3.415037499	0.278780954	0	0.2263112	4.969626351	0.374933953	2.584962501	0.255299148	4	0.316335112
BnaA03g10580D	-2.079727192	0.551203811	-1.489310262	0.4721283	-1.015596855	0.446231375	-0.102447269	0.257350999	-1.104530583	0.491013245
BnaA03g11930D	-2.662965013	0.251618144	-2.662965013	0.2496566	1.366782331	0.254504386	1.452512205	0.273602892	-2.662965013	0.252892846
BnaA03g12400D	0	0.22451918	0	0.2263112	4.544320516	0.337333562	0	0.226982722	0	0.223935767
BnaA03g12550D	1.805905145	0.757808922	0.684446156	0.4896549	0.312377417	0.36818398	0.359358785	0.388361249	0.375988588	0.42847802
BnaA03g12580D	0	0.22451918	0	0.2263112	0	0.21244364	0	0.226982722	0	0.223935767
BnaA03g12690D	0	0.22451918	2.807354922	0.2544626	3.807354922	0.28742867	4.36923381	0.341758692	2.874469118	0.258500211
BnaA03g13950D	-5.459431619	0.434353975	-5.459431619	0.420013	-0.386182637	0.248591004	-5.459431619	0.439970147	1.189225557	0.394155747
BnaA03g16870D	-3.938599455	0.305333932	-3.938599455	0.3001427	0.646363045	0.248049421	0.705256734	0.266555708	1.733825887	0.358235144
BnaA03g17580D	1.31724148	0.429574395	-0.031535573	0.2285128	-0.531381461	0.272614393	-1.26459492	0.342212212	-1.361456459	0.357810243
BnaA03g21790D	1.554588852	0.302874793	2.483815777	0.3633516	1.50779464	0.289654444	1.988684687	0.339979834	2.918863237	0.451609778
BnaA03g21820D	-0.763036573	0.377857621	0.800552277	0.4072264	0.282899712	0.291585209	-0.247615005	0.2878954	-0.01003618	0.225774067
BnaA03g22590D	-1.321928095	0.295922276	0.969303203	0.3048408	-0.711874613	0.257535929	0.116644919	0.240922981	-0.212303604	0.244093223
BnaA03g24010D	-1.321422684	0.556659269	1.739793617	0.6825135	-0.235595636	0.304644845	-0.505789833	0.390435116	-0.713898029	0.475686005
BnaA03g26820D	0	0.22451918	0	0.2263112	7.930737338	0.739144123	6.453956489	0.554743915	6.36923381	0.560853235
BnaA03g27440D	-0.95793594	0.31083122	-2.152951923	0.350631	-2.593524514	0.372463806	-5.815916936	0.477660802	-5.815916936	0.489089084
BnaA03g29470D	2.494630181	0.611706999	1.694654789	0.4577015	1.123792486	0.388640847	0.417120814	0.282314453	0.417120814	0.284960724
BnaA03g30340D	0	0.22451918	2.502500341	0.2479504	3.36923381	0.262696379	3.415037499	0.283188471	4.345774837	0.340140547
BnaA03g30700D	0.667156128	0.369932016	0.572828745	0.3327059	0.007231569	0.213982528	0.09469441	0.248502941	0.108209217	0.252723326
BnaA03g30840D	0.110703464	0.247399961	2.337772373	0.6394598	0.759796303	0.372001479	0.217325884	0.272455881	-0.337500899	0.289396861
BnaA03g31720D	0	0.22451918	0	0.2263112	3.415037499	0.266500669	0	0.226982722	0	0.223935767
BnaA03g34140D	2.921997488	0.368822431	-0.247927513	0.2323787	2.990477226	0.36720869	1.395928676	0.269910881	1.243925583	0.264915548
BnaA03g34320D	-0.493525114	0.431828789	0.465174287	0.4171378	-0.168834117	0.301699162	-0.265218653	0.348273099	-0.051093848	0.254865441
BnaA03g37010D	0.862496476	0.251261492	2.373458396	0.3233713	-2.874469118	0.244608387	-2.874469118	0.260578481	0.862496476	0.252483356
BnaA03g37280D	0.017487427	0.22451918	2.62544157	0.4948021	0.257157839	0.234426186	-0.03562391	0.229441861	-1	0.283021153
BnaA03g39240D	0.037358251	0.235051428	-0.795670065	0.4025195	0.044715202	0.227555127	-0.009111788	0.22910062	0.361055439	0.359291891
BnaA03g39790D	-1.48112669	0.356691852	-3.48112669	0.4022971	-0.80556164	0.301520836	-3.403124178	0.424229015	-1.311201688	0.36194917
BnaA03g40190D	-4.087462841	0.316995579	-1.584962501	0.2652436	0.631355406	0.247928335	-1.502500341	0.27374159	-1.86507042	0.277803463
BnaA03g40400D	0	0.22451918	3.459431619	0.2775591	0	0.21244364	2.584962501	0.255299148	0	0.223935767
BnaA03g40600D	4.247927513	0.327631301	3.169925001	0.2654263	0	0.21244364	0	0.226982722	0	0.223935767
BnaA03g40690D	-2.147259182	0.734846684	1.805107855	0.7319979	-1.063133818	0.608025996	-3.59781265	0.806115485	-0.508268748	0.477097203
BnaA03g41680D	-0.228027956	0.306386276	1.027162331	0.5372437	0.161463423	0.279474444	0.217865605	0.308673694	-0.009522774	0.227755469
BnaA03g42350D	1.052727248	0.597139297	1.556739775	0.6727716	-0.072469638	0.246768114	0.118352704	0.279289514	0.485030809	0.454108546
BnaA03g42550D	-0.033255767	0.238642168	-0.418694954	0.4006305	-0.049516091	0.241744161	-0.020533277	0.235923245	-0.244046305	0.364533798
BnaA03g43060D	0.125223762	0.291974885	-1.00600066	0.5800222	-0.152412601	0.29814805	-0.156770812	0.308618655	-0.394910799	0.441737997
BnaA03g43540D	0.143900113	0.27377021	-1.072300475	0.453895	0.273525005	0.308642872	0.425649635	0.363162228	0.965055407	0.56367343
BnaA03g44700D	1.563429339	0.325117123	2.321928095	0.3834254	1.378511623	0.300849361	0.438884241	0.250350048	1.707819249	0.352293142
BnaA03g52170D	-1.317740298	0.33509678	-1.033947332	0.3059614	-0.159478214	0.235650252	-0.725825037	0.29912334	-0.360175564	0.269582849
BnaA03g53110D	2.680055381	0.780515781	0.777758797	0.4272848	0.494471967	0.369780108	0.787253716	0.455863186	0.106655936	0.264162616
BnaA03g53870D	5.129283017	0.40141428	5.014950341	0.3782649	0	0.21244364	0	0.226982722	5.333900737	0.433548205
BnaA03g55780D	-1.538948945	0.695289108	1.067125937	0.6151106	-1.124286805	0.639952975	-0.358884562	0.391819895	-0.490239524	0.482869685
BnaA03g58600D	-3.772589504	0.296939396	-3.772589504	0.2922413	-0.965234582	0.242503699	-3.772589504	0.301791627	-0.03562391	0.223935767
BnaA04g00800D	-0.966833136	0.254942495	-0.216811389	0.2361918	0.381090167	0.23177331	-1.033947332	0.258636708	-1.10433666	0.260739195
BnaA04g01380D	0.863678529	0.283613371	3.947313575	0.6358867	-0.843274496	0.248584399	0.713118852	0.274897407	0.259087221	0.244471891
BnaA04g10190D	3.058893689	0.262619324	0	0.2263112	0	0.21244364	2.938599455	0.264316725	2.938599455	0.262328719
BnaA04g13540D	-2.216811389	0.370187397	-0.150140349	0.2445027	-1.644905041	0.344409366	-5.841302254	0.480480996	0.195787065	0.254290834
BnaA04g18810D	-0.565597176	0.244172479	-3.624490865	0.2834218	-3.624490865	0.275137377	-3.624490865	0.291867008	-3.624490865	0.291074448
BnaA04g19630D	0.272272613	0.312638698	0.793608531	0.4430633	0.301393378	0.316484818	-0.330060339	0.322838511	0.027539024	0.235368453
BnaA04g21820D	-0.123735368	0.234252263	2.04858018	0.4052472	1.395137942	0.342135158	0.591535155	0.271145954	1.944601761	0.436449857
BnaA04g24320D	1.907832386	0.719466607	1.82178108	0.6788061	-0.361652743	0.333190232	0.599873109	0.425162475	0.175196818	0.298064391
BnaA04g28770D	4.564784619	0.351196766	0	0.2263112	0	0.21244364	0	0.226982722	0	0.223935767
BnaA04g29720D	0	0.22451918	0	0.2263112	5.156504486	0.395014354	0	0.226982722	0	0.223935767
BnaA05g00340D	-1.626185163	0.496997076	-0.269823571	0.2838666	-0.626185163	0.361610131	-0.847996907	0.401982282	-0.115100977	0.261714484
BnaA05g00510D	-2.662965013	0.251618144	-0.160464672	0.23213	-0.160464672	0.219805647	-0.160464672	0.234012293	1.337034987	0.26916015
BnaA05g00710D	0	0.22451918	7.569855608	0.6639874	4.662965013	0.34721415	4.841302254	0.380193473	4.345774837	0.340140547
BnaA05g01050D	1.310256322	0.708892529	-2.979046626	0.8166125	0.604208038	0.523299077	0.507591235	0.475333756	0.427283839	0.483237345
BnaA05g03970D	1.115205856	0.477735655	0.748209256	0.3698351	-0.262329104	0.268638381	0.147410207	0.263737715	-0.681603596	0.357162986
BnaA05g05710D	1.460680165	0.389911145	2.351843673	0.4914139	1.435501602	0.383414351	0.929610672	0.323347071	0.824913293	0.315051604
BnaA05g06600D	0.168367856	0.265107084	-0.1906455	0.2606952	-0.222976154	0.261969865	-0.268648012	0.281845521	-0.841010476	0.381091708
BnaA05g07470D	-1.123846081	0.464215893	1.08549806	0.5067302	0.210154846	0.281620962	0.244996595	0.300512522	0.346200366	0.344162792
BnaA05g07510D	-3.612030744	0.463920885	-1.942179346	0.3873507	-1.31657486	0.370055303	-0.270562463	0.270386417	-0.427606173	0.293810983
BnaA05g12950D	0.45801591	0.450035665	-0.330954879	0.3781108	-0.772543904	0.571271003	0.628481798	0.518851932	0.917423386	0.66015226
BnaA05g19710D	0.588160673	0.39448598	1.466226524	0.5819353	0.461351373	0.356192099	0.462745955	0.359525256	0.398615617	0.359996389
BnaA05g22120D	1.048628111	0.498916834	2.105794664	0.670231	-0.193334538	0.265296418	-0.375378734	0.315262954	0.223081552	0.292994206
BnaA05g23050D	0.070959425	0.256963525	0.313655381	0.3520598	-0.007113457	0.216653017	-0.208123488	0.320832893	-0.116327503	0.291591814
BnaA05g26450D	0	0.22451918	0	0.2263112	1.874469118	0.229217302	1.874469118	0.244405844	2.736965594	0.256833633
BnaA05g27610D	0.368789867	0.400062524	0.148956777	0.2956933	-0.178351169	0.310974321	-0.286510731	0.362887034	-0.503051733	0.486279897
BnaA05g27620D	0.700439718	0.37727641	0.718087584	0.3620681	-0.349235441	0.282911075	0.042644337	0.23380975	-0.184424571	0.268010937
BnaA05g27740D	-0.579441273	0.427410265	-2.394531844	0.6619311	0.383156529	0.377648473	-0.864912377	0.500517366	-0.472211208	0.425114041
BnaA05g28870D	-1.221362587	0.656713428	-3.770736331	0.822636	0.206052478	0.330253355	0.078197695	0.269041266	0.033400982	0.244544542
BnaA05g29060D	0.40082364	0.317394061	1.463711346	0.5297739	0.182789219	0.260072123	-0.034995421	0.232513033	-0.202105407	0.275494029
BnaA05g29680D	0	0.22451918	3.415037499	0.2749172	0	0.21244364	3.415037499	0.283188471	2.502500341	0.251349554
BnaA05g29910D	0.227920553	0.327789813	0.661460638	0.4723947	-0.410283969	0.392671898	0.121742324	0.283192874	-0.270194197	0.361176424
BnaA05g30870D	-4.906890596	0.380200077	-4.906890596	0.370216	-4.906890596	0.370240234	-4.906890596	0.386003382	-2.321928095	0.324701029
BnaA05g32140D	-0.661677467	0.360361848	1.116269689	0.4824756	-0.890878278	0.393376396	-0.446288433	0.324738455	-0.83926417	0.41025133
BnaA05g34070D	0	0.22451918	-2.584962501	0.2496566	2.748938236	0.338535612	1.415037499	0.269946106	2.748938236	0.358627021
BnaA05g36000D	-0.294620749	0.32618708	-0.04142984	0.2382305	-0.303413867	0.325143542	-0.438022308	0.369614992	-0.46454575	0.401687273
BnaA05g37010D	0	0.22451918	0	0.2263112	0	0.21244364	0	0.226982722	0	0.223935767
BnaA05g37380D	0.125123629	0.271760189	-1.10513091	0.4781672	0.438964343	0.375951073	0.265881965	0.320892335	0.093414769	0.264107577
BnaA06g03350D	1.015540851	0.414299993	-3.721424743	0.4484131	0.256945241	0.261274173	0.558683176	0.327499207	0.661044894	0.360515957
BnaA06g05130D	0	0.22451918	0	0.2263112	0	0.21244364	0	0.226982722	0	0.223935767
BnaA06g05440D	0	0.22451918	3.584962501	0.2834218	0	0.21244364	0	0.226982722	0	0.223935767
BnaA06g05610D	-1.1379421	0.4499432	1.673542678	0.6093623	0.080999722	0.238820494	0.338495944	0.320302318	0.298052119	0.320198845
BnaA06g11740D	-0.828519532	0.312213798	1.419407981	0.4318574	-0.567559454	0.280454137	-1.853610513	0.381045475	-0.328948523	0.271055691
BnaA06g11800D	-0.034449159	0.235375057	0.390309066	0.3610818	-0.290858088	0.330220332	-0.238284435	0.317825566	-0.467865188	0.418815386
BnaA06g12480D	-1.384667659	0.71173694	-0.79559364	0.5529518	0.122448132	0.288410564	0.266310108	0.368952323	-0.300188	0.413538254
BnaA06g12690D	0	0.22451918	5.235216462	0.3991203	0	0.21244364	0	0.226982722	5.297680549	0.429594209

BnaA06g12860D	-2.00487672	0.654254289	-0.643754767	0.4166535	-0.157603695	0.282186762	-0.218610781	0.311557734	-0.290228055	0.352607964
BnaA06g12870D	-1.553801569	0.372300891	-1.26934818	0.3404972	-0.166778446	0.240046761	-1.932313192	0.394190972	0.188702208	0.260146976
BnaA06g15430D	-0.176909236	0.298856952	0.294302575	0.3313299	-0.410941327	0.377388689	-0.15820963	0.293403695	-0.340506169	0.376988006
BnaA06g15640D	-4.584962501	0.352933795	-4.584962501	0.3447176	-1.921997488	0.284478583	-4.584962501	0.358510339	-4.584962501	0.361596921
BnaA06g18160D	-2.061448101	0.624284494	-2.047816235	0.5920581	-3.650983332	0.703238931	-1.977211564	0.62328939	-1.187809311	0.571187344
BnaA06g21780D	4.029747343	0.311179066	0	0.2263112	0	0.21244364	3.058893689	0.26634656	0	0.223935767
BnaA06g21900D	-0.76916251	0.357189404	0.655665492	0.356016	-0.826419828	0.362338846	0.202492864	0.275689968	0.392631086	0.326539329
BnaA06g24760D	2.502500341	0.250160714	0	0.2263112	0	0.21244364	0	0.226982722	5.357552005	0.437530822
BnaA06g24950D	-0.308586558	0.324910176	0.036099341	0.2369998	0.135951881	0.267984519	0.010924962	0.229701645	-0.35614381	0.356894396
BnaA06g28210D	0	0.22451918	0	0.2263112	4.584962501	0.342394942	2.415037499	0.249922946	3.874469118	0.307773257
BnaA06g28660D	1.457045026	0.331583096	1.811097324	0.351232	1.19592021	0.299988552	-0.652076697	0.257188083	-1.459431619	0.277114375
BnaA06g29540D	-1.185172614	0.625843196	-3.820093274	0.7976593	-0.036798355	0.234252263	-2.230297619	0.749619131	-0.177787119	0.327485998
BnaA06g30710D	-3	0.262619324	-3	0.2596164	-3	0.249962574	-3	0.26634656	-3	0.264244073
BnaA06g31220D	-1.164854012	0.480379725	-0.824816536	0.3974185	-1.740356183	0.551181796	-3.09085343	0.623247561	-2.839891857	0.644397918
BnaA06g31230D	-0.219876823	0.255605164	1.332464256	0.4106124	1.67286959	0.499341734	-0.933572638	0.319265384	-0.682611065	0.305289901
BnaA06g31470D	0.9274875	0.279580119	1.949626477	0.3580128	0.897430266	0.267478161	0.393663848	0.250224559	1.13492958	0.305100567
BnaA06g31780D	-0.99034983	0.306344447	-0.664579669	0.275679	1.272684576	0.396462979	-1.464281018	0.334751136	0.705643983	0.321863222
BnaA06g37380D	-0.468021955	0.307051147	-4.318126525	0.5123639	0.212023091	0.261254359	-0.791266979	0.351571031	0.53985447	0.354364807
BnaA06g37640D	0.321538651	0.31848163	0.458192114	0.3406711	-0.059623983	0.232365529	-0.321441438	0.311434446	0.121306296	0.267790782
BnaA06g38670D	-3.841302254	0.299596675	-0.966833136	0.2509621	0.033166864	0.21244364	-3.841302254	0.304259572	-0.902702799	0.25601025
BnaA07g00280D	3.459431619	0.281262109	3.36923381	0.2716061	0	0.21244364	0	0.226982722	2.502500341	0.251349554
BnaA07g05740D	0	0.22451918	0	0.2263112	0	0.21244364	3.874469118	0.307674187	0	0.223935767
BnaA07g06740D	0	0.22451918	2.596367264	0.3349625	-2.807354922	0.244608387	0.893084796	0.254513192	-0.070389328	0.223935767
BnaA07g10350D	-1.142550401	0.634235531	1.497672883	0.6967311	0.323736749	0.382705449	0.740824572	0.545735144	0.779949435	0.606867977
BnaA07g11710D	-1.922946741	0.727528708	-0.20805024	0.316738	-0.152988112	0.29507908	0.074153369	0.262370548	-0.205229031	0.344741801
BnaA07g11930D	-0.777382842	0.518620769	1.532631037	0.6901485	-0.038261558	0.233842774	-0.966564989	0.571883036	0.640264651	0.543086671
BnaA07g22130D	-0.325345455	0.279734228	0.022577848	0.2292591	-0.143432103	0.242182271	0.388628496	0.301954542	1.009073584	0.456682166
BnaA07g22240D	-2.944260355	0.682066557	-4.176921112	0.7057641	-0.498849207	0.388240163	-0.575521721	0.410416447	-1.301632513	0.597233964
BnaA07g22980D	-1.715581204	0.595919634	-0.368686193	0.3316668	-0.621572644	0.416384867	-0.439712145	0.366264222	0.007647777	0.226885854
BnaA07g24010D	0.471480253	0.413738596	2.622295009	0.7941742	0.020482848	0.22187511	-0.2304	0.320033728	-0.721301957	0.512161401
BnaA07g24240D	3.584962501	0.287446282	0	0.2263112	0	0.21244364	0	0.226982722	0	0.223935767
BnaA07g25720D	-1.6199498	0.5233321	-1.127351318	0.4409763	0.333163812	0.319333633	0.52616988	0.38246548	0.772900081	0.48502501
BnaA07g25800D	0.267695783	0.266005319	-6.101538026	0.4876537	1.127280664	0.407798795	0.551904212	0.310045264	0.833135726	0.369370619
BnaA07g26980D	2.807354922	0.256930501	4.222392421	0.3190364	2.736965594	0.243100321	3.700439718	0.298240516	4.662965013	0.366724347
BnaA07g28100D	-2.871013559	0.363948184	-5.533978572	0.4262853	-2.074546953	0.337688013	-2.949016071	0.368294058	-5.533978572	0.455887404
BnaA07g29070D	0.05196999	0.246616207	1.735014688	0.7169656	-0.422483684	0.400896914	-0.517626778	0.432980204	-1.064552388	0.627289619
BnaA07g30860D	4.029747343	0.311179066	3.969626351	0.3032513	4.087462841	0.305193033	4.087462841	0.321827997	4.029747343	0.316335112
BnaA07g31540D	-0.707266598	0.374280091	-1.883976204	0.4806263	0.237541616	0.281510884	-0.58852032	0.355452376	0.436202109	0.359441597
BnaA07g31550D	-0.982297998	0.31107119	-3.017921908	0.3658283	-2.97453758	0.374601518	-0.44503224	0.27320441	-2.054447784	0.374438603
BnaA07g31680D	0.798838855	0.423581757	-3.106499621	0.5192064	0.740203968	0.411026278	-0.080556354	0.247945947	1.625804595	0.651966871
BnaA07g31770D	-2.404773636	0.78102434	-3.354454683	0.8058843	-0.56253971	0.483853781	-0.720869286	0.529487654	-0.827997717	0.608631424
BnaA07g32000D	0.44220577	0.400868294	-1.801158656	0.6219024	0.497010859	0.427839568	0.101879614	0.270588961	-0.89426806	0.552165892
BnaA07g34700D	0.988318195	0.293982705	-4.36923381	0.3291019	0.349584438	0.237787964	-0.632268215	0.257128641	0.916168409	0.292542886
BnaA07g36940D	0	0.22451918	0	0.2263112	5.523561956	0.432966994	0	0.226982722	4.662965013	0.366724347
BnaA07g36950D	-1.415037499	0.512049121	-5.667424661	0.6829604	-0.215728691	0.282272623	-0.194554668	0.286074712	-1.223506042	0.525641534
BnaA07g38030D	1.500428991	0.313911198	1.822357085	0.3287673	-0.965234582	0.242503699	0.549338591	0.253802089	0.946228744	0.277532671
BnaA07g38930D	0.2694192	0.356539945	-0.012586004	0.2314761	-0.305952857	0.371655835	-0.042204477	0.245066311	-0.336600037	0.413097943
BnaA08g09740D	0	0.22451918	2.471305719	0.3652933	0.447458977	0.231883388	0.862496476	0.263957871	2.932885804	0.457686076
BnaA08g11190D	0.283540061	0.311027158	0.48416186	0.3516019	0.045305741	0.229316373	-0.645516687	0.386335817	-0.044947406	0.239370883
BnaA08g12050D	-0.747557005	0.332996495	0.710493383	0.3454903	-0.306185358	0.26698281	-0.34586906	0.286171581	0.057626818	0.23802353
BnaA08g15280D	-5.672425342	0.457087252	-5.672425342	0.440952	-5.672425342	0.449846331	-5.672425342	0.462498679	-5.672425342	0.472936261
BnaA08g15610D	-0.916904384	0.335565712	1.673431289	0.5007771	0.959520931	0.392167741	0.236174946	0.269074289	1.176877762	0.468491317
BnaA08g16990D	-0.345759801	0.35630658	-2.230353137	0.6527947	0.551622193	0.445434411	0.069736206	0.256602469	1.055222744	0.645580154
BnaA08g19860D	-0.84434913	0.269728152	-2.303780748	0.2958871	-0.84434913	0.258007063	-2.303780748	0.309182254	-4.718818247	0.37162061
BnaA08g21210D	0.242964031	0.289029201	0.749127479	0.3943517	-0.028263852	0.220510145	-0.446765207	0.324738455	0.017702002	0.228713146
BnaA08g22290D	-0.933661107	0.62286449	0.015208723	0.2368193	0.203736662	0.335114393	0.434869437	0.444353447	0.313634689	0.42381292
BnaA08g22300D	0	0.22451918	4.029747343	0.3056399	0	0.21244364	0	0.226982722	0	0.223935767
BnaA08g22480D	-0.766014127	0.438864965	-3.533179959	0.6551173	0.597687567	0.433057258	0.729998941	0.470516749	-0.027826427	0.236746627
BnaA08g22580D	0.497177017	0.351947497	-0.902702799	0.3744188	0.106915204	0.244302371	-0.290654118	0.295327856	1.352065147	0.601954102
BnaA08g22850D	0	0.22451918	0	0.2263112	0	0.21244364	0	0.226982722	0	0.223935767
BnaA08g23070D	0.091788341	0.263883018	0.677694582	0.4709571	-0.50084129	0.417538483	-0.139315576	0.288668146	-0.278082842	0.359514248
BnaA08g26320D	-2.502500341	0.250160714	-2.502500341	0.2479504	1.404390255	0.250803568	-2.502500341	0.253714027	-2.502500341	0.251349554
BnaA08g26750D	-0.459168157	0.426459192	0.713651747	0.5072651	-0.026745481	0.229105023	0.305270362	0.372560675	-0.07034416	0.268504086
BnaA08g29620D	0.900464326	0.325242612	1.674309986	0.4132432	0.568842835	0.273772412	-2.206450877	0.338819613	0.485426827	0.27992136
BnaA08g29670D	-2.941897045	0.455548364	-2.517399217	0.4177344	-1.746881063	0.406816901	0.253118937	0.275247455	-0.441631709	0.300171281
BnaA08g30200D	-1.247408054	0.623850787	0.427453196	0.3986315	-0.11727946	0.275718588	0.291432401	0.358239547	-0.168211494	0.31865115
BnaA09g00080D	-0.247521406	0.316117158	-0.765720433	0.4352522	0.983661263	0.571202755	0.768916618	0.4988684	0.018449037	0.23310305
BnaA09g02150D	0	0.22451918	0	0.2263112	0	0.21244364	0	0.226982722	0	0.223935767
BnaA09g02730D	0	0.22451918	0	0.2263112	0	0.21244364	2.938599455	0.264316725	0	0.223935767
BnaA09g03290D	-4.058893689	0.314587076	-1.556393349	0.2647857	-4.058893689	0.30296946	-4.058893689	0.319509757	0.799087306	0.27253954
BnaA09g05480D	2.736965594	0.255312357	3.502500341	0.2775591	2.662965013	0.239348868	2.736965594	0.258914104	2.662965013	0.252892846
BnaA09g06090D	1.865834762	0.567612015	-0.022454851	0.2280659	1.168067055	0.442006587	0.545434137	0.32170471	2.171780558	0.654654972
BnaA09g07190D	0	0.22451918	0	0.2263112	2.662965013	0.239348868	0	0.226982722	0	0.223935767
BnaA09g07290D	-1.374848409	0.364621861	0.584962501	0.3067121	0.496735318	0.296193068	0.671945802	0.337595548	0.341358625	0.285830339
BnaA09g10270D	-0.153912683	0.246853975	-1.088536675	0.31001	-0.061400545	0.220875603	0.222392421	0.258436366	-0.792404581	0.307161224
BnaA09g11280D	0	0.22451918	0	0.2263112	0	0.21244364	0	0.226982722	0	0.223935767
BnaA09g14920D	0.685696924	0.427368435	0.786127015	0.4306377	-0.159618393	0.264233066	-0.043832081	0.237039434	-0.157610466	0.279776058
BnaA09g18280D	-2.662965013	0.251618144	-2.662965013	0.2496566	-2.662965013	0.239348868	-2.662965013	0.255299148	-2.662965013	0.252892846
BnaA09g28450D	0	0.22451918	0	0.2263112	0	0.21244364	3.772589504	0.301791627	5.285402219	0.429594209
BnaA09g30490D	-0.847592564	0.469730794	1.68204723	0.6734981	0.058448781	0.238562912	0.922643259	0.539062225	1.582939085	0.725800486
BnaA09g32720D	-0.041500987	0.238785269	0.878107695	0.5104992	0.146733656	0.279983004	0.26052755	0.329174592	0.059694964	0.254466959
BnaA09g35310D	-0.929990279	0.31426785	2.081354695	0.5241599	-0.42303029	0.26094394	-1.412383046	0.348123393	-0.159995884	0.248639438
BnaA09g37190D	-0.187085553	0.248370848	0.654732779	0.2933421	0.654732779	0.295627268	-0.039528364	0.229441861	0.434402824	0.280830603
BnaA09g39030D	-1.777976319	0.492649001	-1.262518328	0.4197951	-0.756017066	0.373157297	-1.371816971	0.458956374	-0.549275645	0.357997376
BnaA09g40030D	-2.321928095	0.246506129	-2.321928095	0.244965	-2.321928095	0.234239054	-2.321928095	0.249922946	-2.321928095	0.247556272
BnaA09g44370D	-0.25667611	0.352654197	-0.395906317	0.3969121	0.338989613	0.394263623	0.505346337	0.463341875	0.268969686	0.386395259
BnaA09g44500D	0	0.22451918	0	0.2263112	3.502500341	0.268900366	3.459431619	0.285733471	3.502500341	0.284370707
BnaA09g44780D	0	0.22451918	0	0.2263112	0	0.21244364	0	0.226982722	3	0.264244073
BnaA09g44790D	0	0.22451918	3.736965594	0.2890842	0	0.21244364	0	0.226982722	0	0.223935767
BnaA09g44970D	-0.277907682	0.284795607	1.167182234	0.474768	-0.031160931	0.219281676	0.786060144	0.411710962	-0.006178575	0.225774067
BnaA09g52130D	1.013056153	0.334387879	-0.736965594	0.2701222	1.413397141	0.384583377	0.051530301	0.230181584	1.567368441	0.433449135
BnaA09g55480D	0.519928615	0.310109109	2.175214448	0.5772791	2.469154048	0.658578146	0.103504576	0.245775212	-0.111508315	0.24608343
BnaA09g56310D	-2.807354922	0.256930501	-2.807354922	0.2544626	-2.807354922	0.244608387	-2.807354922	0.260578481	0.893084796	0.252635264
BnaA10g00430D	1.485316491	0.637225246	-2.259948426	0.5608092	0.8374895	0.488281112	0.92062382	0.50712644	1.285639836	0.640798373
BnaA10g00780D	0.691732616	0.547919088	-5.173428841	0.9110791	0.566940748	0.515010215	0.395065461	0.430452816	0.537061962	0.538698968
BnaA10g04120D	-0.000950079	0.22451918	-4.614709844	0.7269365	-0.325732171	0.336761157	-0.62199988	0.423200888	-0.435763942	0.398479605
BnaA10g10930D	2.770518154	0.419460442	-3.502500341	0.2775591	-3.502500341	0.268900366	-3.502500341	0.285733471	-3.502500341	0.284370707
BnaA10g11980D	-0.323019255	0.320852707	2.028084294	0.7002338	0.035907653	0.226168146	0.15693936	0.282314453	-0.252023625	0.314798425
BnaA10g12770D	-0.918247018	0.57262496	-3.046571115	0.7752342	0.20387683	0.321660678	-0.606881717	0.481350611	-0.504580511	0.482911515
BnaA10g16590D	-0.192176746	0.313523724	-1.920272808	0.6794489	-0.042423032	0.234133379	0.255500733	0.345384656	0.215466814	0.346914738
BnaA10g16980D	2.055853235	0.301925922	0.796466606	0.2451588	1.337034987	0.254504386	-2.662965013	0.255299148	-0.078002512	0.223935767
BnaA10g17370D	0.321928095	0.390769752	-3.711944864	0.8484933	0.339879345	0.404743034	0.198944581	0.334577213	0.404922099	0.467755997
BnaA10g17700D	2.584962501	0.251618144	2.415037499	0.244965	0	0.21244364	0	0.226982722	2.502500341	0.251349554

BnaA10g17890D	-0.369885463	0.347947268	-2.646725668	0.6354772	0.771775686	0.499051129	0.297539198	0.336323048	0.098701505	0.270307161
BnaA10g18810D	-0.960829403	0.531083782	-1.80934165	0.6270607	-0.710734053	0.477667406	-0.547080599	0.425481701	0.244160866	0.345221741
BnaA10g19100D	-1.569855608	0.302874793	-4.984893108	0.3767987	-1.662965013	0.297392916	-4.984893108	0.393279527	-4.984893108	0.39871297
BnaA10g20990D	-0.176111146	0.288377541	-0.496047943	0.3597608	-0.125210248	0.263103667	-0.246899063	0.310549421	-0.069860319	0.255211085
BnaA10g21970D	3.459431619	0.281262109	3.415037499	0.2749172	3.459431619	0.268900366	4.624490865	0.36175103	3.459431619	0.284370707
BnaA10g22930D	-1.79930265	0.666398077	0.48294672	0.4044018	-0.392093141	0.378938585	-0.022302394	0.234338124	-0.345749915	0.388957871
BnaA10g23980D	4.160991877	0.506294251	0.784271309	0.2424156	2.096861539	0.292179629	1.502500341	0.27374159	2.353636955	0.32804079
BnaA10g24770D	-0.500867409	0.398307883	-2.468857014	0.6545119	-0.306489364	0.336833809	-0.402565335	0.368998556	0.015438731	0.232605499
BnaA10g25530D	0.95247163	0.325742365	2.760812336	0.5519876	-0.013420516	0.21244364	0.993305347	0.333335535	-1	0.29657834
BnaA10g26900D	-0.070289698	0.25906381	-1.922417059	0.7033314	0.249784148	0.345831572	0.310037056	0.373830973	-0.04804604	0.255790095
BnaA10g27190D	-1.744161096	0.287893198	1.010726407	0.2933509	1.46529227	0.340565448	0.840801405	0.288674751	0.634350528	0.271533428
BnaA10g28420D	2.473120124	0.791470728	0.710218683	0.4524706	0.198319645	0.295876044	0.179233804	0.296783085	0.438332988	0.414590598
BnaA10g29180D	0.057333175	0.232576878	-0.247521406	0.2565562	-1.351472371	0.352555127	-1.750021747	0.384479904	-1	0.347936261
BnaA10g30020D	0	0.22451918	4.196397213	0.316126	0	0.21244364	3.700439718	0.298240516	0	0.223935767
BnaAnng01040D	0.707311525	0.460065959	2.483649014	0.7596891	-0.532450664	0.382474286	0.014847764	0.230141956	0.4439724	0.401561784
BnaAnng02150D	2.502500341	0.250160714	0	0.2263112	4.604862058	0.342394942	0	0.226982722	2.415037499	0.247556272
BnaAnng03830D	-1.720732567	0.672604266	1.228791482	0.6269814	-0.269579107	0.337936789	-4.326015052	0.807396791	-0.556423357	0.478787999
BnaAnng05010D	0	0.22451918	0	0.2263112	0	0.21244364	0	0.226982722	0	0.223935767
BnaAnng06040D	-0.032061209	0.2266679	2.469485283	0.4876471	2.077167861	0.461882243	1.286621226	0.359212635	-1.922832139	0.315417063
BnaAnng06630D	-2.362285833	0.564292067	-2.887127341	0.5565228	0.098075657	0.245577072	0.060405239	0.243833439	-0.326911502	0.322873736
BnaAnng06640D	-1.776288724	0.536215612	-4.361251225	0.623023	-0.699285654	0.391993818	-0.436751229	0.339312762	-0.333054333	0.327728169
BnaAnng13080D	0.348840757	0.328793723	1.31751618	0.5572625	0.25966541	0.29714414	0.512185061	0.377003417	0.106128351	0.264162616
BnaAnng13560D	0	0.22451918	2.415037499	0.244965	0	0.21244364	0	0.226982722	0	0.223935767
BnaAnng13960D	-0.507656775	0.343165487	-1.513943037	0.4596058	0.350756748	0.316004879	-0.163322267	0.272499912	0.226169112	0.298321973
BnaAnng15540D	-0.657731457	0.508848057	0.136895321	0.2931241	0.075337726	0.260565272	0.014144182	0.232303885	-0.281123544	0.388706894
BnaAnng17460D	0.932885804	0.254942495	-2.874469118	0.2544626	-2.874469118	0.244608387	-2.874469118	0.260578481	-2.874469118	0.258500211
BnaAnng17890D	0.582227533	0.431826588	-5.874469118	0.7591805	0.73470962	0.488248089	0.560853882	0.426197207	0.71736486	0.516775864
BnaAnng21440D	0.487706567	0.309809697	1.621754532	0.4995399	0.441412915	0.292961182	0.536236461	0.322521487	0.836714196	0.395950016
BnaAnng26800D	-0.078002512	0.22451918	-2.662965013	0.2496566	0.796466606	0.235542376	2.055853235	0.305875515	-0.160464672	0.231936225
BnaAnng27960D	0	0.22451918	0	0.2263112	0	0.21244364	3.459431619	0.285733471	0	0.223935767
BnaAnng30810D	-2.807354922	0.256930501	-2.807354922	0.2544626	-2.807354922	0.244608387	0.893084796	0.254513192	-2.807354922	0.258500211
BnaAnng32120D	0	0.22451918	0	0.2263112	0	0.21244364	0	0.226982722	0	0.223935767
BnaAnng32620D	-1.60E-16	0.22451918	-1.463885759	0.4787286	0.580805731	0.39978733	0.40656284	0.347916447	0.827292773	0.503980415
BnaAnng34960D	0.652995708	0.37164923	0.717964632	0.3667948	0.206900245	0.26377294	-0.327839057	0.292945771	-0.323614472	0.29772315
BnaAnng35540D	1.456413427	0.604571753	0.837827274	0.431558	-0.157541277	0.260858079	-0.616395009	0.369551147	-0.078808841	0.251516873
BnaAnng35730D	0	0.22451918	0	0.2263112	0	0.21244364	0	0.226982722	0	0.223935767
BnaAnng36570D	-2.045214714	0.742488288	-3.278641186	0.7853548	0.214872407	0.326695639	0.37107356	0.399214925	-0.46457696	0.466820335
BnaAnng39140D	0.511448576	0.416701892	-1.115477217	0.5128989	0.512056667	0.420684508	-0.028014376	0.236328331	0.038496964	0.243578058
BnaAnng41910D	-1.74723393	0.406623164	-6.554588852	0.5393594	0.705938699	0.349171334	-6.554588852	0.566821656	0.797086587	0.390756543
BnaC01g00270D	1.554075455	0.608085438	0.663218941	0.3779765	-0.835984389	0.391892546	0.174742018	0.276251365	-0.235316987	0.291941861
BnaC01g01250D	3.662965013	0.290878509	0	0.2263112	0	0.21244364	0	0.226982722	0	0.223935767
BnaC01g01660D	-1.501176873	0.726185759	0.179001803	0.3162779	-0.635042804	0.533575945	-0.587425655	0.502855419	0.433122509	0.48281905
BnaC01g01940D	-0.695070909	0.455310596	1.490391928	0.6541244	0.357780974	0.36684103	1.100109299	0.607233435	0.955508081	0.609683768
BnaC01g02200D	-5.68182404	0.457087252	-5.68182404	0.440952	-0.289506617	0.244749287	-5.68182404	0.462498679	-5.68182404	0.472936261
BnaC01g03510D	2.548327499	0.768387404	0.426391997	0.3363847	0.352605693	0.325266829	0.356884574	0.33307355	0.809177658	0.495988763
BnaC01g03950D	-0.728279866	0.441520043	0.758136294	0.4627078	-0.130099991	0.266192451	0.05638247	0.248282786	-0.170709288	0.297485382
BnaC01g05660D	0.026472211	0.22451918	0.637429921	0.2578816	1.013296823	0.280134911	0.704544116	0.269479376	0.584962501	0.261701275
BnaC01g12000D	0	0.22451918	0	0.2263112	0	0.21244364	0	0.226982722	0	0.223935767
BnaC01g14080D	0	0.22451918	2.807354922	0.2544626	2.807354922	0.244608387	0	0.226982722	0	0.223935767
BnaC01g20650D	0	0.22451918	0	0.2263112	0	0.21244364	0	0.226982722	2.736965594	0.256833633
BnaC01g21010D	-0.137453163	0.299737574	0.234666686	0.3346499	0.073790726	0.256186375	0.201765234	0.33082576	-0.141150961	0.314395541
BnaC01g22100D	-0.023348746	0.231738085	0.248517227	0.3160093	-0.034018488	0.226648086	0.104458279	0.270025362	0.447907181	0.433616454
BnaC01g26310D	-2.415037499	0.246506129	-2.415037499	0.244965	2.820178962	0.33426239	-2.415037499	0.249922946	-0.299560282	0.232237839
BnaC01g26440D	-2.736965594	0.255312357	1.321928095	0.261371	-2.736965594	0.243100321	-2.736965594	0.258914104	0.807354922	0.248835376
BnaC01g27820D	-1.051024003	0.35042402	0.043887644	0.2318658	0.771094272	0.355117739	0.197510833	0.264539082	0.277812461	0.277255275
BnaC01g31020D	0	0.22451918	0	0.2263112	0	0.21244364	2.874469118	0.260578481	0	0.223935767
BnaC01g37570D	0	0.22451918	0	0.2263112	0	0.21244364	0	0.226982722	0	0.223935767
BnaC01g38230D	0	0.22451918	0	0.2263112	0	0.21244364	0	0.226982722	0	0.223935767
BnaC01g41180D	-0.085081501	0.237341048	2.395038634	0.6025749	0.127647823	0.236363556	0.624662625	0.326176072	1.578592976	0.534846243
BnaC02g00420D	-2.662965013	0.251618144	-2.662965013	0.2496566	-0.160464672	0.219805647	0.796466606	0.250946669	-0.160464672	0.231936225
BnaC02g01090D	-1.137545041	0.641331149	0.866673202	0.5637989	-0.516947491	0.467852866	-0.030417816	0.240727042	-0.531576082	0.506604671
BnaC02g02590D	0.547487795	0.414934041	2.80359087	0.7893374	-1.693022247	0.596820071	0.211400093	0.303856687	-0.001144542	0.223935767
BnaC02g03720D	-0.723290657	0.33627021	-0.49120918	0.2955062	-0.004127886	0.21244364	0.678071905	0.365401212	0.185413509	0.268737451
BnaC02g04390D	-3.736965594	0.29336847	0.485426827	0.2447581	-3.736965594	0.28129293	-3.736965594	0.298240516	-0.036525876	0.223935767
BnaC02g05740D	1.38827059	0.292967787	-3.502500341	0.2775591	-3.502500341	0.268900366	-3.502500341	0.285733471	1.338801913	0.295627268
BnaC02g05990D	-1.069839625	0.508784212	-2.346324749	0.6119998	-0.501126476	0.382163866	-0.627931261	0.415858695	-0.487187285	0.404809081
BnaC02g06730D	-4.034646143	0.4901018	-3.117108303	0.4369056	-0.59724083	0.303169802	-0.712718048	0.326429251	-0.483471956	0.305631142
BnaC02g07210D	1.442564333	0.687257829	-2.172370248	0.6662198	-0.507088583	0.419971204	0.664186084	0.486559495	0.317278578	0.384642819
BnaC02g07480D	0.074000581	0.22451918	-2.662965013	0.2496566	1.839535328	0.277312515	-2.662965013	0.255299148	-2.662965013	0.252892846
BnaC02g10950D	-2	0.241636285	-2	0.2407733	-2	0.229679629	-2	0.245020078	-2	0.242580753
BnaC02g10960D	0	0.22451918	2	0.2407733	0	0.21244364	2.502500341	0.253714027	0	0.223935767
BnaC02g16640D	-2.119446556	0.773831854	1.39479932	0.6865974	-0.155195347	0.306034027	-1.218908414	0.670250801	0.546561201	0.530687502
BnaC02g22160D	0	0.22451918	0	0.2263112	0	0.21244364	3.169925001	0.272455881	0	0.223935767
BnaC02g22630D	0.078002512	0.22451918	-2.584962501	0.2496566	0.91753784	0.235687678	0	0.226982722	-2.584962501	0.252892846
BnaC02g22880D	-3.058893689	0.262619324	-3.058893689	0.2596164	-3.058893689	0.249962574	-3.058893689	0.26634656	-3.058893689	0.264244073
BnaC02g27500D	0	0.22451918	0	0.2263112	0	0.21244364	0	0.226982722	0	0.223935767
BnaC02g27760D	6.279223644	0.528250379	5.247927513	0.4002409	0	0.21244364	0	0.226982722	0	0.223935767
BnaC02g37590D	-4.064955442	0.502384286	-2.698173111	0.43501	-0.188761643	0.249870108	-0.626382428	0.321002413	-0.612443237	0.329841664
BnaC02g39260D	-2.452512205	0.327622495	-5.115477217	0.3882864	-1.028014376	0.280134911	-5.115477217	0.405830603	-5.115477217	0.411893691
BnaC02g40390D	0	0.22451918	0	0.2263112	4.415037499	0.327201997	1.874469118	0.244405844	1.874469118	0.242076597
BnaC02g41860D	-5.309855263	0.419385589	-0.765534746	0.2725572	1.597035333	0.422866251	-0.049327712	0.230181584	-1.64689025	0.331125172
BnaC02g43910D	-3.321928095	0.275018493	-3.321928095	0.2716061	-0.0489096	0.21244364	1.242856524	0.283357991	0.900464326	0.262859294
BnaC02g44890D	0	0.22451918	2.165878168	0.4806329	1.367277082	0.387399169	-0.087462841	0.236273292	-0.502500341	0.269523407
BnaC02g46060D	0	0.22451918	0	0.2263112	0	0.21244364	0	0.226982722	0	0.223935767
BnaC02g46900D	1.037521119	0.523248441	1.546439429	0.6036845	0.259409542	0.29714414	0.744188113	0.443710592	0.694719436	0.461710522
BnaC03g00040D	-0.014160894	0.230111135	-5.277854275	0.8940391	0.626587568	0.530150322	0.806926145	0.579661136	0.859721151	0.642700518
BnaC03g00940D	-0.22702562	0.341296365	-3.229363001	0.806263	0.169098375	0.311379408	-0.018806517	0.236286502	-0.075022526	0.2771496
BnaC03g01040D	-0.772959853	0.36556853	2.311223796	0.6780509	-1.045134913	0.406422822	-0.361723924	0.303332717	0.017665874	0.228713146
BnaC03g01730D	-0.637248803	0.476496178	2.799122438	0.8132948	-0.357513798	0.381624485	0.021439575	0.23520994	-0.129537753	0.298511307
BnaC03g03210D	1.229481846	0.345098454	-4.857980995	0.3661916	1.016488123	0.311758075	-0.770518154	0.272541742	-0.121015401	0.236000299
BnaC03g03620D	-1.914474292	0.686951812	1.001326372	0.572145	-0.444711123	0.402873912	-0.094313037	0.268035154	-0.511997337	0.458588714
BnaC03g04220D	-3.584962501	0.287446282	-3.584962501	0.2834218	0.852442812	0.2518383	-1	0.255034961	1.949016071	0.353484184
BnaC03g04820D	-1.438884241	0.31602029	-1.175849835	0.2961446	-1.175849835	0.29718597	-1.123382416	0.30647654	-0.780990218	0.287879989
BnaC03g06900D	-1.851242566	0.491440347	-1.676991639	0.450804	0.091588911	0.239388496	-0.789232904	0.378035947	0.653549586	0.414119465
BnaC03g07960D	0	0.22451918	0	0.2263112	0	0.21244364	0	0.226982722	0	0.223935767
BnaC03g08310D	-0.063220509	0.256463771	-0.274161105	0.3532971	-0.164037534	0.308319243	0.310705309	0.384420462	-0.027797061	0.245231428
BnaC03g08600D	-2.662965013	0.251618144	-2.662965013	0.2496566	2.610053482	0.332617827	-0.078002512	0.226982722	-2.662965013	0.252892846
BnaC03g11590D	0.328280791	0.361695991	-1.888544303	0.6379055	-0.031539091	0.226905668	0.2410081	0.328360016	-0.021810834	0.234699179
BnaC03g12320D	-0.255257055	0.30513359	-0.804469531	0.4066958	-0.611194042	0.392328455	-0.071039337	0.250277396	0.617801114	0.456668956
BnaC03g12860D	1.693896872	0.315703265	-0.180572246	0.2322334	1.45169597	0.289306598	1.436099115	0.299636303	-0.133266531	0.231630209
BnaC03g13270D	0.275234859	0.26094394	-4.487265827	0.4053771	0.205225138	0.241165152	-0.018496344	0.228541424	1.193219285	0.414024798
BnaC03g15160D	0	0.22451918	0	0.2263112	0	0.21244364	4.584962501	0.358510339	0	0.223935767
BnaC03g15430D	2.425204683	0.814468192	-0.154357081	0.2915654	0.706361385	0.535713657	0.586755337	0.481321991	0.700191304	0.568334126

BnaC03g15450D	4.95419631	0.384678044	0	0.2263112	3.321928095	0.262696379	0	0.226982722	0	0.223935767
BnaC03g15570D	-3.115477217	0.266122001	-3.115477217	0.2627866	-3.115477217	0.253588538	0.791413378	0.254244602	-3.115477217	0.268165046
BnaC03g16910D	0	0.22451918	0	0.2263112	0	0.21244364	0	0.226982722	0	0.223935767
BnaC03g17390D	0	0.22451918	0	0.2263112	4.502500341	0.335766054	0	0.226982722	0	0.223935767
BnaC03g20340D	-0.370938404	0.278193138	-1.247927513	0.3451293	-1.167007518	0.351751559	-0.173580172	0.256760981	0.285252445	0.277779245
BnaC03g21050D	-1.402964667	0.408435045	-1.779340546	0.4108259	-0.053515509	0.223662774	-1.413691074	0.411768202	-2.724892762	0.50435468
BnaC03g26060D	-0.135776779	0.243881873	-1.254421276	0.3162008	1.096075972	0.37987865	0.163190167	0.252679295	1.21620855	0.426474603
BnaC03g26090D	-1.767553914	0.408633185	0.688751604	0.3342118	-0.628489876	0.306443517	-1.404983835	0.388293001	0.13014751	0.252943482
BnaC03g26620D	-2.313890776	0.377272007	0.50486891	0.2835253	-0.284143432	0.249035718	-0.506535854	0.283346983	-0.689399911	0.302978266
BnaC03g28550D	-1.088536675	0.414755715	2.582575118	0.7167873	0.200139614	0.266269506	-0.696509153	0.360584205	-0.853158612	0.401863398
BnaC03g32460D	-1.818553129	0.368842245	-2.108059746	0.3622046	-0.045323991	0.21990912	-1.224873411	0.34400428	-1.260062839	0.356458487
BnaC03g35980D	0	0.22451918	0	0.2263112	2	0.229679629	0	0.226982722	0	0.223935767
BnaC03g36160D	-0.224754011	0.296071982	1.831224874	0.6734563	0.460332298	0.37480186	0.434374508	0.366224594	-0.167979596	0.288877294
BnaC03g39750D	-0.318525065	0.366563634	0.249659883	0.3315545	0.014688838	0.218106045	-0.141901525	0.29503725	0.011687911	0.230007661
BnaC03g43590D	2.294183104	0.323551816	3.526545814	0.4256248	-2.807354922	0.244608387	0	0.226982722	0	0.223935767
BnaC03g48630D	-0.081638469	0.258037884	-0.334825089	0.3401207	-0.419713986	0.384715471	0.386238218	0.38365432	-0.014075185	0.229408838
BnaC03g51750D	0	0.22451918	0	0.2263112	0	0.21244364	0	0.226982722	0	0.223935767
BnaC03g55550D	-1.124212027	0.418201152	-2.298890559	0.4773834	0.190102883	0.263185125	-0.068358793	0.244198897	-0.281145009	0.294460442
BnaC03g55560D	-0.895289944	0.580240146	-2.070135801	0.733059	0.735189657	0.5608026	0.462271646	0.442512945	-0.39073361	0.44367977
BnaC03g60080D	-0.290372281	0.342652524	-2.582567986	0.695954	0.391175779	0.389809874	-0.054954826	0.247697171	0.479337145	0.457518757
BnaC03g61550D	-4.231325546	0.5540152	0.564784619	0.3354138	0.495410916	0.332052027	-2.896906507	0.515239177	-0.247092862	0.283683821
BnaC03g64160D	0.090197809	0.235231956	1.072603749	0.3538607	-0.22084197	0.239166138	1.441552018	0.433143119	1.566225938	0.478400525
BnaC03g66110D	0.421858765	0.338049068	0.21553875	0.2777616	-0.136136688	0.253531297	-0.108844996	0.257760488	-0.082060015	0.251516873
BnaC03g66940D	-0.170573841	0.283503294	0.261519992	0.3041121	-0.581722672	0.393521699	-0.347451603	0.334271197	-0.228676796	0.311909983
BnaC03g68390D	-2.14004796	0.48665196	0.793671338	0.3861003	-1.588143748	0.452457378	-0.592928811	0.337465656	-0.837863801	0.390142309
BnaC03g71630D	0.932885804	0.240672003	0.710493383	0.2364582	1.347923303	0.239837613	0	0.226982722	0	0.223935767
BnaC03g71860D	2.736965594	0.255312357	0	0.2263112	2.662965013	0.239348868	2.662965013	0.255299148	0	0.223935767
BnaC03g74080D	-2.041820176	0.295294833	-4.544320516	0.3401207	-0.106915204	0.223292913	-1.544320516	0.287252545	-0.175086707	0.240649988
BnaC03g76670D	-1.628670361	0.610262778	-5.551955395	0.7696137	-0.857871172	0.491352284	-2.368355456	0.675567561	-2.015902495	0.684939589
BnaC04g00590D	1.571809167	0.745660731	-2.724399155	0.7939629	0.549499568	0.497646536	0.556959909	0.491781588	0.549147541	0.534066892
BnaC04g01230D	-2.14974712	0.425208708	-2.219009782	0.404393	-6.564784619	0.561685424	-2.14974712	0.429140688	-2.14974712	0.447957395
BnaC04g03690D	1.439479641	0.600483462	0.22446675	0.2811476	-0.027241239	0.220510145	0.184839675	0.281973211	0.423316977	0.362292613
BnaC04g05500D	-0.394824278	0.325744567	0.297292362	0.3021241	0.33408998	0.317319208	-0.372417865	0.323450544	-0.290779396	0.315148473
BnaC04g08290D	-0.19592021	0.287721477	1.13402966	0.53421	0.145116708	0.266489661	0.805827452	0.475181849	0.402387242	0.373956462
BnaC04g08340D	-1.601738213	0.350644176	-1.305345211	0.3206634	-0.653268514	0.280724929	-2.186700714	0.374097362	-0.244803669	0.257373014
BnaC04g12330D	0	0.22451918	0	0.2263112	0	0.21244364	2.874469118	0.260578481	0	0.223935767
BnaC04g22430D	2.28757659	0.308471151	5.227686721	0.6216206	-0.180572246	0.219924531	1.338801913	0.266113195	2.021061616	0.297934499
BnaC04g27380D	-1.77844223	0.392751154	4.33905658	0.8279946	0.44823755	0.290361143	1.208979704	0.445738226	-0.829426159	0.335763852
BnaC04g32490D	0	0.22451918	0	0.2263112	0	0.21244364	0	0.226982722	2.502500341	0.251349554
BnaC04g35730D	-0.563429339	0.302619412	-0.508387976	0.2853702	0.178337241	0.247672954	-2.885357434	0.446116894	-0.905535316	0.348709007
BnaC04g41990D	0	0.22451918	0	0.2263112	0	0.21244364	3.169925001	0.272455881	4.087462841	0.322490665
BnaC04g43020D	3.115477217	0.266122001	2	0.2407733	3	0.249962574	0	0.226982722	0	0.223935767
BnaC04g44010D	-5.544320516	0.639202244	0.415681416	0.3174139	-0.698830465	0.364428124	-0.989731665	0.413104548	-1.736965594	0.522374423
BnaC04g45680D	0.342392197	0.292241273	1.580662363	0.5181893	-0.443950191	0.289779932	-0.017277991	0.228541424	0.471868722	0.329791028
BnaC04g50810D	-6.94641896	0.610282592	-6.94641896	0.5860919	-1.94641896	0.447721829	-6.94641896	0.615253707	-6.94641896	0.636078235
BnaC04g51450D	-1.03562391	0.278439713	3.624015277	0.634612	-1.965234582	0.291523565	-1.898120386	0.304999296	-0.474908955	0.257617387
BnaC04g52010D	-0.578359766	0.26805717	-0.659659868	0.2650719	-0.308752706	0.240092994	-2.44625623	0.337617563	-0.618437205	0.274402057
BnaC05g00500D	1.610298284	0.601879249	-1.253975067	0.4086772	1	0.477649794	0.950894528	0.461496971	1.462526425	0.61303674
BnaC05g00840D	1.478466246	0.737761545	-5.297680549	0.9109096	0.361668569	0.417157614	0.343122814	0.402041724	0.651132989	0.582820371
BnaC05g04250D	0.983835181	0.498555779	-7.902877533	0.7043485	-0.156923156	0.261776128	-0.044896538	0.236403184	-0.743006196	0.417738825
BnaC05g06410D	-0.161918562	0.254836821	-1.395408693	0.3665306	0.189553808	0.251505865	-0.642930099	0.318263676	-0.229822627	0.267493571
BnaC05g07150D	-0.151442715	0.265140107	1.191067845	0.4999868	-0.072408496	0.233307795	0.496553526	0.35442645	-0.085729874	0.251827292
BnaC05g13640D	-0.426219353	0.381650904	0.999273385	0.5552899	-0.340020271	0.352216087	-0.019011969	0.233655641	-0.56225945	0.457142291
BnaC05g14070D	-0.845526538	0.590701944	-1.41105954	0.6874846	0.313358886	0.391258498	0.364347037	0.410671827	-0.203458472	0.356018176
BnaC05g14250D	-1.226068079	0.36610571	1.682538989	0.5064528	0.61762869	0.326598771	1.080319165	0.425655624	1.016425548	0.436559935
BnaC05g17030D	-0.659683191	0.443466219	-0.399830032	0.3532794	-0.3456575	0.3509612	-0.727342245	0.462729843	-0.159802191	0.300133855
BnaC05g17910D	-1.119264901	0.529060552	1.311628746	0.6065752	0.510026757	0.413494223	0.714489846	0.477733453	1.289999931	0.675979253
BnaC05g20740D	0	0.22451918	0	0.2263112	0	0.21244364	3.700439718	0.298240516	3.662965013	0.294931576
BnaC05g22860D	0.202492864	0.243029871	0.704993204	0.269836	-2.102361718	0.288754007	0.167727446	0.242792103	-4.604862058	0.361596921
BnaC05g30570D	-0.132315229	0.268671404	1.020226658	0.5130244	0.150597643	0.269646694	0.343400902	0.338584047	0.064557812	0.250486544
BnaC05g35410D	2.050140644	0.652662563	2.620300303	0.701984	-0.412693559	0.293799975	-0.64056111	0.338284635	-0.375699351	0.305219451
BnaC05g36490D	-0.548557509	0.459385678	0.121925183	0.2812643	-0.097508053	0.268882754	0.028426934	0.239782574	0.140686828	0.309472859
BnaC05g36990D	-0.197680668	0.263196132	-0.873736227	0.3318759	-0.053771256	0.223662774	-0.657112286	0.327160168	0.383914982	0.308275212
BnaC05g39780D	0	0.22451918	0	0.2263112	0	0.21244364	0	0.226982722	0	0.223935767
BnaC05g40470D	0	0.22451918	0	0.2263112	1.874469118	0.229217302	1.874469118	0.244405844	2.736965594	0.256833633
BnaC05g40480D	2.662965013	0.251618144	0	0.2263112	2.736965594	0.243100321	2.807354922	0.260578481	0	0.223935767
BnaC05g41910D	-0.711037332	0.520547131	-3.385275505	0.8004707	-0.399724564	0.414174504	-0.726791734	0.527585508	-1.261360329	0.700302054
BnaC05g42410D	0	0.22451918	6.62935662	0.5482934	0	0.21244364	0	0.226982722	0	0.223935767
BnaC05g43350D	-1.486498172	0.70803172	-3.393616124	0.8171299	0.346543394	0.403278999	-0.030169969	0.241121121	-0.017796158	0.237710909
BnaC05g43530D	0.686154251	0.382265138	1.82164143	0.593084	0.745370294	0.396665522	0.202338474	0.275689968	0.289801315	0.300695252
BnaC05g44010D	-3.459431619	0.281262109	-3.459431619	0.2775591	-3.459431619	0.268900366	-3.459431619	0.285733471	-3.459431619	0.284370707
BnaC05g44230D	0.816914633	0.470292191	0.543431278	0.3723119	0.311079477	0.317528356	0.454211752	0.365189862	0.279047929	0.327197594
BnaC05g45330D	-0.175442006	0.256001444	-2.607158247	0.4092254	-1.463567393	0.376239477	-1.94596016	0.411543644	-6.448460501	0.570042534
BnaC05g46800D	-0.876794859	0.56244276	-1.377032694	0.6377206	-0.880981137	0.573637677	-0.77298082	0.532655694	-0.133780658	0.303603508
BnaC05g47520D	0.711857376	0.440522738	-1.525610366	0.4957554	0.266850341	0.304010796	0.631687027	0.418949681	0.360385005	0.354981243
BnaC05g48630D	0	0.22451918	0	0.2263112	2.502500341	0.237869421	2.415037499	0.249922946	3.906890596	0.310091497
BnaC05g50690D	-0.3419598	0.318972577	0.027415458	0.2325174	-0.384231452	0.323965709	0.003455559	0.227570538	0.243002701	0.311548927
BnaC05g51000D	2.415037499	0.246506129	2.415037499	0.244965	0	0.21244364	3.415037499	0.283188471	0	0.223935767
BnaC06g02930D	0.122422103	0.262579696	0.882673126	0.4549253	0.117523303	0.253881345	-0.191378111	0.284678925	0.162611193	0.283223696
BnaC06g02940D	0	0.22451918	0	0.2263112	5.554588852	0.436480679	0	0.226982722	6.080373416	0.523389341
BnaC06g12130D	1.518381243	0.73164562	-1.052732243	0.591664	-0.567626478	0.486731216	0.448339765	0.437647504	0.809056195	0.618791609
BnaC06g19980D	0	0.22451918	0	0.2263112	0	0.21244364	0	0.226982722	3	0.264244073
BnaC06g22860D	0.181446871	0.242708443	1.318950395	0.3463974	0.834715385	0.292708003	1.174874217	0.351359682	0.20744208	0.246975061
BnaC06g23020D	0	0.22451918	0	0.2263112	0	0.21244364	0	0.226982722	0	0.223935767
BnaC06g23040D	-0.513122499	0.298751277	-2.26710407	0.406436	1.205603977	0.46044903	-2.792195115	0.450971327	0.167665494	0.260855877
BnaC06g24140D	-1.133266531	0.310998538	1.674088391	0.4262346	1.657370996	0.451475483	0.612976877	0.297910282	1.026279325	0.370094931
BnaC06g25000D	-0.762823762	0.484003487	-0.012328029	0.2302432	0.852512388	0.554175913	0.053897315	0.247314101	-0.11284147	0.281869738
BnaC06g29460D	0	0.22451918	5.36923381	0.4104076	4.247927513	0.316359329	4.222392421	0.330838969	0	0.223935767
BnaC06g30790D	0	0.22451918	0	0.2263112	2.584962501	0.239348868	0	0.226982722	0	0.223935767
BnaC06g32180D	-0.363963315	0.262529061	0.165586066	0.2450003	1.535380472	0.440207915	-1.326810316	0.328861971	-0.029544276	0.227819314
BnaC06g33880D	1.27462238	0.454253848	0.345011708	0.2739573	1.195550809	0.442464511	-0.519568083	0.296954806	-0.246837178	0.26631794
BnaC06g34360D	0	0.22451918	0	0.2263112	0	0.21244364	0	0.226982722	0	0.223935767
BnaC06g35340D	-3.736965594	0.29336847	-1	0.2516556	-0.929610672	0.239205766	0.510961919	0.253707422	1.247927513	0.298936208
BnaC06g35350D	0	0.22451918	0	0.2263112	7.50779464	0.686564338	2.807354922	0.260578481	3.700439718	0.297287242
BnaC06g35510D	0.100821532	0.261353429	-1.264624403	0.5063097	0.767736469	0.492668815	0.685431166	0.461695111	0.841803717	0.547060481
BnaC06g36010D	5.745954377	0.46631838	0	0.2263112	0	0.21244364	0	0.226982722	4.807354922	0.381567244
BnaC06g39570D	0	0.22451918	0	0.2263112	0	0.21244364	3.736965594	0.298240516	5.297680549	0.429594209
BnaC06g43380D	0	0.22451918	0	0.2263112	2.662965013	0.239348868	0	0.226982722	0	0.223935767
BnaC07g00540D	2.4064485	0.603999348	0.710941077	0.3105032	-0.529619467	0.270377611	0.361674274	0.277006499	-1.057551023	0.335288316
BnaC07g03440D	-6.351675438	0.537113847	-6.351675438	0.5160846	-6.351675438	0.533934799	-6.351675438	0.542296312	-6.351675438	0.558623058
BnaC07g08320D	0	0.22451918	2.736965594	0.2526947	0	0.21244364	0	0.226982722	0	0.223935767
BnaC07g08510D	0	0.22451918	0	0.2263112	0	0.21244364	0	0.226982722	0	0.223935767
BnaC07g10840D	-2.593198651	0.678458206	-2.885154666	0.663503	-1.116846648	0.546554123	-0.494764692	0.391051552	-1.056766044	0.567911427

BnaC07g13600D	-1.543291631	0.656262109	1.955988088	0.7424024	0.047198444	0.238617951	0.743600866	0.524274367	0.999762461	0.651229349
BnaC07g16030D	-0.953146187	0.397951231	1.627289498	0.5756917	-0.637741215	0.346853095	-1.008428622	0.408153246	0.997195927	0.509310384
BnaC07g18400D	-0.858350182	0.540026507	0.641081533	0.4757146	-0.597816424	0.473874124	-0.561916388	0.45371887	-0.270772595	0.368866462
BnaC07g25120D	-0.002970037	0.225129011	2.194197272	0.7532033	-0.046737155	0.235544577	-0.442746483	0.385428775	0.024272271	0.234699179
BnaC07g25380D	-0.511713519	0.271713956	2.092169844	0.4958523	-0.777607579	0.280192152	0.922832139	0.343136866	0.989946335	0.365095195
BnaC07g25960D	0	0.22451918	0	0.2263112	2.874469118	0.244608387	2.874469118	0.260578481	0	0.223935767
BnaC07g27140D	-1.496997211	0.705180704	-5.3105219	0.8838635	-0.201579332	0.325497992	-1.112813742	0.642984519	-0.005280934	0.230007661
BnaC07g28070D	0.37227518	0.281620962	2.58271356	0.6228183	0.020084246	0.214971028	0.065887936	0.236828085	0.13504996	0.250004403
BnaC07g28680D	0	0.22451918	0	0.2263112	0	0.21244364	0	0.226982722	2.415037499	0.247556272
BnaC07g31380D	0	0.22451918	0	0.2263112	0	0.21244364	4.115477217	0.321827997	3.459431619	0.284370707
BnaC07g31550D	0	0.22451918	3.115477217	0.2627866	0	0.21244364	0	0.226982722	3.115477217	0.268165046
BnaC07g31640D	-1.573589205	0.722964881	-0.004288208	0.2278017	-1.220728221	0.680193033	-2.914116427	0.822686604	-0.162380178	0.327364912
BnaC07g32760D	0.062872478	0.248315809	1.137795597	0.58428	-0.01778134	0.22070168	0.059022208	0.250722111	-0.063217805	0.255184667
BnaC07g33430D	-1.374218595	0.587560323	0.583369243	0.4259947	0.026819598	0.225329353	-0.340559283	0.351064673	0.179447776	0.311804308
BnaC07g33650D	-1.054098734	0.57835121	-1.59269815	0.6366947	-0.51476768	0.435454754	-0.297850741	0.355152964	-0.174413453	0.317660449
BnaC07g34310D	0.33113115	0.369799923	0.171597754	0.2965101	-0.190356131	0.3055717	-0.401976969	0.38950826	-0.445648958	0.439567262
BnaC07g34790D	0	0.22451918	0	0.2263112	0	0.21244364	0	0.226982722	0	0.223935767
BnaC07g35020D	-0.618693358	0.417556096	-2.560917027	0.633542	0.00504263	0.215010656	0.106121186	0.26959826	0.737571641	0.524507732
BnaC07g36700D	-0.0489096	0.227618972	0.468148836	0.2662431	-0.736965594	0.269135933	0.560714954	0.286728574	1.659163443	0.461087481
BnaC07g37430D	0	0.22451918	0	0.2263112	2.807354922	0.244608387	3.807354922	0.304259572	3.772589504	0.301360122
BnaC07g39760D	0	0.22451918	2.807354922	0.2544626	0	0.21244364	0	0.226982722	2.874469118	0.258500211
BnaC07g43900D	-0.251061764	0.253934182	0.896164189	0.3201218	0.222392421	0.241380905	0.096861539	0.239646078	0.766712938	0.32976461
BnaC07g45320D	1.126997666	0.619714062	0.630300457	0.4492365	0.147321988	0.283714643	0.763541247	0.514015111	0.134421499	0.292373366
BnaC07g46430D	4.48112669	0.343167688	2.807354922	0.2544626	0	0.21244364	0	0.226982722	5.073248982	0.407728345
BnaC07g47650D	-2.807354922	0.256930501	-2.807354922	0.2544626	2.349149564	0.321896245	0.777607579	0.250946669	0.817135943	0.248835376
BnaC07g48440D	-1.723371292	0.719352126	-0.547004445	0.4464603	-0.057700107	0.247774226	0.26265728	0.354063193	-0.348311859	0.419493466
BnaC08g06060D	-3.222392421	0.268539311	0.777607579	0.2505504	2.111508315	0.326402832	3.135159583	0.441865687	0.86507042	0.259354415
BnaC08g10700D	-0.700112959	0.491438145	0.248363176	0.3286858	-0.03796785	0.233382648	0.25604586	0.344246451	-0.048744334	0.25361936
BnaC08g17410D	-0.055444327	0.243401934	0.411796215	0.3474079	-0.063541795	0.236522068	-0.426305226	0.354930607	-0.046054368	0.244965039
BnaC08g17650D	0	0.22451918	0	0.2263112	0	0.21244364	0	0.226982722	0	0.223935767
BnaC08g18080D	1.697696951	0.566810648	-0.004459648	0.2263112	0.797141109	0.380431241	-0.205423683	0.266472049	0.207044457	0.271610483
BnaC08g18090D	0	0.22451918	5.247927513	0.4002409	3.584962501	0.275137377	3.502500341	0.285733471	4.89077093	0.389404787
BnaC08g18260D	0	0.22451918	0	0.2263112	0	0.21244364	0	0.226982722	0	0.223935767
BnaC08g18470D	0	0.22451918	6.648657176	0.5500458	0	0.21244364	0	0.226982722	2.662965013	0.252892846
BnaC08g18480D	-0.761147802	0.570297915	-0.267611023	0.3595693	0.11017333	0.283774085	0.440005561	0.448901863	0.299969468	0.418137307
BnaC08g20580D	-2.938599455	0.260714978	2.38332864	0.325848	1.88582898	0.294748846	-0.064130337	0.226982722	0.935869663	0.256159956
BnaC08g23560D	-0.234108891	0.3187216	0.871957686	0.5210843	-0.301373917	0.341063	0.059351401	0.251954983	-0.086705676	0.270787101
BnaC08g24310D	0	0.22451918	0	0.2263112	0	0.21244364	0	0.226982722	0	0.223935767
BnaC08g26660D	-0.736965594	0.348231269	0.934411658	0.4064823	0.014178935	0.213982528	0.099773135	0.251186639	-0.639410285	0.350131213
BnaC08g28910D	-0.116023796	0.243914897	0.915607813	0.3530285	0.131529623	0.236363556	3.20E-16	0.226982722	0.632268215	0.33621297
BnaC08g30340D	1.925999419	0.529734228	2.187627003	0.5392604	0.321928095	0.258198598	0.49683159	0.296021346	0.328325866	0.273261651
BnaC08g30630D	0	0.22451918	0	0.2263112	0	0.21244364	0	0.226982722	2.874469118	0.258500211
BnaC08g31190D	-0.556922681	0.438990454	-1.483311438	0.6083012	-1.397154794	0.638988693	-1.636136814	0.66493184	-1.230175493	0.647352408
BnaC08g36920D	-0.784184318	0.502580225	0.006249952	0.2296026	0.283848889	0.347119483	0.680549083	0.500299412	0.615263654	0.522394237
BnaC08g37160D	0	0.22451918	0	0.2263112	0	0.21244364	3.584962501	0.291867008	0	0.223935767
BnaC08g37480D	0	0.22451918	0	0.2263112	0	0.21244364	0	0.226982722	3	0.264244073
BnaC08g37720D	0.496483677	0.382608581	1.090774753	0.5247631	-0.887220615	0.445797668	0.12400664	0.268671404	-0.259686731	0.315883793
BnaC08g38370D	0	0.22451918	2.736965594	0.2526947	2.736965594	0.243100321	0	0.226982722	4.247927513	0.334339445
BnaC08g42690D	0	0.22451918	0	0.2263112	0	0.21244364	2.662965013	0.255299148	2.662965013	0.252892846
BnaC08g48430D	-0.326732888	0.341380024	0.367434809	0.3497085	-0.389978025	0.359291891	-0.280499548	0.327626898	-0.389978025	0.383889887
BnaC08g48630D	-2.284586841	0.802802142	0.787882525	0.554253	-0.742387267	0.569608827	-2.958728106	0.837516292	-0.540787306	0.531204868
BnaC09g01940D	-0.750106694	0.39934922	0.755164331	0.4198523	-0.257066683	0.28915469	-1.489169229	0.510805241	-0.507648649	0.372190813
BnaC09g02330D	0.596644306	0.272988658	2.436863862	0.4693169	0.449802917	0.249324122	0.085391491	0.236273292	-0.249027548	0.2443442
BnaC09g02660D	-0.992305608	0.320106379	2.011526253	0.5175794	-0.137503524	0.235036017	-1.224966365	0.341749886	-0.558209773	0.29182738
BnaC09g03530D	-4.029747343	0.311179066	-4.029747343	0.3056399	-1.527247003	0.258504614	-0.570315725	0.250647258	-0.060120992	0.229884374
BnaC09g05060D	0	0.22451918	0	0.2263112	2.662965013	0.239348868	0	0.226982722	0	0.223935767
BnaC09g05300D	-1.676592976	0.365046761	-1.022367813	0.313396	0.682688533	0.317380852	0.28748745	0.269413329	1.814734452	0.556828789
BnaC09g05650D	0.253815876	0.301109144	0.161528286	0.2693913	0.27978356	0.302962855	0.275234859	0.310595653	1.449540347	0.65623569
BnaC09g07170D	-1.102497074	0.439056501	0.158889479	0.2647042	-0.281094377	0.28699056	-0.449971289	0.330471309	0.290761135	0.316084135
BnaC09g10430D	0.197345003	0.29283129	-0.3143435	0.3071084	0.344919738	0.333183627	-0.256098359	0.307022526	0.187202993	0.298874564
BnaC09g11720D	0	0.22451918	3.222392421	0.2654263	0	0.21244364	0	0.226982722	0	0.223935767
BnaC09g12530D	0	0.22451918	0	0.2263112	0	0.21244364	0	0.226982722	0	0.223935767
BnaC09g13310D	-2.662965013	0.251618144	-2.662965013	0.2496566	-2.662965013	0.239348868	-2.662965013	0.255299148	-2.662965013	0.252892846
BnaC09g15550D	-0.297748282	0.336362676	-0.085381579	0.2581083	-0.437151339	0.381571647	-0.366290465	0.35971459	-0.330078936	0.367602769
BnaC09g29430D	-2.072860402	0.746145074	-1.714914349	0.6848427	-0.132956838	0.286048293	-0.044857123	0.245147769	0.122991107	0.299079309
BnaC09g29440D	1.073336612	0.443380359	-0.697676463	0.3155029	0.344758803	0.282114111	-0.634482637	0.323877646	-6.763765654	0.612961887
BnaC09g33810D	2.77118131	0.530890045	2.150559677	0.4207924	1.263034406	0.329027088	0.99034983	0.309512487	1.492280498	0.376259291
BnaC09g34140D	2.126305317	0.572296928	-0.323788437	0.2598013	0.717499772	0.323948096	-1.260062839	0.34654928	-0.956056652	0.329097538
BnaC09g35120D	-1.371367028	0.63372477	-1.859202361	0.6639874	-0.033880893	0.228466572	-0.28553303	0.350432826	-0.117183539	0.291076649
BnaC09g36060D	-4.415037499	0.338183363	-4.415037499	0.3309579	-4.415037499	0.327201997	-4.415037499	0.343660837	-4.415037499	0.345725897
BnaC09g40110D	2.736965594	0.255312357	2.584962501	0.2496566	4.029747343	0.299295061	0	0.226982722	0	0.223935767
BnaC09g40660D	1.064599672	0.648173588	-3.325639106	0.8160159	0.525585308	0.485414685	0.529497931	0.476507186	0.903028791	0.650813255
BnaC09g41070D	0	0.22451918	0	0.2263112	0	0.21244364	0	0.226982722	0	0.223935767
BnaC09g41280D	-1.527247003	0.545994928	-4.919564426	0.6844376	0.265602518	0.309155835	-0.849680332	0.444190532	-0.122856748	0.272279756
BnaC09g42570D	0.956599036	0.484556078	-1.182034684	0.4286255	0.491365782	0.357233435	0.053797995	0.239716528	1.40789407	0.630897971
BnaC09g42790D	0	0.22451918	-3.415037499	0.2749172	-3.415037499	0.266500669	-3.415037499	0.283188471	-3.415037499	0.281841118
BnaC09g44160D	0.902702799	0.273404752	-0.665580961	0.2477368	0.283792966	0.228363098	-0.61667136	0.250755134	0.646363045	0.262051323
BnaC09g45060D	-0.371968777	0.240403413	-1.067114196	0.2546387	0.398549376	0.234930343	-1	0.258636708	-0.652076697	0.248683469
BnaC09g46360D	1.75802721	0.321772958	-3.502500341	0.2775591	1.216317907	0.27224233	1.831400396	0.331312304	1.706953025	0.328476699
BnaC09g47470D	-1.097862013	0.540550477	0.24081486	0.3098449	0.191074256	0.296510092	-0.58328884	0.421635581	-0.506343151	0.430074148
BnaC09g49710D	-0.232258574	0.262962767	-3.67914494	0.4427727	0.517252273	0.3082576	-0.532303551	0.3026018	0.227745656	0.269367096
BnaC09g50770D	0.310424123	0.304308007	-0.188929662	0.2665975	-0.342252697	0.29464097	-0.385821711	0.313149459	-0.133602588	0.263119078
BnaC09g50990D	2.584962501	0.251618144	3.459431619	0.2775591	5.029747343	0.382276146	4.058893689	0.319509757	2.584962501	0.252892846
BnaC09g54190D	-0.677422676	0.473744232	-2.563477442	0.7026753	0.132773049	0.278998908	0.397616064	0.395005548	-0.140862536	0.299266441
BnaCnng01000D	0	0.22451918	0	0.2263112	0	0.21244364	0	0.226982722	0	0.223935767
BnaCnng01390D	1.042176793	0.525139579	-1.284956461	0.4607815	0.158586285	0.266723027	-0.331320965	0.317026401	0.185494159	0.292384374
BnaCnng01850D	0	0.22451918	0	0.2263112	0	0.21244364	0	0.226982722	0	0.223935767
BnaCnng02030D	-0.448959886	0.384867378	0.36099766	0.3522909	0.41856919	0.39038448	0.525610366	0.42903061	0.528337063	0.463826218
BnaCnng03200D	-0.004078293	0.225809292	0.169087627	0.3066637	-0.067531206	0.254935891	-0.137931203	0.300519127	-0.267725438	0.383843654
BnaCnng03520D	1.13676858	0.468577178	1.253235058	0.4629544	-0.189761417	0.253000722	0.561878888	0.342476399	-0.004046833	0.223935767
BnaCnng06440D	-0.98269763	0.554283789	0.227357166	0.3177903	-0.098512123	0.263676072	-0.016994284	0.23414879	-0.235248459	0.346663761
BnaCnng06970D	0.429684275	0.250237768	-1.527247003	0.2647857	0.670692375	0.251208655	-4.029747343	0.316059918	-1.527247003	0.273442178
BnaCnng12230D	-2.415037499	0.246506129	-2.415037499	0.244965	0	0.21244364	0.087462841	0.226982722	-2.415037499	0.247556272
BnaCnng13370D	3.415037499	0.278780954	0	0.2263112	3.772589504	0.285134647	3.772589504	0.301791627	2.415037499	0.247556272
BnaCnng13680D	0.932885804	0.254942495	2.295455884	0.3170924	0.8259706	0.239005425	-2.874469118	0.260578481	1.2410081	0.269107313
BnaCnng16520D	0.896435357	0.422133133	-0.878107695	0.3449377	1.069177488	0.46887659	0.910036623	0.426421765	1.48724268	0.589180669
BnaCnng17820D	-0.058893689	0.22451918	-3.058893689	0.2596164	-3.058893689	0.249962574	-3.058893689	0.26634656	-3.058893689	0.264244073
BnaCnng17910D	3.807354922	0.299596675	3.772589504	0.2922413	6.564784619	0.561685424	5.087462841	0.403131495	5.772589504	0.485231956
BnaCnng19930D	-2.807354922	0.256930501	-2.807354922	0.2544626	-0.144389909	0.219651538	-2.807354922	0.260578481	-0.144389909	0.231790923
BnaCnng21270D	-2.557995453	0.330680457	-5.142957954	0.3895391	1.375367354	0.374887721	-1.598637438	0.316015886	-0.683526335	0.275073532
BnaCnng22510D	1.695488958	0.532501585	-0.882278041	0.3149966	1.037474705	0.409170365	0.511064387	0.312079503	1.292424101	0.4881226
BnaCnng23360D	0	0.22451918	0	0.2263112	0	0.21244364	0	0.226982722	0	0.223935767
BnaCnng24780D	0	0.22451918	0	0.2263112	2.938599455	0.248216739	3.321928095	0.279293917	0	0.223935767

BnaCnng28030D	-1.088579046	0.511051816	-3.382310249	0.6590339	-0.080297871	0.245497816	0.102285293	0.265232572	0.65004659	0.4871319
BnaCnng28540D	0	0.22451918	0	0.2263112	0	0.21244364	0	0.226982722	0	0.223935767
BnaCnng29120D	-1.803254412	0.485967276	-4.041659152	0.5512456	-0.11388119	0.24317077	-0.679715378	0.361189633	-0.594575925	0.361975589
BnaCnng31260D	0.859438672	0.38943781	0.482738992	0.3030751	0.257541407	0.264371764	0.068822033	0.240390204	0.718044732	0.381483585
BnaCnng31270D	0.128054559	0.292258885	0.100470325	0.2740828	-0.194132293	0.318290095	-0.359567994	0.394501391	-0.59381133	0.528507961
BnaCnng35720D	0	0.22451918	0	0.2263112	5.357552005	0.415103561	0	0.226982722	0	0.223935767
BnaCnng35740D	0	0.22451918	0	0.2263112	5.357552005	0.415103561	0	0.226982722	0	0.223935767
BnaCnng43220D	0.582519325	0.31016635	-1.73940877	0.3507388	0.877794068	0.351469759	-2.534588978	0.396746979	-1.336649601	0.362448924
BnaCnng43450D	3.109624491	0.476463155	3.348262243	0.4821123	1.493539473	0.298546532	1.506959989	0.311810913	2.3594028	0.405445331
BnaCnng44540D	-1.087462841	0.282142731	-1.161463423	0.2784815	-0.142604395	0.227376801	-4.824428435	0.377267604	-2.017073513	0.312792807
BnaCnng46710D	-1.253756592	0.461613653	-4.259050892	0.5922606	-0.129767875	0.252161929	-0.075050705	0.248844183	-0.811238357	0.420724136
BnaCnng47140D	-3.948367232	0.462791486	-2.626439137	0.4028299	-0.522102477	0.284542428	-0.893084796	0.332582602	-0.626439137	0.314915108
BnaCnng49340D	-2.874469118	0.256930501	-2.874469118	0.2544626	-2.874469118	0.244608387	-0.137503524	0.23380975	-2.874469118	0.258500211
BnaCnng49840D	-0.181750437	0.268299341	-1.959358016	0.4575562	0.22575339	0.272215911	-0.091147888	0.249594914	0.612183969	0.394589454
BnaCnng51960D	4.029747343	0.311179066	0	0.2263112	3	0.249962574	0	0.226982722	2.115477217	0.242580753
BnaCnng52970D	0	0.22451918	0	0.2263112	3.36923381	0.262696379	0	0.226982722	0	0.223935767
BnaCnng52990D	0.109902995	0.248641639	-3.396129037	0.4798227	-1.244125943	0.400095548	-0.212907213	0.270511906	0.13438568	0.257786907
BnaCnng58140D	0	0.22451918	0	0.2263112	0	0.21244364	0	0.226982722	2.736965594	0.256833633
BnaCnng59970D	-0.040845052	0.233547765	1.670054438	0.6013068	0.030472352	0.220510145	-0.126177106	0.26293855	0.502918331	0.379112508
BnaCnng61080D	-2.584962501	0.251618144	-2.584962501	0.2496566	0.830074999	0.235586407	0.078002512	0.226982722	-2.584962501	0.252892846
BnaCnng61660D	0	0.22451918	5.014950341	0.3782649	2.874469118	0.244608387	3.807354922	0.304259572	0	0.223935767
BnaCnng62330D	-1.219699318	0.493239019	-1.75105135	0.519552	0.464715147	0.367296752	0.081743149	0.253174645	-0.114399373	0.266637166
BnaCnng62710D	0.620470602	0.39913787	0.511925465	0.3520069	0.468467509	0.355751788	1.170603119	0.550448677	1.549948911	0.665438198
BnaCnng63810D	0	0.22451918	0	0.2263112	0	0.21244364	2.807354922	0.260578481	2.807354922	0.258500211
BnaCnng65380D	0.635114705	0.377179541	-7.351675438	0.6366661	0.052046748	0.226421325	-1.135738039	0.421728046	-0.171766348	0.270005548
BnaCnng67570D	1.481470623	0.520778294	0.19379693	0.2583967	0.306170909	0.274593593	-1.312738923	0.391328948	-0.234736411	0.271117334
BnaCnng68780D	0	0.22451918	2.415037499	0.244965	3.36923381	0.262696379	0	0.226982722	0	0.223935767
BnaCnng69610D	4.029747343	0.311179066	0	0.2263112	0	0.21244364	0	0.226982722	0	0.223935767
BnaCnng72020D	0.231822667	0.31537083	-0.543500358	0.3859175	-0.434257701	0.373824369	-0.740250896	0.457820371	-0.472241675	0.413001074
BnaCnng76050D	1.770469854	0.632738473	-2.227068909	0.478832	0.526846755	0.351623868	0.866552726	0.439807232	0.434514874	0.346489838
BnaCnng78680D	-1.846144871	0.702926309	-1.514976104	0.6340462	-0.090184813	0.261153087	-0.999597181	0.578311582	-0.730667654	0.548964828
BnaUnng04070D	0.711709894	0.444067244	1.234856387	0.5540372	0.26251273	0.302929832	0.173744873	0.284654708	0.029427972	0.235368453

BnNAC

GeneID	log2(Cold/Control)	Prob.	log2(Heat/Control)	Prob.	log2(Drought/Contro	Prob.	log2(Salt/Control	Prob.	log2(ABA/Control)	Prob.
BnaA01g01440D	-0.614108846	0.280322044	0.415037499	0.2687925	0.146220741	0.233012787	0.931052646	0.347403484	0.371094152	0.274538554
BnaA01g07600D	-0.332575339	0.257320177	-0.133266531	0.2400005	0.606024116	0.286131953	0.75802721	0.315529342	-0.332575339	0.258918507
BnaA01g08340D	-4.736965594	0.365418824	-4.736965594	0.3561613	0.776103988	0.277572299	1.744161096	0.405044648	3.837943242	0.720120469
BnaA01g08370D	-0.442004547	0.30082074	-2.791413378	0.4532742	0.066567617	0.22956735	-1.040641984	0.379770774	-0.215728691	0.272222516
BnaA01g08830D	0	0.22451918	2.662965013	0.2496566	0	0.21244364	0	0.226982722	0	0.223935767
BnaA01g16400D	2.505717881	0.805397777	2.696600388	0.7977716	-0.217836354	0.308748547	0.333813704	0.360174716	0.749964824	0.552795537
BnaA01g26760D	0	0.22451918	0	0.2263112	0	0.21244364	0	0.226982722	2.662965013	0.252892846
BnaA01g28710D	0.241621099	0.259918014	-0.107528464	0.2390781	-0.841634404	0.297547025	-1.617202838	0.358902216	-1.040414268	0.334387879
BnaA01g28930D	-0.277109591	0.249398975	2.942856093	0.5710244	-0.664132714	0.260047906	-0.642106408	0.271641305	-2.516575526	0.338101906
BnaA01g28990D	0.961525852	0.271161365	-3.662965013	0.2864842	-1	0.239685706	-3.662965013	0.295451143	-1	0.253185653
BnaA01g30220D	-3.700439718	0.29336847	-3.700439718	0.2890842	-3.700439718	0.28129293	2.149226009	0.373870601	-3.700439718	0.297287242
BnaA01g31650D	-0.384812241	0.38714599	1.25953746	0.6416041	-0.164797784	0.295636074	0.519536602	0.450788598	0.072911266	0.266879337
BnaA01g31660D	-0.967484838	0.599347459	0.585103025	0.4751554	-0.349917672	0.394730353	0.109758161	0.285172074	-0.364498655	0.429352038
BnaA01g31670D	0.090123646	0.263647451	1.726465182	0.7057685	-0.001836658	0.213101906	0.139282296	0.290000088	-0.294204956	0.364047254
BnaA01g31680D	0	0.22451918	0	0.2263112	0	0.21244364	0	0.226982722	0	0.223935767
BnaA01g33090D	0	0.22451918	2.662965013	0.2496566	0	0.21244364	0	0.226982722	0	0.223935767
BnaA01g33110D	0	0.22451918	0	0.2263112	0	0.21244364	3.415037499	0.283188471	0	0.223935767
BnaA01g34470D	-1.557995453	0.308686903	-2.142957954	0.3118549	-1.268488836	0.291844993	-1.727920455	0.319736518	-1.027480736	0.29657834
BnaA02g01460D	0.344804696	0.390804977	0.383551727	0.3943781	1.376084774	0.717141763	0.398862036	0.416822977	0.005602706	0.229849149
BnaA02g04040D	-2.89704281	0.48532222	0.476932033	0.3102192	-1.382469637	0.407248406	-1.273347914	0.4039879	-0.575114715	0.333571101
BnaA02g05540D	-3.645716532	0.518457853	2.748815312	0.7110523	0.036711778	0.220651044	1.102259843	0.471461217	0.992544195	0.472150305
BnaA02g05600D	0.754703871	0.419249093	-3.118786152	0.5325654	-2.076350886	0.52183284	-0.796858058	0.385921924	0.484009641	0.369190091
BnaA02g09970D	2.415037499	0.246506129	0	0.2263112	2.415037499	0.234239054	0	0.226982722	2.415037499	0.247556272
BnaA02g12310D	0.263034406	0.249255874	0.407424315	0.2556954	-0.736965594	0.260629117	0.953706348	0.320260488	-0.503766418	0.260741396
BnaA02g18100D	-2.557995453	0.330680457	-2.557995453	0.3185301	-2.557995453	0.322140618	-2.557995453	0.334931664	-5.142957954	0.413141974
BnaA02g18390D	0.773248299	0.44974506	2.082687282	0.6917622	1.044339539	0.535095019	1.257470244	0.581015094	1.584962501	0.684175649
BnaA02g20430D	0.788917674	0.33580348	-5.906890596	0.4657196	0.779609932	0.327897689	0.180572246	0.255255116	-0.404390255	0.275295889
BnaA02g26420D	-0.683863185	0.374476029	0.26340485	0.2880649	-0.058986315	0.231491511	-0.971387261	0.42488728	-0.850221572	0.426978759
BnaA02g33380D	-0.678071905	0.251006112	-4	0.3056399	-4	0.299295061	-4	0.316059918	-4	0.316335112
BnaA02g33910D	1.519026602	0.713352883	2.410019425	0.7864578	0.48626754	0.441845873	1.336525471	0.685956709	0.848801701	0.606429867
BnaA02g34250D	-1.531804565	0.423196485	0.089963377	0.2434262	-1.856531293	0.444454718	-1.083600202	0.388700289	-1.95806932	0.476804396
BnaA03g01250D	0.161216065	0.311872556	2.059417201	0.7726056	-0.237790236	0.348682588	0.3248077	0.388257776	-0.004900598	0.225741044
BnaA03g02640D	0.962245078	0.59406152	2.937673315	0.8206303	0.000443565	0.21244364	0.554173476	0.460543697	-0.35649876	0.405440928
BnaA03g02680D	-0.839781719	0.474096481	0.917647978	0.5139425	-0.495573461	0.391749445	-0.417223638	0.368371112	-0.308880376	0.353730758
BnaA03g04240D	-0.536512849	0.381186375	2.44864103	0.7541367	1.970800024	0.731901	0.960708854	0.532490577	1.768160216	0.735896826
BnaA03g04640D	0.336624606	0.313109831	-2.480511337	0.5082933	0.509146719	0.355368717	-0.686962214	0.370165381	0.145093148	0.271667723
BnaA03g04920D	0	0.22451918	0	0.2263112	4.169925001	0.310820212	0	0.226982722	0	0.223935767
BnaA03g06460D	2	0.278307619	-2.115477217	0.2407733	1.299560282	0.239822202	-0.115477217	0.226982722	1.657112286	0.265391085
BnaA03g07000D	-1.569020957	0.391073567	0.390789953	0.2834108	-0.335348611	0.266496266	-1.206450877	0.370530839	0.42303029	0.30779087
BnaA03g08460D	-0.402632478	0.273466395	0.660208395	0.3072603	0.128642151	0.234472419	0.259292004	0.266828701	-0.494424954	0.287131459
BnaA03g08510D	1.913960169	0.443582902	-0.503766418	0.2543261	-0.984893108	0.271744778	-0.030696797	0.228935503	1.13058411	0.354750079
BnaA03g19110D	3.906890596	0.305333932	0	0.2263112	0	0.21244364	2.938599455	0.264316725	0	0.223935767
BnaA03g19960D	-2	0.241636285	2.297680549	0.2813171	0.807354922	0.22873296	2.392317423	0.297036264	-2	0.242580753
BnaA03g24360D	0	0.22451918	0	0.2263112	2.502500341	0.237869421	0	0.226982722	0	0.223935767
BnaA03g27400D	-0.064440999	0.248549174	0.49892267	0.4007164	0.300509111	0.3440329	0.726142854	0.497978971	0.583443075	0.487737328
BnaA03g31260D	0.369025102	0.326944415	-0.562495161	0.3378509	-0.039348667	0.224503769	-0.369054113	0.317821163	-0.301054013	0.312299658
BnaA03g31270D	-0.199769512	0.276161101	0.4439724	0.324886	-0.282063634	0.283666209	-0.149241954	0.267812797	-0.185555653	0.278512364
BnaA03g32520D	-1.655783838	0.349415707	-0.525723297	0.2711261	-1.296241451	0.324500687	-0.669458775	0.293681091	-1.655783838	0.36299491
BnaA03g32550D	0.047305715	0.227618972	-0.125530882	0.2353508	0.913288367	0.276418683	0.396890153	0.25318125	-0.0489096	0.229285551
BnaA03g33770D	-0.160464672	0.235593011	-1.662965013	0.2713815	0.233199176	0.228226602	-4.247927513	0.332813766	-0.662965013	0.255519303
BnaA03g33890D	3.969626351	0.308750749	5.169925001	0.3931739	0	0.21244364	0	0.226982722	0	0.223935767
BnaA03g33910D	0	0.22451918	0	0.2263112	0	0.21244364	0	0.226982722	0	0.223935767
BnaA03g39450D	-1.298034889	0.439170982	-0.762703156	0.3501818	0.183403836	0.262154796	-0.022855053	0.230692346	0.208150499	0.28413514
BnaA03g43500D	-2.874469118	0.256930501	-2.874469118	0.2544626	0.788495895	0.235542376	-2.874469118	0.260578481	-2.874469118	0.258500211
BnaA03g48570D	1.380257395	0.697679999	3.25412559	0.8393458	0.254810622	0.34295854	1.052866734	0.630856141	1.960497919	0.803460407
BnaA03g49310D	2.308122295	0.326422646	-2.807354922	0.2544626	1.308122295	0.254477967	2.280107919	0.327745782	1.695145418	0.293276005
BnaA03g49460D	-0.606178987	0.277920145	0.111421283	0.2390781	-0.277853121	0.242235109	-1.370111628	0.32417926	-0.031942893	0.227819314
BnaA03g58970D	0	0.22451918	0	0.2263112	4.700439718	0.351890257	0	0.226982722	4.718818247	0.37162061
BnaA04g09470D	1.248411071	0.401645444	1.813903708	0.4641432	1.018262206	0.360022808	0.962767094	0.357484413	1.334735871	0.439499014
BnaA04g10830D	0	0.22451918	0	0.2263112	3.321928095	0.262696379	0	0.226982722	0	0.223935767
BnaA04g19530D	-1.564719281	0.53588758	-2.685618714	0.581997	-0.390162831	0.331389359	-1.271842863	0.506426345	-1.461785758	0.56139702
BnaA04g24740D	-1.046693235	0.313473088	0.080418682	0.2357163	0.398866209	0.264864912	0.550869302	0.296565131	0.398866209	0.279919159
BnaA05g01110D	0	0.22451918	0	0.2263112	3.273018494	0.259946634	2.415037499	0.249922946	0	0.223935767
BnaA05g10110D	-1.046012398	0.337624168	1.70689474	0.4907689	1.24894865	0.43901247	1.791557137	0.540226848	0.1991001	0.260556466
BnaA05g17900D	0.336427665	0.273583078	0.313157885	0.2641252	1.330645312	0.440000969	-0.334735871	0.27053172	-0.280628466	0.265217162
BnaA05g17950D	0	0.22451918	0	0.2263112	0	0.21244364	0	0.226982722	0	0.223935767
BnaA05g18390D	-0.67556505	0.306771549	1.981058436	0.5388112	0.154509949	0.241173958	1.215205881	0.438920004	0.879023802	0.387925341
BnaA05g18430D	0.256945241	0.272004562	2.690388855	0.6685182	-0.747419952	0.316498027	-0.20085283	0.263020008	-0.894573646	0.3520928
BnaA05g22540D	-2.047934477	0.430078552	-1.374744793	0.3705683	-0.664930805	0.314912906	-1.971985624	0.430567297	-0.344498506	0.287576174

BnaA05g24040D	-0.009235391	0.226716334	-0.619567546	0.3799909	0.617471651	0.433607647	0.629423039	0.433270809	0.298326823	0.344123164
BnaA05g24050D	0.992840208	0.499051129	3.782064471	0.8355437	-0.520122568	0.345961464	0.565100731	0.386443693	-0.70734319	0.409956321
BnaA05g26240D	0	0.22451918	0	0.2263112	2.807354922	0.244608387	0	0.226982722	0	0.223935767
BnaA05g26290D	2.502500341	0.250160714	0	0.2263112	2.584962501	0.239348868	0	0.226982722	0	0.223935767
BnaA05g28370D	-0.005327384	0.227803903	0.739826058	0.5063317	-0.03981245	0.233842774	-0.015627978	0.234492233	0.083824993	0.272502113
BnaA05g32430D	-2.874469118	0.256930501	-2.874469118	0.2544626	0.540568381	0.228607471	-2.874469118	0.260578481	-2.874469118	0.258500211
BnaA05g34370D	3.772589504	0.296939396	0	0.2263112	2.807354922	0.244608387	0	0.226982722	0	0.223935767
BnaA05g37400D	2.046693235	0.43334346	2.658963082	0.4890142	0.785495488	0.274983268	1.329501315	0.34734184	0.275634443	0.247589295
BnaA05g37410D	5.235216462	0.412186498	8.027905997	0.7195789	4.718818247	0.351890257	5.718818247	0.468081827	5.849665727	0.494500511
BnaA06g01770D	1.940920767	0.517396703	2.037360528	0.5030029	0.671377253	0.304325619	-0.481869008	0.275980573	0.367509129	0.276803956
BnaA06g08290D	2.584962501	0.251618144	2.502500341	0.2479504	2.502500341	0.237869421	2.502500341	0.253714027	2.502500341	0.251349554
BnaA06g15870D	0.069974024	0.250929057	0.871912476	0.4984501	-0.060347775	0.238562912	-0.639261231	0.427383846	-0.313715846	0.352748864
BnaA06g19740D	-0.85025288	0.288436983	-5.415037499	0.4158961	-2.415037499	0.339590158	-1.476438044	0.322477456	0.285402219	0.258379126
BnaA06g21090D	1.154328146	0.476080084	2.848858474	0.7194798	0.643235542	0.357325901	-0.263034406	0.278695093	0.195550809	0.272491106
BnaA06g21810D	2.874469118	0.256930501	0	0.2263112	0	0.21244364	0	0.226982722	0	0.223935767
BnaA06g22900D	0.959488452	0.481544348	1.629172144	0.597267	0.432621183	0.337690215	0.588002955	0.384713269	0.767681614	0.462707827
BnaA06g23210D	1.714245518	0.31300856	3.154818109	0.4326213	1.87036472	0.316103949	-1	0.251534485	1.209453366	0.284412537
BnaA06g23510D	-1.736140961	0.583604125	-0.067114196	0.2478733	0.373935872	0.350888548	0.352775965	0.346963172	0.261796572	0.331517049
BnaA06g27060D	0.196325737	0.310813607	1.263361956	0.6215436	-0.215855505	0.310399715	-0.083618815	0.261771725	-0.101783738	0.276568389
BnaA06g31080D	1.056583528	0.379700324	3.288569498	0.6896531	-0.222392421	0.241380905	0.875218375	0.350168639	-1.750771394	0.371710874
BnaA06g34140D	-0.41349203	0.320936366	0.09643976	0.2499273	0.428313042	0.336144722	-0.167028604	0.273757001	-0.976990022	0.446002413
BnaA06g39010D	-1.72631835	0.380473071	-6.22881869	0.5018911	-1.045596866	0.333586512	-0.184424571	0.257111029	-0.546994651	0.300710663
BnaA06g40380D	2.736965594	0.255312357	3.624490865	0.2834218	3.624490865	0.275137377	0	0.226982722	0	0.223935767
BnaA07g02060D	-0.515211392	0.305598119	-2.97783835	0.4442302	-0.1406335	0.242182271	-1.515211392	0.410057593	-0.934510918	0.369231921
BnaA07g03200D	0.379673214	0.304814365	-2.168037892	0.4378941	-1	0.375563599	-2.577428828	0.486757635	0.335460508	0.305032319
BnaA07g05000D	1.234853321	0.61196238	1.421951607	0.6196392	-0.040483577	0.228649301	0.556215912	0.421276727	0.283951373	0.344138575
BnaA07g08370D	0.876617584	0.284108722	-0.760812336	0.2531526	3.20E-16	0.21244364	-1.608809243	0.284487389	0.278716028	0.244500511
BnaA07g10300D	3.45037646	0.764882525	3.501813016	0.7522764	0.154920577	0.241173958	0.579109774	0.324650393	0.921997488	0.40513271
BnaA07g12690D	0.149848209	0.249117176	0.20069735	0.252261	0.446980392	0.280176741	-0.127489735	0.24882657	0.592122292	0.322889147
BnaA07g13130D	-0.337869639	0.266027335	-0.519109953	0.272676	0.380948609	0.266256296	0.099535674	0.241973123	-0.44170545	0.27883159
BnaA07g14730D	-0.074000581	0.228660309	6.40599236	0.8681466	0.867896464	0.255241907	2.185866545	0.378586336	2.329123596	0.409949716
BnaA07g15270D	-0.387023123	0.265166526	-2.350497247	0.3463357	-0.097516506	0.225897355	-0.362570079	0.265430713	1.266174113	0.424719962
BnaA07g17790D	0.005088877	0.22451918	-0.127849955	0.2465325	-0.190461932	0.248196925	-1.262015193	0.378623763	-0.769618811	0.340037074
BnaA07g24270D	-1.309173115	0.702373719	1.622145701	0.7288717	-0.042400417	0.23856071	0.685717025	0.546968016	0.543300149	0.536715365
BnaA07g28000D	-0.519496158	0.441894308	2.96995336	0.8242519	0.040418312	0.234485628	1.045037214	0.628438832	1.277931924	0.720450703
BnaA07g29730D	2.662965013	0.251618144	3.938599455	0.3001427	2.584962501	0.239348868	0	0.226982722	3.459431619	0.284370707
BnaA07g32710D	3.415037499	0.278780954	2.502500341	0.2479504	3.969626351	0.297018652	0	0.226982722	0	0.223935767
BnaA07g35010D	1.417085328	0.367252721	-0.350907162	0.2467637	0.373458396	0.245777414	0.398549376	0.261040808	-0.626541604	0.265192944
BnaA07g37110D	-0.630613613	0.316834866	0.286493379	0.2714674	0.069262662	0.227916182	-1.793233814	0.420986121	-0.538419915	0.31624705
BnaA08g00130D	0.407877708	0.284460971	-0.704015172	0.2912418	0.551241883	0.29704507	0.531613076	0.305879918	1.227484891	0.450306457
BnaA08g06490D	0.019306426	0.230751788	0.377899865	0.3543098	0.053760482	0.235544577	0.030594498	0.236627743	-0.219009782	0.321854415
BnaA08g13670D	-2.584962501	0.251618144	-2.584962501	0.2496566	-2.584962501	0.239348868	-2.584962501	0.255299148	-2.584962501	0.252892846
BnaA08g18520D	-3.510405539	0.661224418	3.699047827	0.843379	0.224973805	0.294011325	0.223782971	0.302637025	-0.291982019	0.329060111
BnaA08g24840D	0.736965594	0.244328789	-0.169925001	0.2322334	-0.169925001	0.219924531	-0.08246216	0.226982722	0.784271309	0.245229226
BnaA08g29850D	0	0.22451918	1.874469118	0.2401326	0	0.21244364	0	0.226982722	0	0.223935767
BnaA08g30060D	0	0.22451918	1.874469118	0.2401326	0	0.21244364	0	0.226982722	0	0.223935767
BnaA09g00360D	1.584962501	0.237046039	0	0.2263112	0	0.21244364	0	0.226982722	0	0.223935767
BnaA09g00440D	0	0.22451918	0	0.2263112	2.662965013	0.239348868	0	0.226982722	0	0.223935767
BnaA09g02500D	0	0.22451918	6.163230349	0.4948748	2.321928095	0.234239054	3.222392421	0.272455881	0	0.223935767
BnaA09g04810D	-0.008782073	0.227337173	1.538164726	0.6506658	-0.313924555	0.330649635	-0.469560118	0.381056483	-0.210122336	0.314972348
BnaA09g06060D	0	0.22451918	0	0.2263112	0	0.21244364	0	0.226982722	0	0.223935767
BnaA09g06870D	-0.062644937	0.235652453	1.910428282	0.5809336	-0.024732521	0.216633203	0.731232481	0.377082673	-0.532787036	0.322761457
BnaA09g08940D	1.018859027	0.53330075	-2.191343985	0.5590854	-0.275234859	0.302258357	-0.352624892	0.328749692	-0.940498988	0.485295801
BnaA09g08950D	0	0.22451918	2.736965594	0.2526947	4.89077093	0.36890609	2.736965594	0.258914104	0	0.223935767
BnaA09g12800D	0	0.22451918	-2.502500341	0.2479504	1.527247003	0.258504614	-2.502500341	0.253714027	-2.502500341	0.251349554
BnaA09g13880D	0	0.22451918	0	0.2263112	0	0.21244364	0	0.226982722	0	0.223935767
BnaA09g19070D	2.206450877	0.458177023	0.222392421	0.241469	0.703282468	0.268774878	0.534776744	0.270465673	1.018378529	0.323135722
BnaA09g19790D	0	0.22451918	6.491853096	0.5318851	0	0.21244364	0	0.226982722	0	0.223935767
BnaA09g19800D	0	0.22451918	4.196397213	0.316126	0	0.21244364	0	0.226982722	0	0.223935767
BnaA09g23230D	-0.02127666	0.232356723	-0.303899218	0.3372521	-0.064795123	0.24390609	0.262673326	0.340248424	0.009715155	0.229144651
BnaA09g24190D	-2.502500341	0.250160714	-2.502500341	0.2479504	-2.502500341	0.237869421	-2.502500341	0.253714027	-2.502500341	0.251349554
BnaA09g28720D	0.045196553	0.237942073	0.905123669	0.4619175	-0.476975575	0.346456814	-0.06501353	0.247732396	-0.345517341	0.334293212
BnaA09g35990D	0	0.22451918	0	0.2263112	0	0.21244364	0	0.226982722	0	0.223935767
BnaA09g41400D	0	0.22451918	0	0.2263112	0	0.21244364	0	0.226982722	0	0.223935767
BnaA09g47260D	0.479167837	0.244066804	0.277533976	0.2362975	-0.090197809	0.218825954	0.977973694	0.271245024	-3.459431619	0.284370707
BnaA09g51460D	0.469485283	0.247340519	-1.478047297	0.2550526	-0.530514717	0.23200007	-3.700439718	0.298240516	-0.530514717	0.245055303
BnaA09g51470D	1.94753258	0.277810067	0	0.2263112	-2.222392421	0.233252756	-2.222392421	0.248806756	-2.222392421	0.246453292
BnaA09g51610D	0	0.22451918	0	0.2263112	2.115477217	0.229679629	0	0.226982722	0	0.223935767
BnaA09g51620D	1.351036952	0.672853042	-0.371383149	0.3570507	1.754258229	0.743987548	1.352643389	0.677491722	-0.221806793	0.334766547
BnaA10g00280D	0.518989612	0.466890785	2.866433871	0.8241528	0.63317529	0.521456374	1.135374286	0.667175226	0.71446022	0.587256508
BnaA10g00830D	1.585785722	0.612710909	0.510615159	0.3410828	0.973825117	0.482658336	0.709532364	0.411244232	1.037791385	0.534168164
BnaA10g00870D	-1.523561956	0.26665698	-1.523561956	0.2614855	-0.665580961	0.238778664	-3.938599455	0.310144334	-3.938599455	0.310091497
BnaA10g00890D	0	0.22451918	3.772589504	0.2922413	2.938599455	0.248216739	3.415037499	0.283188471	0	0.223935767
BnaA10g00910D	0	0.22451918	4.169925001	0.316126	0	0.21244364	0	0.226982722	0	0.223935767
BnaA10g00920D	2.779609932	0.332269981	4.35520425	0.4820353	2.751320887	0.323725739	0	0.226982722	2.341036918	0.310448149
BnaA10g05960D	-0.986127825	0.381666314	0.388662084	0.2992444	-0.493814613	0.307931769	-1.786596362	0.458249674	-0.221811743	0.277006499
BnaA10g13930D	3.969626351	0.308750749	3	0.2596164	3.058893689	0.249962574	3.058893689	0.26634656	0	0.223935767
BnaA10g14070D	-0.223407332	0.259424865	2.313380575	0.5951425	0.437686238	0.286486403	-0.621956708	0.305494646	1.28368426	0.48099616
BnaA10g19630D	0.534501166	0.342311283	-0.2403792	0.2677401	-1.311586151	0.417142203	-1.821260524	0.461393497	-1.896548651	0.490187661
BnaA10g20110D	-1.078411687	0.604124837	2.083163997	0.7636408	-0.098699086	0.268046162	0.384421683	0.400485223	-0.043149871	0.249181021
BnaA10g22600D	0.845392661	0.514039329	0.942357224	0.5146756	0.111634525	0.260116154	-0.375866902	0.35289857	-0.074161348	0.25793221
BnaA10g22680D	1.909473027	0.73321533	3.010673502	0.8094442	0.201545137	0.295510585	-0.295641403	0.32958188	-0.543725468	0.426269858
BnaA10g25760D	0.90738885	0.530016027	1.243593318	0.5869835	0.139346852	0.271315474	-0.216176578	0.304774737	0.226809693	0.324355384
BnaA10g30260D	0	0.22451918	0	0.2263112	2.736965594	0.243100321	0	0.226982722	0	0.223935767
BnaAnng05950D	1.786864194	0.769899873	2.456595562	0.8012544	0.141090068	0.295653686	-0.166734924	0.315375233	-0.495390783	0.497587094
BnaAnng06380D	0.750972452	0.260468403	3.231162631	0.4775023	0.342887714	0.231696256	2.034765418	0.365975818	1.017487427	0.283021153
BnaAnng07000D	-1.954976075	0.533619976	-0.193239146	0.2713727	-0.375396771	0.313596375	-0.346034712	0.314681743	0.771879491	0.472749128
BnaAnng08430D	0.497499659	0.247435186	-0.628031223	0.2423496	0.46712601	0.231916411	0	0.226982722	-0.563900885	0.245055303
BnaAnng11180D	3.584962501	0.287446282	0	0.2263112	0	0.21244364	0	0.226982722	0	0.223935767
BnaAnng17650D	-1.661414561	0.34216598	0.873622714	0.3221891	0.444974783	0.270214696	0.301304423	0.266183645	-0.752562449	0.298881169
BnaAnng19940D	-3.169925001	0.268539311	1.637429921	0.2886461	-0.94753258	0.232222428	-3.169925001	0.272455881	-0.94753258	0.245312885
BnaAnng32450D	0	0.22451918	3.807354922	0.2947973	0	0.21244364	0	0.226982722	2.415037499	0.247556272
BnaAnng33660D	0.57475643	0.337368787	-1.755977563	0.4024952	0.084639813	0.232191606	-0.894953976	0.353922294	-0.166116263	0.260855877
BnaAnng34420D	-0.517848305	0.395049579	1.203043675	0.5864639	0.209247045	0.300825144	0.86402233	0.524129064	0.391953886	0.396319877
BnaAnng34430D	-0.689456669	0.350408609	1.40753681	0.5191051	-0.477260014	0.31117026	-0.156209204	0.265622248	-0.246746751	0.285150058
BnaC01g02440D	0	0.22451918	0	0.2263112	0	0.21244364	0	0.226982722	0	0.223935767
BnaC01g02450D	-2.493040011	0.365117211	-0.181838323	0.2451632	-0.058102955	0.220875603	0.946829341	0.352079591	0.64689025	0.315648226
BnaC01g09270D	-0.38827059	0.253009528	-0.544996093	0.2546255	-0.171952683	0.227865547	-4.89077093	0.384616401	0.704175659	0.292657367
BnaC01g09890D	-4.938599455	0.383147962	-2.523561956	0.3080154	-0.064130337	0.217751594	0.542527234	0.275315703	2.958237476	0.621673447
BnaC01g09920D	-0.597748656	0.343828155	-1.699732688	0.4475127	-0.824428435	0.374623534	-0.506513403	0.330599	-0.539026217	0.349114093
BnaC01g10510D	0	0.22451918	2.662965013	0.2496566	0	0.21244364	0	0.226982722	0	0.223935767
BnaC01g19550D	-0.201715005	0.287087428	0.670546729	0.4079639	-0.203582572	0.278739124	1.106335506	0.555701592	0.931880846	0.542421801
BnaC01g23890D	3.415037499	0.278780954	0	0.2263112	5.790076931	0.462470059	2.502500341	0.253714027	0	0.223935767

BnaC01g36040D	0	0.22451918	0	0.2263112	4.058893689	0.30296946	0	0.226982722	0	0.223935767
BnaC01g36350D	0	0.22451918	0	0.2263112	0	0.21244364	0	0.226982722	0	0.223935767
BnaC01g38180D	0	0.22451918	0	0.2263112	2.662965013	0.239348868	3.584962501	0.291867008	0	0.223935767
BnaC01g40250D	-0.084888898	0.244209905	4.219887614	0.8529448	0.565597176	0.363611346	2.096326117	0.690522738	2.601356939	0.776365846
BnaC01g40260D	0.528467614	0.306115485	2.888602981	0.6652423	1.136294224	0.416083254	0.668901319	0.33206964	0.163261271	0.254874247
BnaC01g43150D	0	0.22451918	0	0.2263112	0	0.21244364	0	0.226982722	0	0.223935767
BnaC02g00940D	-0.615261101	0.42623023	1.20783177	0.5932117	-0.269752028	0.320016115	-0.372176285	0.356821744	-0.011509748	0.228367501
BnaC02g00990D	1.632550582	0.750697894	2.37318192	0.7957484	-1.18705323	0.668425711	-0.481929383	0.448085086	-0.533708676	0.511586794
BnaC02g01470D	0.024247546	0.226284829	1.262015193	0.3561613	-2.712718048	0.335706612	0.30718151	0.259931223	0	0.223935767
BnaC02g02740D	-0.640608472	0.444201539	0.722088521	0.47313	-0.561704445	0.426364525	-0.198413558	0.307004914	-0.1278641	0.288613107
BnaC02g04540D	-0.219183131	0.343447286	0.012675717	0.2323149	-0.206843778	0.337536106	0.495880332	0.471765032	-0.07977871	0.280991317
BnaC02g07990D	-2.552296312	0.484633133	0.812067049	0.3786634	-1.142652071	0.395441456	-1.305502547	0.414771126	0.370299185	0.314485804
BnaC02g09420D	2.415037499	0.246506129	0	0.2263112	0	0.21244364	0	0.226982722	0	0.223935767
BnaC02g10100D	-4.087462841	0.316995579	-4.087462841	0.3109633	-0.028569152	0.21244364	-4.087462841	0.321827997	-4.087462841	0.322490665
BnaC02g10310D	0	0.22451918	0	0.2263112	0	0.21244364	0	0.226982722	0	0.223935767
BnaC02g10340D	0	0.22451918	3.624490865	0.2834218	2.736965594	0.243100321	0	0.226982722	2.736965594	0.256833633
BnaC02g13890D	0	0.22451918	0	0.2263112	0	0.21244364	0	0.226982722	3.321928095	0.277510656
BnaC02g20880D	0	0.22451918	0	0.2263112	0	0.21244364	0	0.226982722	0	0.223935767
BnaC02g24050D	-0.959358016	0.268149635	-4.544320516	0.3401207	-1.084888898	0.261190514	-1.041820176	0.27570758	-1.959358016	0.297373102
BnaC02g25890D	-2.502500341	0.250160714	-2.502500341	0.2479504	-2.502500341	0.237869421	-2.502500341	0.253714027	-2.502500341	0.251349554
BnaC02g27990D	-0.075037054	0.243225809	-1.189033824	0.4045273	0.261627585	0.281724436	-0.754887502	0.37037673	-0.257981178	0.29145972
BnaC02g34540D	-1.342003872	0.494388231	0.452163618	0.3425997	-0.152808843	0.262044718	-0.246455664	0.297872856	-1.466699619	0.543242981
BnaC02g42720D	1.425995312	0.713978125	2.831033673	0.8215484	0.492241947	0.460360967	1.194230973	0.675422259	0.482571784	0.48987504
BnaC02g43170D	-4.36923381	0.336391296	-2.784271309	0.290163	1.284208429	0.316559671	-1.784271309	0.288106749	-0.494764692	0.2516974
BnaC03g03740D	1.279681072	0.662045599	2.747364543	0.805477	0.010011031	0.217033886	0.487020525	0.42870478	-0.652953982	0.504493378
BnaC03g03780D	-0.153951365	0.290180616	1.037474705	0.571053	-0.718480274	0.481599387	-0.343505173	0.358748107	-0.493496777	0.439972348
BnaC03g06170D	0.188823367	0.29669062	-1.499232627	0.5390798	0.128254491	0.266687802	-0.96871791	0.493265437	-0.187202993	0.303616718
BnaC03g08290D	0.430634354	0.240480468	-2.938599455	0.257569	0.38332864	0.224836204	-0.938599455	0.247538659	0.476438044	0.241385308
BnaC03g08880D	1.037474705	0.326794709	1.102569734	0.3219293	-1.046293652	0.274195111	1.066769012	0.33557672	1.649312912	0.429889218
BnaC03g10790D	-1.444784843	0.266588732	1.575114715	0.3204344	-0.029747343	0.21244364	2.488577964	0.437246821	0.908852112	0.279846507
BnaC03g23960D	-3.321928095	0.275018493	-0.658963082	0.2423738	0.707819249	0.238873331	0.584962501	0.250755134	-0.584962501	0.241482176
BnaC03g32420D	-0.577766999	0.369564356	-0.249393495	0.2861958	0.092545742	0.244496108	0.538538164	0.386817958	0.357552005	0.349446529
BnaC03g36600D	0.723959884	0.394891067	0.482782106	0.32268	0.611434712	0.366284036	-0.075948853	0.245799429	-0.043816232	0.236367959
BnaC03g36610D	0.402935732	0.313596375	1.71440947	0.5683627	0.18097727	0.257623992	0.47654564	0.332774138	0.10617719	0.254229191
BnaC03g38960D	-1.026967048	0.290035313	-5.169925001	0.3931739	-0.688798312	0.26310807	-1.026967048	0.293353059	-1.08246216	0.300992462
BnaC03g39160D	7.662965013	0.700007045	4.841302254	0.3646725	2.662965013	0.239348868	0	0.226982722	0	0.223935767
BnaC03g50150D	0.540568381	0.281290729	1.482016199	0.3853385	-1.964666927	0.322362975	-1.427010141	0.316660943	-0.23236271	0.250037426
BnaC03g50570D	0.494925271	0.432920762	1.498805857	0.6768313	1.026390747	0.621389447	1.214905663	0.657255011	-0.376074344	0.40954463
BnaC03g52400D	0.475379456	0.409357498	2.917039778	0.8092791	-0.468516381	0.393079186	-0.801656834	0.481170083	-0.100267106	0.273690954
BnaC03g53950D	1.347350692	0.360511554	-4.906890596	0.370216	-4.906890596	0.370240234	0.062735755	0.23086847	0.727315424	0.295809997
BnaC03g54890D	-6.539158811	0.559538906	-3.124121312	0.4295172	-0.118496763	0.235639244	-0.509411468	0.301512029	-1.09069831	0.37733365
BnaC03g58370D	-3.4018964	0.795094491	2.529637246	0.7987116	-0.937715722	0.577932914	0.841104881	0.566295484	0.351329011	0.417168622
BnaC03g60970D	-1.019091964	0.485467523	-0.901054506	0.435195	-1.044947406	0.497906319	-0.170241139	0.286235426	-0.110323415	0.270040773
BnaC03g70990D	0.148627239	0.268010937	-2.471524691	0.5115472	-0.215184937	0.271315474	-0.212391722	0.281427225	0.174467243	0.27942601
BnaC03g71120D	-0.078002512	0.229063193	-4.247927513	0.3211411	-1.584962501	0.266287118	-4.247927513	0.332813766	-0.703606997	0.255607366
BnaC03g71220D	-0.154593348	0.303002483	2.428249475	0.7976087	0.208428527	0.329167988	0.421360248	0.424438163	0.106066809	0.294367977
BnaC03g71470D	0.207764807	0.321233576	1.114790899	0.6055008	0.626456243	0.496310191	0.698813698	0.511393057	0.267487534	0.368866462
BnaC03g73730D	-2.201633861	0.332045423	2.049327712	0.4597027	0.176877762	0.233613812	-0.823122238	0.285997658	-0.801095932	0.284903484
BnaC03g74980D	-4.938599455	0.383147962	-2.523561956	0.3080154	-1.61667136	0.293978301	-0.523561956	0.262258269	-2.436099115	0.328091426
BnaC04g00680D	0	0.22451918	0	0.2263112	0	0.21244364	3.415037499	0.283188471	0	0.223935767
BnaC04g11030D	0	0.22451918	3.255982783	0.4995663	-3.969626351	0.297018652	-3.969626351	0.313622794	-3.969626351	0.31401247
BnaC04g11040D	0	0.22451918	2.874469118	0.2544626	0	0.21244364	0	0.226982722	0	0.223935767
BnaC04g16940D	1.880701072	0.390001409	1.308122295	0.3145805	1.465663572	0.336010427	-0.070389328	0.23154655	1.80407979	0.398836257
BnaC04g31690D	0.214443668	0.267982317	2.822468695	0.6990384	0.600294154	0.334722516	1.760357443	0.578278559	1.777992862	0.615317553
BnaC04g36420D	3.502500341	0.281262109	0	0.2263112	0	0.21244364	0	0.226982722	0	0.223935767
BnaC04g43920D	-2.467778961	0.426104741	-1.091630475	0.3385312	-1.056583528	0.346201434	0.07566427	0.241697929	-0.315008681	0.27709236
BnaC04g48620D	0	0.22451918	0	0.2263112	4.700439718	0.351890257	0	0.226982722	0	0.223935767
BnaC04g51650D	-2.502500341	0.250160714	-2.502500341	0.2479504	-2.502500341	0.237869421	-2.502500341	0.253714027	-0.087462841	0.223935767
BnaC04g52880D	1.724365557	0.29448466	0.68589141	0.2450817	1.283792966	0.258266846	1.705256734	0.294755451	0.798366139	0.252382085
BnaC05g00370D	1.644054158	0.75772086	1.578646266	0.7217364	0.333247739	0.39864252	0.551919946	0.490823911	0.131063756	0.312112526
BnaC05g00890D	2.874469118	0.256930501	0	0.2263112	0	0.21244364	0	0.226982722	2.874469118	0.258500211
BnaC05g00910D	2.003160341	0.479968033	0.347019076	0.2550768	2.267772325	0.522742083	1.520256811	0.411592078	-0.309328058	0.253463049
BnaC05g00930D	0	0.22451918	0	0.2263112	0	0.21244364	0	0.226982722	0	0.223935767
BnaC05g00940D	1.510595055	0.649965656	0.747797314	0.4396267	-0.041000463	0.227555127	-0.671119144	0.408536317	-0.960132617	0.499070943
BnaC05g00970D	-0.087462841	0.22451918	-2.502500341	0.2479504	-2.502500341	0.237869421	-2.502500341	0.253714027	1.338801913	0.264957378
BnaC05g01000D	-0.064130337	0.22451918	4.283792966	0.5353988	2.605721061	0.352200676	0.833990049	0.25431285	0.833990049	0.252439325
BnaC05g01020D	2.184424571	0.41104389	4.332382453	0.6747618	-4.196397213	0.310820212	-0.974004791	0.264556694	-4.196397213	0.3284789
BnaC05g01030D	0	0.22451918	4.142957954	0.3140147	0	0.21244364	0	0.226982722	2.415037499	0.247556272
BnaC05g01040D	0	0.22451918	6.559695742	0.5393594	0	0.21244364	0	0.226982722	2.222392421	0.246453292
BnaC05g09630D	-0.021061616	0.22451918	-0.154328146	0.2358	-0.131244533	0.22382569	-2.301169535	0.299270844	0.350907162	0.251069957
BnaC05g20530D	1.28298948	0.606438673	1.082083689	0.5315813	-0.016441147	0.218742295	-0.165843825	0.284161559	0.227637531	0.315676847
BnaC05g21870D	0.781654412	0.292199443	-1.431339312	0.2871403	-0.367208974	0.240559724	-0.325986311	0.252833404	-0.367208974	0.253995826
BnaC05g24890D	0	0.22451918	2.321928095	0.244965	2.321928095	0.234239054	0	0.226982722	0	0.223935767
BnaC05g28010D	-1.273018494	0.357548258	0.965386245	0.3671955	-1.398549376	0.359472419	0.351472371	0.283199479	0.078656944	0.239947691
BnaC05g28020D	0.908021678	0.351599651	3.08246216	0.6599321	-0.280107919	0.24683196	-0.354108501	0.267284424	0.277109591	0.265217162
BnaC05g28390D	0	0.22451918	0	0.2263112	0	0.21244364	0	0.226982722	0	0.223935767
BnaC05g35830D	-0.038474148	0.227088397	-1.662965013	0.327781	1.93446684	0.511245553	0.403124178	0.276302001	0.169925001	0.248945454
BnaC05g38130D	1.157541277	0.427628219	1.682304378	0.4923628	1.060372618	0.408615573	0.653197236	0.333736218	0.645335119	0.344299288
BnaC05g38150D	1.675008107	0.582930448	4.09078269	0.8334258	0.055424039	0.226687714	0.328692502	0.297516203	-0.328535149	0.293786766
BnaC05g40360D	-3.772589504	0.296939396	-3.772589504	0.2922413	-0.03562391	0.21244364	-3.772589504	0.301791627	-0.965234582	0.256159956
BnaC05g40430D	0	0.22451918	2.321928095	0.244965	0	0.21244364	0	0.226982722	0	0.223935767
BnaC05g42540D	-0.11092715	0.275760418	0.374432716	0.3753875	0.014252155	0.217921114	-0.057104451	0.254193966	-0.189424499	0.327778805
BnaC05g47490D	2.736965594	0.255312357	2.736965594	0.2526947	2.736965594	0.243100321	2.807354922	0.260578481	2.807354922	0.258500211
BnaC05g47500D	1.211504105	0.331752615	2.227805918	0.4310759	-0.365284464	0.237876026	-0.341036918	0.250013209	-0.415037499	0.251424407
BnaC06g05920D	2.776790604	0.658071788	5.390823176	0.886349	0.470791481	0.280991317	1.994107432	0.547738561	3.091147888	0.730720966
BnaC06g05930D	0	0.22451918	4.029747343	0.3056399	0	0.21244364	0	0.226982722	0	0.223935767
BnaC06g08090D	-0.242673452	0.293681091	0.881773919	0.4520941	0.663350399	0.42255363	-0.011027022	0.22910062	-0.223308128	0.297740762
BnaC06g12550D	-0.047123912	0.235302406	4.627399414	0.8757507	-0.066984334	0.232134365	1.122801089	0.509083624	0.045633229	0.239555814
BnaC06g13440D	0	0.22451918	0	0.2263112	0	0.21244364	0	0.226982722	3.662965013	0.294931576
BnaC06g30680D	1.681256162	0.50159833	2.402689107	0.5853764	2.986273017	0.699102205	2.712718048	0.664738103	2.759736902	0.700189774
BnaC06g32940D	4.415037499	0.338183363	0	0.2263112	0	0.21244364	0	0.226982722	0	0.223935767
BnaC06g37170D	0	0.22451918	-3.415037499	0.2749172	0.523561956	0.235220948	-3.415037499	0.283188471	-3.415037499	0.281841118
BnaC06g39940D	3.273018494	0.272268748	3.807354922	0.2947973	0	0.21244364	0	0.226982722	0	0.223935767
BnaC06g43410D	-2.3582108	0.648464194	1.828724145	0.6925218	1.626170321	0.700132534	0.62275242	0.443875709	0.866495488	0.561289144
BnaC07g06080D	1.25885873	0.412741291	-0.926833587	0.2975426	-0.776416745	0.289810754	-0.762202886	0.301681549	-0.539810464	0.286098929
BnaC07g10350D	2.951744831	0.371327803	0.839535328	0.2452072	1.706268797	0.269987935	2.438573014	0.331904523	1.818161677	0.293403695
BnaC07g11950D	-0.468238894	0.32372794	-0.322603672	0.2877171	-0.199609026	0.264076755	-0.725305577	0.369029378	-0.601264313	0.36541222
BnaC07g13550D	1.99149762	0.717874881	2.160647188	0.7127056	-0.386548942	0.331389359	-0.390584456	0.337355578	-0.11882066	0.270212494
BnaC07g16780D	-1.231005552	0.405093082	-0.820293978	0.3388196	-0.459891735	0.300318785	-0.201081915	0.269908679	0.359704746	0.312370108
BnaC07g19280D	2.736965594	0.255312357	4.222392421	0.3190364	2.736965594	0.243100321	0	0.226982722	0	0.223935767
BnaC07g21770D	0.037198618	0.23809398	0.098817736	0.2617035	0.348243696	0.356978055	0.078569052	0.258872274	-0.507668477	0.425578569
BnaC07g25530D	0	0.22451918	4.271887412	0.6917688	0.944290567	0.282699725	2.155278225	0.434199866	-0.022026306	0.223935767
BnaC07g29890D	0.919163068	0.591100426	1.449312268	0.6804682	-0.139197741	0.288271866	-0.238434436	0.337342369	0.038528708	0.248758322

BnaC07g34980D	0	0.22451918	2.736965594	0.2526947	2.736965594	0.243100321	0	0.226982722	2.736965594	0.256833633
BnaC07g40860D	1.517680598	0.733184508	2.714279057	0.8157935	0.283142995	0.366464564	0.921296252	0.607797034	1.835530536	0.801450386
BnaC07g41360D	-1.161154792	0.306298214	-1.242074787	0.2987953	0.314873337	0.24739776	-0.228268988	0.25200782	-0.03562391	0.228581053
BnaC07g41390D	-0.125530882	0.230485399	-3.584962501	0.2834218	1.337869639	0.282572035	2.407503827	0.392055462	2.466699619	0.410625594
BnaC07g41850D	-0.521537121	0.333485241	-0.453365618	0.3081387	-1.032789935	0.412950439	-0.793909391	0.378035947	-0.983880335	0.429495139
BnaC07g46020D	1.934904972	0.288042904	2.901221846	0.3417521	0	0.21244364	0	0.226982722	2.94596016	0.372463806
BnaC07g49940D	-1.584962501	0.270740868	3.330389674	0.5233585	0.612976877	0.247800645	1.304854582	0.315980662	1.762202886	0.371732889
BnaC08g07040D	-0.4164841	0.370513227	1.115410483	0.5746548	0.379245497	0.370654127	0.323488796	0.349889042	0.072742121	0.259970851
BnaC08g07270D	0	0.22451918	0	0.2263112	5.64385619	0.446543996	0	0.226982722	0	0.223935767
BnaC08g07470D	3.662965013	0.290878509	4.624490865	0.3475356	2.736965594	0.243100321	2.736965594	0.258914104	4.969626351	0.395547131
BnaC08g13100D	-2.584962501	0.251618144	3.029747343	0.3573193	1.736965594	0.270016556	-0.08246216	0.226982722	0.830074999	0.248835376
BnaC08g15440D	3.321928095	0.275018493	4.222392421	0.3190364	0	0.21244364	2.415037499	0.249922946	0	0.223935767
BnaC08g21160D	0.783580178	0.479937212	1.649841337	0.6535586	-0.321928095	0.322061362	-0.623913482	0.404437018	0.133488866	0.280346261
BnaC08g34030D	0	0.22451918	0	0.2263112	2.584962501	0.239348868	2.584962501	0.255299148	3.544320516	0.288610906
BnaC08g41360D	1.50779464	0.299715559	-0.607682577	0.2423496	2.076815597	0.334848005	-0.04580369	0.226982722	-0.04580369	0.223935767
BnaC08g43050D	0	0.22451918	4.790076931	0.3605402	0	0.21244364	0	0.226982722	0	0.223935767
BnaC08g49760D	0	0.22451918	5.073248982	0.384645	0	0.21244364	0	0.226982722	0	0.223935767
BnaC08g49770D	0	0.22451918	0	0.2263112	0	0.21244364	0	0.226982722	0	0.223935767
BnaC09g04330D	0.286446817	0.332113671	1.595115217	0.6589964	0.026249899	0.224398094	0.035226254	0.239104495	-0.178739476	0.301921519
BnaC09g05610D	-2.662965013	0.251618144	-2.662965013	0.2496566	-2.662965013	0.239348868	-2.662965013	0.255299148	-0.160464672	0.231936225
BnaC09g06370D	-1.930291028	0.513535172	0.529965223	0.3477447	-0.63584367	0.359437194	0.35115062	0.320143806	0.218305464	0.292994206
BnaC09g09210D	1.558558529	0.675662228	-1.39273205	0.5251506	-0.411724765	0.355359911	0.155977462	0.287657631	-0.477928854	0.401702684
BnaC09g13140D	4.584962501	0.352933795	0	0.2263112	2.938599455	0.248216739	0	0.226982722	0	0.223935767
BnaC09g18980D	-0.798366139	0.307341752	0.521050737	0.2901498	-0.810966176	0.301520836	0.14404637	0.251692997	-0.209227962	0.256675121
BnaC09g21000D	0.729548123	0.343649829	-0.584962501	0.288164	0.473931188	0.291435503	0.232923089	0.267324052	-0.012383724	0.225774067
BnaC09g26970D	-6.36923381	0.539244954	-6.36923381	0.518077	-0.754523966	0.310613266	-2.866733469	0.435503188	-2.023458973	0.424026472
BnaC09g33190D	-3.115477217	0.266122001	-3.115477217	0.2627866	-0.056583528	0.21244364	-3.115477217	0.269941703	-0.893084796	0.245312885
BnaC09g34220D	0	0.22451918	3	0.2596164	0	0.21244364	0	0.226982722	0	0.223935767
BnaC09g34230D	0	0.22451918	2.222392421	0.24384	0	0.21244364	0	0.226982722	0	0.223935767
BnaC09g40700D	0.689659879	0.251006112	-0.047305715	0.2263112	0.367731785	0.228490789	0	0.226982722	-0.047305715	0.223935767
BnaC09g43200D	4.297680549	0.331045916	4.247927513	0.3211411	5.824428435	0.468004773	4.345774837	0.338317658	0	0.223935767
BnaC09g43890D	-1.475778584	0.690086829	0.822787158	0.546334	-0.20650645	0.32644246	-0.210123275	0.330279774	-0.2493731	0.370511025
BnaC09g47170D	0.472614775	0.418694301	0.913766538	0.5392031	-0.063649804	0.243137747	-0.349953989	0.365211878	-0.540995657	0.463205379
BnaC09g47250D	2.223811698	0.782232995	3.111895529	0.8224532	-0.761068743	0.485650252	-0.207324973	0.31112843	-0.133816304	0.289909824
BnaC09g50530D	0.215861423	0.326728663	2.032324938	0.7532121	-0.454823318	0.415405175	0.082317348	0.265164324	0.032218736	0.24215145
BnaCnng01350D	0.58496250 1	0.240608158	-2.584962501	0.2496566	-0.08246216	0.21244364	1.187627003	0.262348533	-0.08246216	0.223935767
BnaCnng01500D	4.297680549	0.331045916	2.321928095	0.244965	0	0.21244364	0	0.226982722	0	0.223935767
BnaCnng01510D	2.263498775	0.804415883	0.747057741	0.5178128	-0.210130285	0.322373983	0.994256017	0.616160749	0.037099018	0.248875004
BnaCnng01530D	0	0.22451918	0	0.2263112	0	0.21244364	0	0.226982722	0	0.223935767
BnaCnng06310D	2.938599455	0.260714978	3.36923381	0.2716061	3.807354922	0.28742867	0	0.226982722	4.169925001	0.3284789
BnaCnng09850D	-0.345842993	0.323008031	1.731557632	0.646714	0.273356877	0.304633837	0.668635788	0.430494646	1.248099683	0.627269805
BnaCnng18420D	0.794139303	0.296840325	-1.600392541	0.2940157	-0.773229138	0.260734792	0.103622631	0.236867713	-0.969626351	0.284152753
BnaCnng30350D	0	0.22451918	2.502500341	0.2479504	5.977279923	0.486207246	0	0.226982722	0	0.223935767
BnaCnng30500D	-8.732450113	0.815331202	-2.801712775	0.6308011	-0.459431619	0.368892881	0.978356321	0.548667618	0.160345653	0.294478055
BnaCnng36980D	0.318607737	0.267480362	0.842458723	0.3253527	0.517058436	0.284542428	-0.334419039	0.266797879	-0.888354644	0.316480415
BnaCnng38230D	0	0.22451918	0	0.2263112	0	0.21244364	0	0.226982722	0	0.223935767
BnaCnng43170D	0	0.22451918	0	0.2263112	0	0.21244364	0	0.226982722	0	0.223935767
BnaCnng44060D	0.376563351	0.250063845	-0.703606997	0.2504513	-0.623436649	0.241779386	-0.584962501	0.253953996	-1.510961919	0.277224453
BnaCnng48900D	0	0.22451918	0	0.2263112	0	0.21244364	0	0.226982722	0	0.223935767
BnaCnng49350D	4.48112669	0.343167688	6.709658248	0.5585856	2.874469118	0.244608387	0	0.226982722	3.807354922	0.303865494
BnaCnng49700D	-0.073284426	0.245678344	0.894131365	0.4611888	0.010171805	0.215010656	0.163863249	0.280057857	-0.224094717	0.300303375
BnaCnng60050D	0	0.22451918	0	0.2263112	0	0.21244364	0	0.226982722	0	0.223935767
BnaCnng60710D	4.544320516	0.348121191	3	0.2596164	0	0.21244364	5.309855263	0.42492911	4.564784619	0.359917133
BnaCnng63190D	0	0.22451918	0	0.2263112	4.142957954	0.308874036	0	0.226982722	0	0.223935767
BnaCnng64100D	1.723952267	0.659560041	1.019496176	0.4839573	0.623057803	0.404443623	0.740494406	0.437044278	0.770721193	0.477685019
BnaCnng69540D	0	0.22451918	3.502500341	0.2775591	0	0.21244364	0	0.226982722	0	0.223935767
BnaCnng72050D	3.502500341	0.281262109	3.502500341	0.2775591	0	0.21244364	0	0.226982722	3.502500341	0.284370707

BnWRKY

GeneID	log2(Cold/Control)	Prob.	log2(Heat/Control)	Prob.	log2(Drought/Contro	Prob.	log2(Salt/Control	Prob.	log2(ABA/Control)	Prob.
BnaA01g05060D	0.226439254	0.291763535	-1.162850476	0.4338762	-1.372445623	0.489518387	-0.995953167	0.438429057	-1.810397514	0.561894572
BnaA01g05500D	-5.938599455	0.486235866	-2.353636955	0.3628914	0.053866872	0.21990912	-5.938599455	0.49170013	-1.795641501	0.379640882
BnaA01g08990D	0.953457414	0.335468844	-0.249359469	0.2480384	0.99426363	0.334319631	-0.106915204	0.240247103	0.326500825	0.264105375
BnaA01g13440D	1.701593037	0.733699672	-0.23672179	0.3156768	-0.815153293	0.518173853	-1.709635102	0.669548505	-1.784209585	0.710200254
BnaA01g15760D	0.245641636	0.316830463	-1.235150309	0.5104772	-0.096288315	0.253399204	-0.266637701	0.31962644	-0.501177859	0.411083518
BnaA01g34790D	-0.439117954	0.389724013	-0.37208477	0.3528633	0.092903995	0.257284952	-0.398698762	0.377903854	-0.609154348	0.48016177
BnaA02g05730D	-2.807354922	0.256930501	-2.807354922	0.2544626	-2.807354922	0.244608387	-2.807354922	0.260578481	-2.807354922	0.258500211
BnaA02g10650D	-0.076217787	0.255975026	0.249578125	0.3156394	-0.097350599	0.25845618	-0.062565686	0.251259291	-0.240103872	0.334447321
BnaA02g13630D	0.1745516	0.295239794	-0.772873248	0.4421739	-0.339052273	0.345327416	-0.094074361	0.263656258	-0.153422371	0.295068072
BnaA02g14710D	1.881355504	0.291400278	-2.662965013	0.2496566	1.818161677	0.277312515	-2.662965013	0.255299148	0	0.223935767
BnaA02g21850D	1.479223247	0.508504614	-0.015375226	0.2280659	-1.701714747	0.400595301	-0.859256024	0.337846525	-0.202143737	0.262379355
BnaA02g24470D	0.896164189	0.264340942	1.399930607	0.2897117	0.852442812	0.2518383	-3.584962501	0.291867008	-1	0.253185653
BnaA02g31030D	-2.05621601	0.526759925	-1.843409637	0.4819582	-1.134749412	0.445960583	-1.340424439	0.470179911	-1.665264129	0.532422329
BnaA02g31540D	-0.05246742	0.227618972	0.872125177	0.3029518	0.321928095	0.245200606	-1.485426827	0.314481401	-1.597901556	0.324313555
BnaA02g34400D	4.392317423	0.338183363	3.321928095	0.2716061	3.36923381	0.262696379	6.470319935	0.556668076	0	0.223935767
BnaA02g35700D	2.415037499	0.246506129	0	0.2263112	2.321928095	0.234239054	2.321928095	0.249922946	0	0.223935767
BnaA02g36910D	1.366408538	0.587978618	1.806482588	0.6393916	0.620470602	0.401335024	0.940809534	0.489359875	0.556881625	0.408454859
BnaA03g04160D	-3.906890596	0.305333932	1.49683159	0.3072581	-0.032421478	0.21244364	0.983880335	0.283067385	0.934411658	0.280306633
BnaA03g05230D	0.338479271	0.275284881	1.415904243	0.432749	0.444218525	0.281455846	1.413302448	0.461600444	0.589579129	0.32487275
BnaA03g10980D	-1.250908632	0.441623516	0.812262695	0.4048487	-0.389672702	0.303933742	0.312899604	0.306408292	0.741194824	0.44068125
BnaA03g13820D	2.94394419	0.568263676	4.600275556	0.7595966	-0.965234582	0.262445401	0.227410496	0.249372556	-0.03562391	0.228581053
BnaA03g14060D	-0.389361365	0.387647945	-0.224254684	0.3144858	-0.250050685	0.333018511	-0.080361785	0.264433407	-0.326401571	0.391965198
BnaA03g17120D	-4.459431619	0.343167688	-4.459431619	0.3356031	-4.459431619	0.332505548	-2.722466024	0.305424196	-1.874469118	0.293432315
BnaA03g17130D	0	0.22451918	0	0.2263112	0	0.21244364	1.736965594	0.240291134	0	0.223935767
BnaA03g17820D	0.704462954	0.460640565	1.95894984	0.7030144	0.138368968	0.269041266	-0.193325434	0.294156628	-0.769468234	0.474387087
BnaA03g19180D	0	0.22451918	0	0.2263112	0	0.21244364	0	0.226982722	0	0.223935767
BnaA03g19190D	0	0.22451918	0	0.2263112	0	0.21244364	0	0.226982722	0	0.223935767
BnaA03g20910D	-3.459431619	0.281262109	-3.459431619	0.2775591	1.525461489	0.292945771	-3.459431619	0.285733471	1.525461489	0.310298443
BnaA03g21390D	0	0.22451918	0	0.2263112	3.700439718	0.28129293	2.807354922	0.260578481	0	0.223935767
BnaA03g25430D	1.127755547	0.437156557	-1.308631272	0.355721	0.240597017	0.256970129	0.637429921	0.339548329	-0.188072348	0.260146976
BnaA03g27660D	0.385431037	0.29944917	0.432959407	0.2958056	0.551516018	0.325698334	0.64096791	0.351364085	0.057450272	0.23802353
BnaA03g28720D	-0.42370236	0.326440259	0.63171076	0.3779919	-0.006493762	0.213982528	-0.317615102	0.307460636	-0.171815998	0.278452922
BnaA03g37950D	0.6604527	0.459293212	0.235954872	0.304876	0.022673501	0.221137589	-0.17178919	0.291323224	0.199212651	0.31460689
BnaA03g43640D	3.370601942	0.658818116	1.409723699	0.3605666	0.404390255	0.251294515	0.055495113	0.23086847	-0.891065628	0.285865564
BnaA03g46020D	-0.260764232	0.251941773	-0.901221846	0.2825654	-0.288244969	0.242358396	-5.403722186	0.433074871	-2.740757173	0.364824404
BnaA03g46280D	2.623851514	0.673575152	-1.407175382	0.3523878	0.219009782	0.251149213	-0.24423681	0.266044947	-0.24423681	0.265945877
BnaA03g46550D	0.063702841	0.245378932	0.587418152	0.4035982	-0.152822574	0.270137641	-0.474409162	0.369212107	-1.650322233	0.61551129
BnaA03g48030D	0	0.22451918	0	0.2263112	0	0.21244364	2	0.245020078	0	0.223935767
BnaA03g48160D	-0.483815777	0.277519462	-1.728928275	0.3428198	0.521808772	0.286468791	-0.065963262	0.236828085	-0.198413558	0.254290834
BnaA03g51140D	-0.637429921	0.327783208	1.238719681	0.4623248	0.595493168	0.344482018	-0.128416273	0.255327768	-0.746243408	0.357433777
BnaA03g51590D	0.337996464	0.295898059	1.263034406	0.4698012	0.800192396	0.394177763	-0.360922227	0.293709712	-0.400999666	0.304922241
BnaA03g59960D	0	0.22451918	3.584962501	0.2834218	1.874469118	0.229217302	2	0.245020078	1.874469118	0.242076597
BnaA04g00590D	0.4978017	0.296666402	2.679656911	0.6264464	0.070734512	0.224032636	-0.753981571	0.307799676	-0.581342185	0.297714344
BnaA04g01860D	0.49410907	0.334883229	0.384231452	0.298584	0.53207692	0.339059583	0.123271375	0.257582162	-0.486782107	0.328346807

BnaA04g02560D	2.997943414	0.831996988	-4.035788329	0.7236253	-2.069896183	0.666508155	-0.132497723	0.280467347	-1.186668265	0.601249604
BnaA04g13570D	0.870345681	0.541660062	1.834777618	0.7109818	0.040992571	0.231240533	-0.235819876	0.320088767	-1.137800558	0.603037268
BnaA04g14500D	0.256991083	0.33691967	-0.318721781	0.341052	-0.4425332	0.398446581	-0.949534933	0.541008401	-0.737160145	0.527330128
BnaA04g17420D	1.216745858	0.448036652	4.007377733	0.8034208	0.238404739	0.255307954	-0.231815675	0.265785163	0.174925683	0.259182694
BnaA04g17690D	-1.375654093	0.65318213	0.307627148	0.3564034	-0.745932031	0.526836979	-0.48000235	0.428751013	-0.751940765	0.562011254
BnaA04g20400D	-0.982926487	0.313283754	-5.807354922	0.4550222	-1.807354922	0.348301719	-1.461580085	0.34433011	-1.807354922	0.369489503
BnaA04g22040D	2.502009711	0.612939871	2.990587676	0.6493448	-1.284881108	0.326693438	-0.942775131	0.315417063	-1.059588796	0.332653052
BnaA04g23470D	-4.772589504	0.366913681	-4.772589504	0.3578168	-4.772589504	0.356718271	-4.772589504	0.372730195	-4.772589504	0.376655571
BnaA04g23480D	0.530442512	0.41934376	0.059756983	0.2469574	-0.450192164	0.376946176	-0.655164112	0.434257107	-0.799631822	0.503469654
BnaA04g25840D	0	0.22451918	0	0.2263112	0	0.21244364	0	0.226982722	0	0.223935767
BnaA04g26970D	0.844721775	0.382053788	2.097243862	0.5899028	-0.765044117	0.322321146	-0.188445089	0.262592906	-1.392317423	0.408276533
BnaA05g00620D	-1.014075185	0.287292173	-2.516575526	0.3160027	-0.599037686	0.256501198	0.324726728	0.260129363	0.493408563	0.27477412
BnaA05g01410D	1.213675204	0.401870002	0.701776166	0.305728	0.332319701	0.256408732	-0.245756414	0.258938321	1.004338936	0.385961552
BnaA05g01840D	-4.392317423	0.338183363	-0.094636874	0.2311811	0.289506617	0.234527458	0.546282033	0.26544172	-4.392317423	0.345725897
BnaA05g07220D	-2.321928095	0.246506129	-2.321928095	0.244965	0.847996907	0.232222428	-2.321928095	0.249922946	0	0.223935767
BnaA05g11870D	-0.685226624	0.319551587	1.445928807	0.4720755	0.731858704	0.354113829	-0.299168192	0.276370249	0.428988945	0.311700835
BnaA05g12160D	0.129096731	0.276370249	1.813859838	0.6969887	-0.217743062	0.300459685	0.179311604	0.298055585	-0.456291658	0.406345768
BnaA05g34110D	-0.634580433	0.311965022	1.395004234	0.462917	-0.708161566	0.313323382	-0.483374629	0.298233911	-0.327071398	0.28140521
BnaA05g34850D	1.908704166	0.375988499	3.03030276	0.4876823	0.972519264	0.274804942	-0.598637438	0.253953996	-0.11321061	0.231344006
BnaA06g08890D	1.047507666	0.581411374	2.056996277	0.7314277	-0.240845487	0.309294533	0.107681644	0.271729367	-0.085675115	0.266681197
BnaA06g16820D	-3.700439718	0.29336847	-3.700439718	0.2890842	0.884522783	0.255290341	-3.700439718	0.298240516	1.415037499	0.312841241
BnaA06g23750D	-0.516123624	0.345857991	-2.300394933	0.5173285	-2.446473193	0.561073391	0.038531238	0.235659058	-1.172639386	0.486130191
BnaA06g26680D	4.120093845	0.501816284	1.187627003	0.2549315	-2.584962501	0.239348868	0.91753784	0.251118391	2.67556505	0.352621174
BnaA06g28100D	-3.736965594	0.29336847	2	0.3415341	-3.736965594	0.28129293	2.053111336	0.366110113	2.044394119	0.376994611
BnaA06g29220D	-2.234169589	0.429446705	-1.306925932	0.3604455	-0.450661409	0.283670612	0.147976029	0.257379619	-0.450661409	0.300631407
BnaA06g33730D	1.345965337	0.602878756	0.930274591	0.4733964	1.008277103	0.533241308	0.454745331	0.364289425	0.196789565	0.297835429
BnaA06g36020D	-4.058893689	0.314587076	-4.058893689	0.3086671	1.286881148	0.30039584	-1.473931188	0.273629311	0.35614381	0.247972366
BnaA06g40630D	-4.297680549	0.331045916	0.560300446	0.2547906	-1.560714954	0.265813784	0.872244453	0.284099915	-0.673189684	0.255519303
BnaA07g07190D	-0.652076697	0.247672954	-3.874469118	0.2978024	-0.704544116	0.238873331	-0.067114196	0.23154655	-0.101879614	0.23117889
BnaA07g07600D	0.929610672	0.251417803	1.465663572	0.2676279	0	0.21244364	0.067114196	0.226982722	-2.807354922	0.258500211
BnaA07g16850D	1.317824227	0.677000775	0.309393416	0.3503668	-1.167714687	0.613926169	0.073977297	0.260034697	-0.404828614	0.421831519
BnaA07g20110D	4.142957954	0.320454225	2.662965013	0.2496566	0	0.21244364	0	0.226982722	0	0.223935767
BnaA07g24310D	0.236876668	0.267984519	-0.833057623	0.3150472	0.142856758	0.240579538	0.250465732	0.272493307	1.700027107	0.58178784
BnaA07g26870D	3.584962501	0.287446282	0	0.2263112	0	0.21244364	0	0.226982722	0	0.223935767
BnaA07g27840D	1.153709415	0.394100708	-1.400879436	0.3235518	0.469485283	0.277187027	-0.078951341	0.237884832	-0.632695112	0.294013526
BnaA07g28340D	-0.319538339	0.366380905	-1.208807741	0.5931104	-0.211463824	0.319135493	0.211747607	0.326024164	0.452526923	0.463117317
BnaA07g35260D	6.290791001	0.949762672	4.195475295	0.8383463	-0.117753531	0.240438638	-0.717600269	0.341732273	-0.612518223	0.340210997
BnaA08g08590D	1.733213459	0.39567262	3.043826241	0.5434565	1.559761947	0.366629681	2.192645078	0.459238173	0.20894689	0.244093223
BnaA08g10660D	1.921997488	0.294667389	2.818161677	0.3441056	0.881355504	0.235687678	0	0.226982722	-2.662965013	0.252892846
BnaA08g12420D	1.07091724	0.56453644	2.157966049	0.726298	-1.289643515	0.539920832	-0.576976517	0.396885678	-0.59547286	0.428753214
BnaA08g12810D	-0.441837559	0.340545634	0.960520807	0.4764059	-0.500731248	0.351515992	0.087566966	0.255272729	-0.406213649	0.350285322
BnaA08g14350D	-0.158570504	0.29447145	-1.028861004	0.5251198	0.138995024	0.281407411	0.154918366	0.294786273	-0.301853655	0.368256631
BnaA08g18040D	-4.938599455	0.383147962	-4.938599455	0.3727918	-4.938599455	0.373315809	-4.938599455	0.388896227	0.162938571	0.243476787
BnaA08g24160D	0.125530882	0.240645585	1.739348245	0.4224017	0.168599604	0.233459703	-0.492598483	0.269780989	-0.729038678	0.28444556
BnaA09g00120D	0.829859268	0.409447761	-0.168200321	0.2573092	1.064130337	0.473044137	-1.137503524	0.40599572	-0.341036918	0.299138751
BnaA09g00350D	0.214738849	0.25528814	-0.611929548	0.2766036	-0.22090893	0.241380905	-1.667424661	0.350765261	-1.667424661	0.360443306
BnaA09g05000D	2.409875794	0.471668164	-0.084888898	0.2308685	-0.485426827	0.241244408	0.245756414	0.246543556	-0.637429921	0.258729173
BnaA09g07010D	-3.584962501	0.287446282	-3.584962501	0.2834218	0.039528364	0.21244364	0	0.226982722	0.938599455	0.270696837
BnaA09g13370D	1.18422366	0.591527528	2.180073609	0.7276344	0.42722804	0.370768608	0.35347317	0.346190426	0.27158899	0.334748934
BnaA09g16360D	-0.078002512	0.22451918	-2.662965013	0.2496566	-2.662965013	0.239348868	-2.662965013	0.255299148	-2.662965013	0.252892846
BnaA09g17960D	-0.353636955	0.246972859	2.334419039	0.4338652	0.978938384	0.288809046	-0.860596943	0.267178749	0.061400545	0.229884374
BnaA09g24840D	-2.938599455	0.260714978	-2.938599455	0.257569	-2.938599455	0.248216739	0.935869663	0.257969636	-2.938599455	0.262328719
BnaA09g26610D	0	0.22451918	0	0.2263112	3.736965594	0.28129293	0	0.226982722	2.874469118	0.258500211
BnaA09g35840D	-0.509092843	0.410315175	-1.139701498	0.5369575	-0.492582521	0.407605058	0.462797015	0.409454366	0.365997724	0.40219143
BnaA09g44440D	2	0.241636285	0	0.2263112	0	0.21244364	0	0.226982722	0	0.223935767
BnaA09g45940D	0.29480874	0.34874203	0.499130178	0.4107511	-0.425718953	0.392753355	0.303132474	0.35359206	-0.093310211	0.27294903
BnaA09g54080D	-3.874469118	0.303103755	-3.874469118	0.2978024	-1	0.243164166	-0.935869663	0.257969636	-0.067114196	0.230366515
BnaA09g55250D	-0.184950686	0.263403079	-0.518988463	0.3019435	-0.695145418	0.332747719	-1.189342455	0.400093346	-0.860393933	0.374139191
BnaA10g10500D	-0.79400763	0.432275705	-0.521071367	0.356016	-0.269927372	0.304010796	0.203605777	0.296754465	0.024545498	0.232605499
BnaA10g18980D	0	0.22451918	0	0.2263112	0	0.21244364	2.736965594	0.258914104	0	0.223935767
BnaA10g20210D	3.969626351	0.308750749	4.459431619	0.3356031	0	0.21244364	0	0.226982722	0	0.223935767
BnaAnng00020D	-3.14974712	0.41800081	-3.275278002	0.4021452	-0.377157616	0.262152594	-0.351005328	0.27296444	-0.211147664	0.258812832
BnaAnng01980D	-1.71666818	0.460330145	1.383221052	0.5118818	0.358260308	0.300001761	-0.035946696	0.23380975	0.108758315	0.255523706
BnaAnng10540D	-0.057195402	0.234232449	1.002546682	0.3922712	0.217285531	0.254533006	0.025266759	0.230390732	-1.057195402	0.375953274
BnaAnng23990D	4.620671402	0.824472067	4.656347134	0.814455	0.913843356	0.32867704	0.520832163	0.28769726	-0.36705747	0.262249463
BnaAnng30430D	-0.970853654	0.25815897	-4.027497343	0.3056399	-0.029747343	0.21244364	-0.029747343	0.226982722	-0.970853654	0.259724277
BnaAnng32610D	2.451650073	0.687711349	1.59064241	0.5204437	0.029573115	0.217762602	0.432010578	0.311447656	-0.512136187	0.318149195
BnaAnng38320D	0	0.22451918	3.772589504	0.2922413	0	0.21244364	0	0.226982722	2.938599455	0.262328719
BnaAnng39080D	-2.807354922	0.256930501	-2.807354922	0.2544626	-2.807354922	0.244608387	-2.807354922	0.260578481	-2.807354922	0.258500211
BnaAnng39820D	0	0.22451918	4.584962501	0.3447176	4.544320516	0.337333562	0	0.226982722	4.459431619	0.351192363
BnaAnng41650D	-1.395928676	0.266540297	0.310340121	0.241797	-4.058893689	0.30296946	-1.473931188	0.273629311	-4.058893689	0.320225263
BnaC01g06660D	0.109016727	0.255528109	-2.745293954	0.5320415	-0.312334547	0.294026736	0.878375798	0.462016538	-0.088737369	0.25408609
BnaC01g06900D	-0.346560964	0.370246839	0.07812978	0.2599797	0.320476448	0.366803604	-0.005329862	0.230141956	-0.042702375	0.247391155
BnaC01g10570D	1.335184192	0.471269682	1.172389041	0.4160018	0.005538184	0.21244364	0.276840205	0.275489626	-0.097555309	0.245114157
BnaC01g13500D	2.807354922	0.312755381	1.398549376	0.248831	0	0.21244364	0.932885804	0.243873067	1.8259706	0.265430713
BnaC01g15640D	2.184655384	0.762890116	-0.056709442	0.2454076	-0.206047383	0.293553401	-1.821589558	0.615183258	-1.275468784	0.58815034
BnaC01g16120D	-0.854676972	0.41302309	0.252871076	0.2894057	-0.144183589	0.257258533	-0.27800925	0.302253954	-0.809316438	0.43296039
BnaC01g18490D	0	0.22451918	0	0.2263112	1.874469118	0.229217302	0	0.226982722	0	0.223935767
BnaC01g18810D	-0.059010644	0.249920744	-1.480495298	0.6031055	0.354125011	0.375369862	0.163799429	0.299775001	-0.057257318	0.257273944
BnaC02g01720D	0.944043594	0.364401705	3.649490502	0.7388315	0.470791481	0.280991317	1.12389647	0.399626616	0.395301281	0.284240815
BnaC02g09670D	1.483309521	0.625394079	-1.097297201	0.441498	-1.112286376	0.474902251	0.087872756	0.255272729	-1.488915813	0.5511884
BnaC02g14940D	-0.224215157	0.277961975	-2.40053793	0.4914381	-1.00547513	0.40787585	-0.935869663	0.400577689	-0.128514741	0.261945648
BnaC02g17970D	0	0.22451918	-2.736965594	0.2526947	-2.736965594	0.243100321	-2.736965594	0.258914104	-2.736965594	0.256833633
BnaC02g22950D	0	0.22451918	2.874469118	0.2544626	0	0.21244364	0	0.226982722	0	0.223935767
BnaC02g26030D	3.922861229	0.84901062	3.259721672	0.7866889	-0.002649578	0.21244364	-0.389672702	0.313149459	-0.062206423	0.243740974
BnaC02g27650D	-0.954833684	0.347742524	-1.444218525	0.3672109	0.596808744	0.326603174	-3.75234082	0.480122142	-3.822730148	0.504352478
BnaC02g32270D	-2.662965013	0.251618144	-2.662965013	0.2496566	0.881355504	0.235687678	-0.078002512	0.226982722	-2.662965013	0.252892846
BnaC02g34990D	2.350274489	0.733787735	-0.490433819	0.3229002	0.358272203	0.317361038	0.39674635	0.334059847	0.354723136	0.335195851
BnaC02g39170D	-0.744161096	0.260468403	2.425763906	0.4415817	0.592122292	0.253738244	-0.780686972	0.2635748	0.77940086	0.284863856
BnaC02g39360D	-1.563900885	0.391022931	0.416362897	0.2872107	0.684026628	0.341793917	-0.400962314	0.286937722	0.424289813	0.30779087
BnaC02g40180D	1.777607579	0.479184279	2.99138687	0.6279193	-0.485426827	0.260001673	0.950074775	0.350509881	-0.47175189	0.274432879
BnaC02g43320D	-0.572578776	0.250750731	-4.273018494	0.3211411	-1.5360529	0.265813784	-0.025090981	0.226982722	-4.273018494	0.334339445
BnaC03g05760D	-3.969626351	0.308750749	0.82045058	0.2636144	-3.969626351	0.297018652	-3.969626351	0.313622794	-3.969626351	0.31401247
BnaC03g06770D	-0.932885804	0.240672003	1.251538767	0.2613204	-0.932885804	0.22873296	-0.070389328	0.226982722	-0.932885804	0.241552626
BnaC03g13730D	-1.484121811	0.364648279	-0.530514717	0.2814911	0.804554478	0.347828384	0.933196629	0.379555021	0.492868211	0.310036458
BnaC03g16740D	2.428441751	0.586237187	3.64160608	0.7185882	-1.566346823	0.329625911	-0.485426827	0.276381257	-2.408805546	0.379717937
BnaC03g17030D	-0.009656225	0.22915786	-0.383995673	0.3819349	-0.088365372	0.263559389	-0.266105316	0.352205079	-0.051431877	0.255862746
BnaC03g20650D	0	0.22451918	0	0.2263112	2.584962501	0.239348868	0	0.226982722	0	0.223935767
BnaC03g21360D	1.403486206	0.685941298	2.151133254	0.7598079	0.29339319	0.350934781	0.097750822	0.272656222	-0.901749197	0.574685618
BnaC03g22830D	0.038474148	0.22451918	-3.624490865	0.2834218	-0.080170349	0.218539751	-3.624490865	0.291867008	0.518467089	0.248406073
BnaC03g22840D	-0.956931278	0.247939343	-3.459431619	0.2775591	-3.459431619	0.268900366	-3.459431619	0.285733471	-3.459431619	0.284370707
BnaC03g25800D	0	0.22451918	0	0.2263112	2.736965594	0.243100321	4.222392421	0.330838969	4.247927513	0.334339445
BnaC03g29850D	2.019718235	0.476705326	-0.885541275	0.2759211	0.624133098	0.277653757	1.465435352	0.39834531	0.43148802	0.271060094

BnaC03g32670D	0.918316202	0.499013702	0.806409203	0.4424315	-0.050507902	0.230060499	0.216234134	0.297113319	0.384100518	0.362578816
BnaC03g33830D	-1.695889651	0.573107101	0.113873777	0.2607238	-0.287193679	0.311667811	-0.348870575	0.334271197	-0.559153314	0.413866286
BnaC03g44980D	0.508417123	0.433231181	0.391436752	0.3725144	0.128311408	0.27689422	0.065296738	0.257520519	0.115706584	0.288212424
BnaC03g49470D	-2.68242831	0.399012382	-2.829269698	0.3835641	-1.261585189	0.338211984	-0.729734025	0.305338335	-0.855264907	0.322944186
BnaC03g60600D	-1	0.251897742	0.395928676	0.242026	-3.662965013	0.278871218	-1	0.255034961	-0.038474148	0.223935767
BnaC03g65200D	0.469485283	0.253518088	-0.893084796	0.2535291	0.691877705	0.254488975	-1.378511623	0.273567667	-1.452512205	0.273318891
BnaC03g67380D	-1.929276676	0.516181444	-3.654416835	0.5607498	-2.246758866	0.538798038	-1.737745219	0.50718368	-3.11937956	0.604452869
BnaC03g67520D	2.199245575	0.662965849	2.321143808	0.650767	-0.035721922	0.220651044	-0.097297201	0.248687872	0.293421667	0.294002519
BnaC03g67830D	-0.437961633	0.370138962	0.884234347	0.4998085	-0.393121067	0.355511818	0.033234282	0.236328331	0.133993425	0.285240322
BnaC04g00310D	0.684498174	0.304847388	-0.559427409	0.267848	0.500073603	0.27186146	0.795529487	0.321827997	0.237039197	0.255127426
BnaC04g01210D	1.451080249	0.569408486	1.196397213	0.4883912	0.014241923	0.215010656	0.088809267	0.250563599	-0.509674373	0.342546849
BnaC04g01540D	0.912537159	0.247939343	2.770518154	0.3303943	2.38827059	0.306399486	0.866733469	0.251118391	3.825426495	0.479921801
BnaC04g05170D	-1.079434467	0.29662017	-5.222392421	0.3967756	0.192645078	0.233862588	-5.222392421	0.415008894	-2	0.336160132
BnaC04g06800D	3.686420486	0.810551622	4.414328859	0.8505099	-0.703018262	0.32811124	-0.106915204	0.249988992	-0.084888898	0.245301877
BnaC04g08020D	-3.115477217	0.266122001	-3.115477217	0.2627866	1.276840205	0.261822361	1.299560282	0.280238385	0.584962501	0.245143365
BnaC04g13870D	-1.347151511	0.567431488	0.252142516	0.3100321	0.086963226	0.252170735	0.54897685	0.427154884	0.115114266	0.280095283
BnaC04g14500D	1.117039421	0.410365811	3.838937567	0.7733299	-1.449307401	0.353757177	0.373814837	0.284262831	-0.006789166	0.223935767
BnaC04g21660D	0.578267848	0.329870284	1.431845787	0.4712477	-0.448825813	0.283670612	-0.931520262	0.349056853	-0.369025102	0.289130473
BnaC04g23420D	0.634482637	0.321442724	0.514573173	0.2911493	0.682359264	0.322305735	-0.881355504	0.320652365	-0.313366081	0.270476681
BnaC04g35770D	1.21447855	0.579172391	1.901196988	0.6751493	0.227344726	0.292239071	-0.07782714	0.252542798	-0.711929043	0.434970411
BnaC04g36310D	0.447295859	0.397099229	-0.013108052	0.2299196	-0.111879726	0.262289091	-0.419120986	0.375055039	-0.40501404	0.396242823
BnaC04g38910D	0.638023744	0.40315351	2.687267263	0.7509643	-0.831461539	0.40428511	1.763331971	0.666111874	0.923151921	0.515071859
BnaC04g40940D	5	0.388874212	5.662965013	0.4402453	3.938599455	0.293320036	3.058893689	0.26634656	0	0.223935767
BnaC04g41050D	1.995001794	0.667049738	3.567753211	0.8067407	0.133350387	0.24909516	0.734682848	0.405178943	0.496786689	0.364058262
BnaC04g41330D	-1.570951301	0.721410582	0.216431661	0.3309336	-0.551942022	0.488485857	-0.272169982	0.362464335	-0.739514129	0.593462697
BnaC04g47350D	0.522476767	0.39151608	-0.943868125	0.4296228	-1.055351233	0.485302406	-0.369294963	0.334614639	-1.02815827	0.509862975
BnaC04g49810D	0	0.22451918	0	0.2263112	4.700439718	0.351890257	0	0.226982722	3.169925001	0.270666015
BnaC04g51410D	0	0.22451918	0	0.2263112	0	0.21244364	0	0.226982722	0	0.223935767
BnaC05g10200D	0.992586763	0.518671404	1.589337622	0.6212992	-0.800797206	0.421673007	0.13929495	0.274283173	-0.383614435	0.347262584
BnaC05g20030D	0	0.22451918	0	0.2263112	0	0.21244364	0	0.226982722	0	0.223935767
BnaC05g22280D	-0.061400545	0.22451918	-0.125530882	0.2317997	-0.061400545	0.21244364	1.415037499	0.280407904	1.772589504	0.304574395
BnaC05g22810D	0	0.22451918	2.807354922	0.2544626	0	0.21244364	3.736965594	0.298240516	0	0.223935767
BnaC05g23680D	0.265999864	0.284080101	-3.491853096	0.493556	-0.190102883	0.255891366	-2.574315257	0.492649001	-1.606495662	0.466712459
BnaC05g47140D	-1.950417971	0.344233242	1.092794096	0.3504681	0.884522783	0.325676318	0.249519599	0.258938321	-0.516015147	0.275298091
BnaC05g48590D	-0.044394119	0.227088397	0.479167837	0.2541015	-1.137503524	0.261611011	0.415037499	0.256113724	-0.552541023	0.255061379
BnaC06g13990D	0	0.22451918	3.700439718	0.2890842	0	0.21244364	0	0.226982722	0	0.223935767
BnaC06g15910D	1.270761628	0.642339462	-0.010279592	0.2296026	-1.073544485	0.551183997	0.630716944	0.463738156	-0.609597385	0.467207809
BnaC06g19560D	0.144389909	0.230756191	1.479992941	0.2646756	-2.662965013	0.239348868	0.074000581	0.226982722	-2.662965013	0.252892846
BnaC06g25390D	-2.736965594	0.255312357	0.963474124	0.2481441	0.925999419	0.239205766	0	0.226982722	2.419538892	0.341192892
BnaC06g30480D	1.169925001	0.259143066	-2.736965594	0.2526947	2.137503524	0.302082233	0.887525271	0.254513192	-2.736965594	0.256833633
BnaC06g30870D	-2.321928095	0.246506129	3.263034406	0.3600845	2.378511623	0.296730248	0	0.226982722	-0.447458977	0.232277467
BnaC06g40170D	0.931895554	0.417923756	1.894363498	0.5759185	1.047037456	0.447891349	-0.759311753	0.341747684	-1.519928615	0.43672285
BnaC07g08810D	-4.772589504	0.366913681	-4.772589504	0.3578168	-1.602664502	0.283994241	-2.450661409	0.313495104	-4.772589504	0.376655571
BnaC07g09430D	0	0.22451918	2.736965594	0.2526947	0	0.21244364	2.736965594	0.258914104	0	0.223935767
BnaC07g19090D	0	0.22451918	-0.160464672	0.23213	0.144389909	0.219651538	-0.078002512	0.226982722	1.752072487	0.28952235
BnaC07g22240D	1.19064291	0.546512293	2.363577527	0.714348	0.53722989	0.373474321	0.223002054	0.291404681	0.12268252	0.265932668
BnaC07g27240D	1.661246086	0.511412871	2.50326814	0.6141815	-0.448052557	0.272590176	-1.328470941	0.359749815	-0.136850868	0.250004403
BnaC07g27510D	-5.874469118	0.480199197	-1	0.3056422	-0.745186101	0.289401265	-1.459431619	0.346670365	-0.540568381	0.286098929
BnaC07g27590D	1.321928095	0.255801103	-0.106915204	0.2263112	-2.222392421	0.233252756	-2.222392421	0.248806756	0.777607579	0.241512998
BnaC07g30300D	3.683452364	0.68361205	-0.690671942	0.2631059	-2.353636955	0.309512487	-0.294743266	0.252538395	-2.275634443	0.323538607
BnaC07g35130D	3.689299161	0.73461552	2.44625623	0.5443548	-0.175086707	0.233613812	0.402098444	0.27433601	-0.485426827	0.272136655
BnaC07g38350D	-4.169925001	0.322490665	-1.506959989	0.2677379	0.394859617	0.237957484	-0.026967048	0.226982722	0.704544116	0.269094103
BnaC07g38550D	1.561402711	0.562753179	0.127067462	0.2513584	-1.461277678	0.421573937	-0.572105808	0.326587763	-0.451153461	0.316310895
BnaC07g38840D	-0.340221253	0.353360897	-0.929747804	0.4890671	-0.664886045	0.458353147	-0.110685798	0.273629311	-0.447066353	0.418799975
BnaC07g40660D	-0.797183117	0.35868206	-1.489515715	0.4090515	0.123828587	0.244412448	-0.12429411	0.256503399	-0.821029859	0.38009
BnaC07g42990D	-0.210094049	0.289727095	0.776161531	0.4379733	-0.186576242	0.275973969	-0.405086033	0.340233013	-0.378196315	0.348926961
BnaC07g43320D	0.839145989	0.499557487	1.325053154	0.595629	0.03330359	0.224398094	-0.120398868	0.270721054	-0.535872424	0.413073726
BnaC07g43490D	1.908584397	0.745211614	1.162136199	0.589687	-0.613444268	0.436617176	-0.392963046	0.36718007	-1.24346572	0.612470939
BnaC07g47230D	-4.321928095	0.333071348	-4.321928095	0.3260616	-4.321928095	0.321880834	-4.321928095	0.338317658	-4.321928095	0.340140547
BnaC07g49530D	0.549687026	0.265109285	2.453717967	0.4482348	-4.523561956	0.337333562	2.679378103	0.510386946	0.549687026	0.267431928
BnaC08g11240D	5.209453366	0.409434552	5.68182404	0.440952	4.169925001	0.310820212	4.584962501	0.358510339	0	0.223935767
BnaC08g12240D	-0.496241532	0.446724524	-0.984227212	0.5674733	-0.123998399	0.281550513	0.222356459	0.33628342	-0.173106665	0.327246028
BnaC08g18410D	2	0.241636285	1.874469118	0.2401326	0	0.21244364	0	0.226982722	0	0.223935767
BnaC08g27340D	-0.195088438	0.327164571	-1.044346651	0.5942002	-1.095770152	0.648237434	0.191244046	0.325392317	0.138055791	0.313957431
BnaC08g29410D	6.14974712	0.51271179	6.215937399	0.4997556	6.05166212	0.494689845	3.36923381	0.279293917	0	0.223935767
BnaC08g32160D	-2.807354922	0.256930501	-2.807354922	0.2544626	-2.807354922	0.244608387	-2.807354922	0.260578481	-2.807354922	0.258500211
BnaC08g40010D	-0.110644064	0.272310578	0.641142534	0.4581638	0.28862612	0.346820071	0.233587509	0.326070397	0.062181995	0.257769294
BnaC09g03790D	2.274362412	0.3748591	1.477047162	0.294969	1.909487707	0.333232062	-3.624490865	0.291867008	1.490986353	0.313180281
BnaC09g04560D	0.379889556	0.328192698	-0.214572917	0.2738363	-0.651436779	0.366706735	-0.109329757	0.258579467	0.497219606	0.381487988
BnaC09g06700D	0	0.22451918	0	0.2263112	0	0.21244364	0	0.226982722	0	0.223935767
BnaC09g13680D	1.60319434	0.663582285	2.41666613	0.7459734	0.664793524	0.440923421	0.943126745	0.519309856	0.475668329	0.405559812
BnaC09g17450D	-1.98550043	0.376637958	-3.541893779	0.4003773	0.028854863	0.215717355	-0.325575872	0.268708831	-1.652076697	0.38058535
BnaC09g19320D	3.270089163	0.480870672	3.56358885	0.4937432	-1.765534746	0.247382349	2.355480655	0.380578745	0.719892081	0.255624978
BnaC09g22830D	0	0.22451918	0	0.2263112	3.415037499	0.266500669	0	0.226982722	0	0.223935767
BnaC09g36400D	-4.984893108	0.387562084	-4.984893108	0.3767987	-4.984893108	0.377943482	-4.984893108	0.393279527	-0.380031049	0.254068477
BnaC09g44020D	0	0.22451918	4.392317423	0.3309579	0	0.21244364	0	0.226982722	0	0.223935767
BnaC09g53550D	0.024005463	0.231738085	0.030340029	0.2360047	0.421076416	0.389497252	0.584424483	0.446513174	0.152245136	0.298266934
BnaCnng01360D	-0.425793896	0.366226796	0.647869252	0.433535	0.196908587	0.295167142	0.132379792	0.278968086	-1.053211806	0.556229966
BnaCnng02000D	0.339310173	0.346701187	0.289208509	0.3180589	-0.4223423	0.359492233	-0.575159326	0.404487654	-1.116023796	0.559970411
BnaCnng05780D	0	0.22451918	0	0.2263112	0	0.21244364	0	0.226982722	0	0.223935767
BnaCnng11190D	0	0.22451918	0	0.2263112	0	0.21244364	0	0.226982722	0	0.223935767
BnaCnng22580D	-3.115477217	0.266122001	-3.115477217	0.2627866	-0.893084796	0.232222428	-3.115477217	0.269941703	1.206450877	0.273114146
BnaCnng26190D	2.057638626	0.459524376	0.359081093	0.2531306	0.911066272	0.299143154	1.068895081	0.333377364	0.605721061	0.282836222
BnaCnng39610D	0.652649308	0.3297514	2.236275361	0.5787365	-0.968839069	0.324229895	0.932285681	0.382177076	-0.044787922	0.231324192
BnaCnng39880D	0	0.22451918	0	0.2263112	0	0.21244364	0	0.226982722	0	0.223935767
BnaCnng41380D	-0.125530882	0.230485399	-3	0.2596164	-0.061400545	0.21244364	-0.061400545	0.226982722	2.115477217	0.333379566
BnaCnng45760D	1.373598225	0.435939096	0.339357901	0.2649948	-1.771925433	0.355265244	-2.455451768	0.385571876	-0.214443668	0.25455282
BnaCnng52600D	2.597359046	0.804902427	-0.741171705	0.4322119	-2.663565135	0.689448378	-0.837594535	0.479096217	-1.410544518	0.622104953
BnaCnng60780D	0	0.22451918	0	0.2263112	3.841302254	0.28742867	0	0.226982722	0	0.223935767
BnaCnng66020D	2.301026318	0.650298091	2.437709326	0.6402018	-0.597367522	0.303169802	-1.319459839	0.385109549	-1.682029919	0.42874661
BnaCnng68630D	-0.381562795	0.311011748	-0.940293754	0.3815012	-0.097179069	0.240786484	-0.152567639	0.267812797	-0.086352038	0.251827292
BnaCnng74680D	0	0.22451918	0	0.2263112	2.938599455	0.248216739	0	0.226982722	0	0.223935767
BnaCnng75330D	-6.513069582	0.555639949	-2.425606741	0.4078274	-0.574470127	0.296243704	-6.513069582	0.560936895	-2.370111628	0.453331396
BnaCnng77260D	0	0.22451918	4.790076931	0.3605402	0	0.21244364	0	0.226982722	0	0.223935767
