# Supplementary material for: Exploring transcription factors reveals crucial members and regulatory networks involved in different abiotic stresses in Brassica napus L
Source: BMC Plant Biol. 2018 Sep 19;18:202. doi: 10.1186/s12870-018-1417-z (PMC6146658; doi:10.1186/s12870-018-1417-z)
Supplement: Supplementary file 5 — The finally identified 315 crucial DEGs. (PDF 128 kb) [file 12870_2018_1417_MOESM5_ESM.pdf]

Additional file 5 The 315 crucial DEGs

Family	GeneID	Cold	Probability	Heat	Probability	Drought	Probability	ABA	Probability	Salt	Probability
AP2/EREBPs	BnaA01g00710D	1.77053102	0.730952129	1.752480589	0.7003747	0.649428116	0.480742981	0.33899283	0.38592633	0.83867897	0.53262707
AP2/EREBPs	BnaA01g02720D	0	0.22451918	7.21916852	0.62068495	0	0.21244364	0	0.22393577	0	0.22698272
AP2/EREBPs	BnaA01g13420D	-0.7901267	0.443818468	-2.810945206	0.62583219	-0.434693596	0.357942337	-0.9587605	0.51508287	-1.879658	0.60447929
AP2/EREBPs	BnaA01g27570D	-0.162402	0.29647927	1.658222345	0.7000907	0.257538418	0.336367079	0.4533348	0.44920568	0.39779255	0.394519
AP2/EREBPs	BnaA01g28970D	2.4335943	0.736658565	1.944444852	0.64271813	0.265481726	0.288637324	2.15597788	0.73881389	1.44443489	0.58876457
AP2/EREBPs	BnaA01g31290D	2.21723072	0.314604688	6.303780748	0.7869113	-2.736965594	0.243100321	0.88752527	0.25263526	2.66675659	0.35602698
AP2/EREBPs	BnaA02g04480D	7.37213954	0.664275776	5.392317423	0.41369677	0	0.21244364	0	0.22393577	0	0.22698272
AP2/EREBPs	BnaA02g18720D	0.00675627	0.229069798	1.737572622	0.73269356	-0.427703087	0.42764363	-0.1808836	0.33367678	-0.4733345	0.4376453
AP2/EREBPs	BnaA02g32120D	0.516791	0.259339005	5.182814513	0.79844746	-0.04508789	0.217157174	-0.9779737	0.27069684	-0.0223678	0.22698272
AP2/EREBPs	BnaA02g34370D	7.35251641	0.917450421	6.375660079	0.8334346	1.280107919	0.265435116	-0.9004643	0.24531289	0.05062607	0.22698272
AP2/EREBPs	BnaA03g04290D	2.34221549	0.74966096	3.094089163	0.79350717	0.535743873	0.385239441	0.63024338	0.44264504	1.26452711	0.58219513
AP2/EREBPs	BnaA03g33790D	1.34691074	0.692462309	1.364787905	0.66608105	0.043390066	0.235069041	-0.0041034	0.22537338	-0.2711843	0.34993527
AP2/EREBPs	BnaA03g34290D	1.43976459	0.377095882	-0.145197916	0.2384022	-1.062735755	0.274426274	2.99037558	0.62921158	0.53287399	0.27509775
AP2/EREBPs	BnaA03g55280D	-1.725825	0.267037849	5.163386392	0.74771699	-0.381870635	0.228512804	-0.9668331	0.25615996	-0.3388019	0.24356485
AP2/EREBPs	BnaA04g13590D	2.67430999	0.811828525	1.10086168	0.57306307	0.637074533	0.470725897	-0.0033204	0.22466448	0.20667196	0.31061106
AP2/EREBPs	BnaA04g29310D	1.66953183	0.699969619	4.061209901	0.86611672	-1.695871814	0.611385572	-1.2442467	0.58634286	0.41884509	0.37807558
AP2/EREBPs	BnaA05g23130D	-0.0405382	0.231625806	2.451813191	0.6766067	-0.165389647	0.250235567	0.15816769	0.26496398	-0.3337872	0.29055268
AP2/EREBPs	BnaA05g24390D	3.57054573	0.851476364	1.233557429	0.57794392	1.688863367	0.703214713	-0.3053368	0.34270096	0.36893158	0.3554832
AP2/EREBPs	BnaA05g25200D	1.2481246	0.685564831	0.207683988	0.32290236	0.087812675	0.265970094	0.29753492	0.40212758	0.17761471	0.31935345
AP2/EREBPs	BnaA06g40170D	0.01312743	0.226716334	2.109785303	0.66171096	0.723185155	0.408945806	-0.2593467	0.29407737	-0.4371855	0.32195128
AP2/EREBPs	BnaA07g08440D	0.59371706	0.409309063	2.2178471	0.71910555	0.358949764	0.335970799	-0.908441	0.47645655	-0.8882228	0.44367096
AP2/EREBPs	BnaA07g12040D	1.90137253	0.782561027	-0.758550319	0.52527387	0.522826754	0.481528937	0.13276923	0.30960275	0.30311439	0.37734466
AP2/EREBPs	BnaA07g13990D	1.5050464	0.727183064	-2.764426956	0.76853711	0.164092297	0.303343725	-0.0040355	0.22546365	0.26164847	0.35398834
AP2/EREBPs	BnaA07g23090D	1.88383772	0.760569675	0.950071419	0.56100074	-2.106768611	0.719149583	-0.6845662	0.52382525	-1.2787473	0.62295255
AP2/EREBPs	BnaA07g23650D	-3.1874656	0.643992832	1.993447248	0.6942874	-0.171015062	0.272794921	-0.1841604	0.29276084	0.24156679	0.30686401
AP2/EREBPs	BnaA07g33640D	-7.8621207	0.72375524	-0.854626189	0.38542437	-3.359620385	0.603876061	-0.2327641	0.29628553	-4.803227	0.6532856
AP2/EREBPs	BnaA08g01300D	2.9173159	0.768504086	3.068159894	0.76183557	0.79262015	0.414544366	0.47634372	0.35419309	-0.1493227	0.26477905
AP2/EREBPs	BnaA08g04090D	1.59549005	0.698049861	-0.092880967	0.25839674	-0.218827217	0.303235848	-0.2490524	0.33437467	0.45662512	0.39921272
AP2/EREBPs	BnaA08g11220D	2.87446912	0.256930501	7.544320516	0.66112535	0	0.21244364	2.87446912	0.25850021	0	0.22698272
AP2/EREBPs	BnaA08g13860D	3.57399138	0.458099968	7.502379789	0.90286511	3.2410081	0.414110659	0.8259706	0.25243933	3.10281081	0.40770413
AP2/EREBPs	BnaA08g19490D	-0.1104735	0.275623921	0.309496275	0.35215444	-0.47102838	0.422522808	-0.5788733	0.49593152	-1.7574505	0.69411788
AP2/EREBPs	BnaA09g30360D	-0.1600404	0.260508031	0.691534165	0.3567535	-2	0.462985223	-7.0588937	0.65091673	-7.0588937	0.62955502
AP2/EREBPs	BnaA09g30810D	0.20737118	0.326136444	2.819387478	0.81812057	-0.636088003	0.494198897	0.17868242	0.32956207	-0.3189967	0.36960398
AP2/EREBPs	BnaA09g47030D	0	0.22451918	7.303780748	0.63121279	0	0.21244364	0	0.22393577	0	0.22698272
AP2/EREBPs	BnaA10g00620D	-0.7689085	0.392442936	-1.235084835	0.43790509	0.461153783	0.350736641	1.62306711	0.67634031	0.74745797	0.43372213
AP2/EREBPs	BnaA10g20950D	-3.5066777	0.625590017	-1.901537361	0.51774675	-0.998213381	0.452428758	-1.4666003	0.54547976	-1.0454001	0.4591325
AP2/EREBPs	BnaA10g25000D	1.2014074	0.592877083	5.028684036	0.90564127	-0.303170531	0.315329001	0.05242092	0.24634101	0.62772482	0.43359224
AP2/EREBPs	BnaAnng01320D	1.23583268	0.46539813	6.343285285	0.9421717	-0.353360603	0.270518511	0.48542683	0.32524481	1.40880555	0.50108317
AP2/EREBPs	BnaAnng03730D	1.48367196	0.732114551	2.246126938	0.78784697	0.56435507	0.501805277	0.5997155	0.55519523	1.3440144	0.71348057
AP2/EREBPs	BnaAnng06940D	-1.6153819	0.629689316	0.546959612	0.42005266	-1.038493518	0.552670048	-0.4033788	0.40131961	-3.9090423	0.76157579
AP2/EREBPs	BnaAnng13220D	0.47293588	0.273008472	3.542898441	0.67081	-0.835613182	0.272603385	-2.2730185	0.34628289	-5.2730185	0.42132956
AP2/EREBPs	BnaAnng20420D	-0.0331669	0.2266679	0.431339312	0.26547915	3.107890723	0.66356027	0.72379021	0.31771769	-0.0443941	0.22944186
AP2/EREBPs	BnaAnng21280D	0.22899153	0.295169344	2.081021009	0.68928326	-0.256751364	0.28915469	-0.483946	0.36640292	-1.1666877	0.47499031
AP2/EREBPs	BnaAnng23490D	-0.6485276	0.25421378	4.527881406	0.70807355	-0.648527629	0.241852038	-0.0766213	0.23070335	-4.2730185	0.33281377
AP2/EREBPs	BnaAnng28960D	-8.5091152	0.793604037	0.432421553	0.35257494	-1.586283045	0.568230653	-0.2083813	0.30471089	-5.4502215	0.7406654
AP2/EREBPs	BnaAnng35170D	3.40759269	0.811498292	1.973593269	0.63287937	1.047305715	0.490784283	1.32644711	0.58651899	1.93933722	0.6641723
AP2/EREBPs	BnaAnng36290D	0	0.22451918	8.215937399	0.74138751	0	0.21244364	3.32192809	0.27751066	3.4150375	0.28318847
AP2/EREBPs	BnaAnng40580D	2.05556055	0.785902991	1.12659757	0.62013896	-0.580168605	0.472561996	0.48303662	0.47918648	-0.0462627	0.24951566
AP2/EREBPs	BnaC01g01700D	1.40330266	0.685239001	2.170528867	0.76128738	0.654298773	0.498058227	-0.0087238	0.22875057	0.71417304	0.50901538
AP2/EREBPs	BnaC01g10100D	0.17992797	0.272409648	1.951279185	0.63294542	-0.140792344	0.250301613	-1.1347686	0.45383775	-0.886841	0.39214132
AP2/EREBPs	BnaC01g35070D	0.81802737	0.41903334	1.726731537	0.58633846	2.303818714	0.710363169	0.37550914	0.32775239	0.45424157	0.33315941
AP2/EREBPs	BnaC01g36330D	2.14337484	0.677749304	1.238091108	0.48636576	1.782335147	0.630409225	0.74137847	0.42358616	0.38795132	0.31697577
AP2/EREBPs	BnaC02g40810D	3.54432052	0.285033376	8.156504486	0.73451205	5.437405312	0.422300451	3.50250034	0.28437071	0	0.22698272
AP2/EREBPs	BnaC02g43290D	3.76387229	0.834491352	2.032228333	0.64043512	0.062365881	0.229362605	-1.9265789	0.51681549	-0.0501088	0.23795969
AP2/EREBPs	BnaC02g48800D	-1.7288619	0.642715929	1.52791999	0.66590713	-2.119546207	0.683975307	-0.3541948	0.38167732	-2.2636082	0.69043908
AP2/EREBPs	BnaC03g05820D	1.99919917	0.75592439	2.832974286	0.80407024	0.47405928	0.409973934	0.36016625	0.39165918	1.06381702	0.59486949
AP2/EREBPs	BnaC03g38390D	0.58736608	0.493375515	1.55110889	0.70691993	0.0287964	0.230252034	0.11653663	0.3006204	0.29949194	0.37346331
AP2/EREBPs	BnaC03g39000D	1.5556785	0.720926239	2.001398534	0.7505592	-0.065618379	0.250002202	0.31649323	0.3963551	-0.057083	0.25419397
AP2/EREBPs	BnaC03g49530D	3.44959223	0.673823928	2.740332445	0.54823831	-5.156504486	0.395014354	-0.4935395	0.26367387	-0.2657336	0.25239089
AP2/EREBPs	BnaC03g66140D	0	0.22451918	7.306821202	0.63121279	3.841302254	0.28742867	2.87446912	0.25850021	2.93859946	0.26431672
AP2/EREBPs	BnaC03g69420D	2.78751011	0.733800944	3.984146049	0.82138989	1.177787119	0.484859893	1.41651491	0.56497235	1.22745156	0.49322581
AP2/EREBPs	BnaC04g35800D	2.83135618	0.793584223	1.316568231	0.55989336	1.026504635	0.532125119	-0.5281389	0.38112033	-0.6293288	0.38179841
AP2/EREBPs	BnaC04g53380D	6.89481776	0.604060992	8.244760234	0.74469645	0	0.21244364	2.73696559	0.25683363	3.66296501	0.29545114
AP2/EREBPs	BnaC05g00680D	-0.9641033	0.526914034	-0.670264491	0.43347996	0.34081617	0.362972894	1.2718611	0.69280355	0.96819794	0.57732749
AP2/EREBPs	BnaC05g17200D	-4.9654345	0.711133714	0.239284835	0.29598392	-0.987460804	0.474327645	0.45062728	0.39761659	-8.4248661	0.7908719
AP2/EREBPs	BnaC05g17550D	0.95777176	0.547736359	1.763236016	0.68464018	-0.490986353	0.383892088	0.19392896	0.30989996	-0.8042755	0.46049526
AP2/EREBPs	BnaC05g18050D	2.19155039	0.590294656	3.740812317	0.7603165	-0.516575526	0.276709289	-6.101538	0.52617211	-0.4770472	0.28410872
AP2/EREBPs	BnaC05g38410D	4.05234124	0.874315316	2.569933219	0.7678194	0.194026896	0.287067614	-0.2993712	0.3376748	-1.0627904	0.49926468
AP2/EREBPs	BnaC05g42130D	1.42211613	0.602141234	4.290062625	0.86610791	0.057561958	0.232365529	-0.0316654	0.23438656	0.19044118	0.2853438
AP2/EREBPs	BnaC06g38250D	0	0.22451918	7.125843933	0.6091686	0	0.21244364	0	0.22393577	0	0.22698272
AP2/EREBPs	BnaC07g16160D	1.49303038	0.73263632	-1.116429177	0.62151934	0.556475334	0.500039628	0.05360163	0.25890089	0.07863424	0.27033138
AP2/EREBPs	BnaC07g31340D	-1.3438887	0.635574078	0.550363839	0.44169617	-1.259619741	0.634647223	-1.5153059	0.70133679	-1.5387048	0.6691236
AP2/EREBPs	BnaC07g45030D	0	0.22451918	7.142957954	0.61050495	0	0.21244364	0	0.22393577	0	0.22698272
AP2/EREBPs	BnaC08g12880D	1.77146844	0.486363556	3.766750444	0.73553357	0.630191755	0.29705828	-1.4119777	0.34513808	-2.9023033	0.38055453
AP2/EREBPs	BnaC08g15650D	3.36923381	0.275018493	7.815916936	0.69389332	0	0.21244364	0	0.22393577	0	0.22698272
AP2/EREBPs	BnaC08g20020D	-4.018859	0.6638861	-0.444481277	0.33252536	-1.812830052	0.567072634	-0.3125902	0.33016309	-5.0443941	0.70525776
AP2/EREBPs	BnaC08g21640D	0.35216667	0.396302265	1.658620437	0.72500132	1.105820619	0.669920568	0.28578228	0.39834971	1.20672098	0.68393788
AP2/EREBPs	BnaC08g36830D	0	0.22451918	7.459431619	0.65041697	3.36923381	0.262696379	4.34577484	0.34014055	0	0.22698272
AP2/EREBPs	BnaC09g04110D	0	0.22451918	8.236811481	0.7442055	6.215937399	0.515446123	0	0.22393577	5.05889369	0.3988913
AP2/EREBPs	BnaC09g06610D	4.88702529	0.723052943	0.621488377	0.25022676	-1.478047297	0.247023495	-3.7004397	0.29728724	-0.5849625	0.24737795
AP2/EREBPs	BnaC09g20100D	5.12722055	0.660090616	2.935459748	0.35735012	1.807354922	0.280934077	1.82781902	0.29725642	0.92599942	0.25451319
AP2/EREBPs	BnaC09g27360D	0.28795204	0.332826975	-0.398046783	0.34294313	-0.254414094	0.309014935	-0.7846623	0.49322581	-2.2294472	0.65102901
AP2/EREBPs	BnaC09g28190D	-2.4789209	0.566568477	3.585471837	0.82485073	-0.827360614	0.406121209	0.27386144	0.31483145	-3.7396851	0.61959298
AP2/EREBPs	BnaC09g49920D	0.94049638	0.583637148	3.909963219	0.8677833	-0.916160407	0.550930818	-0.2425424	0.3484206	0.28575836	0.35362729
AP2/EREBPs	BnaCnng04580D	-0.5991414	0.473898341	-0.587048364	0.45100655	-0.735042855	0.529210257	-0.2793523	0.37913673	-1.8100832	0.71982986
AP2/EREBPs	BnaCnng08620D	-1.6770527	0.573606855	-2.668924812	0.61003382	0.10445521	0.252844412	-1.5977638	0.60509793	-1.4687012	0.55402621
AP2/EREBPs	BnaCnng31550D	4.27301849	0.327631301	9.321928095	0.8533631	0	0.21244364	0	0.22393577	3.36923381	0.27929392
AP2/EREBPs	BnaCnng33070D	2.02577691	0.78882886	1.381788978	0.67651203	1.509688307	0.732576878	-0.2638149	0.37448484	-0.1546932	0.30362773
AP2/EREBPs	BnaCnng36390D	0.15544812	0.296283332	-0.107372723	0.26915355	-0.873676428	0.526009194	-2.0870169	0.72804167	-1.5491355	0.64382992
AP2/EREBPs	BnaCnng67070D	0.1095271	0.27193191	1.502108846	0.66436164	0.359238988	0.373104459	-0.514193	0.44557531	-0.3722037	0.36798364

AP2/EREBPs	BnaCnng69830D	0.83650127	0.266971802	4.315041709	0.63322502	0.855610091	0.25511862	0.85561009	0.26970393	-3.8073549	0.30425957
AP2/EREBPs	BnaCnng75020D	3.53003629	0.777977386	5.287526641	0.89274895	-3.442943496	0.451182676	-6.4429435	0.57004253	-1.2334901	0.36913505
AP2/EREBPs	BnaCnng77210D	-1.7425038	0.430362552	-3.398549376	0.46848251	0.502500341	0.319153105	2.09621532	0.67953917	0.54857835	0.33857524
BnbZIPs	BnaC09g09560D	-2.781563	0.611328331	0.259777757	0.29699884	-1.296935267	0.508114939	0.08985276	0.2594777	-1.8097967	0.55936498
BnbZIPs	BnaC07g27220D	-2.7343356	0.832760929	1.473392691	0.70724356	-0.896473265	0.621642626	-0.7216666	0.60519259	-0.4779959	0.46324281
BnbZIPs	BnaA06g29500D	-2.1642459	0.801419564	1.828795733	0.75379108	-0.530497474	0.494397038	-0.9782825	0.67941148	-0.6231487	0.51887615
BnbZIPs	BnaCnng01910D	-1.85619	0.762051323	1.257114879	0.66818794	-0.367618003	0.414302195	0.03529361	0.25214872	-0.4666609	0.45125313
BnbZIPs	BnaA09g03330D	-1.3672771	0.651222745	2.648605534	0.80709738	-0.391730126	0.397953433	-1.7776592	0.74083272	-0.3020929	0.36094526
BnbZIPs	BnaA06g29270D	-1.2738983	0.694553788	-0.085149669	0.27303269	-0.240238284	0.353713146	-0.5623848	0.54200571	-0.326456	0.39555153
BnbZIPs	BnaC07g27440D	-1.2110928	0.680855701	0.01078792	0.23221362	-0.297207395	0.383407746	-0.2607566	0.38972842	0.05118745	0.25351148
BnbZIPs	BnaA02g00920D	-1.1905544	0.671774279	-0.938095439	0.5841303	-0.755371789	0.570669978	-0.32594	0.42543327	-0.8678514	0.59200967
BnbZIPs	BnaC01g04330D	1.26265826	0.643173853	1.424129062	0.644224	0.277627996	0.335616348	1.6926655	0.75583413	1.52532688	0.69872794
BnbZIPs	BnaA01g27940D	2.25067206	0.7697964	-3.4325419	0.6689783	1.050266056	0.583874916	0.59190522	0.47028118	0.63757904	0.45077759
BnbZIPs	BnaC08g20170D	3.79236221	0.75431285	2.682809824	0.59025283	0.670935724	0.300378227	0.28345395	0.26305743	-0.8728881	0.30140856
BnbZIPs	BnaA08g11080D	-0.6499272	0.43013359	-8.997179481	0.82420348	-0.160077215	0.279474444	-1.1104668	0.57428273	-3.0352475	0.69870372
BnbZIPs	BnaA02g02830D	-0.476206	0.387179013	-5.663558104	0.77090158	0.360196249	0.361616735	0.42390474	0.41288219	0.15502407	0.29125938
BnbZIPs	BnaC02g06270D	-0.9837166	0.517634471	-4.925017214	0.75653202	0.303801477	0.341818134	0.38128023	0.39970807	-0.0256082	0.23596728
BnbZIPs	BnaA03g34610D	0.82511799	0.581517049	-3.42170123	0.82051807	0.170529272	0.312814823	-0.0957308	0.29044921	-0.1714746	0.31684587
BnbZIPs	BnaA05g22650D	0.50593802	0.417965585	-3.145886435	0.68429233	-0.006702428	0.21572396	0.38056766	0.39865133	-0.1030625	0.26799332
BnbZIPs	BnaC07g45120D	0.95549362	0.570148209	-2.292063454	0.657517	-0.437240671	0.387912131	-0.1004084	0.27510656	-2.1166752	0.68110448
BnbZIPs	BnaA04g05810D	-0.4492114	0.38923967	-2.200207568	0.65198668	0.749751749	0.517467153	0.06487467	0.25676759	0.49821298	0.42254923
BnbZIPs	BnaCnng20200D	-0.5965026	0.500209148	-2.047363392	0.75094887	-0.426577632	0.440894801	-0.2016957	0.35211041	-0.5089519	0.46663761
BnbZIPs	BnaA03g52920D	0.80454272	0.539075434	-1.670126477	0.63812128	-0.398824455	0.389618338	0.50104615	0.47424839	-0.5021642	0.42289047
BnbZIPs	BnaC09g45380D	-0.7231878	0.515895241	-1.255865282	0.61719548	0.212681815	0.326057188	-0.4164127	0.44742682	0.12734612	0.29318354
BnbZIPs	BnaA04g23630D	-0.1116342	0.274668446	1.357077699	0.65379636	-0.6321467	0.473502061	-0.0409198	0.2471754	0.08081394	0.26431232
BnbZIPs	BnaC05g51490D	-0.5567361	0.483649037	1.376611148	0.68662158	-0.243936534	0.348394184	-0.2578008	0.38442927	0.03940465	0.24636963
BnbZIPs	BnaA09g39870D	0.3068571	0.382987249	1.424848005	0.69321744	-0.391683288	0.421298742	-0.7392677	0.59602751	-0.5924919	0.49587208
BnbZIPs	BnaC08g32220D	0.41763663	0.436725052	1.50113071	0.71025529	-0.297544329	0.384420462	-0.3003988	0.41337974	-0.627488	0.52013324
BnbZIPs	BnaA05g01520D	-0.9132723	0.489124309	1.626022705	0.66772562	0.458120356	0.396009458	1.8992452	0.76752219	1.15823712	0.60942839
BnbZIPs	BnaA01g31420D	-0.3287581	0.375303815	1.669798899	0.71880174	-0.106420594	0.273974955	-0.0222195	0.23690954	0.08618327	0.27190769
BnbZIPs	BnaA09g00170D	0.06822524	0.251563105	1.707409273	0.68610862	1.188819313	0.630664606	0.20860342	0.32267339	0.2929956	0.33899794
BnbZIPs	BnaAnng39310D	0.25173176	0.305177622	1.81400277	0.66073347	0.815831281	0.478836433	0.01758431	0.23113486	0.64224694	0.42354433
BnbZIPs	BnaC04g33670D	-0.1036744	0.269252615	1.865346065	0.72792719	-0.064695768	0.24390609	0.21744002	0.34043556	0.12971554	0.2870478
BnbZIPs	BnaC04g01070D	-0.6700647	0.473180633	2.136223172	0.75950192	0.421929471	0.407552221	1.84085354	0.78400745	1.15881723	0.64398182
BnbZIPs	BnaA05g08020D	-0.6981633	0.440927824	2.168218679	0.74049588	0.267981979	0.323276621	0.68798984	0.51161541	0.97142126	0.55818935
BnbZIPs	BnaAnng26550D	0.6600117	0.459984501	2.233727491	0.74568495	0.577403084	0.439560657	1.8552995	0.76221864	1.5859456	0.69394396
BnbZIPs	BnaA08g04620D	0.97028515	0.617829529	2.251944224	0.78382912	0.248103405	0.349510374	0.39393984	0.44884682	0.4600594	0.44141877
BnbZIPs	BnaC06g18530D	-0.2415749	0.349175737	2.338754102	0.79551939	-0.191641174	0.323353676	-0.1182542	0.30374441	-0.5896123	0.49616048
BnbZIPs	BnaA07g19330D	0.81789488	0.533538518	2.728835549	0.80159569	-0.075345125	0.251261492	0.29304228	0.36987698	-0.1038739	0.27084434
BnbZIPs	BnaC07g44670D	0.72694368	0.486574906	2.913232122	0.80523046	0.686333036	0.482347916	2.69701347	0.83138496	1.99134881	0.75377127
BnbZIPs	BnaC04g56840D	2.11425718	0.465994751	2.849757364	0.54055488	3.02358682	0.600245694	1.4229427	0.38852857	0.78682611	0.29764389
BnbZIPs	BnaC01g43800D	0.23927692	0.281576931	1.406174223	0.51690576	1.545880335	0.584414298	1.81787391	0.6650111	0.57744565	0.36032442
BnbZIPs	BnaA01g02570D	-1.0415423	0.533360192	-0.639531443	0.41842571	0.0818933	0.250697894	1.74646298	0.76058509	0.67106588	0.47721829
BnbZIPs	BnaC01g03810D	-0.0916602	0.259231128	-1.486877501	0.55778206	0.064681841	0.240121614	1.33958147	0.69071207	-0.0274459	0.23596728
BnbZIPs	BnaA01g26200D	0.54829837	0.436249516	0.76231419	0.48209033	-0.107092967	0.262667759	1.23869547	0.68154259	0.24715705	0.32732969
BnMYBs	BnaC04g50810D	-6.946419	0.610282592	-6.94641896	0.58609188	-1.94641896	0.447721829	-6.946419	0.63607823	-6.946419	0.61525371
BnMYBs	BnaA03g05370D	-2.4940335	0.624887721	-1.244913875	0.48636576	-0.536186986	0.379367889	0.24933819	0.32443024	-1.1466499	0.50686445
BnMYBs	BnaA07g31770D	-2.4047736	0.78102434	-3.354454683	0.80588432	-0.56253971	0.483853781	-0.8279977	0.60863142	-0.7208693	0.52948765
BnMYBs	BnaC08g48630D	-2.2845868	0.802802142	0.787882525	0.55425297	-0.742387267	0.569608827	-0.5407873	0.53120487	-2.9587281	0.83751629
BnMYBs	BnaA03g40690D	-2.1472592	0.734846684	1.805107855	0.73199787	-1.063133818	0.608025996	-0.5082687	0.4770972	-3.5978126	0.80611548
BnMYBs	BnaC02g16640D	-2.1194466	0.773831854	1.39479932	0.68659736	-0.155195347	0.306034027	0.5465612	0.5306875	-1.2189084	0.6702508
BnMYBs	BnaA06g18160D	-2.0614481	0.624284494	-2.047816235	0.5920581	-3.650983332	0.703238931	-1.1878093	0.57118734	-1.9772116	0.62328939
BnMYBs	BnaA06g12860D	-2.0048767	0.654254289	-0.643754767	0.41665346	-0.157603695	0.282186762	-0.2902281	0.35260796	-0.2186108	0.31155773
BnMYBs	BnaC03g03620D	-1.9144743	0.686951812	1.001326372	0.57214502	-0.444711123	0.402873912	-0.5119973	0.45858871	-0.094313	0.26803515
BnMYBs	BnaCnng78680D	-1.8461449	0.702926309	-1.514976104	0.6340462	-0.090184813	0.261153087	-0.7306677	0.54896483	-0.9995972	0.57831158
BnMYBs	BnaAnng03830D	-1.7207326	0.672604266	1.228791482	0.6269814	-0.269579107	0.337936789	-0.5564234	0.478788	-4.3260151	0.80739679
BnMYBs	BnaA01g00670D	-1.594192	0.736786255	0.184572523	0.31607093	-0.371226942	0.416409085	0.27294327	0.39561538	-0.8090157	0.57594271
BnMYBs	BnaC07g31640D	-1.5735892	0.722964881	-0.004288208	0.2278017	-1.220728221	0.680193033	-0.1623802	0.32736491	-2.9141164	0.8226866
BnMYBs	BnaC07g13600D	-1.5432916	0.656262109	1.955988088	0.74240243	0.047198444	0.238617951	0.99976246	0.65122935	0.74360087	0.52427437
BnMYBs	BnaC01g01660D	-1.5011769	0.726185759	0.179001803	0.31627787	-0.635042804	0.533575945	0.43312251	0.48281905	-0.5874257	0.50285542
BnMYBs	BnaC05g43350D	-1.4864982	0.70803172	-3.393616124	0.81712987	0.346543394	0.403278999	-0.0177962	0.23771091	-0.03017	0.24112112
BnMYBs	BnaA01g32200D	-1.43444	0.66292402	-3.378786973	0.77881398	0.605239164	0.500123287	0.17359132	0.32459976	0.17267561	0.31060446
BnMYBs	BnaA06g12480D	-1.3846677	0.71173694	-0.79559364	0.55295185	0.122448132	0.288410564	-0.300188	0.41353825	0.26631011	0.36895232
BnMYBs	BnaC09g35120D	-1.371367	0.63372477	-1.859202361	0.66398737	-0.033880893	0.228466572	-0.1171835	0.29107665	-0.285533	0.35043283
BnMYBs	BnaA08g30200D	-1.2474081	0.623850787	0.427453196	0.39863151	-0.11727946	0.275718588	-0.1682115	0.31865115	0.2914324	0.35823955
BnMYBs	BnaA05g28870D	-1.2213626	0.656713428	-3.770736331	0.82263597	0.206052478	0.330253355	0.03340098	0.24454454	0.07819769	0.26904127
BnMYBs	BnaA07g10350D	-1.1425504	0.634235531	1.497672883	0.69673113	0.323736749	0.382705449	0.77994943	0.60686798	0.74082457	0.54573514
BnMYBs	BnaC09g40660D	1.06459967	0.648173588	-3.325639106	0.81601589	0.525585308	0.485414685	0.90302879	0.65081326	0.52949793	0.47650719
BnMYBs	BnaC07g45320D	1.12699767	0.619714062	0.630300457	0.4492365	0.147321988	0.283714643	0.1344215	0.29237337	0.76354125	0.51401511
BnMYBs	BnaA05g01050D	1.31025632	0.708892529	-2.979046626	0.81661251	0.604208038	0.523299077	0.42728384	0.48323735	0.50759123	0.47533376
BnMYBs	BnaC02g07210D	1.44256433	0.687257829	-2.172370248	0.66621975	-0.507088583	0.419971204	0.31727858	0.38464282	0.66418608	0.48655949
BnMYBs	BnaAnng35540D	1.45641343	0.604571753	0.837827274	0.431558	-0.157541277	0.260858079	-0.0788088	0.25151687	-0.616395	0.36955115
BnMYBs	BnaC05g00840D	1.47846625	0.737761545	-5.297680549	0.9109096	0.361668569	0.417157614	0.65113299	0.58282037	0.34312281	0.40204172
BnMYBs	BnaA10g00430D	1.48531649	0.637225246	-2.259948426	0.5608092	0.8374895	0.488281112	1.28563984	0.64079837	0.92062382	0.50712644
BnMYBs	BnaC06g12130D	1.51838124	0.73164562	-1.052732243	0.59166402	-0.567626478	0.486731216	0.8090562	0.61879161	0.44833977	0.4376475
BnMYBs	BnaC01g00270D	1.55407546	0.608085438	0.663218941	0.3779765	-0.835984389	0.391892546	-0.235317	0.29194186	0.17474202	0.27625136
BnMYBs	BnaC04g00590D	1.57180917	0.745660731	-2.724399155	0.79396289	0.549499568	0.497646536	0.54914754	0.53406689	0.55695991	0.49178159
BnMYBs	BnaC05g00500D	1.61029828	0.601879249	-1.253975067	0.40867722	1	0.477649794	1.46252643	0.61303674	0.95089453	0.46149697
BnMYBs	BnaCnng76050D	1.77046985	0.632738473	-2.227068909	0.47883203	0.526846755	0.351623868	0.43451487	0.34648984	0.86655273	0.43980723
BnMYBs	BnaA10g28420D	2.47312012	0.791470728	0.710218683	0.45247059	0.198319645	0.295876044	0.43833299	0.4145906	0.1792338	0.29678308
BnMYBs	BnaA03g29470D	2.49463018	0.611706999	1.694654789	0.45770149	1.123792486	0.388640847	0.41712081	0.28496072	0.41712081	0.28231445
BnMYBs	BnaC01g03510D	2.5483275	0.768387404	0.426391997	0.33638469	0.352605693	0.325266829	0.80917766	0.49598876	0.35688457	0.33307355
BnMYBs	BnaA03g06490D	2.62066166	0.823263412	-3.188557853	0.75275195	0.420646612	0.413412766	0.83139675	0.60205317	0.76732271	0.53636091
BnMYBs	BnaA03g53110D	2.68005538	0.780515781	0.777758797	0.42728478	0.494471967	0.369780108	0.10665594	0.26416262	0.78725372	0.45586319
BnMYBs	BnaA02g15320D	-2.3297476	0.596564691	-8.268347055	0.74738895	-0.520154205	0.364192557	-3.9225722	0.69476514	-1.5912147	0.54413461
BnMYBs	BnaC05g04250D	0.98383518	0.498555779	-7.902877533	0.70434852	-0.156923156	0.261776128	-0.7430062	0.41773882	-0.0448965	0.23640318
BnMYBs	BnaCnng65380D	0.6351147	0.377179541	-7.351675438	0.63666605	0.052046748	0.226421325	-0.1717663	0.27000555	-1.135738	0.42172805
BnMYBs	BnaAnng17890D	0.58222753	0.431826588	-5.874469118	0.75918049	0.73470962	0.488248089	0.71736486	0.51677586	0.56085388	0.42619721
BnMYBs	BnaA03g00640D	-0.4686791	0.424396333	-5.812787444	0.8632701	0.298972746	0.365022544	0.23805757	0.36019673	-0.0722703	0.26106503
BnMYBs	BnaA10g00780D	0.69173262	0.547919088	-5.173428841	0.91107912	0.566940748	0.515010215	0.53706196	0.53869897	0.39506546	0.43045282
BnMYBs	BnaC09g41280D	-1.527247	0.545994928	-4.919564426	0.68443763	0.265602518	0.309155835	-0.1228567	0.27227976	-0.8496803	0.44419053
BnMYBs	BnaA10g04120D	-0.0009501	0.22451918	-4.614709844	0.72693649	-0.325732171	0.336761157	-0.4357639	0.3984796	-0.6219999	0.42320089
BnMYBs	BnaA10g17370D	0.32192809	0.390769752	-3.711944864	0.84849325	0.339879345	0.404743034	0.4049221	0.467756	0.19894458	0.33457721

BnMYBs	BnaA08g22480D	-0.7660141	0.438864965	-3.533179959	0.6551173	0.597687567	0.433057258	-0.0278264	0.23674663	0.72999894	0.47051675
BnMYBs	BnaC05g41910D	-0.7110373	0.520547131	-3.385275505	0.80047069	-0.399724564	0.414174504	-1.2613603	0.70030205	-0.7267917	0.52758551
BnMYBs	BnaA10g12770D	-0.918247	0.57262496	-3.046571115	0.77523425	0.20387683	0.321660678	-0.5045805	0.48291152	-0.6068817	0.48135061
BnMYBs	BnaA10g17890D	-0.3698855	0.347947268	-2.646725668	0.63547721	0.771775686	0.499051129	0.0987015	0.27030716	0.2975392	0.33632305
BnMYBs	BnaC03g60080D	-0.2903723	0.342652524	-2.582567986	0.69595398	0.391175779	0.389809874	0.47933714	0.45751876	-0.0549548	0.24769717
BnMYBs	BnaA10g24770D	-0.5008674	0.398307883	-2.468857014	0.65451187	-0.306489364	0.336833809	0.01543873	0.2326055	-0.4025653	0.36899856
BnMYBs	BnaA08g16990D	-0.3457598	0.35630658	-2.230353137	0.65279466	0.551622193	0.445434411	1.05522274	0.64558015	0.06973621	0.25660247
BnMYBs	BnaC03g55560D	-0.8952899	0.580240146	-2.070135801	0.73305902	0.735189657	0.5608026	-0.3907336	0.44367977	0.46227165	0.44251295
BnMYBs	BnaA10g26900D	-0.0702897	0.25906381	-1.922417059	0.7033314	0.249784148	0.345831572	-0.048046	0.25579009	0.31003706	0.37383097
BnMYBs	BnaC03g11590D	0.32828079	0.361695991	-1.888544303	0.63790553	-0.031539091	0.226905668	-0.0218108	0.23469918	0.2410081	0.32836002
BnMYBs	BnaA10g18810D	-0.9608294	0.531083782	-1.80934165	0.62706066	-0.710734053	0.477667406	0.24416087	0.34522174	-0.5470806	0.4254817
BnMYBs	BnaA03g09270D	-0.7804205	0.521764592	-1.686036653	0.66396536	0.362982419	0.39222278	-0.0998804	0.28341963	-0.1195037	0.28542525
BnMYBs	BnaC08g31190D	-0.5569227	0.438990454	-1.483311438	0.60830119	-1.397154794	0.638988693	-1.2301755	0.64735241	-1.6361368	0.66493184
BnMYBs	BnaC05g14070D	-0.8455265	0.590701944	-1.41105954	0.68748459	0.313358886	0.391258498	-0.2034585	0.35601818	0.36434704	0.41067183
BnMYBs	BnaA01g30920D	0.83420708	0.561533517	-1.384861618	0.62449584	1.000711739	0.62676785	-0.3472439	0.40909992	0.69887814	0.52172496
BnMYBs	BnaC05g17910D	-1.1192649	0.529060552	1.311628746	0.60657517	0.510026757	0.413494223	1.28999993	0.67597925	0.71448985	0.47773345
BnMYBs	BnaA07g11930D	-0.7773828	0.518620769	1.532631037	0.69014847	-0.038261558	0.233842774	0.64026465	0.54308667	-0.966565	0.57188304
BnMYBs	BnaC02g46900D	1.03752112	0.523248441	1.546439429	0.60368453	0.259409542	0.29714414	0.69471944	0.46171052	0.74418811	0.44371059
BnMYBs	BnaA03g42350D	1.05272725	0.597139297	1.556739775	0.67277158	-0.072469638	0.246768114	0.48503081	0.45410855	0.1183527	0.27928951
BnMYBs	BnaCnng59970D	-0.0408451	0.233547765	1.670054438	0.60130684	0.030472352	0.220510145	0.50291833	0.37911251	-0.1261771	0.26293855
BnMYBs	BnaA06g05610D	-1.1379421	0.4499432	1.673542678	0.60936234	0.080999722	0.238820494	0.29805212	0.32019884	0.33849594	0.32030232
BnMYBs	BnaA09g30490D	-0.8475926	0.469730794	1.68204723	0.6734981	0.058448781	0.238562912	1.58293909	0.72580049	0.92264326	0.53906222
BnMYBs	BnaA07g29070D	0.05196999	0.246616207	1.735014688	0.71696564	-0.422483684	0.400896914	-1.0645524	0.62728962	-0.5176268	0.4329802
BnMYBs	BnaA03g24010D	-1.3214227	0.556659269	1.739793617	0.68251347	-0.235595636	0.304644845	-0.713898	0.47568601	-0.5057898	0.39043512
BnMYBs	BnaC03g36160D	-0.224754	0.296071982	1.831224874	0.67345627	0.460332298	0.37480186	-0.1679796	0.28887729	0.43437451	0.36622459
BnMYBs	BnaA03g01420D	0.43173554	0.404756244	2.249370577	0.7675288	0.133579346	0.278496953	0.17631782	0.31998089	0.27307641	0.34350453
BnMYBs	BnaA03g30840D	0.11070346	0.247399961	2.337772373	0.63945983	0.759796303	0.372001479	-0.3375009	0.28939686	0.21732588	0.27245588
BnMYBs	BnaAnng01040D	0.70731153	0.460065959	2.483649014	0.75968905	-0.532450664	0.382474286	0.4439724	0.40156178	0.01484776	0.23014196
BnMYBs	BnaC03g28550D	-1.0885367	0.414755715	2.582575118	0.71678731	0.200139614	0.266269506	-0.8531586	0.4018634	-0.6965092	0.36058421
BnMYBs	BnaA07g24010D	0.47148025	0.413738596	2.622295009	0.79417424	0.020482848	0.22187511	-0.721302	0.5121614	-0.2304	0.32003373
BnMYBs	BnaC03g01730D	-0.6372488	0.476496178	2.799122438	0.81329476	-0.357513798	0.381624485	-0.1295378	0.29851131	0.02143958	0.23520994
BnMYBs	BnaC02g02590D	0.5474878	0.414934041	2.80359087	0.78933742	-1.693022247	0.596820071	-0.0011445	0.22393577	0.21140009	0.30385669
BnMYBs	BnaC04g51450D	-1.0356239	0.278439713	3.624015277	0.634612	-1.965234582	0.291523565	-0.474909	0.25761739	-1.8981204	0.3049993
BnMYBs	BnaC04g27380D	-1.7784422	0.392751154	4.33905658	0.82799456	0.44823755	0.290361143	-0.8294262	0.33576385	1.2089797	0.44573823
BnMYBs	BnaA05g00710D	0	0.22451918	7.569855608	0.66398737	4.662965013	0.34721415	4.34577484	0.34014055	4.84130225	0.38019347
BnMYBs	BnaA02g03510D	0.78526115	0.418022826	-1.50622583	0.43234616	1.881554812	0.659379513	0.68294706	0.41925129	0.64061556	0.38370936
BnMYBs	BnaA01g31080D	0.6436085	0.329165786	-3.163746427	0.40850329	2.323165929	0.633843654	-0.4379214	0.28800548	1.31073954	0.4574439
BnMYBs	BnaC06g35350D	0	0.22451918	0	0.22631125	7.50779464	0.686564338	3.70043972	0.29728724	2.80735492	0.26057848
BnMYBs	BnaA03g26820D	0	0.22451918	0	0.22631125	7.930737338	0.739144123	6.36923381	0.56085324	6.45395649	0.55474391
BnMYBs	BnaA08g22580D	0.49717702	0.351947497	-0.902702799	0.37441879	0.106915204	0.244302371	1.35206515	0.6019541	-0.2906541	0.29532786
BnMYBs	BnaC09g42570D	0.95659904	0.484556078	-1.182034684	0.42862552	0.491365782	0.357233435	1.40789407	0.63089797	0.05379799	0.23971653
BnMYBs	BnaC09g05650D	0.25381588	0.301109144	0.161528286	0.26939131	0.27978356	0.302962855	1.44954035	0.65623569	0.27523486	0.31059565
BnMYBs	BnaCnng62710D	0.6204706	0.39913787	0.511925465	0.35200694	0.468467509	0.355751788	1.54994891	0.6654382	1.17060312	0.55044868
BnMYBs	BnaA07g31680D	0.79883885	0.423581757	-3.106499621	0.51920638	0.740203968	0.411026278	1.6258046	0.65196687	-0.0805564	0.24794595
BnMYBs	BnaA02g33410D	-0.1847399	0.252351263	1.945218714	0.52067482	0.921612101	0.364234387	2.1867809	0.62775635	0.54273427	0.30959174
BnMYBs	BnaA01g21090D	-0.8860867	0.433068266	1.09024912	0.51553419	-1.048209093	0.468022385	-0.9273547	0.47102311	-3.4493074	0.63576121
BnNACs	BnaC06g43410D	-2.3582108	0.648464194	1.828724145	0.69252175	1.626170321	0.700132534	0.86649549	0.56128914	0.62275242	0.44387571
BnNACs	BnaC09g43890D	-1.4757786	0.690086829	0.822787158	0.54633397	-0.20650645	0.32644246	-0.2493731	0.37051103	-0.2101233	0.33027977
BnNACs	BnaA07g24270D	-1.3091731	0.702373719	1.622145701	0.72887166	-0.042400417	0.23856071	0.54330015	0.53671537	0.68571702	0.54696802
BnNACs	BnaA10g20110D	-1.0784117	0.604124837	2.083163997	0.76364085	-0.098699086	0.268046162	-0.0431499	0.24918102	0.38442168	0.40048522
BnNACs	BnaC03g03740D	1.27968107	0.662045599	2.747364543	0.80547703	0.010011031	0.217033886	-0.652954	0.50449338	0.48702053	0.42870478
BnNACs	BnaC05g20530D	1.28298995	0.606438673	1.082083689	0.53158133	-0.016441147	0.218742295	0.22763753	0.31567685	-0.1658438	0.28416156
BnNACs	BnaA03g48570D	1.3802574	0.697679999	3.25412559	0.83934579	0.254810622	0.34295854	1.96049792	0.80346041	1.05286673	0.63085614
BnNACs	BnaC02g42720D	1.42599531	0.713978125	2.831033673	0.8215484	0.492241947	0.460360967	0.48257178	0.48987504	1.19423097	0.67542226
BnNACs	BnaC07g40860D	1.5176806	0.733184508	2.714279057	0.81579353	0.283142995	0.366464564	1.83553054	0.80145039	0.92129625	0.60779703
BnNACs	BnaA02g33910D	1.5190266	0.713352883	2.410019425	0.78645778	0.48626754	0.441845873	0.8488017	0.60642987	1.33652547	0.68595671
BnNACs	BnaC09g09210D	1.55855853	0.675662228	-1.39273205	0.52515059	-0.411724765	0.355359911	-0.4779289	0.40170268	0.15597746	0.28765763
BnNACs	BnaC02g00990D	1.63255058	0.750697894	2.37318192	0.79574835	-1.18705323	0.668425711	-0.5337087	0.51158679	-0.4819294	0.44808509
BnNACs	BnaC05g00370D	1.64405416	0.75772086	1.578646266	0.72173641	0.333247739	0.39864252	0.13106376	0.31211253	0.55191995	0.49082391
BnNACs	BnaCnng64100D	1.72395227	0.659560041	1.019496176	0.48395725	0.623057803	0.404443623	0.77072119	0.47768502	0.74049441	0.43704428
BnNACs	BnaAnng05950D	1.78686419	0.769899873	2.456595562	0.80125445	0.141090068	0.295653686	-0.4953908	0.49758709	-0.1667349	0.31537523
BnNACs	BnaA10g22680D	1.90947303	0.73321533	3.010673502	0.80944424	0.201545137	0.295510585	-0.5437255	0.42626986	-0.2956414	0.32958188
BnNACs	BnaC07g13550D	1.99149762	0.717874881	2.160647188	0.71270563	-0.386548942	0.331389359	-0.1188207	0.27021249	-0.3905845	0.33735558
BnNACs	BnaC09g47250D	2.2238117	0.782232995	3.111895529	0.82245324	-0.761068743	0.485650252	-0.1338163	0.28990982	-0.207325	0.31112843
BnNACs	BnaA01g16400D	2.50571788	0.805397777	2.696600388	0.79777158	-0.217836354	0.308748547	0.74996482	0.55279554	0.3338137	0.36017472
BnNACs	BnaC06g05920D	2.7767906	0.658071788	5.390823176	0.88634903	0.470791481	0.280991317	3.09114789	0.73072097	1.99410743	0.54773856
BnNACs	BnaA07g10300D	3.45037646	0.764882525	3.501813016	0.75227641	0.154920577	0.241173958	0.92199749	0.40513271	0.57910977	0.32465039
BnNACs	BnaC03g39160D	7.66296501	0.700007045	4.841302254	0.3646725	2.662965013	0.239348868	0	0.22393577	0	0.22698272
BnNACs	BnaC03g71470D	0.20776481	0.321233576	1.114790899	0.60550081	0.626456243	0.496310191	0.26748753	0.36886646	0.6988137	0.51139306
BnNACs	BnaA01g31650D	-0.3848122	0.38714599	1.25953746	0.64160414	-0.164797784	0.295636074	0.07291127	0.26687934	0.5195366	0.4507886
BnNACs	BnaC03g50570D	0.49492527	0.432920762	1.498805857	0.67683126	1.026390747	0.621389447	-0.3760743	0.40954463	1.21490566	0.65725501
BnNACs	BnaA09g04810D	-0.0087821	0.227337173	1.538164726	0.65066575	-0.313924555	0.330649635	-0.2101223	0.31497235	-0.4695601	0.38105648
BnNACs	BnaC09g04330D	0.28644682	0.332113671	1.595115217	0.65899644	0.026249899	0.224398094	-0.1787395	0.30192152	0.03522625	0.23910449
BnNACs	BnaC08g21160D	0.78358018	0.479937212	1.649841337	0.6535586	-0.321928095	0.322061362	0.13348887	0.28034626	-0.6239135	0.40443702
BnNACs	BnaC09g50530D	0.21586142	0.326728663	2.032324938	0.75321207	-0.454823318	0.415405175	0.03221874	0.24215145	0.08231735	0.26516432
BnNACs	BnaA03g01250D	0.16121607	0.311872556	2.059417201	0.77260559	-0.237790236	0.348682588	-0.0049006	0.22574104	0.3248077	0.38825778
BnNACs	BnaC03g71220D	-0.1545933	0.303002483	2.428249475	0.79760867	0.208428527	0.329167988	0.10606681	0.29436798	0.42136025	0.42443816
BnNACs	BnaA03g04240D	-0.5365128	0.381186375	2.44864103	0.75413673	1.970800024	0.731901	1.76816022	0.73589683	0.96070885	0.53249058
BnNACs	BnaA05g18430D	0.25694524	0.272004562	2.690388855	0.66851818	-0.747419952	0.316498027	-0.8945736	0.3520928	-0.2008528	0.26302001
BnNACs	BnaA02g05540D	-3.6457165	0.518457853	2.748815312	0.71105226	0.036711778	0.220651044	0.9925442	0.47215031	1.10225984	0.47146122
BnNACs	BnaC04g31690D	0.21444367	0.267982317	2.822468695	0.69903836	0.600294154	0.334722516	1.77799286	0.61531755	1.76035744	0.57827856
BnNACs	BnaA06g21090D	1.15432815	0.476080084	2.848858474	0.71947982	0.643235542	0.357325901	0.19555081	0.27249111	-0.2630344	0.27869509
BnNACs	BnaA10g00280D	0.51898961	0.466890785	2.866433871	0.82415284	0.63317529	0.521456374	0.71446022	0.58725651	1.13537429	0.66717523
BnNACs	BnaC01g40260D	0.52846761	0.306115485	2.888602981	0.66524226	1.136294224	0.416083254	0.16326127	0.25487425	0.66890132	0.33206964
BnNACs	BnaC03g52400D	0.47537946	0.409357498	2.917039778	0.80927912	-0.468516381	0.393079186	-0.1002671	0.27369095	-0.8016568	0.48117008
BnNACs	BnaA03g02640D	0.96224508	0.59406152	2.937673315	0.82063035	0.000443565	0.21244364	-0.3564988	0.40544093	0.55417348	0.4605437
BnNACs	BnaC05g28020D	0.90802168	0.351599651	3.08246216	0.6599321	-0.280107919	0.24683196	0.27710959	0.26521716	-0.3541085	0.26728442
BnNACs	BnaA06g31080D	1.05658353	0.379700324	3.288569498	0.68965312	-0.222392421	0.241380905	-1.7507714	0.37171087	0.87521838	0.35016864
BnNACs	BnaA05g24050D	0.99284021	0.499051129	3.782064471	0.8355437	-0.520122568	0.345961464	-0.7073432	0.40995632	0.56510073	0.38644369
BnNACs	BnaC05g38150D	1.67500811	0.582930448	4.09078269	0.8334258	0.055424039	0.226687714	-0.3285351	0.29378677	0.3286925	0.2975162
BnNACs	BnaC07g25530D	0	0.22451918	4.271887412	0.69176882	0.944290567	0.282699725	-0.0220263	0.22393577	2.15527823	0.43419987
BnNACs	BnaC06g12550D	-0.0471239	0.235302406	4.627399414	0.87575073	-0.066984334	0.232134365	0.04563323	0.23955581	1.12280109	0.50908362
BnNACs	BnaA07g14730D	-0.0740006	0.228660309	6.40599236	0.86814655	0.867896464	0.255241907	2.3291236	0.40994972	2.18586655	0.37858634

BnNACs	BnaA02g01460D	0. 3448047	0. 390804977	0. 383551727	0. 3943781	1. 376084774	0. 717141763	0. 00560271	0. 22984915	0. 39886204	0. 41682298
BnWRKYs	BnaC03g21360D	1. 40348621	0. 685941298	2. 151133254	0. 75980794	0. 29339319	0. 350934781	−0. 9017492	0. 57468562	0. 09775082	0. 27265622
BnWRKYs	BnaC04g41050D	1. 99500179	0. 667049738	3. 567753211	0. 80674073	0. 133350387	0. 24909516	0. 49678669	0. 36405826	0. 73468285	0. 40517894
BnWRKYs	BnaCnng66020D	2. 30102632	0. 650298091	2. 437709326	0. 64020175	−0. 597367522	0. 303169802	−1. 6820299	0. 42874661	−1. 3194598	0. 38510955
BnWRKYs	BnaC04g06800D	3. 68642049	0. 810551622	4. 414328859	0. 85050988	−0. 703018262	0. 32811124	−0. 0848889	0. 24530188	−0. 1069152	0. 24998899
BnWRKYs	BnaC01g18810D	−0. 0590106	0. 249920744	−1. 480495298	0. 60310552	0. 354125011	0. 375369862	−0. 0572573	0. 25727394	0. 16379943	0. 299775
BnWRKYs	BnaC05g10200D	0. 99258676	0. 518671404	1. 589337622	0. 62129918	−0. 800797206	0. 421673007	−0. 3836144	0. 34726258	0. 13929495	0. 27428317
BnWRKYs	BnaA05g12160D	0. 12909673	0. 276370249	1. 813859838	0. 69698871	−0. 217743062	0. 300459685	−0. 4562917	0. 40634577	0. 1793116	0. 29805558
BnWRKYs	BnaA03g17820D	0. 70446295	0. 460640565	1. 95894984	0. 70301437	0. 138368968	0. 269041266	−0. 7694682	0. 47438709	−0. 1933254	0. 29415663
BnWRKYs	BnaA06g08890D	1. 04750767	0. 581411374	2. 056996277	0. 73142767	−0. 240845487	0. 309294533	−0. 0856751	0. 2666812	0. 10768164	0. 27172937
BnWRKYs	BnaA04g22040D	2. 50200971	0. 612939871	2. 990587676	0. 64934482	−1. 284881108	0. 326693438	−1. 0595888	0. 33265305	−0. 9427751	0. 31541706
BnWRKYs	BnaC03g16740D	2. 42844175	0. 586237187	3. 64160608	0. 71858819	−1. 566346823	0. 329625911	−2. 4088055	0. 37971794	−0. 4854268	0. 27638126
BnWRKYs	BnaC02g01720D	0. 94404359	0. 364401705	3. 649490502	0. 7388315	0. 470791481	0. 280991317	0. 39530128	0. 28424082	1. 12389647	0. 39962662
BnWRKYs	BnaA04g17420D	1. 21674586	0. 448036652	4. 007377733	0. 80342078	0. 238404739	0. 255307954	0. 17492568	0. 25918269	−0. 2318157	0. 26578516
BnWRKYs	BnaA03g13820D	2. 94394419	0. 568263676	4. 600275556	0. 75959659	−0. 965234582	0. 262445401	−0. 0356239	0. 22858105	0. 2274105	0. 24937256
BnWRKYs	BnaC09g13680D	1. 60319434	0. 663582285	2. 41666613	0. 74597335	0. 664793524	0. 440923421	0. 47566833	0. 40555981	0. 94312674	0. 51930986
BnWRKYs	BnaC02g26030D	3. 92286123	0. 84901062	3. 259721672	0. 78668895	−0. 002649578	0. 21244364	−0. 0622064	0. 24374097	−0. 3896727	0. 31314946
BnWRKYs	BnaAnng23990D	4. 6206714	0. 824472067	4. 656347134	0. 81445498	0. 913843356	0. 32867704	−0. 3670575	0. 26224946	0. 52083216	0. 28769726
BnWRKYs	BnaA07g35260D	6. 290791	0. 949762672	4. 195475295	0. 83834628	−0. 117753531	0. 240438638	−0. 6125182	0. 340211	−0. 7176003	0. 34173227
BnWRKYs	BnaA09g13370D	1. 18422366	0. 591527528	2. 180073609	0. 72763438	0. 42722804	0. 370768608	0. 27158899	0. 33474893	0. 35347317	0. 34619043
BnWRKYs	BnaC03g67380D	−1. 9292767	0. 516181444	−3. 654416835	0. 56074976	−2. 246758866	0. 538798038	−3. 1193796	0. 60445287	−1. 7377452	0. 50718368
BnWRKYs	BnaC07g27240D	1. 66124609	0. 511412871	2. 50326814	0. 61418155	−0. 448052557	0. 272590176	−0. 1368509	0. 2500044	−1. 3284709	0. 35974982
BnWRKYs	BnaC02g40180D	1. 77760758	0. 479184279	2. 99138687	0. 62791926	−0. 485426827	0. 260001673	−0. 4717519	0. 27443288	0. 95007477	0. 35050988
BnWRKYs	BnaC04g14500D	1. 11703942	0. 410365811	3. 838937567	0. 7733299	−1. 449307401	0. 353757177	−0. 0067892	0. 22393577	0. 37381484	0. 28426283
BnWRKYs	BnaC03g67520D	2. 19924558	0. 662965849	2. 321143808	0. 65076702	−0. 035721922	0. 220651044	0. 29342167	0. 29400252	−0. 0972972	0. 24868787
BnWRKYs	BnaA04g13570D	0. 87034568	0. 541660062	1. 834777618	0. 71098181	0. 040992571	0. 231240533	−1. 1378006	0. 60303727	−0. 2358199	0. 32008877
BnWRKYs	BnaC04g35770D	1. 21447855	0. 579172391	1. 901196988	0. 67514927	0. 227344726	0. 292239071	−0. 711929	0. 43497041	−0. 0778271	0. 2525428
BnWRKYs	BnaA08g12420D	1. 07091724	0. 56453644	2. 157966049	0. 72629804	−1. 289643515	0. 539920832	−0. 5954729	0. 42875321	−0. 5769765	0. 39688568
BnWRKYs	BnaA03g46550D	0. 06370284	0. 245378932	0. 587418152	0. 40359822	−0. 152822574	0. 270137641	−1. 6503222	0. 61551129	−0. 4744092	0. 36921211
BnWRKYs	BnaC06g15910D	1. 27076163	0. 642339462	−0. 010279592	0. 22960257	−1. 073544485	0. 551183997	−0. 6095974	0. 46720781	0. 63071694	0. 46373816
BnWRKYs	BnaA07g16850D	1. 31782423	0. 677000775	0. 309393416	0. 35036678	−1. 167714687	0. 613926169	−0. 4048286	0. 42183152	0. 0739773	0. 2600347
BnWRKYs	BnaC02g09670D	1. 48330952	0. 625394079	−1. 097297201	0. 44149803	−1. 112286376	0. 474902251	−1. 4889158	0. 5511884	0. 08787276	0. 25527273
BnWRKYs	BnaA01g13440D	1. 70159304	0. 733699672	−0. 23672179	0. 31567685	−0. 815153293	0. 518173853	−1. 7842096	0. 71020025	−1. 7096351	0. 6695485
BnWRKYs	BnaC01g15640D	2. 18465538	0. 762890116	−0. 056709442	0. 24540755	−0. 206047383	0. 293553401	−1. 2754688	0. 58815034	−1. 8215896	0. 61518326
BnWRKYs	BnaCnng52600D	2. 59735905	0. 804902427	−0. 741171705	0. 43221186	−2. 663565135	0. 689448378	−1. 4105445	0. 62210495	−0. 8375945	0. 47909622
BnWRKYs	BnaA03g46280D	2. 62385151	0. 673575152	−1. 407175382	0. 35238781	0. 219009782	0. 251149213	−0. 2442368	0. 26594588	−0. 2442368	0. 26604495
BnWRKYs	BnaA04g02560D	2. 99794341	0. 831996988	−4. 035788329	0. 72362535	−2. 069896183	0. 666508155	−1. 1866683	0. 6012496	−0. 1324977	0. 28046735
BnWRKYs	BnaC07g30300D	3. 68345236	0. 68361205	−0. 690671942	0. 26310587	−2. 353636955	0. 309512487	−2. 2756344	0. 32353861	−0. 2947433	0. 2525384
BnWRKYs	BnaC08g27340D	−0. 1950884	0. 327164571	−1. 044346651	0. 59420022	−1. 095770152	0. 648237434	0. 13805579	0. 31395743	0. 19124405	0. 32539232
