# Supplementary material for: Exploring transcription factors reveals crucial members and regulatory networks involved in different abiotic stresses in Brassica napus L
Source: BMC Plant Biol. 2018 Sep 19;18:202. doi: 10.1186/s12870-018-1417-z (PMC6146658; doi:10.1186/s12870-018-1417-z)
Supplement: Supplementary file 6 — Detailed information for the 153 common DEGs. (PDF 61 kb) [file 12870_2018_1417_MOESM6_ESM.pdf]

Additional file 6 The153 Common DEGs					
153 Common DEGs	Family	group	chromosome	Homologous genes	Response
BnaA01g02720D	AP2/EREBPs	ERFB3	A01	AT4G344100	Heat
BnaA01g13420D	AP2/EREBPs	ERFB5	A01	AT4G237500	Heat;salt
BnaA01g27570D	AP2/EREBPs	ERFB2	A01	AT3G167700	Heat
BnaA02g18720D	AP2/EREBPs	DREBA6	A02	AT1G780800	Heat
BnaA03g04290D	AP2/EREBPs	ERFB4	A03	AT5G133300	Cold;heat
BnaA03g33790D	AP2/EREBPs	ERFB1	A03	AT3G152100	Cold;heat
BnaA05g23130D	AP2/EREBPs	ERFB2	A05	AT3G167700	Heat
BnaA06g40170D	AP2/EREBPs	ERFB3	A06	AT5G472300	Heat
BnaA07g08440D	AP2/EREBPs	ERFB1	A07	AT1G03800;AT5G44210;AT1G80580	Heat
BnaA07g13990D	AP2/EREBPs	AP2	A07	AT2G285500	Cold;heat
BnaA09g30810D	AP2/EREBPs	DREBA6	A09	AT1G221900	Heat
BnaA10g25000D	AP2/EREBPs	DREBA2	A10	AT2G403500	Heat
BnaAnng21280D	AP2/EREBPs	ERFB3	Ann	AT2G44840;AT3G232300	Heat
BnaC01g10100D	AP2/EREBPs	ERFB3	C01	AT4G17500;AT2G44840;AT3G23230	Heat
BnaC03g05820D	AP2/EREBPs	ERFB4	C03	AT5G133300	Cold;heat
BnaC03g39000D	AP2/EREBPs	ERFB1	C03	AT3G152100	Cold;heat
BnaC05g17550D	AP2/EREBPs	DREBA6	C05	AT1G221900	Heat
BnaC05g38410D	AP2/EREBPs	ERFB1	C05	AT3G152100	Cold;heat
BnaC05g42130D	AP2/EREBPs	DREBA2	C05	AT3G110200	Cold;heat
BnaC07g45030D	AP2/EREBPs	ERFB3	C07	AT4G344100	Heat
BnaC09g49920D	AP2/EREBPs	DREBA2	C09	AT2G403500	Heat
BnaCnng67070D	AP2/EREBPs	ERFB1	Cnn	AT1G03800;AT5G44210;AT1G80580	Heat
BnaC07g27220D	BnbZIPs	VIII	C07	AT5G494500	Cold;heat
BnaA06g29500D	BnbZIPs	VIII	A06	AT5G494500	Cold;heat
BnaA06g29270D	BnbZIPs	VII	A06	AT5G287700	Cold
BnaC07g27440D	BnbZIPs	VII	C07	AT5G287700	Cold
BnaA01g27940D	BnbZIPs	III	A01	AT3G176090	Cold;heat
BnaA02g02830D	BnbZIPs	XII	A02	AT5G158300	Heat
BnaC02g06270D	BnbZIPs	XII	C02	AT5G158300	Heat
BnaA03g34610D	BnbZIPs	III	A03	AT3G176090	Heat
BnaA05g22650D	BnbZIPs	III	A05	AT3G176090	Heat
BnaA04g05810D	BnbZIPs	I	A04	AT3G519600	Heat
BnaCnng20200D	BnbZIPs	III	Cnn	AT5G112600	Heat
BnaC09g45380D	BnbZIPs	III	C09	AT5G112600	Heat
BnaC05g51490D	BnbZIPs	III	C05	AT1G437000	Heat
BnaA09g39870D	BnbZIPs	X	A09	AT3G624200	Heat
BnaC08g32220D	BnbZIPs	X	C08	AT3G624200	Heat
BnaA05g01520D	BnbZIPs	VII	A05	AT2G462700	Heat;salt;ABA
BnaA01g31420D	BnbZIPs	III	A01	AT3G108000	Heat
BnaA09g00170D	BnbZIPs	VII	A09	AT4G011200	Heat;drought
BnaAnng39310D	BnbZIPs	III	Ann	AT1G437000	Heat
BnaC04g33670D	BnbZIPs	III	C04	AT2G212300	Heat
BnaC04g01070D	BnbZIPs	VII	C04	AT2G462700	Heat;salt;ABA
BnaA05g08020D	BnbZIPs	V	A05	AT2G362700	Heat
BnaAnng26550D	BnbZIPs	V	Ann	AT4G340000	Heat;salt;ABA
BnaC06g18530D	BnbZIPs	X	C06	AT3G624200	Heat
BnaA07g19330D	BnbZIPs	X	A07	AT3G624200	Heat
BnaC07g44670D	BnbZIPs	V	C07	AT4G340000	Heat;salt;ABA
BnaA01g02570D	BnbZIPs	X	A01	AT4G345900	ABA
BnaC01g03810D	BnbZIPs	X	C01	AT4G345900	ABA
BnaA01g26200D	BnbZIPs	V	A01	AT3G192900	ABA
BnaC03g03620D	BnMYBs	R2R3	C03	AT5G08490;AT5G08510;AT5G08520	Cold
BnaA01g00670D	BnMYBs	R2R3	A01	AT4G372600	Cold
BnaC07g13600D	BnMYBs	R2R3	C07	AT1G226400	Cold;heat
BnaC01g01660D	BnMYBs	R2R3	C01	AT4G372600	Cold
BnaC05g43350D	BnMYBs	1R	C05	AT3G096000	Cold;heat
BnaA01g32200D	BnMYBs	1R	A01	AT3G09600;AT5G576500	Cold;heat
BnaA06g12480D	BnMYBs	1R	A06	AT1G18330;AT3G101130	Cold
BnaA05g28870D	BnMYBs	1R	A05	AT3G096000	Cold;heat
BnaA07g10350D	BnMYBs	R2R3	A07	AT1G226400	Cold;heat
BnaC09g40660D	BnMYBs	1R	C09	AT5G173000	Cold;heat
BnaC07g45320D	BnMYBs	R2R3	C07	AT4G349900	Cold
BnaA05g01050D	BnMYBs	1R	A05	AT2G468300	Cold;heat
BnaC02g07210D	BnMYBs	1R	C02	AT5G173000	Cold;heat

BnaAnng35540D	BnMYBs	1R	Ann	AT3G163500	Cold
BnaC05g00840D	BnMYBs	1R	C05	AT1G010600	Cold;heat
BnaA10g00430D	BnMYBs	1R	A10	AT1G015200	Cold;ABA
BnaC01g00270D	BnMYBs	1R	C01	AT4G392500	Cold
BnaC04g00590D	BnMYBs	1R	C04	AT2G468300	Cold;heat
BnaC05g00500D	BnMYBs	1R	C05	AT1G015200	Cold;ABA
BnaCnng76050D	BnMYBs	R2R3	Cnn	AT2G167200	Cold
BnaA03g29470D	BnMYBs	1R	A03	AT3G06490;AT1G48000;AT5G49620	Cold
BnaC01g03510D	BnMYBs	R2R3	C01	AT4G349900	Cold
BnaA03g06490D	BnMYBs	1R	A03	AT5G173000	Cold;heat
BnaA03g53110D	BnMYBs	R2R3	A03	AT4G349900	Cold
BnaCnng65380D	BnMYBs	1R	Cnn	AT5G076900	Heat
BnaAnng17890D	BnMYBs	1R	Ann	AT1G010600	Heat
BnaA03g00640D	BnMYBs	1R	A03	AT3G09600;AT1G01520;AT5G02840;AT5G57650	Heat
BnaA10g00780D	BnMYBs	1R	A10	AT1G010600	Heat
BnaC09g41280D	BnMYBs	R2R3	C09	AT5G166000	Heat
BnaA10g17370D	BnMYBs	1R	A10	AT5G173000	Heat
BnaA10g17890D	BnMYBs	R2R3	A10	AT5G166000	Heat
BnaC03g60080D	BnMYBs	R2R3	C03	AT4G386200	Heat
BnaA10g24770D	BnMYBs	R2R3	A10	AT5G057900	Heat
BnaA08g16990D	BnMYBs	R2R3	A08	AT4G386200	Heat;ABA
BnaA10g26900D	BnMYBs	1R	A10	AT5G028400	Heat
BnaC05g14070D	BnMYBs	1R	C05	AT1G18330;AT3G101130	Heat
BnaC05g17910D	BnMYBs	R2R3	C05	AT1G226400	Heat;ABA
BnaA03g42350D	BnMYBs	1R	A03	AT3G168570	Heat
BnaA06g05610D	BnMYBs	1R	A06	AT1G097100	Heat
BnaA09g30490D	BnMYBs	R2R3	A09	AT1G226400	Heat;ABA
BnaA07g29070D	BnMYBs	1R	A07	AT1G710300	Heat;ABA
BnaA03g24010D	BnMYBs	R2R3	A03	AT4G094600	Heat
BnaA03g01420D	BnMYBs	R2R3	A03	AT5G04760;AT3G105800	Heat
BnaAnng01040D	BnMYBs	1R	Ann	AT5G04760;AT3G105800	Heat
BnaC03g28550D	BnMYBs	R2R3	C03	AT4G094600	Heat
BnaA07g24010D	BnMYBs	1R	A07	AT1G700000	Heat
BnaC03g01730D	BnMYBs	R2R3	C03	AT5G047600	Heat
BnaC02g02590D	BnMYBs	1R	C02	AT3G10580;AT5G047600	Heat
BnaA08g22580D	BnMYBs	R2R3	A08	AT1G185700	ABA
BnaC09g05650D	BnMYBs	R2R3	C09	AT5G624700	ABA
BnaC09g43890D	BnNACs	VI	C09	AT5G131800	Cold
BnaA07g24270D	BnNACs	VI	A07	AT1G694900	Cold
BnaA10g20110D	BnNACs	VI	A10	AT5G131800	Cold;heat
BnaC03g03740D	BnNACs	VI	C03	AT5G63790;AT5G087900	Cold;heat
BnaC05g20530D	BnNACs	III	C05	AT1G255800	Cold
BnaA03g48570D	BnNACs	VI	A03	AT4G274100	Cold;heat;salt;ABA
BnaC02g42720D	BnNACs	VI	C02	AT5G637900	Cold;heat;salt
BnaC07g40860D	BnNACs	VI	C07	AT4G274100	Cold;heat;ABA
BnaA02g33910D	BnNACs	VI	A02	AT5G637900	Cold;heat;salt
BnaC02g00990D	BnNACs	VI	C02	AT5G63790;AT5G087900	Cold;heat;drought
BnaC05g00370D	BnNACs	VI	C05	AT1G017200	Cold;heat
BnaCnng64100D	BnNACs	VI	Cnn	AT1G528800	Cold
BnaAnng05950D	BnNACs	VI	Ann	AT5G63790;AT5G08790;AT1G01720	Cold;heat
BnaA10g22680D	BnNACs	VI	A10	AT5G087900	Cold;heat
BnaC07g13550D	BnNACs	VIII	C07	AT1G328700	Cold;heat
BnaC09g47250D	BnNACs	VI	C09	AT5G087900	Cold;heat
BnaA07g10300D	BnNACs	VIII	A07	AT1G328700	Cold;heat
BnaC03g50570D	BnNACs	VI	C03	AT5G63790;AT1G017200	Heat;drought;salt
BnaA09g04810D	BnNACs	VII	A09	AT3G49530;AT5G245900	Heat
BnaC09g04330D	BnNACs	VII	C09	AT3G49530;AT5G245900	Heat
BnaC08g21160D	BnNACs	VII	C08	AT3G49530;AT5G245900	Heat
BnaC09g50530D	BnNACs	VIII	C09	AT3G10500;AT5G044100	Heat
BnaC03g71220D	BnNACs	VIII	C03	AT3G10500;AT5G044100	Heat
BnaC04g31690D	BnNACs	IX	C04	AT5G396100	Heat;ABA
BnaA06g21090D	BnNACs	VII	A06	AT3G49530;AT5G245900	Heat
BnaA10g00280D	BnNACs	VI	A10	AT1G017200	Heat;salt
BnaC01g40260D	BnNACs	IX	C01	AT5G18270;AT3G040600	Heat
BnaC03g52400D	BnNACs	VII	C03	AT3G49530;AT5G245900	Heat
BnaA03g02640D	BnNACs	VI	A03	AT5G087900	Heat

BnaA05g24050D	BnNACs	VI	A05	AT3G155000	Heat
BnaC05g38150D	BnNACs	VI	C05	AT3G155000	Heat
BnaC03g21360D	BnWRKYs	I	C03	AT2G384700	Cold;heat
BnaC04g41050D	BnWRKYs	I	C04	AT2G302500	Cold;heat
BnaC04g06800D	BnWRKYs	I	C04	AT2G384700	Cold;heat
BnaC05g10200D	BnWRKYs	I	C05	AT1G13960;AT2G033400	Heat
BnaA05g12160D	BnWRKYs	I	A05	AT2G302500	Heat
BnaA03g17820D	BnWRKYs	I	A03	AT2G384700	Heat
BnaA06g08890D	BnWRKYs	I	A06	AT1G139600	Heat
BnaC02g01720D	BnWRKYs	I	C02	AT5G071000	Heat
BnaA04g17420D	BnWRKYs	I	A04	AT2G302500	Heat
BnaA03g13820D	BnWRKYs	I	A03	AT2G302500	Heat
BnaC09g13680D	BnWRKYs	IIb	C09	AT1G623000	Cold;heat
BnaC02g26030D	BnWRKYs	IIb	C02	AT1G808400	Cold;heat
BnaA09g13370D	BnWRKYs	IIb	A09	AT1G623000	Heat
BnaC02g40180D	BnWRKYs	IIc	C02	AT5G261700	Heat
BnaC04g14500D	BnWRKYs	IIc	C04	AT2G302500	Heat
BnaC03g67520D	BnWRKYs	IId	C03	AT2G24570;AT4G31550;AT4G24240;AT2G23320	Cold;heat
BnaA04g13570D	BnWRKYs	IId	A04	AT2G233200	Heat;ABA
BnaC04g35770D	BnWRKYs	IId	C04	AT2G233200	Heat
BnaA08g12420D	BnWRKYs	IId	A08	AT4G31550;AT2G24570;AT4G24240	Heat
BnaC06g15910D	BnWRKYs	III	C06	AT3G564000	Cold
BnaA07g16850D	BnWRKYs	III	A07	AT3G564000	Cold;drought
